# Identification of chilling stress-responsive tomato microRNAs and their target genes by high-throughput sequencing and degradome analysis

**DOI:** 10.1186/1471-2164-15-1130

**Published:** 2014-12-17

**Authors:** Xue Cao, Zhen Wu, Fangling Jiang, Rong Zhou, Zeen Yang

**Affiliations:** Key Laboratory of Horticultural Plant Biology and Germplasm Innovation in East China, Ministry of Agriculture, College of Horticulture, Nanjing Agricultural University, Nanjing, 210095 P.R. China

## Abstract

**Background:**

MicroRNAs (miRNAs) are a class of noncoding small RNAs (sRNAs) that are 20–24 nucleotides (nt) in length. Extensive studies have indicated that miRNAs play versatile roles in plants, functioning in processes such as growth, development and stress responses. Chilling is a common abiotic stress that seriously affects plants growth and development. Recently, chilling-responsive miRNAs have been detected in several plant species. However, little is known about the miRNAs in the model plant tomato. ‘LA1777’ (*Solanum habrochaites*) has been shown to survive chilling stress due to its various characteristics.

**Results:**

Here, two small RNA libraries and two degradome libraries were produced from chilling-treated (CT) and non-chilling-treated (NT) leaves of *S. habrochaites* seedlings. Following high-throughput sequencing and filtering, 161 conserved and 236 novel miRNAs were identified in the two libraries. Of these miRNAs, 192 increased in the response to chilling stress while 205 decreased. Furthermore, the target genes of the miRNAs were predicted using a degradome sequencing approach. It was found that 62 target genes were cleaved by 42 conserved miRNAs, while nine target genes were cleaved by nine novel miRNAs. Additionally, nine miRNAs and six target genes were validated by quantitative real-time PCR (qRT-PCR). Target gene functional analysis showed that most target genes played positive roles in the chilling response, primarily by regulating the expression of anti-stress proteins, antioxidant enzyme and genes involved in cell wall formation.

**Conclusions:**

Tomato is an important model plant for basic biological research. In this study, numerous conserved and novel miRNAs involved in the chilling response were identified using high-throughput sequencing, and the target genes were analyzed by degradome sequencing. The work helps identify chilling-responsive miRNAs in tomato and increases the number of identified miRNAs involved in chilling stress. Furthermore, the work provides a foundation for further study of the regulation of miRNAs in the plant response to chilling stress.

**Electronic supplementary material:**

The online version of this article (doi:10.1186/1471-2164-15-1130) contains supplementary material, which is available to authorized users.

## Background

Chilling, a type of low temperature stress, is a common abiotic environmental stress that seriously affects the growth and development of plants [1]. Chilling stress has attracted increasing attention. To date, many studies about plant morphological and physiological responses to chilling stress have been reported [2, 3]. In addition, genes related to the chilling response have been identified, such as genes encoding the transcription factors DREB/CBF [4], WRKY [5], MYB [6] and so on.

In recent years, microRNAs (miRNAs) have become a research hotspot [7–9]. MiRNAs are a distinct class of tiny noncoding RNAs approximately 21 nucleotides (nt) in length [10, 11]. The RNAs participate in regulating gene expression at the posttranscriptional level in both plants and animals [12, 13]. Prior to their regulation of gene expression, miRNAs are generated from hairpin precursors by dicer-like (DCL) into miRNA::miRNA* duplexes [14]. The miRNA* strand is then degraded, the mature miRNA joins with argonaute (AGO) and the RNA-induced silencing complex (RISC) is generated. Finally, the silencing complex targets protein-coding mRNAs by cleaving the mRNAs at specific positions or by repressing translation [12, 15].

Since the discovery of miRNA genes in the nematode *Caenorhabditis elegans* 20 years ago [16], miRNAs have been intensively studied. Notably, in recent years, new generation sequencing technology has led to the identification of numerous miRNAs, especially novel and low copy number miRNAs, in many plant species, such as wild soybean [17], rape [18], radish [19], *Medicago truncatula* [20] and so on. In addition, the interaction between miRNAs and target genes has received much attention. Degradome sequencing is an emerging technology used to predict and verify target genes of miRNAs. Compared with traditional methods, i.e., bioinformatics prediction and 5’ RACE, degradome sequencing is rapid and effective [17, 21]. With the utilization of the two high-sequencing approaches, numerous miRNAs have been identified, and great progress has been made in elucidating the interaction between miRNAs and target genes, especially chilling stress-related miRNAs. For example, many chilling-responsive miRNAs have been detected in *Arabidopsis* [22, 23], *Brachypodium* [24], *Prunus persica* [25], *Populus tomentosa* [26], *Oryza sativa* [27] and *Zea may* [28]. Studies of the functions of miRNAs have been carried out simultaneously.

Tomato is a globally important vegetable of the Solanum family [29]. Although tomato is widely grown in various temperature zones, its growth and development are rather sensitive to temperature stress, including extreme chilling stress [3]. However, most cultivated and commercial tomato cultivars are considered to be sensitive to chilling stress [3, 30].

With the completion of the tomato genome sequence [31], tomato has become a model system for research on the interaction between miRNAs and their target genes. Although many tomato miRNAs have been identified by traditional Sanger cloning, deep sequencing and bioinformatics approach [29, 32–35], chilling-responsive miRNAs in tomato have not been reported.

‘LA1777’ (*S. habrochaites*) is one kind of wild tomato. And it exhibits higher chilling-tolerance ability than other cultivars in our previous research, which is in agreement with other studies [36, 37]. In this study, using ‘LA1777’, two small RNA libraries from leaves treated with and without chilling stress (4°C) were constructed for high-throughput Solexa sequencing. Then 4,342,604 and 7,231,609 clean reads were obtained from the two libraries, respectively. The results showed that small RNAs (sRNAs) 21 and 24 nt in length were the most abundant classes. A total of 161 conserved and 236 novel miRNAs were identified in the two libraries. Based on the abundance of miRNAs, the expression amounts between the two libraries were compared, 192 increased and 205 decreased miRNAs were detected in the chilling stress. In addition, degradome sequencing analysis was adopted to identify target genes of the miRNAs. It was found that 62 target genes were cleaved by 42 conserved miRNAs and nine target genes were cleaved by nine novel miRNAs. The verification of nine miRNAs and six target genes by quantitative real-time PCR (qRT-PCR) basically confirmed the sequencing results. Target gene functional analysis showed most target genes were involved in the defense response through regulating the expression of anti-stress proteins, antioxidant enzyme and genes involved in cell wall formation. In addition to identify chilling-responsive conserved and novel miRNAs in tomato, the work lays the foundation for further elucidating the regulation of miRNAs in response to chilling stress.

## Results

### Analysis of small RNA data from the libraries

To identify miRNAs from tomato that respond to chilling stress, two small RNA libraries from tomato leaves treated with and without chilling stress were constructed and sequenced using an Illumina Genome Analyzer II (LC Sciences, Hangzhou, China). A total of 6,199,678 and 9,919,778 raw reads were generated from the chilling-treated (CT) library and non-chilling-treated (NT) library, respectively (Table 1). After removing 3’ and 5’ adaptors, sequences with <15 nt and >29 nt and junk reads, the remaining reads were searched against the Rfam (http://rfam.xfam.org/), repeat databases (http://www.girinst.org/repbase/update/index.html) and tomato genome sequence (http://solgenomics.net); 13.71% and 12.59% (reads/reads) of total sRNAs in the CT and NT library were filtered out, respectively. Finally, 4,342,604 and 7,231,609 clean reads corresponding to 1,208,259 and 1,795,513 unique reads were obtained from the two libraries, respectively. The number and percentage of various sRNAs were listed in Table 1.

**Table 1:** **Statistics of sRNA sequences from the CT and NT libraries**

Type	NT				CT			
	Number of total sRNA	Percentage of total (%)	Number of unique sRNA	Percentage of unique (%)	Number of total sRNA	Percentage of total (%)	Number of unique sRNA	Percentage of unique (%)
Raw reads	9,919,778	100.00	2,280,543	100.00	6,199,678	100.00	1,521,423	100.00
3ADT&length filter^a^	1,432,000	14.44	333,989	14.65	1,009,234	16.28	196,123	12.89
Junk reads	27,989	0.28	14,986	0.66	14,948	0.24	8,225	0.54
Rfam^b^	1,181,057	11.91	118,282	5.19	795,804	12.84	95,041	6.25
mRNA	4,132	0.04	2,480	0.11	3,320	0.05	2,007	0.13
Repeats^c^	63,383	0.64	20,334	0.89	50,626	0.82	16,844	1.11
rRNA	860,830	8.68	72,264	0.73	524,172	8.45	53,070	0.86
tRNA	197,083	1.99	19,182	0.19	184,888	2.98	19,919	0.32
snoRNA	17,886	0.18	5,419	0.05	17,466	0.28	4,893	0.08
snRNA	15,389	0.16	5,618	0.06	12,882	0.21	4,598	0.07
other Rfam RNA	89,869	0.91	15,799	0.16	56,396	0.91	12,561	0.20
Clean reads	7,231,609	72.90	1,795,513	78.73	4,342,604	70.05	1,208,259	79.42

^a^reads lacking three ADTs or with lengths <17 nt or >25 nt were removed.

^b^collection of many common noncoding RNA families other than micro RNAs; http://rfam.janelia.org.

^c^downloaded from http://www.girinst.org/repbase.

**Figure 1:**
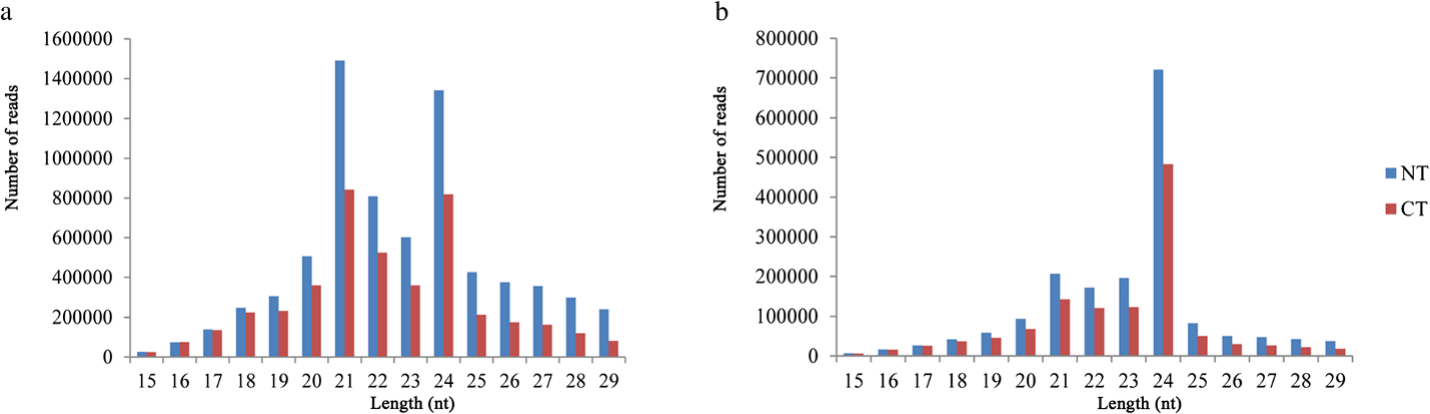
**Length distribution of sRNAs in two libraries from tomato.** **a** size distribution of total sequences. **b** size distribution of unique sequences.

The length distribution of the sRNAs from the two libraries ranged from 15 to 29 nt, as shown in Figure 1. The proportion of 19–25 nt sequences was high in both libraries, comprising over 75% of the sequences; sRNAs 21 nt and 24 nt in length were the two main sRNA classes among the sequences. Among unique sequences, 24 nt sRNA was the most abundant category, which was in agreement with previous reports on cucumber [38], grapevine [39] and rice [40].

### Identification of conserved miRNAs from tomato

**Figure 2:**
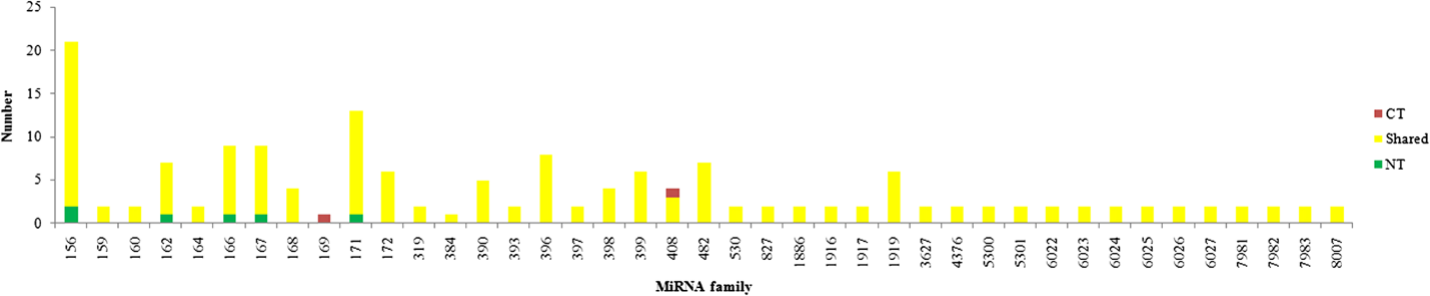
**Numbers of conserved miRNAs in different miRNA families in tomato.**

To detect conserved miRNAs from the two tomato libraries, all clean reads were aligned against mature plant miRNAs and the precursors in miRBase 20.0 (June 2013), allowing for a maximum of one mismatch in the first 16 nt of the miRNA and three mismatches in total between the target miRNAs and the known miRNAs. Finally, 161 conserved miRNAs belonging to 41 miRNA families were identified in the two libraries (Additional file 1: Table S1, Additional file 2: Figure S1). Interestingly, the number of miRNAs varied among families (Figure 2). The largest family was the 156 miRNA family with 21 members, including two members only identified in the NT library and the remaining 19 ones presented in both two libraries, followed by the 171 miRNA family, while the 169 and 384 miRNA families were the smallest, each containing only one miRNA member. In addition, the member of the 169 miRNA family and one member of 408 miRNA family were exclusively detected in the CT library. Among these, 47 miRNAs designated as p3 or p5 were identified for the first time in tomato, which comprised new flanking sequences or other sequences of known miRNAs deposited in miRBase 20.0.

**Figure 3:**
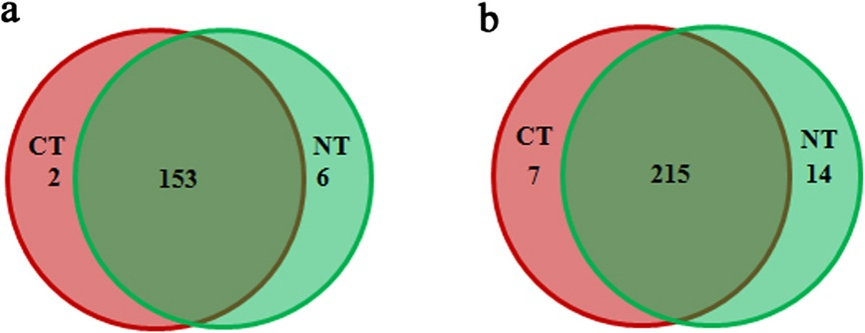
**Venn diagram of miRNAs identified in the CT (pink) and NT (green) libraries.** **a** conserved miRNAs. **b** novel miRNAs.

In the CT and NT libraries, there were 153 miRNAs in common, while two miRNAs that were sha-miR169c-3p_stu and sha-miR408a-5p_stu were only found in the CT library; six miRNAs that were sha-miR156e-p3_stu, sha-miR156f-p5_stu, sha-miR162-p5_cme, sha-miR166g-p5_nta, sha-miR167-p3 and sha-miR171h-p5_vvi were specific to the NT library (Figure 3, Additional file 1: Table S1). On the other hand, the frequency of miRNAs in the two libraries can be regarded as an index for estimating the expression amounts of miRNAs. Therefore, after sequencing, raw reads obtained by sequencing were normalized for the chi-square test, the fisher-exact test and log_2_ ratio determination between the CT and NT libraries. The result of this comparison showed that 66 miRNA members increased in response to chilling stress with log_2_ (CT/NT) > 0, whereas 95 members decreased with log_2_ (CT/NT) < 0. Only miRNAs with significant expression amounts (chi-square test and fisher-exact test) of both < 0.05 and | log_2_ (CT/NT) | ≥ 1 were considered to be significantly regulated. Among these miRNAs, 12 miRNAs that were sha-miR166a-p5, sha-miR319, sha-miR397_nta, sha-miR397-p5, sha-miR398a-3p_stu, sha-miR398a-5p_stu, sha-miR398a-p3_cme, sha-miR399-p5, sha-miR408_nta, sha-miR408a-3p_stu, sha-miR408b-5p_stu and sha-miR530a_cme significantly increased in response to chilling stress, while 20 miRNAs significantly decreased (Additional file 1: Table S1). Of the 47 newly identified miRNAs, most had lower expression amounts with norm read <100, possibly because the new flanking sequences or other miRNA sequences were theoretically more volatile than the others [13, 41].

### Identification of novel miRNAs from tomato

To identify potentially novel miRNAs from tomato, after filtering out the conserved miRNAs, sequences ranging from 19 to 24 nt were used to further analysis using strict criteria for miRNA prediction. Second structures of the pre-miRNAs were predicted by UNAfold software (http://mfold.rna.albany.edu/?q=mfold/RNA-Folding-Form) [17, 42]; only those with stable hairpin structures were considered. The minimal folding free index (MFEI) was an important feature used to distinguish miRNAs from other noncoding RNAs [43]. In this study, miRNAs with MEFIs exceeding 0.80 were regarded as candidate miRNAs. Ultimately, 236 novel miRNAs corresponding to 224 unique sequences generated from 171 precursors were discovered from tomato in the two libraries (Additional file 3: Table S2, Additional file 4: Figure S2).

As shown in a Venn diagram, 215 novel miRNAs were shared in both libraries; 14 novel miRNAs were unique to the NT library, while seven novel miRNAs were only identified in the CT library (Figure 3). Meanwhile, the expression amounts of the miRNAs between the two libraries were compared. Of the miRNAs identified, 126 miRNAs increased in response to chilling stress, while 110 miRNAs decreased. Moreover, 11 miRNAs significantly increased, while six miRNAs significantly decreased (Additional file 3: Table S2). On the other hand, compared with the conserved miRNAs, most of the novel miRNAs had low expression amounts with norm read <100, which was consistent with previous reports in *Arabidopsis* [41, 44] and wheat [45]. However, several miRNAs, such as PC-58-3p and PC-58-5p, were present at high amounts with norm read > 1,000, which was different from previous results.

### Identification of conserved miRNAs targets in tomato

To understand the function of the miRNAs, high-throughput degradome sequencing, a new technology [21, 46], was utilized to identify the miRNAs targets. In this study, 12,654,699 and 10,844,042 raw reads corresponding to 8,431,161 and 7,202,445 unique reads were initially obtained in the CT and NT library, respectively (Table 2). Following BLAST analysis, 74.14% and 64.13% of the reads could be mapped to mRNA (ftp://ftp.solgenomics.net/est_sequences/species/Tomato), respectively.

**Table 2:** **Summary of degradome library data**

Library	Raw reads	Unique raw reads	cDNA mapped reads	Total number of input cDNAs	Number of coverd cDNAs
CT	12,654,699	8,431,161	9,382,112	223,441	200,249
	-	-	74.14%	-	89.62%
NT	10,844,042	7,202,445	6,954,750	223,441	198,805
	-	-	64.13%	-	88.97%

For data analysis, CleaveLand3.0 software was used to detect the cleavage products guided by miRNAs. According to the sequencing result, the abundance of sequenced tags was plotted on every transcript, and the cleaved target transcripts were classified into five categories, namely, category 0, category 1, category 2, category 3 and category 4. For the conserved miRNAs, 281 sequences of 62 target genes from 42 conserved miRNAs were identified out of 161 conserved miRNAs (Additional file 5: Table S3). However, the targets of most miRNAs were not identified, perhaps because the abundance of cleavage products was too low to be detected or because the interaction between miRNAs and the targets did not involve cleaving.

Of these sequences, 182 sequences of target genes were shared between the two libraries, including 97 decreased, 83 increased and two constant sequences, while 88 sequences were only identified in the CT library and 11 were only found in the NT library. In the CT library, 270 sequences for conserved miRNAs were identified. The abundance ranged from eight to 3,872 tags per billion. Of these sequences, 146 belonged in category 0, while 13 sequences of conserved miRNAs were in category 1; 96 sequences were in category 2; two were in category 3 and 13 were in category 4. By contrast, 193 sequences of miRNAs were identified in the NT library with abundance ranging from nine to 7,470 tags per billion. Of these, the most abundant category was category 0 including 98 sequences. Additionally, eight, 53 and 34 sequences of conserved miRNAs were grouped into category 1, 2 and 4, respectively (Additional file 5: Table S3). All of the identified target plots (t-plots) were shown in Additional file 6: Figure S3 and additional file 7: Figure S4. Sequenced tags of targets between the two libraries were compared, and the result showed that there were more specific targets detected in the CT library than in the NT library. The result suggested that chilling stress might cause the cleavage of miRNA targets, leading to the accumulation of cleaved fragments.

Among the identified targets, more target genes were predicted to be cleaved by one miRNA. For example, *TCP2*, *TCP4* and *TRPB2* were targeted by sha-miR319b_stu. On the other hand, one target was usually cleaved by more than one miRNAs. For example, *SPL6* was predicted to be cleaved by sha-miR156a, sha-miR156c, sha-miR156c_nta, sha-miR156d_nta, sha-miR156e_stu, sha-miR156g_stu, sha-miR156h_stu, sha-miR156i_stu and sha-miR156j_stu. By contrast, only one target was found for sha-miR156d_nta and sha-miR393-5p_stu. Interestingly, several targets were cleaved by pairs of miRNAs, such as sha-miR396b_nta and sha-miR396c_nta, both of which targeted *CYSPL*, *RD21A*, *DRM1*, *CPR1*, *CYP40* and *DRM2*, which suggested that the two miRNAs might cooperate to regulate gene expression (Additional file 5: Table S3).

To understand more about the roles of miRNAs in the response to chilling stress, gene ontology (GO) and kyoto encyclopedia of genes and genomes (KEGG) pathway analysis were performed. GO analysis showed that the targets were associated with various functions (Additional file 8: Figure S5). GO enrichment analysis showed that the target genes of differentially expressed conserved miRNAs were mainly involved in metal ion binding, cytoplasm and superoxide dismutase activity. However, fewer functions were found using KEGG analysis (Additional file 9: Table S4). Gene functional analysis showed that sha-miR398_nta targeted 62 sequences of Cu/Zn superoxide dismutase (EC:1.15.1.1) gene, including 18 found in both libraries. The result suggested that the 18 sequences were not key regulators of the chilling response, while the remaining 44 sequences might play important roles in the stress response. Additionally, transcription factors and stress-responsive proteins were also targeted by miRNAs. For example, scarecrow-like protein, ribosomal protein and TCP family transcription factors targeted by sha-miR171 (sha-miR171a, sha-miR171a_nta, sha-miR171b-3p_stu, sha-miR171c_mtr and sha-miR171d), sha-miR156i-p3_nta and sha-miR319b_stu, respectively, were mainly found in the CT library, which suggested that these miRNAs might play important roles in the chilling stress response, which was in agreement with the results reported in rice [27]. On the other hand, sha-miR396 (sha-miR396a_nta and sha-miR396b_nta) directly targeted low-temperature-induced proteins that might play positive roles in the chilling response, and sha-miR160a and sha-miR167b_nta might participate in the stress response by targeting an auxin response factor gene.

### Identification of novel miRNAs targets in tomato

Like the conserved miRNAs, the targets of most novel miRNAs were not identified; out of the 236 novel miRNAs identified, only 34 sequences of nine genes were predicted for nine novel miRNAs (Table 3, Additional file 10: Table S5). Among these target sequences, 20 were identified in both libraries, including 15 decreased and five increased, while 11 were only found in the CT library and three were unique to the NT library. The abundance of the sequenced tags was plotted for every transcript (Additional file 6: Figure S3, Additional file 7: Figure S4, Additional file 10 Table S5); the transcripts were distributed into four categories. Unlike the targets of conserved miRNAs, most tags of novel miRNAs were grouped into category 2. In the CT library, 31 sequences with abundance ranging from eight to 9,509 tags per billion were detected. Of these 31 sequences, 14 were in category 2; 11 were in category 0; five were in category 4 and only one was in category 1. By contrast, 23 sequences with abundance ranging from 18 to 3,381 tags per billion were found in the NT library. There were five, two and 16 sequences in category 0, 1 and 2, respectively.

**Table 3:** **Functional description of target genes cleaved by novel miRNAs from the CT and NT libraries**

miRNA	Target gene NO.	Target gene description	Annotation	
			GO	KEGG
PC-46-5p	SGN-E548613	Probable protein phosphatase 2C 35-like	P2C65	-
PC-58-5p	SGN-E233471	Receptor-like protein kinase HAIKU2-like	IKU2	Protein-serine/threonine kinase
	SGN-E236245, SGN-E244660, SGN-E232041 and SGN-E378026	As above	As above	-
PC-75-3p	SGN-E263621, SGN-E355515, SGN-E356830, SGN-E356831 and SGN-E707134	Uncharacterized protein	AGO2	Argonaute
PC-89-5p	SGN-E746319	Bidirectional sugar transporter SWEET11-like	SWT11	-
PC-93-3p	SGN-E226448 and SGN-E703722	60S ribosomal protein L8-like	RL8	Large subunit ribosomal protein L8e
	SGN-E287356, SGN-E708964 and SGN-E713893	Bibosomal protein L2-like	RL8	As above
PC-102-5p	SGN-E233896, SGN-E350418, SGN-E351849, SGN-E352584, SGN-E353944, SGN-E701432, SGN-E705552, SGN-E713099 and SGN-E713896	Cellulose synthase A catalytic subunit 1 [UDP-forming]-like	CESA1	Cellulose synthase A
PC-117-5p	SGN-E720902	18.5 kDa class I heat shock protein-like isoform 1	HSP11	-
PC-125-3p	SGN-E294611 and SGN-E705851	Xyloglucan endotransglucosylase/hydrolase protein	XTH32	Xyloglucan:xyloglucosyl transferase
PC-146-5p	SGN-E208808, SGN-E254753, SGN-E262673, SGN-E269493 and SGN-E303554	Inositol-pentakisphosphate 2-kinase-like	IPPK	Inositol-pentakisphosphate 2-kinase

Unlike the conserved miRNAs, more target tags of the novel miRNAs were detected in the NT library, which suggested that under chilling stress, cleaving might not be the main mode of action between novel miRNAs and target genes. Perhaps eliciting mRNA degradation or arresting mRNA translation played a dominant role in this interaction. GO enrichment analysis showed that integral to membrane, cellulose biosynthetic process and cellular cell wall organization were the main functions of the target genes cleaved by the differentially expressed novel miRNAs (Additional file 8: Figure S5).

Compared with the targets of conserved miRNAs, target gene description showed that the target genes of novel miRNAs were with special functions (Table 3). Receptor-like protein kinase is regarded as the sensor or transducer of stress signals. A receptor-like protein kinase gene targeted by PC-58-5p was identified in the current study. The gene encoding xyloglucan endotransglucosylase/hydrolases (EC:2.4.1.207) targeted by PC-125-3p was detected, and the tag sequences were only detected in the NT library. In addition, PC-102-5p, targeting a cellulose synthase (EC:2.4.1.12) subunit gene, was detected in the two libraries. And the inositol-pentakisphosphate 2-kinase-like (EC:2.7.1.158) gene, which plays a role in the reaction between ATP and ADP and is targeted by PC-146-5p, was also identified.

### qRT-PCR verification

**Figure 4:**
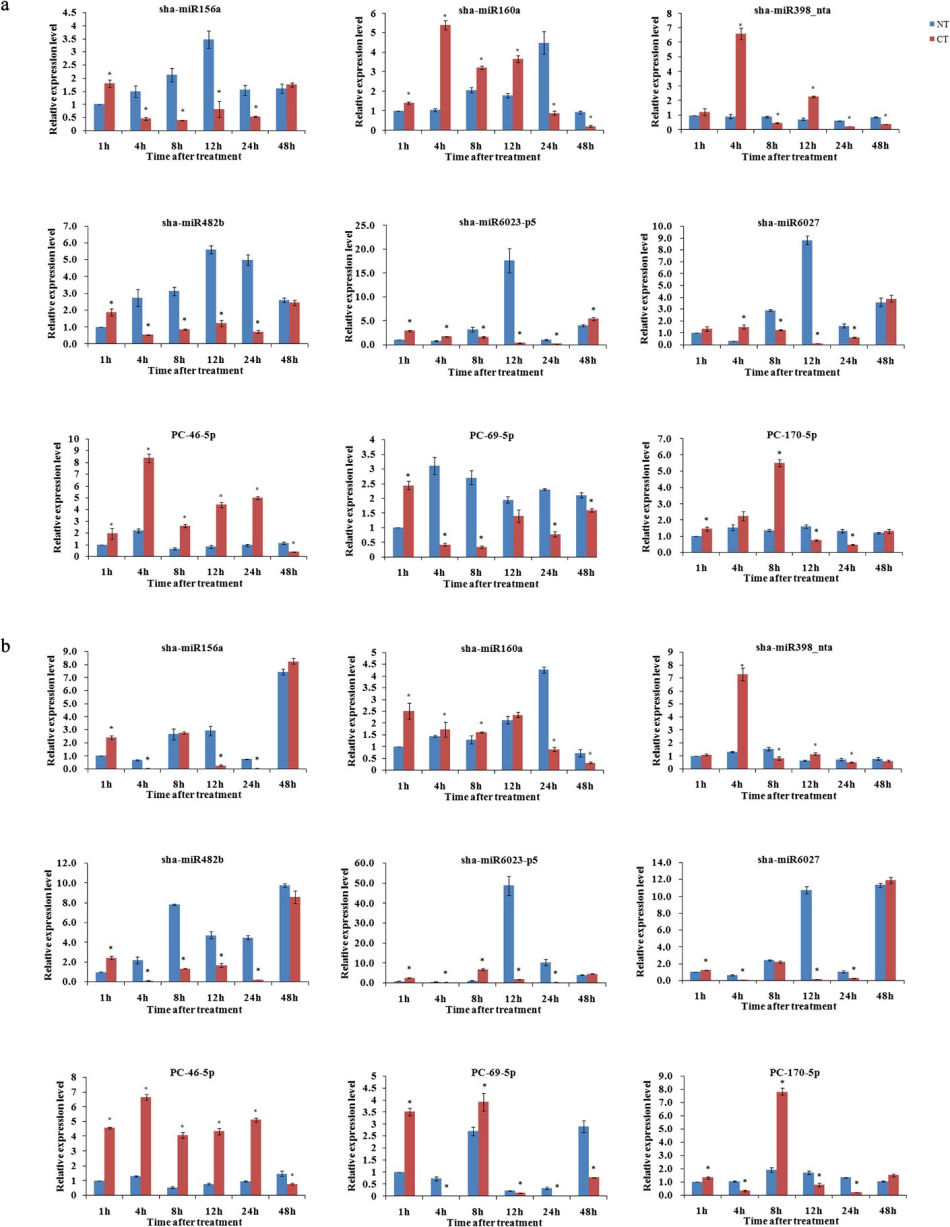
**qRT-PCR validation of chilling-responsive conserved and novel miRNAs in ‘LA1777’ (a) and ‘Hezuo908’ (b) tomato.** The reference gene was *5S rRNA*. All reactions were repeated three times. Normalized miRNA expression amount at 1 h without chilling treatment was arbitrarily set to 1. Differences between the NT and CT libraries were tested with a T test. *indicates a significant difference between the two libraries (<0.05).

**Figure 5:**
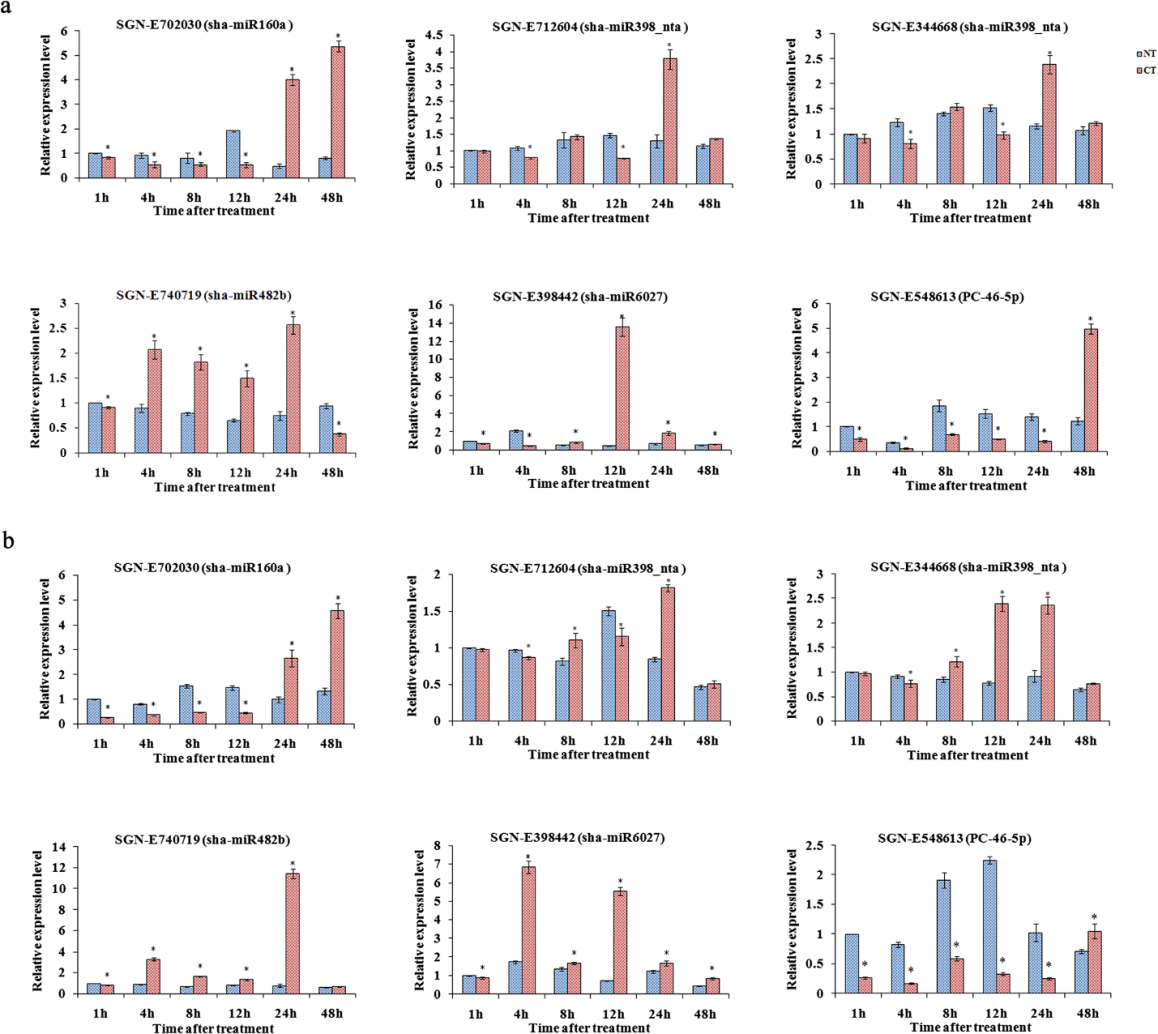
**qRT-PCR validation of target genes in ‘LA1777’ (a) and ‘Hezuo908’ (b) tomato.** The reference gene was *Actin*. All reactions were repeated three times. Normalized target gene expression amount at 1 h without chilling treatment was arbitrarily set to 1. Differences between the NT and CT libraries were tested with a T test. *indicates a significant difference between the two libraries (<0.05).

To examine the miRNAs expression amounts and verify the sequencing result, nine miRNAs including six conserved miRNAs and three novel miRNAs were selected for further analysis. Following previous studies [39, 47, 48], poly (A)-tailed qRT-PCR was utilized to analyze the temporal and spatial expression patterns of these miRNAs in tomato. For further verification and confirmation, another chilling-tolerant tomato cultivar, ‘Hezuo908’, was subjected to the same treatment as the tomato cultivar ‘LA1777’. Quantitative analysis showed that compared with the expression amounts of miRNAs in the NT libraries, the expression amounts of the nine miRNAs varied in the CT libraries of both cultivars (Figure 4). At 1 h of treatment, the expression amounts of sha-miR156a, sha-miR482b and PC-69-5p were significantly higher in both CT libraries than that in the NT libraries, followed by a marked decrease and then gradually increased. The expression amounts of sha-miR6023-p5 and sha-miR6027 were also significantly higher in both CT libraries at 1 h and then obviously decreased at 12 h. At 4 h, the expression amounts of sha-miR160a, sha-miR398_nta and PC-46-5p were significantly higher in both CT libraries than that in the NT libraries and then decreased, while the expression amounts of PC-170-5p were significantly higher in the CT libraries at 8 h.Meanwhile, the expression amounts of six target genes cleaved by sha-miR160a, sha-miR398_nta, sha-miR482b, sha-miR6027 and PC-46-5p were also verified by qRT-PCR. As shown in Figure 5, the expression amounts of target genes cleaved by sha-miR160a and PC-46-5p were both significantly lower in the CT libraries, followed by an increase, and they were significantly higher in the CT libraries at 48 h. The expression amounts of the target genes cleaved by sha-miR398_nta and sha-miR6027 exhibited the same trend, while they were significantly higher in the CT libraries and peaked at 24 h and 12 h, respectively. At 1 h, the expression amounts of target genes of sha-miR482b were significantly lower in CT libraries, followed by an increase and peaked at 24 h. The results were basically identical to the sequencing data.

### Network analysis

**Figure 6:**
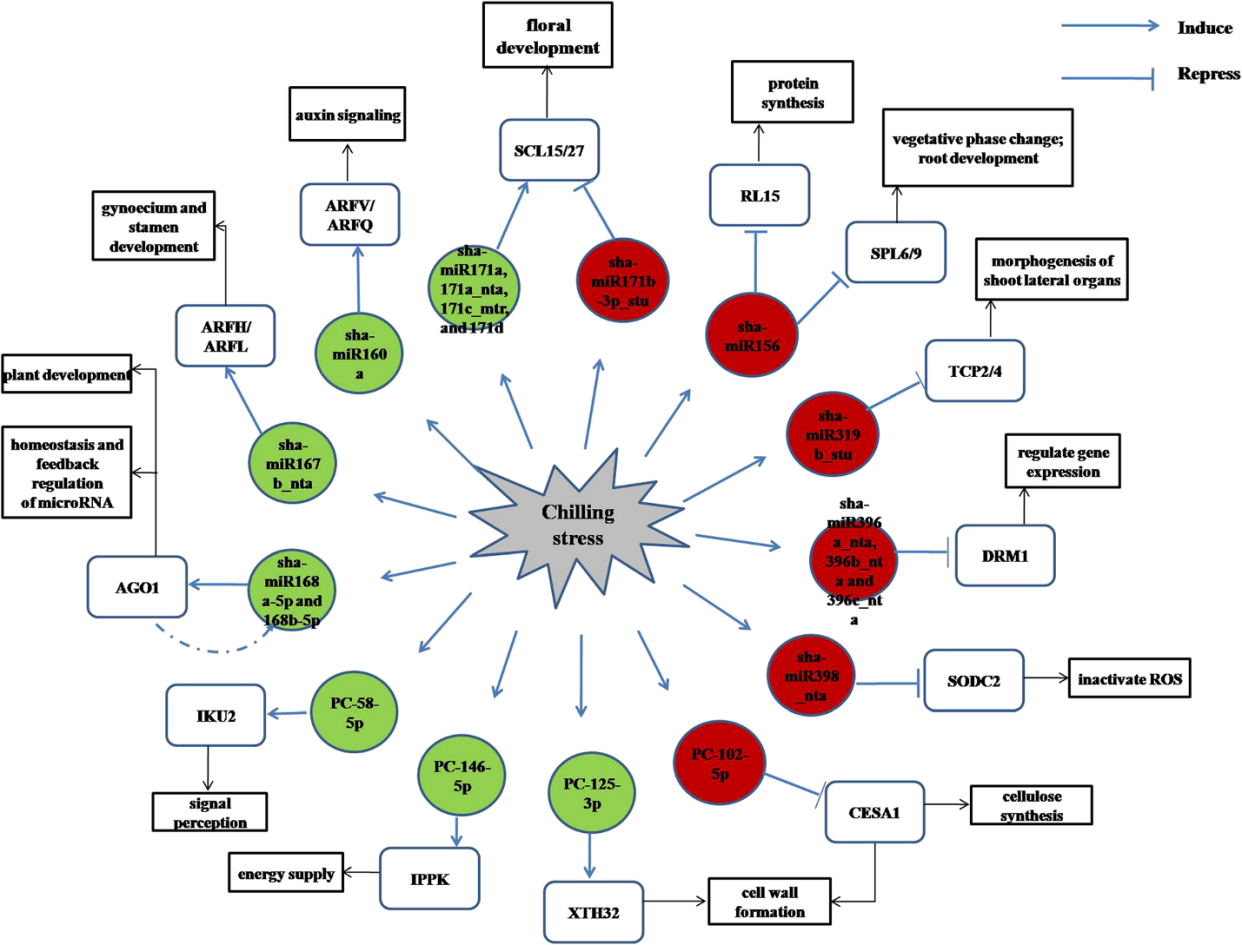
**MicroRNA-gene network analysis.** Target genes of microRNAs identified in the CT and NT libraries from tomato were assembled into the network according to GO annotation. The circles represent miRNAs (red indicates upregulated miRNAs and green indicates downregulated miRNAs), and rectangles represent the target genes.

A total of 71 genes targeted by 51 miRNAs were detected in response to chilling stress. The network between miRNAs and target genes was elucidated according to GO analysis (Figure 6). As shown in the network, the increased sha-miR156 (sha-miR156a, sha-miR156c, sha-miR156c_nta, sha-miR156d_nta, sha-miR156e_stu, sha-miR156g_stu, sha-miR156h_stu, sha-miR156i_stu, sha-miR156j_stu and sha-miR156i-p3_nta), sha-miR171b-3p_stu, sha-miR319b_stu, sha-miR396 (sha-miR396a_nta, sha-miR396b_nta and sha-miR396c_nta), sha-miR398_nta and PC-102-5p played important regulatory roles in the network by regulating the expression of genes encoding anti-stress proteins (*SPL*, *RL*, *SCL* and *TCP*), antioxidant enzyme (*SODC2*) and cellulose synthesis (*CESA1*), while the decreased sha-miR160a, sha-miR167b_nta, sha-miR168 (sha-miR168a-5p and sha-miR168b-5p), sha-miR171a, sha-miR171a_nta, sha-miR171c_mtr, sha-miR171d, PC-58-5p, PC-146-5p and PC-125-3p were involved in signal transduction and cell wall formation, as well as regulation of genes encoding anti-stress proteins (*SCL15/27*). Of the target genes examined, *SPL*, *SCL*, *TCP*, *SODC2*, *CESA1*, auxin response factors genes (*ARFV*, *ARFQ*, *ARFH* and *ARFL*), *IKU2*, *IPPK* and *XTH32* might be the most important genes in the entire network.

## Discussion

MiRNAs, a class of noncoding small RNAs, are important regulators involved in plant growth, development and stress responses that have received increasing amounts of attention [9, 32, 49–51]. In recent years, a new approach for discovering miRNAs, high-throughput sequencing technology, has been widely used to identify conserved and novel miRNAs in plants, which has enlarged the realm of miRNA research and made miRNA a hotspot of epigenetic research. Abiotic stress, a common stress that seriously affects plant production, has also received increasing attention. The miRNA regulation theory and the utilization of high-throughput sequencing have opened up a new area of abiotic stress research. In recent years, great progress has been made in understanding the role of miRNAs in the response to various environmental stresses, such as drought [52, 53], salt [54, 55] and waterlogging stresses [56]. Chilling is a common abiotic stress that seriously affects normal plant growth, development, yield and quality. Plant miRNAs involved in chilling response have been increasingly studied during the past few years. However, to our knowledge, few such studies have been reported in tomato.

Tomato is an important vegetable crop that is grown worldwide [57]. Chilling is a common abiotic stress that affects tomato cultivation, especially in the greenhouse during the cooler seasons [58]. In China, solar greenhouses are the principal systems employed for tomato cultivation in the spring and winter, when chilling stress is common [58]. Since miRNAs are thought to be important for the abiotic stress response and a high-through sequencing approach is available, research on chilling-responsive miRNAs in tomato will help to reveal the response mechanisms of the miRNAs and to further elucidate the mechanism of miRNA regulation in general. More importantly, the study provides a theoretical foundation for breeding chilling-tolerant tomato cultivars and for further research on miRNA regulation of the response to chilling stress.

### Conserved and novel miRNAs in tomato

To identify chilling-responsive miRNAs from tomato, two small RNA libraries from tomato seedlings treated with and without chilling stress were constructed. A high-through sequencing approach was utilized to identify conserved and novel miRNAs; 4,342,604 and 7,231,609 clean reads were obtained by high-throughput sequencing of the two libraries, respectively. Following filtering, a total of 161 conserved miRNAs belonging to 41 miRNA families were identified (Additional file 1: Table S1). Unexpectedly, 47 new flanking sequences noted as p3 or p5 were discovered (Additional file 1: Table S1). In miRBase 20.0, there were 46 precursors and 48 mature miRNA sequences from tomato. In this study, 161 conserved miRNA sequences generated from 92 precursors were identified. All conserved miRNAs were divided into 41 miRNA families, and several miRNA families were the first to be detected in tomato, such as miR164, 384, 390, 396, 398, 408, 530, 827, 1886, 3627, 7981, 7982, 7983 and 8007. Although miR395, 1918, 5302, 5303 and 5304 were deposited in miRBase, they were not detected in the current study, perhaps because these miRNAs have specific expression patterns. Additionally, compared with the miRNAs deposited in miRBase, many of the conserved miRNAs identified in the current study were also detected in other members of the Solanaceae family, such as potato and tobacco, which suggested that these miRNAs were conserved in Solanaceae. Therefore, there was more vast development space for tomato miRNAs.

Additionally, 236 novel miRNAs generated from 171 precursors were identified (Additional file 3: Table S2). In contrast to conserved miRNAs, most novel miRNAs exhibited low expression amounts with norm read <100, which was previously reported in *Arabidopsis* [41, 44, 59], wheat [45] and grapevine [60]. However, several novel miRNAs exhibited moderate or high expression amounts with norm read > 1,000. In the current study, a total of 161 conserved and 236 novel miRNAs were firstly discovered to be associated with the tomato chilling response.

### Differentially expressed miRNAs in tomato

The abundance of miRNAs can be regarded as an index for estimating the expression amounts of miRNAs. Therefore, to identify chilling-responsive miRNAs, the expression amounts of miRNAs between the two libraries were compared. In total, 192 increased and 205 decreased miRNAs were differentially expressed between the two libraries. And the miRNAs were expected to play key roles in the chilling response. It is worth to noting that 49 miRNAs were significantly regulated by chilling stress (Additional file 1: Table S1, Additional file 3: Table S2). Of these, 23 miRNAs including 12 conserved and 11 novel miRNAs significantly increased in the response to chilling stress, while 26 miRNAs including 20 conserved and six novel miRNAs significantly decreased. In short, more miRNAs were cold-suppressed miRNAs, which was in agreement with previous research on *Brachypodium* [24].

Of the miRNAs with significantly altered expression, several chilling-responsive miRNAs were conserved among several plant species. MiR397 was induced by chilling stress in *Brachypodium*, *Populus tomentosa* and *Arabidopsis* [22, 24, 26]. MiR319 was induced by chilling stress in *Arabidopsis* [22]. In the current study, sha-miR397(sha-miR397_nta and sha-miR397-p5) and sha-miR319 (sha-miR319 and sha-miR319b_stu) were both found to be induced by chilling stress, which indicated that some miRNAs involved in chilling stress showed consistency among several plant species. Additionally, some miRNAs, such as miR169, 172 and 393, increased under the chilling stress in *Arabidopsis* [22] and *Brachypodium* [24], but in this study, no obvious change in expression was detected. On the other hand, several previously reported chilling-responsive miRNAs, such as miR402 in *Arabidopsis* [22], were not detected in the current study, which suggested that these miRNAs might be species-specific and their expression was not specifically altered in tomato, or perhaps the altered expression of these miRNAs did not occur during the duration of the study.To confirm and verify the sequencing result, nine miRNAs, including conserved sha-miR156a, sha-miR160a, sha-miR398_nta, sha-miR482b, sha-miR6023-p5 and sha-miR6027 and the novel PC-46-5p, PC-69-5p and PC-170-5p, were chosen for qRT-PCR verification in the sequenced tomato cultivar ‘LA1777’. As shown in Figure 4, the results of verification were consistent with the sequencing results. For further confirmation, the expression of these miRNAs was also examined in another more chilling-tolerant tomato cultivar, ‘Hezuo908’. The results showed a similar trend in the two tomato cultivars, while the range of altered expression differed among cultivars. The result suggested that there was a consistent relationship between the miRNA sequences of the two tomato cultivars. However, the extent of the responses differed between the cultivars under chilling stress, perhaps due to differences in the levels of chilling tolerance.

### Target genes of tomato miRNAs

To study the function of miRNAs in the chilling stress response, the two libraries were also used to degradome sequencing. As expected, many predicted targets for conserved miRNAs were identified, including those that function in various biological processes (Additional file 5: Table S3). For example, *SPL*, *AP2* and *MADS-box*, which were targeted by sha-miR156 (sha-miR156a, sha-miR156c, sha-miR156c_nta, sha-miR156d_nta, sha-miR156e_stu, sha-miR156g_stu, sha-miR156h_stu, sha-miR156i_stu and sha-miR156j_stu), sha-miR172 (sha-miR172a, sha-miR172b, sha-miR172i_nta and sha-miR172c-3p_aly) and sha-miR396a_nta, respectively, participated in plant development. In addition, genes involved in regulating the plant response to chilling stress were also identified. For example, most genes encoding anti-stress proteins (scarecrow-like protein and ribosomal protein) targeted by sha-miR171a_nta, sha-miR171b-3p_stu, sha-miR171c_mtr and sha-miR156i-p3_nta were only detected in the CT library, which was in agreement with previous results from *Arabidopsis* [22]. Moreover, the Cu/Zn superoxide dismutase-encoding gene cleaved by increased sha-miR398_nta was also mainly detected in the CT library, which suggested that sha-miR398_nta and the gene played an important role in the chilling response, which was also in agreement with previous studies of *Arabidopsis* [23] *.* In the current study, auxin response factors genes were cleaved by decreased sha-miR160a and sha-miR167b_nta and more tag sequences were mainly detected in the NT library, while only miR167-targeted auxin response factors genes were identified in the chilling response in rice [27]. The results suggested that among different plant species, conserved miRNAs might share similar functions by targeting homologous genes in the chilling stress response. Additionally, most of the conserved miRNA targets were classified as category 0 (Additional file 5: Table S3, Additional file 6: Figure S3, Additional file 7: Figure S4), containing a cleavage site in only one position, further confirming the accuracy of the degradome sequencing result.

Targets of novel miRNAs were also examined (Table 3, Additional file 10: Table S5). Compared with conserved miRNAs, only nine target genes of nine novel miRNAs were identified. GO annotation showed that some specific targets cleaved only by novel miRNAs were identified. Receptor-like protein kinases play important roles in defense responses. When plants suffer from abiotic stress, receptor-like kinases may be the first sensor or transducer of stress signals. Moreover, several related genes encoding receptor-like protein induced by chilling stress have been reported in rice [61], maize [28] and *Arabidopsis thaliana* [62]. In the current study, a receptor-like protein kinase gene targeted by PC-58-5p was also identified, which suggested that PC-58-5p was an important regulator of the chilling response. A cellulose synthase subunit gene targeted by PC-102-5p was detected in the research; this gene played an important role in cellulose synthesis [63] and cell wall formation [64]. In addition, cellulose synthase was involved in the low temperature stress response by affecting fiber quality, cell wall formation and cell wall loosening [65]. Perhaps the cellulose synthase subunit gene played an essential role in the low temperature response. In addition, PC-125-3p targeted a gene encoding xyloglucan endotransglucosylase/hydrolases, which regulated another major component of the plant cell wall. Several related genes in *Arabidopsis* and rice played a role in the cold stress response [66, 67]. Interestingly, PC-146-5p targeted the inositol-pentakisphosphate 2-kinase-like gene, encoding an enzyme that catalyzed the chemical reaction between the ATP and ADP [68, 69]. Perhaps this enzyme participated in the stress response by controlling the energy supply.

In the CT library, 301 target tags were cleaved by 48 miRNAs with the abundance of the target tags ranging from eight to 9,509 tags per billion, while 216 target tags were cleaved by 48 miRNAs in the NT library with an abundance ranging from nine to 7,470 tags per billion. Of these, 171 and 16 target tags which were cleaved by 33 conserved and four novel miRNAs respectively increased in the stress, while 108 and 18 target tags which were cleaved by 37 conserved and five novel miRNAs respectively decreased. Combined with the miRNA expression analysis, the result showed that the abundance of the target tags was basically consistent with the miRNA expression amount. On the other hand, the expression amounts of six target genes cleaved by sha-miR160a, sha-miR398_nta, sha-miR482b, sha-miR6027 and PC-46-5p were also verified by qRT-PCR. Compared with the expression amounts of these miRNAs, it was found that the decline in expression of target genes was basically accompanied by an increase in miRNAs expression amounts, which suggested that the tomato miRNAs positively regulated their target genes, which were involved in the chilling response.

Surprisingly, several low temperature-induced transcription factor genes, such as genes encoding DREB/CBF, WRKY and MYB were not detected in the current study, perhaps because these genes were not expressed at the time of sampling or in the leaves.

### Network analysis

By elucidating the network between miRNAs and their target genes (Figure 6), the increased sha-miR156 (sha-miR156a, sha-miR156c, sha-miR156c_nta, sha-miR156d_nta, sha-miR156e_stu, sha-miR156g_stu, sha-miR156h_stu, sha-miR156i_stu, sha-miR156j_stu and sha-miR156i-p3_nta), sha-miR171b-3p_stu, sha-miR319b_stu, sha-miR396 (sha-miR396a_nta, sha-miR396b_nta and sha-miR396c_nta) and sha-miR398_nta targeted genes with various functions, which suggested that these miRNAs played important regulatory roles in the tomato chilling response network. The corresponding target genes, including *SPL*, *SCL*, *TCP*, *DRM1* and *SODC2*, played important roles in regulating plant development, gene expression and ROS inactivation. The decreased sha-miR160a, sha-miR167b_nta, sha-miR168 (sha-miR168a-5p and sha-miR168b-5p), sha-miR171a, sha-miR171a_nta, sha-miR171c_mtr and sha-miR171d targeted genes that function in signal transduction, regulation of the expression of miRNAs and genes encoding anti-stress proteins. Of the novel miRNAs, PC-102-5p increased in the stress, which played an important role in cellulose synthesis. While PC-58-5p, PC-125-3p and PC-146-5p all decreased in the stress, and their target genes were involved in signal perception, cell wall formation and energy supply. Therefore, these miRNAs might be involved in the chilling response by inhibiting plant development, cell wall formation and genes expression, as well as improving signal perception, energy supply and regulating miRNA expression.

## Conclusions

In summary, in the present study, using high-throughput sequencing, hundreds of chilling-responsive miRNAs were identified in tomato. The information fills the gaps in the knowledge of tomato chilling-responsive miRNAs and increases the knowledge of the response of miRNAs to stress. Additionally, many target genes of miRNAs were identified by degradome sequencing. The result showed that the target genes were involved in various functions. Notably, most target genes of differentially expressed miRNAs actively respond to chilling stress by regulating the expression of anti-stress proteins, antioxidant enzyme and genes involved in cell wall formation. The findings lay the foundation for exploring the role of the regulation of miRNAs in the plant response to chilling stress.

## Methods

### Plant materials and treatments

The wild tomato cultivar ‘LA1777’ (*S. habrochaites*) with higher chilling-tolerance ability, was obtained from the Tomato Genetic Resource Center, University of California. After soaking and pregermination, the tomato seeds were sown in an aperture disk and cultured in the greenhouse (average day/night temperatures, 25 ± 1°C/20 ± 1°C). Seedlings at the three-leaf stage were transferred into nutrition pots. At the five-leaf stage, healthy tomato seedlings were pretreated for two days under a 14-h light (25°C)/10-h dark (20°C) photoperiod (photo intensity 180 μmol m^−2^ s^−1^). The seedlings were then divided into two groups for treatment; one group was exposed to 4°C/4°C for chilling treatment (CT), while the other group was not treated with chilling stress (NT). Both groups were grown under a 14 h light/10 h dark photoperiod at a light intensity of 180 μmol m^−2^ s^−1^. The third leaf from the stem apex was harvested at 1, 4, 8, 12, 24 and 48 h after treatment. To reduce the differences between individuals, samples were collected from six tomato seedlings at a single time point. And there were three biological replicates per treatment. Sample from the two groups were immediately frozen in liquid nitrogen and stored at -80°C until use.

### Small RNA library construction and deep sequencing

Tomato leaves were harvested at 1, 4, 8, 12, 24 and 48 h after chilling treatment and leaves from seedlings not exposed to chilling treatment were harvested at the same time points. To construct two small RNA libraries, samples from the two treatments harvested at different time points were individually subjected to small RNA extraction using an Illumina Truseq Small RNA Preparation kit (Illumina, San Diego, USA). Equal quantities of small RNA extracted from different time points in one treatment group were pooled as one small RNA library, and the two small RNA libraries were then constructed. Following isolation and ligation, the small RNAs were reverse transcribed to cDNAs. The purified cDNAs from the two RNA libraries were sequenced with an Illumina Genome Analyzer II (LC Sciences, Hangzhou, China) according to the manufacturer’s protocols.

### Identification of conserved and novel miRNAs

After sequencing, raw reads were obtained using Illumina sequencing-related software analysis. Next, the ACGT101-miR program (version 4.2; LC Sciences, Hangzhou, China), a proprietary pipeline script, was utilized for further analysis [70]. Raw reads with low quality and low copy numbers were removed. Adapter sequences were removed, and the remaining sequences with lengths between 19 and 24 nt were subjected to analysis with miRBase 20.0 (http://www.mirbase.org/) for conserved and novel miRNAs identification. A maximum of one mismatch in the first 16 nt of the miRNA and three mismatches in total were allowed between the target miRNAs and known miRNAs deposited in the miRBase database [35]. The specific criteria of miRNAs prediction were described by Meyers et al. [71]. Stable hairpin structures were also an important identification index [11, 71]. Therefore, hairpin RNA structures were predicted using UNAfold software (http://mfold.rna.albany.edu/?q=mfold/RNA-Folding-Form) [17, 42]. In addition, MFEI was another important feature to be taken into consideration [43]. Only miRNAs with perfect hairpin structures that met the criteria for miRNAs were regarded as novel miRNAs candidates. All of the sequences were aligned to the tomato genome database (http://solgenomics.net) by BLAST. Sequences that matched mRNAs, repeat sequences, tRNAs, sRNAs, snRNAs, snoRNAs, other noncoding RNAs in Rfam (http://www.sanger.ac.uk/software/Rfam) [72] and the noncoding database of GenBank (http://www.ncbi.nlm.nih.gov/) were discarded. Finally, the remaining sequences were grouped into families.

### Degradome library construction and data analysis

Two degradome libraries, i.e., the CT library and the NT library, were constructed from tomato leaves following the method reported by Ma et al. [73]. Poly A-enriched RNA was obtained and ligated to oligonucleotide adaptors harboring an MmeI recognition site. Following reverse transcription, first-strand cDNA was generated from the ligated sequence. After a short PCR amplification, additional DNA products were produced. Following purification, digestion, ligation and purification, the cDNA library was subjected to cluster analysis in an Illumina Cluster Station and sequenced with an Illumina Genome Analyzer II (LC Sciences, Hangzhou, China).

After sequencing, a public software package, CleaveLand3.0, was used to analyze the generated sequencing data [46, 74]. MRNA data from the Tomato Genomics Database (http://solgenomics.net) was used as reference data to map the degradome sequencing products.

Alignment between the miRNA and its target was scored by base pairing. Mismatched pairs or single nucleotide bulges were scored as 1, and G:U pairs were scored as 0.5. The score was doubled if the mismatches were located in the core position (2–13) of the miRNA sequence. Based on the abundance of the degradome sequences and cleavage sequences, the targets were grouped into five categories. Category 0 targets were targets in which only one degradome tag was detected, and it was detected at the expected site, which was the most abundant category (>1). Category 1 comprised degradome sequences (>1) whose scores were equal to the maximum, and there were more maximum positions. Category 2 included the sequences (>1) whose abundance fell between the maximum and median values. Category 3 consisted of the sequences (>1) with scores below the median value. The remaining sequences, which included only one raw read, were defined as category 4.

### Analysis of miRNAs and target genes by qRT-PCR

Small RNA was extracted from tomato seedlings exposed to chilling treatment or no chilling treatment for 1, 4, 8, 12, 24 and 48 h using RNAiso (Takara, Dalian, China). According to the poly (A) polymerase procedure (Ambion, Austin, TX), small RNA was ligated to poly (A) tails. Using T4 RNA ligase (Invitrogen, Carlsbad, CA), 5’ adaptor (5’-CGACUGGAGCACGAGGACACUGACAUGGACUGAAGGAGUAGAAA-3’) was added to the poly (A)-tailed RNA [47]. Small RNA and RT primer (ATTCTAGAGGCCGAGGCGGCCGACATG-d [T] _30_ [A, G or C] [A, G, C or T]) were utilized to amplify the cDNA by reverse transcription [39, 48]. The products were used as templates to analyze the expression of the miRNAs. Meanwhile, total RNA was extracted and utilized to detect the expression amounts of the target genes. qRT-PCR was carried out to detect the expression of the identified miRNAs and target genes. The primers were listed in Additional file 11: Table S6 and Additional file 12: Table S7. The amplification was carried out with SYBR Premix Ex Taq™ (Takara, Dalian, China) in an Eppendorf real-time PCR machine (Mastercycler®ep realplex, Hamburg, Germany) following the manufacturer’s instructions. All reactions were repeated three times, and *5S rRNA* and *Actin* were utilized as the reference genes. The expression amounts of the miRNAs and target genes were calculated using the 2^-ΔΔCt^ method.

For further validation, another chilling tolerant tomato cultivar, ‘Hezuo908’, was subjected to the same treatment as the tomato cultivar ‘LA1777’, and qRT-PCR was performed to examine the expression amounts of miRNAs and target genes using the process described above.

### GO, KEGG and network analysis

GO analysis was utilized to study the functions of the target genes of the miRNAs based on the database (http://www.geneontology.org/). KEGG pathway analysis of the miRNAs targets was performed simultaneously based on the KEGG database (http://www.genome.jp/kegg/). Network between miRNAs and their target genes was subsequently assembled according to the GO analysis results.

### Statistical analysis

Statistical analysis was performed to compare the miRNAs expression and target genes degradation between the CT and NT libraries. The differences between the two libraries were tested using the chi-square test and the fisher-exact test, and the log_2_ ratio was regarded as a threshold to detect miRNAs expression and target genes degradation fold changes.

### Data access

The sRNA sequence data from this study have been submitted to Gene Expression Omnibus (GEO) under accession number GSE57335 at http://www.ncbi.nlm.nih.gov/geo/query/acc.cgi?acc=GSE57335.

## Electronic supplementary material

Additional file 1: Table S1: - Conserved miRNAs from tomato in the CT and NT libraries. ^a^sequence in miRbase, “Yes” indicates that the sequence can be found in miRBase; “Different” indicates that the sequence is different from the miRNA sequence deposited in miRBase; “New” indicates that the sequence is newly discovered compared with the miRNA sequences deposited in miRBase; ^b^Pre-miRNA sequence in miRBase, “Yes” indicates that the pre-miRNA sequence can be found in miRBase; “Predicted” indicates that the pre-miRNA sequence was not found in miRBase and was predicted by UNAFold software; ^c^*indicates a significant difference between the CT and NT libraries. Chi-square test and fisher-exact test results were both <0.05 and | log_2_ (CT/NT) | ≥1. (XLSX 34 KB)

Additional file 2: Figure S1: - Secondary structures of 161 conserved miRNAs identified in the CT and NT libraries. The mature miRNAs sequences are underlined in yellow. (DOCX 13 MB)

Additional file 3: Table S2: - Novel miRNAs identified in the CT and NT libraries in tomato. ^a^“PC” indicates predicted candidate; ^b^dashed boxes indicate two identical sequences; ^c^*indicates significant difference between the CT and NT libraries. Chi-square test and fisher-exact test values were both <0.05 and | log_2_ (CT/NT) | ≥ 1. (XLSX 38 KB)

Additional file 4: Figure S2: - Secondary structures of 236 putative novel miRNAs identified in the CT and NT libraries. The mature miRNAs sequences are underlined in yellow. (DOCX 19 MB)

Additional file 5: Table S3: - Targets of conserved miRNAs from tomato in the CT and NT libraries. *indicates significant difference between the CT and NT libraries. Chi-square test and fisher-exact test results were both <0.05 and | log_2_ (CT/NT) | ≥ 1. (XLSX 37 KB)

Additional file 6: Figure S3: - T-plots for targets of tomato miRNAs identified in the NT library. According to German et al, t-plots are referred to as “target plots” and the normalized numbers are used to plot the cleavages on target mRNAs. The frequency of degradome tags with 5’ends at the indicated positions is marked in black, and the frequency cleaved at position 10 of the inset miRNA target alignment is highlighted in red. The abscissa number t indicates the cleavage site detected in the target mRNA. (DOCX 14 MB)

Additional file 7: Figure S4: - T-plots for targets of tomato miRNAs identified in the CT library. According to German et al, t-plots are referred to as “target plots” and the normalized numbers are used to plot the cleavages on target mRNAs. The frequency of degradome tags with 5’ends at the indicated positions is marked in black, and the frequency cleaved at position 10 of the inset miRNA target alignment is highlighted in red. The abscissa number t indicates the cleavage site detected in the target mRNA. (DOCX 13 MB)

Additional file 8: Figure S5: - GO enrichment analysis of the functions of target genes cleaved by conserved and novel miRNAs. (JPEG 728 KB)

Additional file 9: Table S4: - KEGG pathway analysis of target genes of conserved and novel miRNAs in the CT and NT libraries. (XLSX 9 KB)

Additional file 10: Table S5: - Targets of novel miRNAs in the CT and NT libraries. *indicates significant difference between the CT and NT libraries. Chi-square test and fisher-exact test results were both <0.05 and | log_2_ (CT/NT) | ≥ 1. (XLSX 13 KB)

Additional file 11: Table S6: - MiRNAs and the primers used for qRT-PCR verification. (XLSX 8 KB)

Additional file 12: Table S7: - Target genes and the primers used for qRT-PCR verification. (XLSX 8 KB)

## List of abbreviations

MiRNAs: microRNAs
sRNAs: small RNAs
nt: nucleotides
CT: chilling-treated
NT: non-chilling-treated
qRT-PCR: quantitative real-time PCR
DCL: dicer-like
AGO: argonaute
RISC: RNA-induced silencing complex
MFEI: minimal folding free index
t-plots: target plots
GO: Gene ontology
KEGG: Kyoto encyclopedia of genes and genomes.
