# Supplementary material for: Identification of chilling stress-responsive tomato microRNAs and their target genes by high-throughput sequencing and degradome analysis
Source: BMC Genomics. 2014 Dec 17;15(1):1130. doi: 10.1186/1471-2164-15-1130 (PMC4377850; doi:10.1186/1471-2164-15-1130)
Supplement: Supplementary file 2 — Additional file 2: Figure S1: - Secondary structures of 161 conserved miRNAs identified in the CT and NT libraries. The mature miRNAs sequences are underlined in yellow. (DOCX 13 MB) [file 12864_2014_6877_MOESM2_ESM.docx]

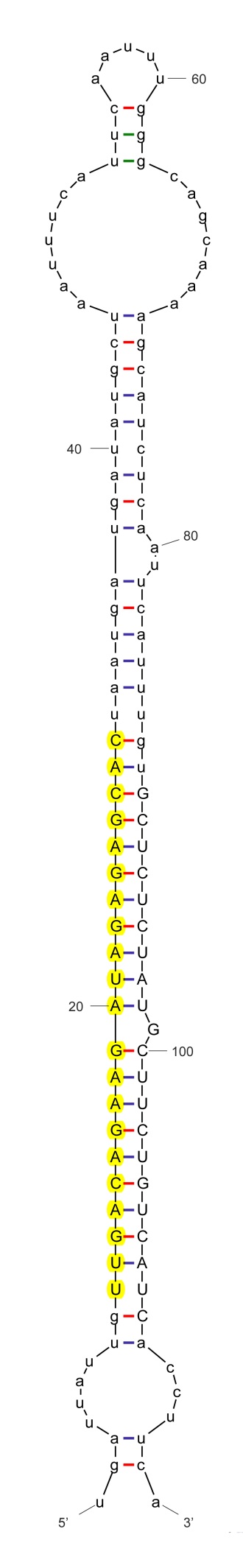


sha-miR156a


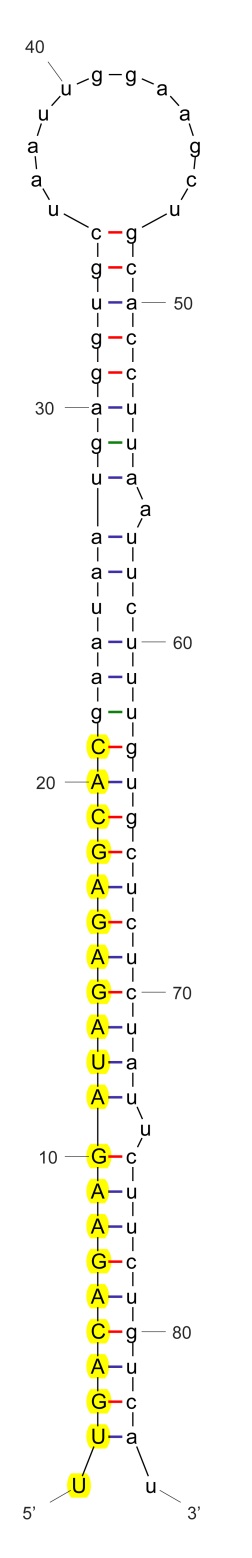


sha-miR156c


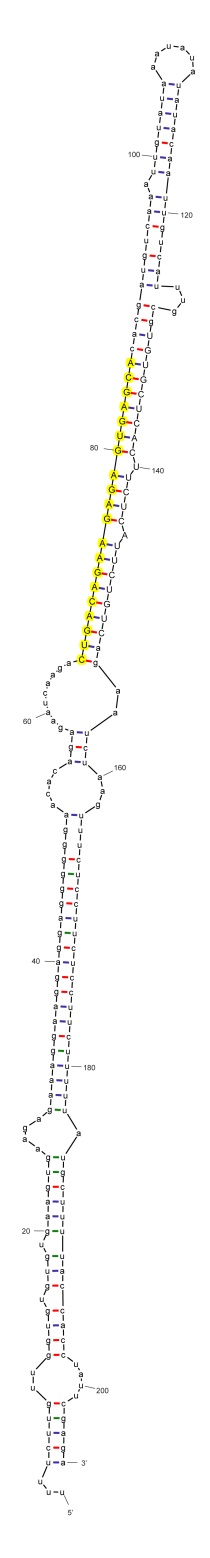


sha-miR156c_nta


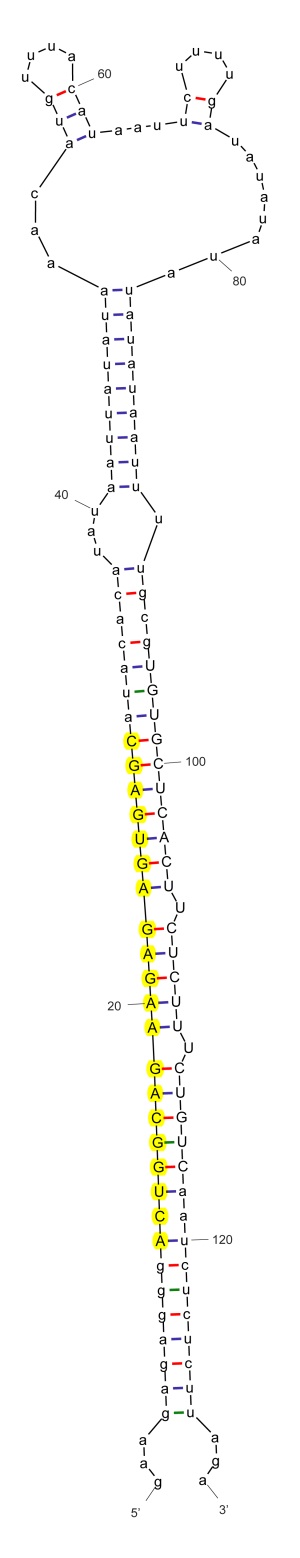


sha-miR156c-p5_nta


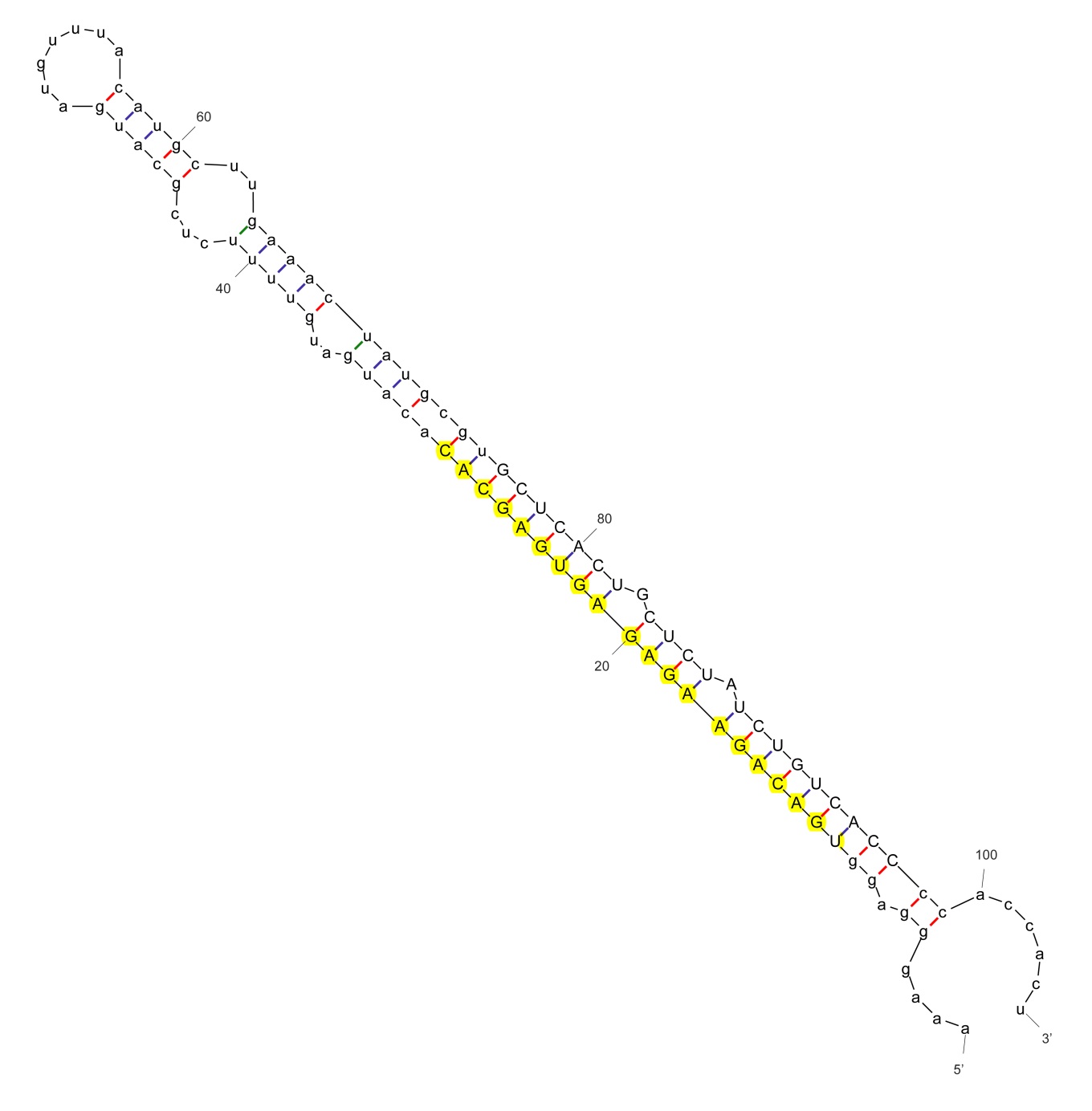


sha-miR156d_nta


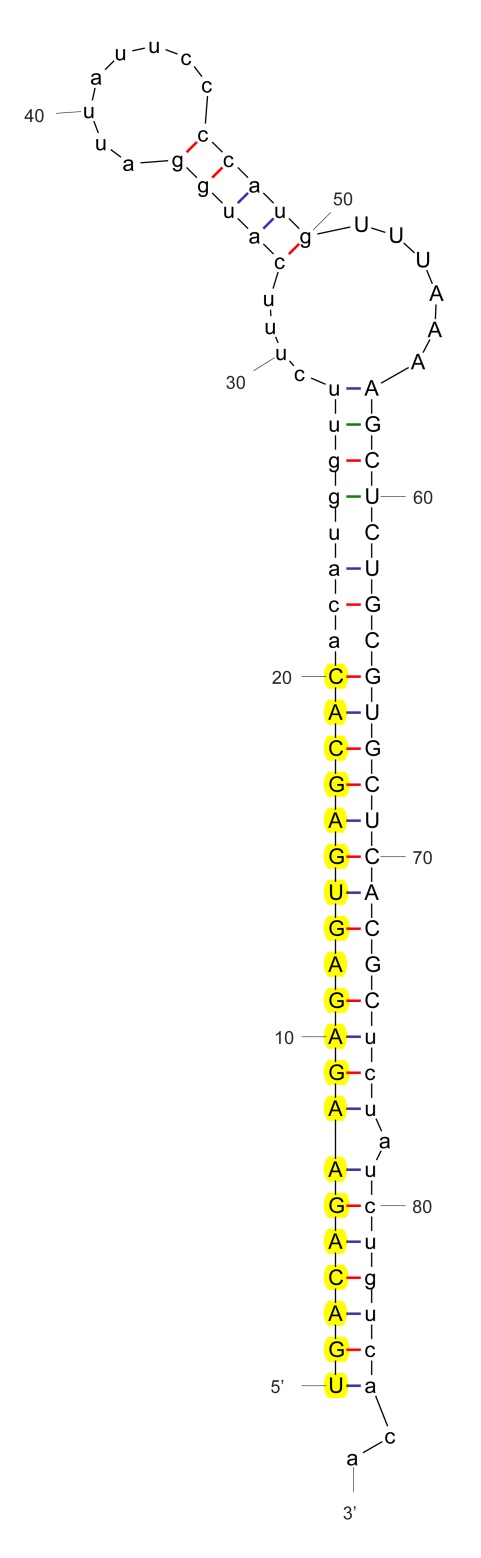


sha-miR156e_stu


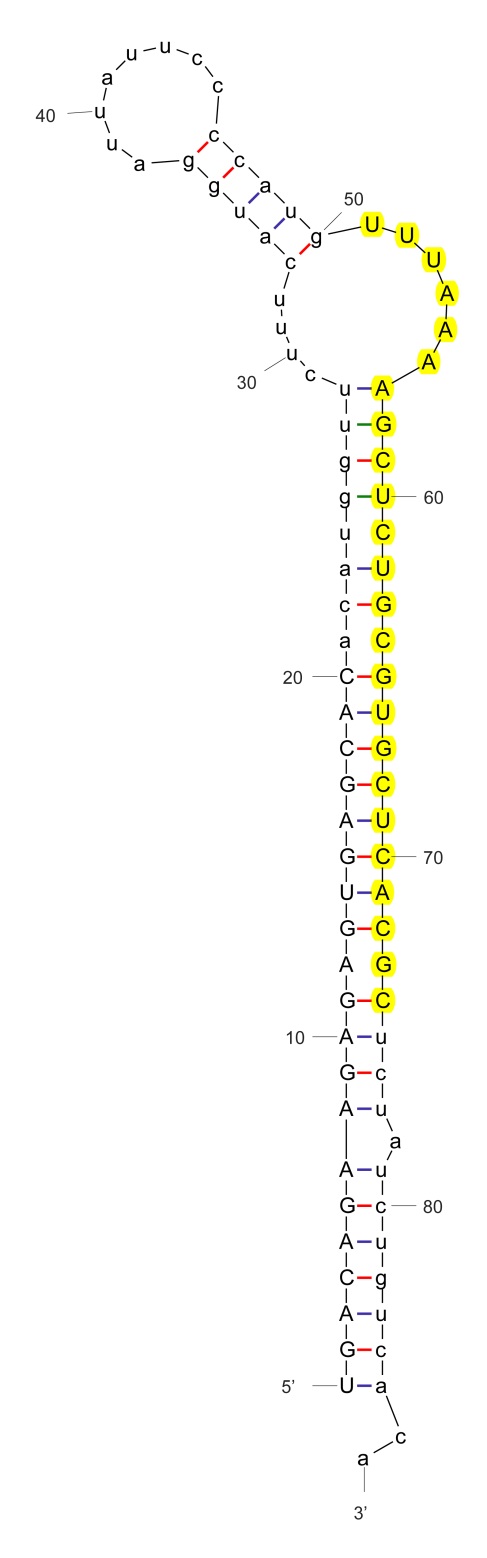


sha-miR156e-p3_stu


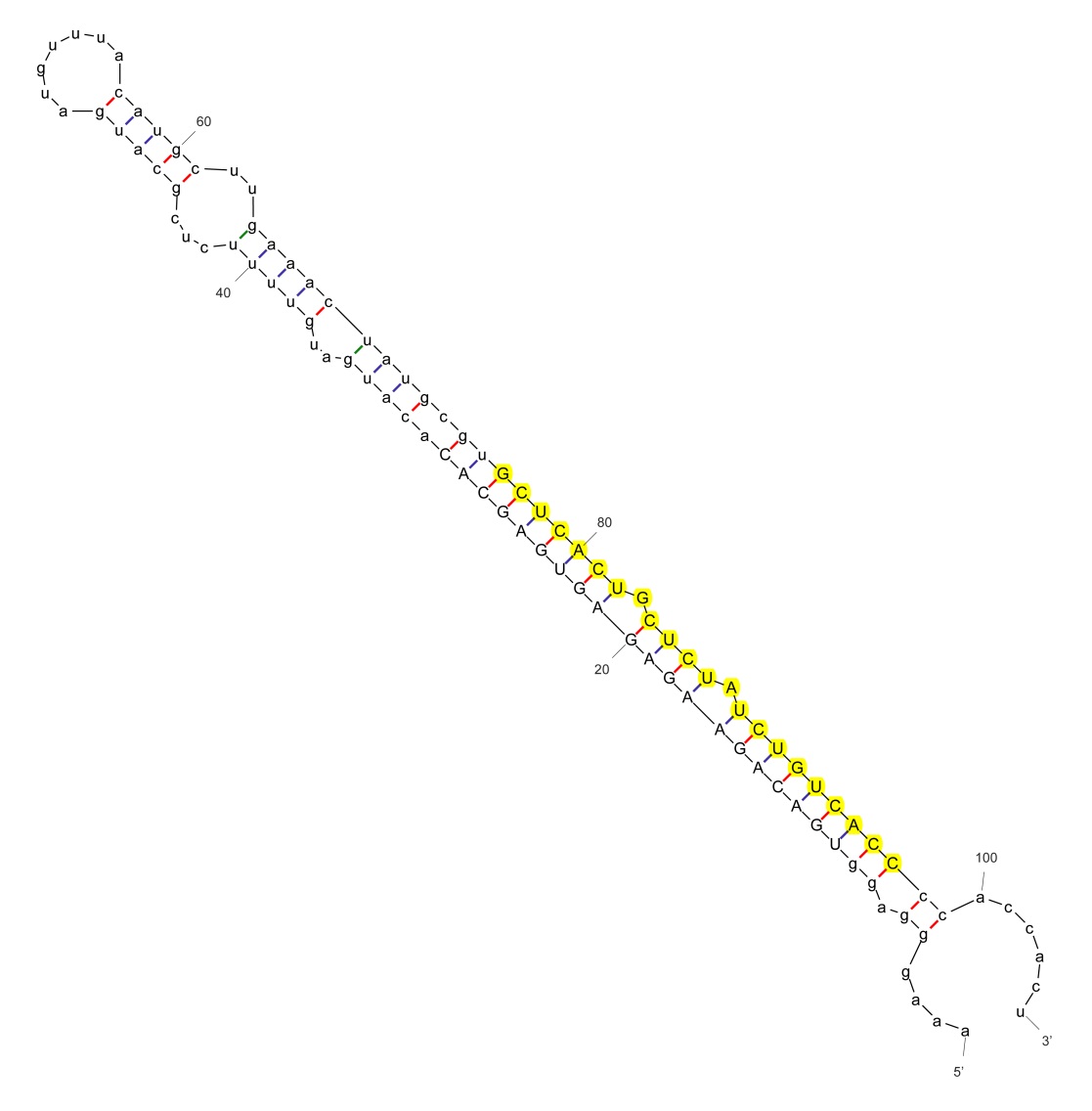


sha-miR156e-p3_nta


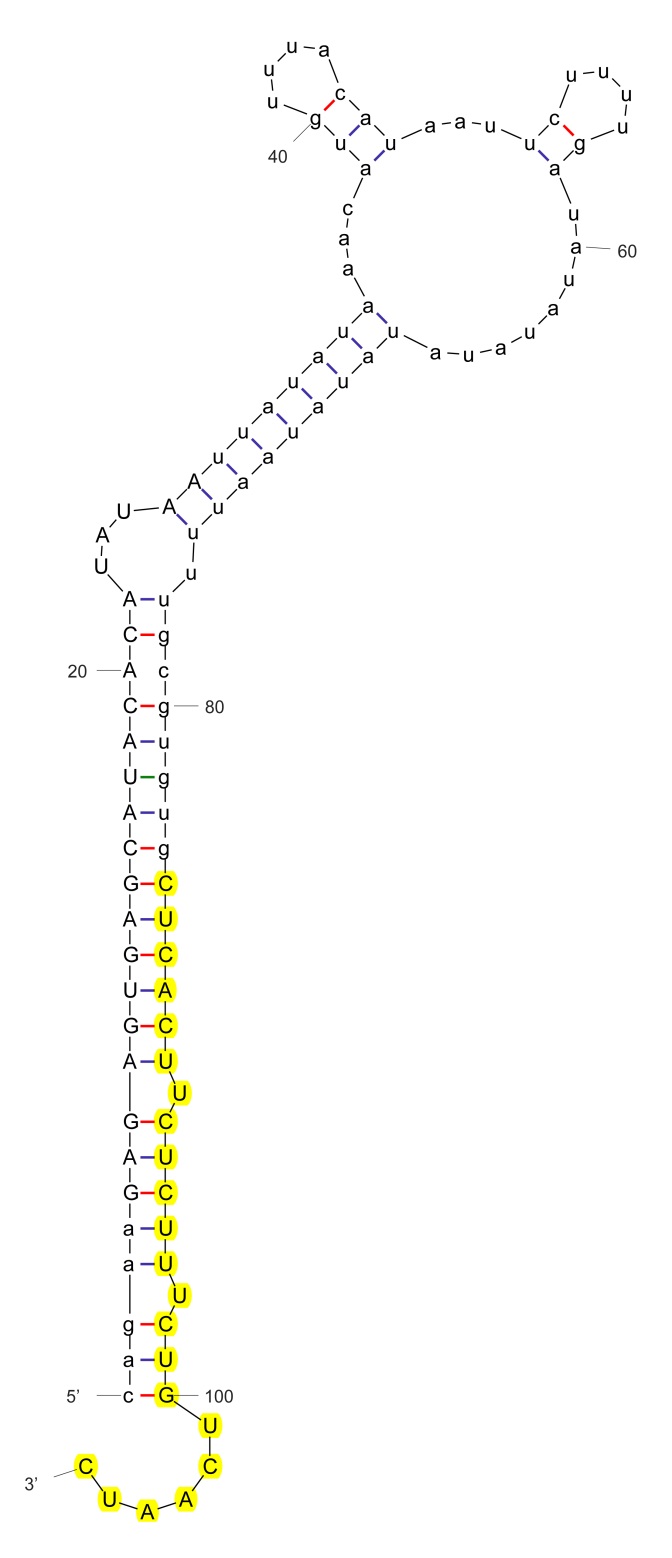


sha-miR156f-3p_stu


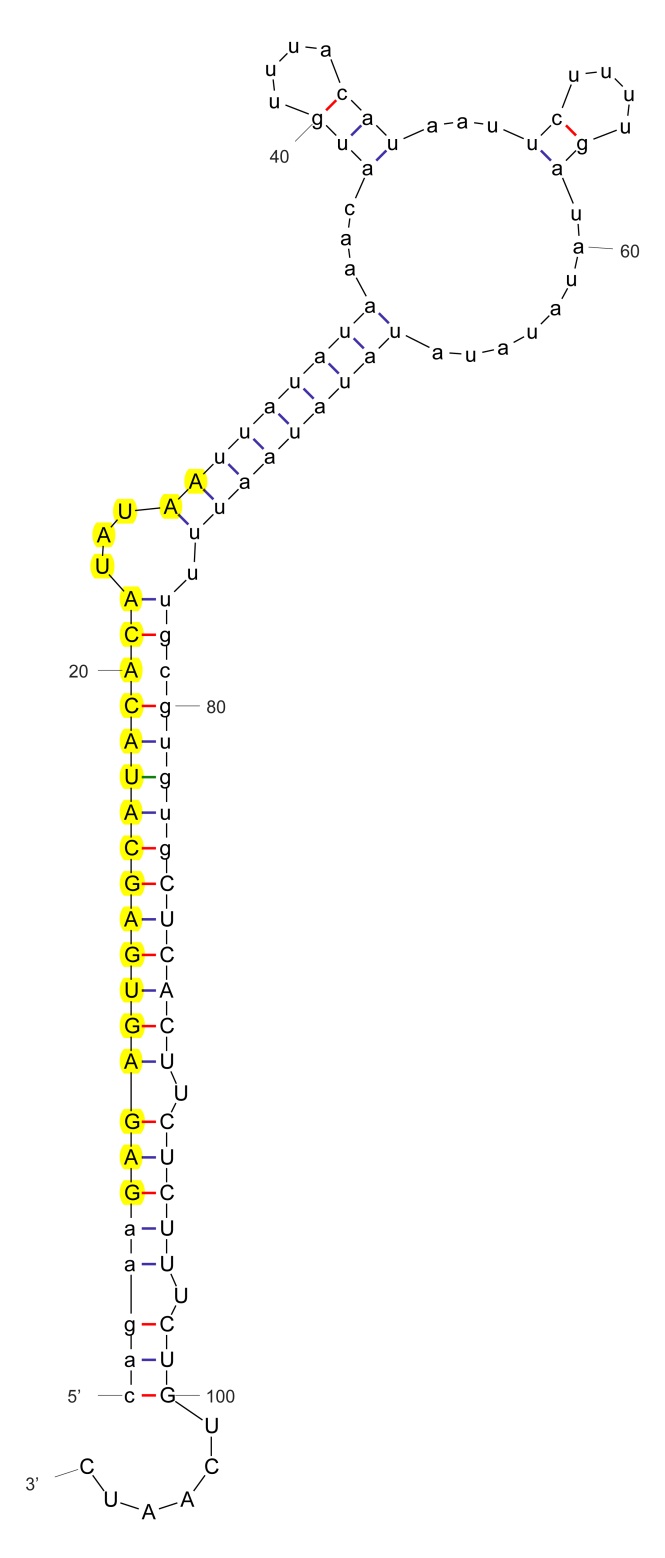


sha-miR156f-p5_stu


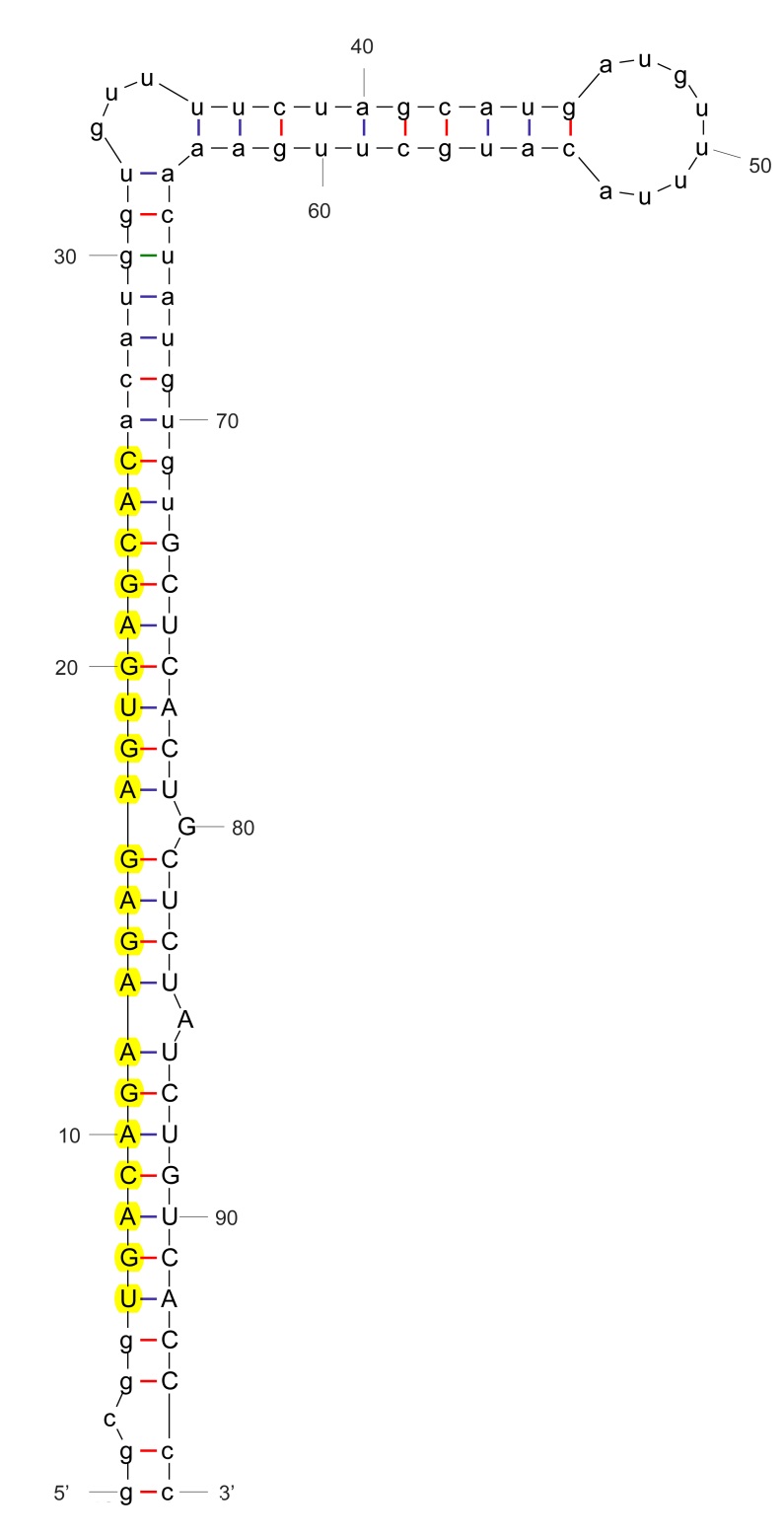


sha-miR156g_stu


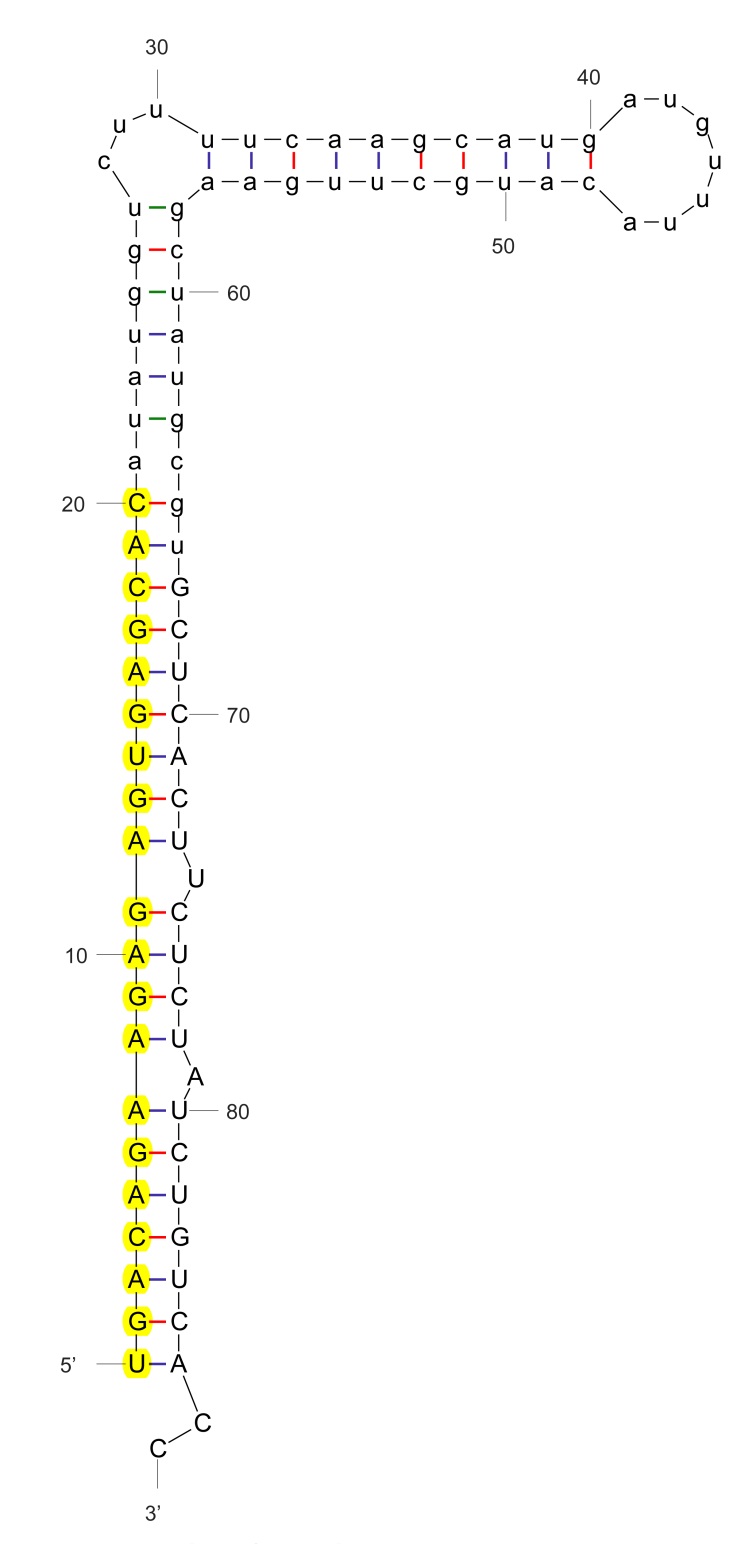


sha-miR156h_stu


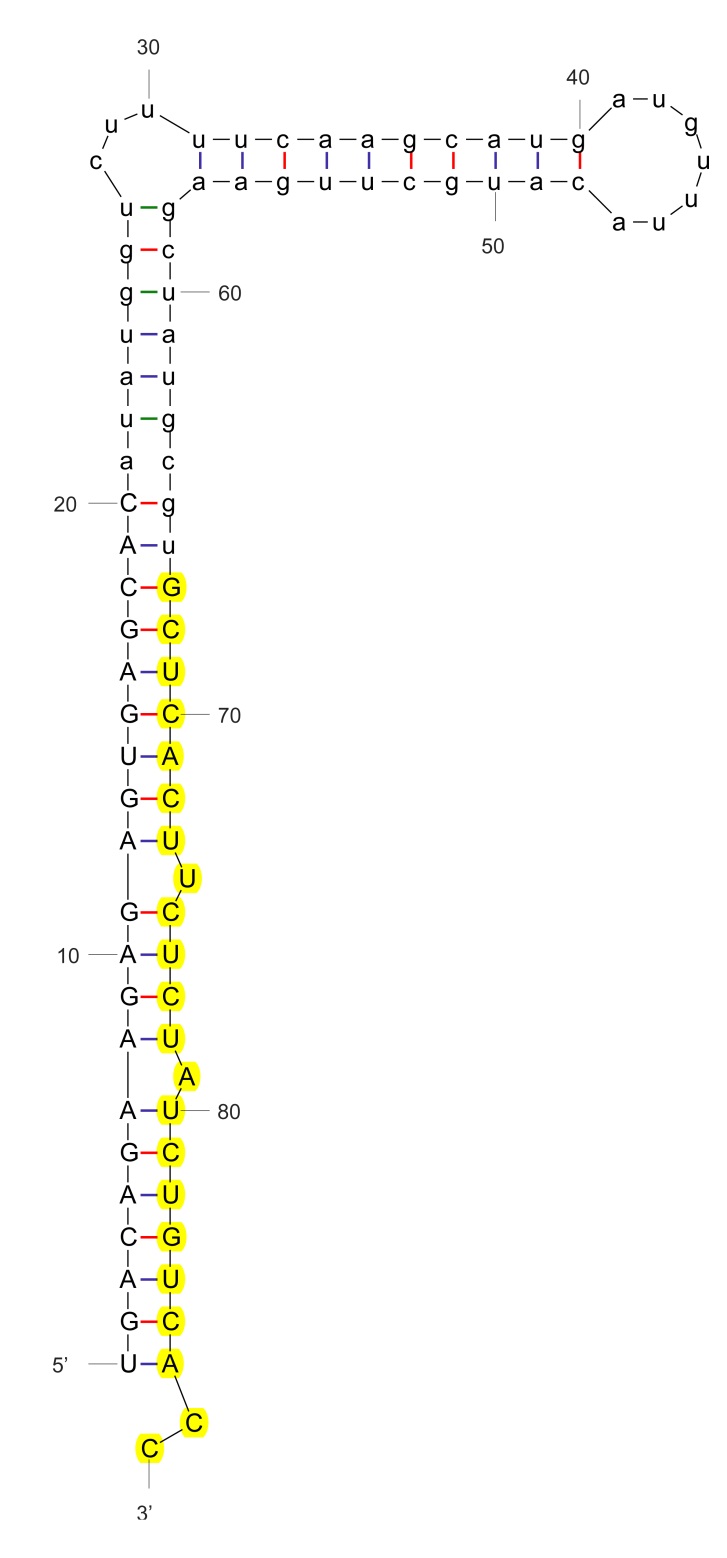


sha-miR156h-3p_stu


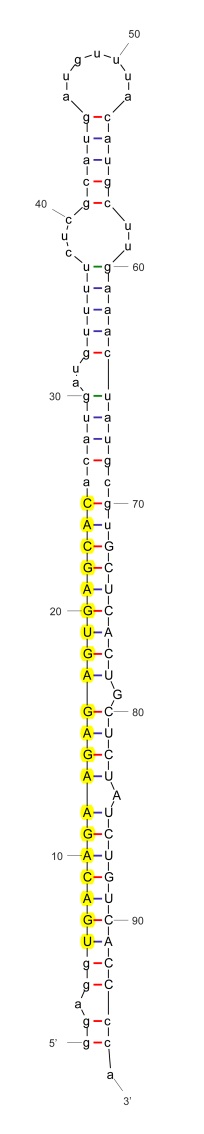


sha-miR156i_stu


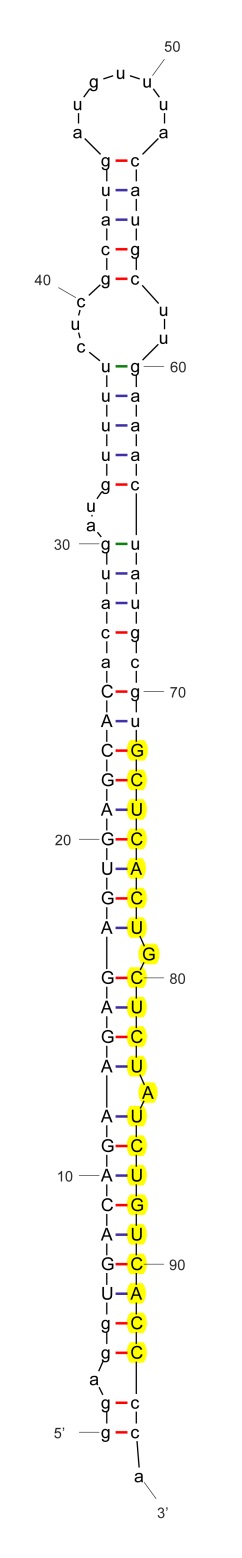


sha-miR156i-3p_stu


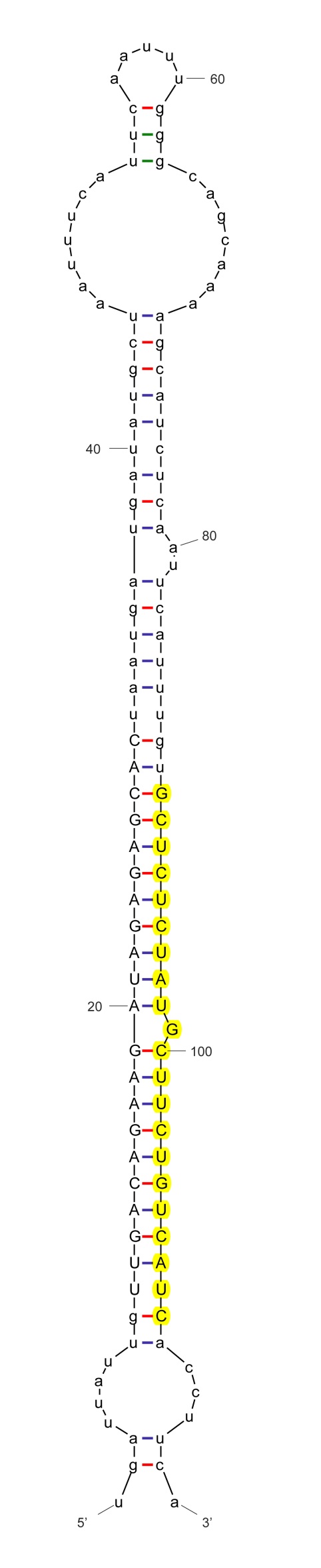


sha-miR156i-p3_nta


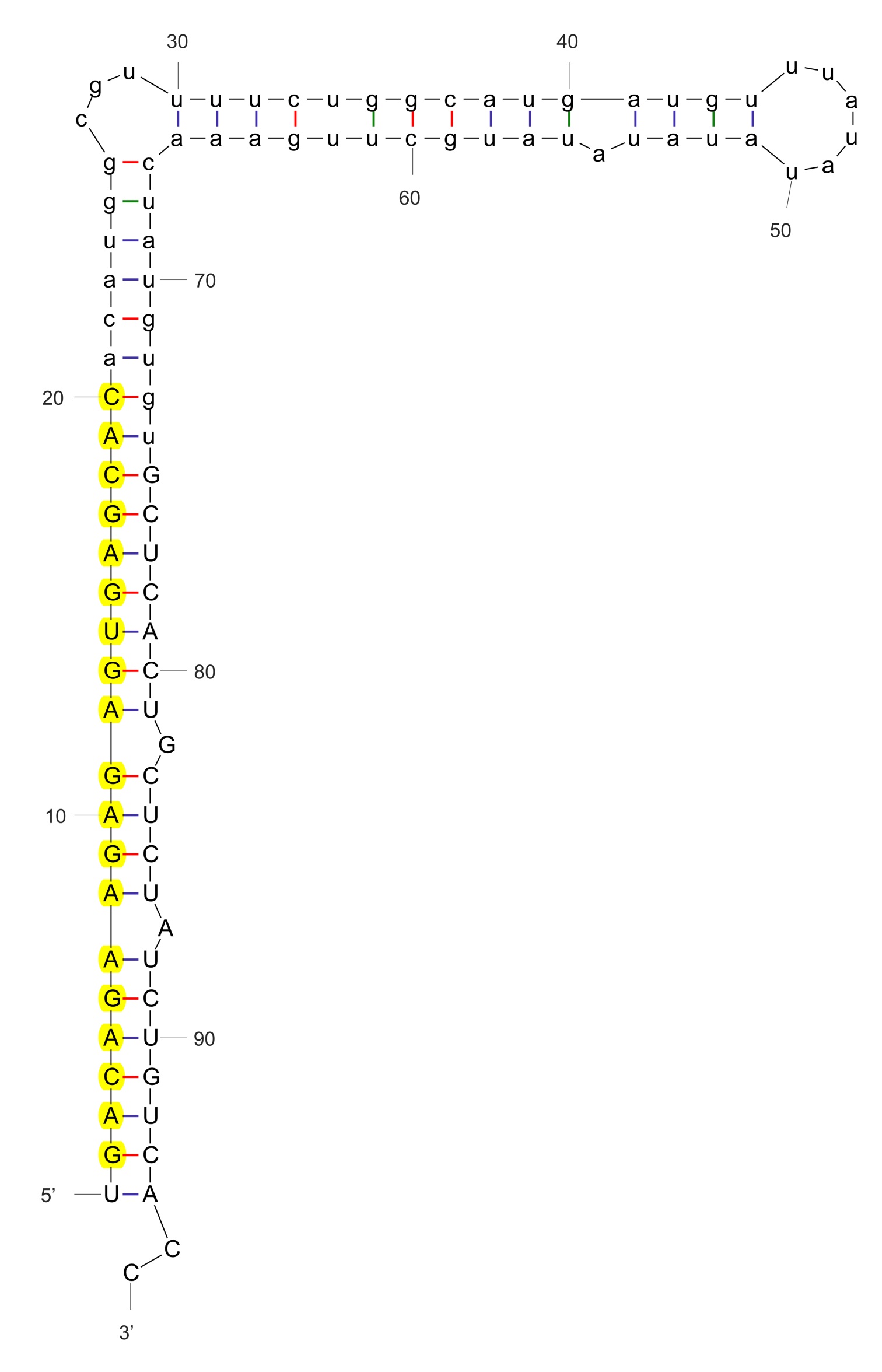


sha-miR156j_stu


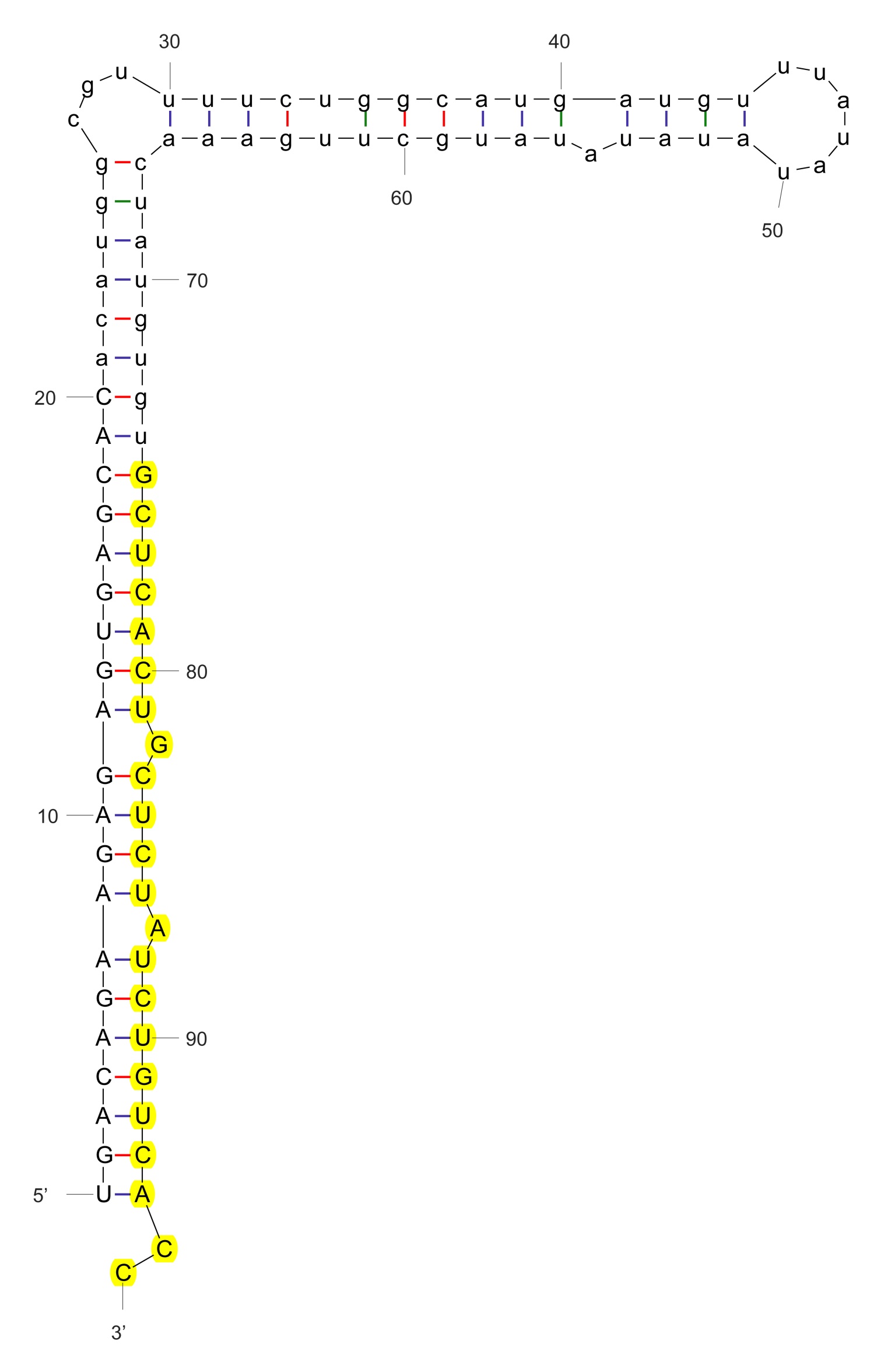


sha-miR156j-3p_stu


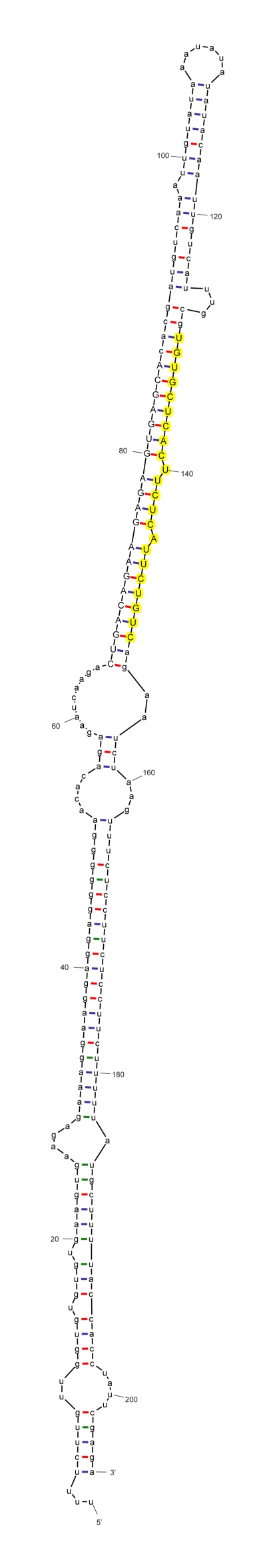


sha-miR156j-p3_cme


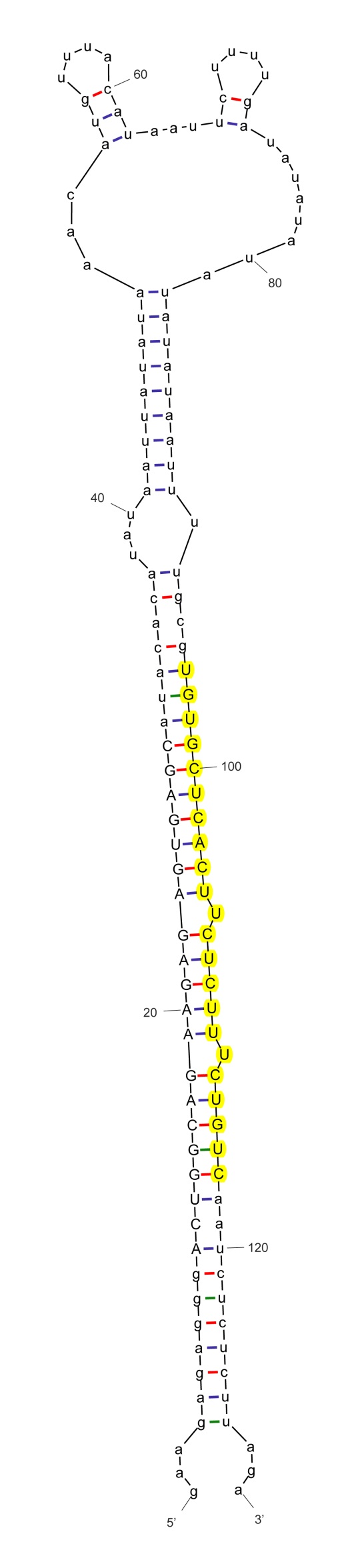


sha-miR156j-p3_cme


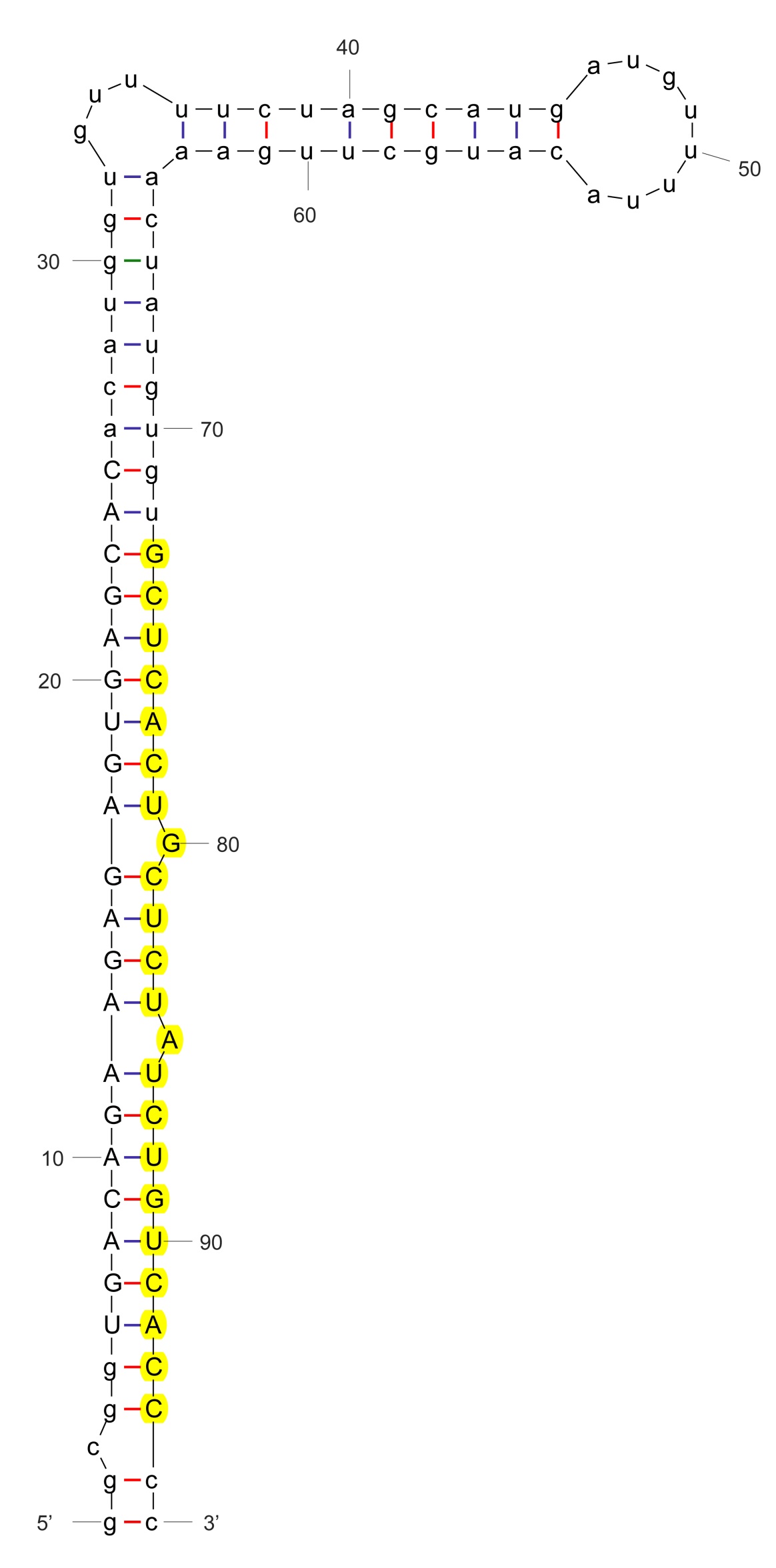


sha-miR156k-3p_stu


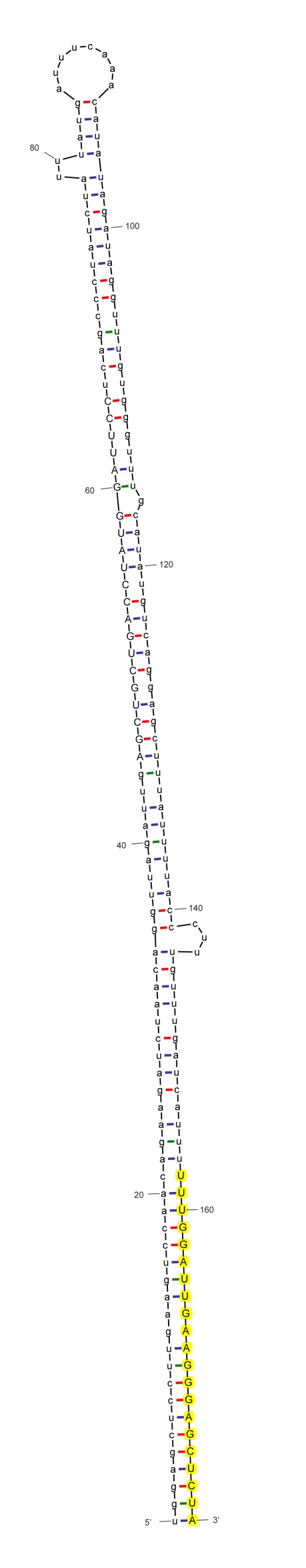


sha-miR159


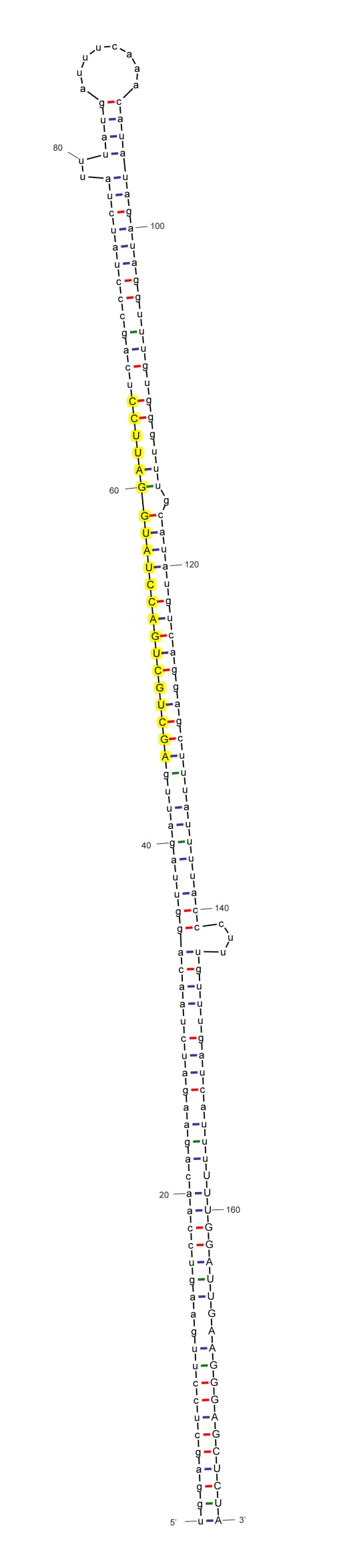


sha-miR159-p5


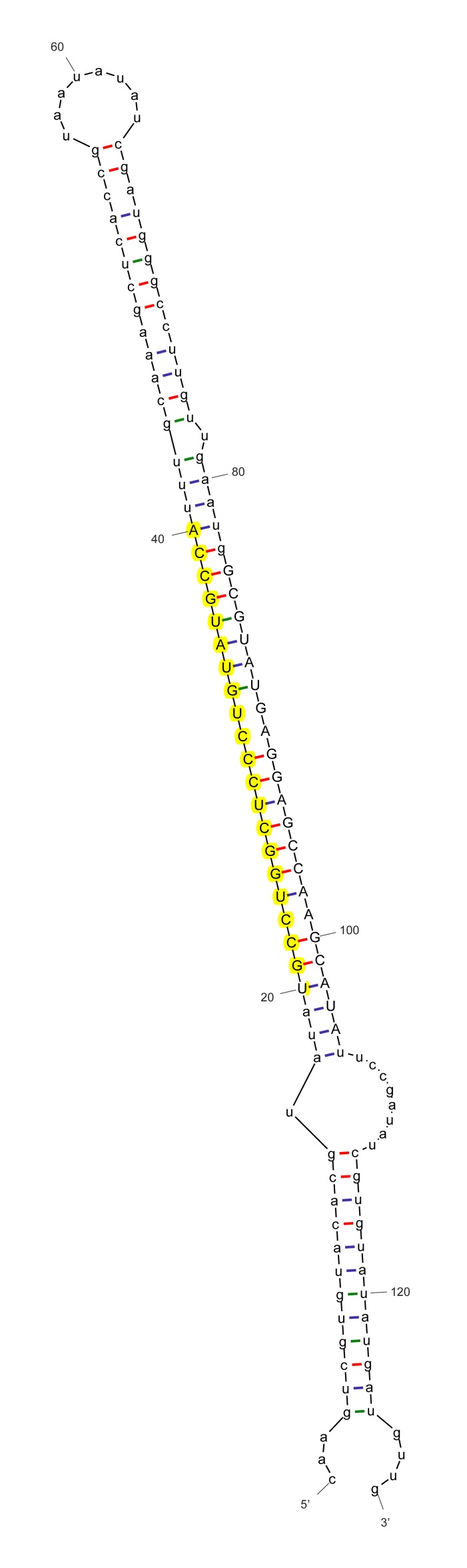


sha-miR160a


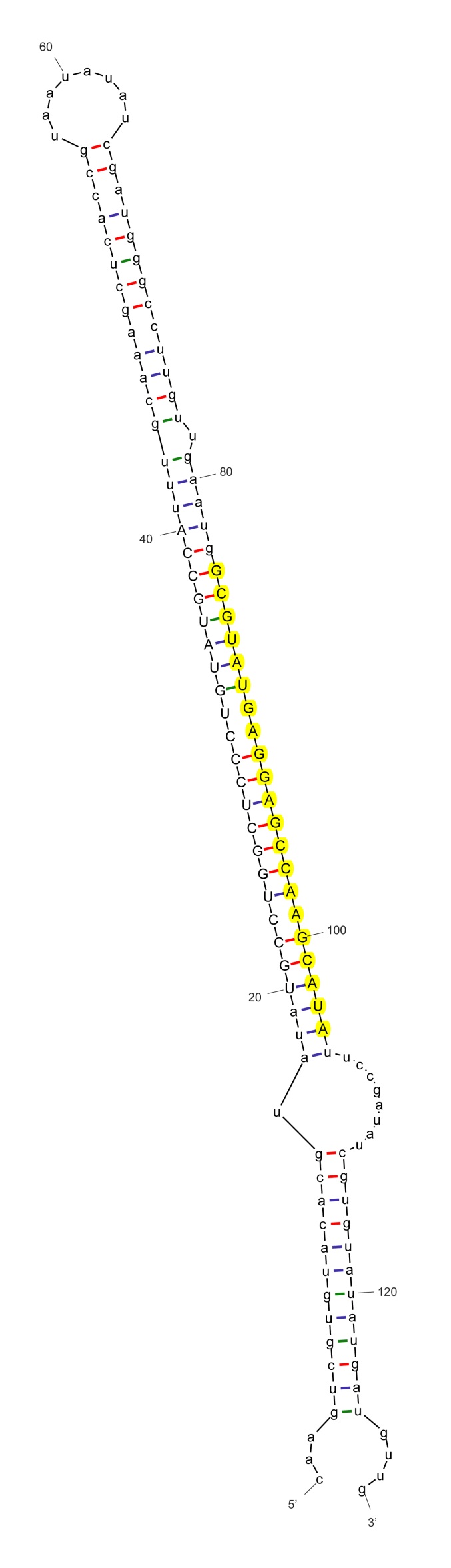


sha-miR160a-3p_stu


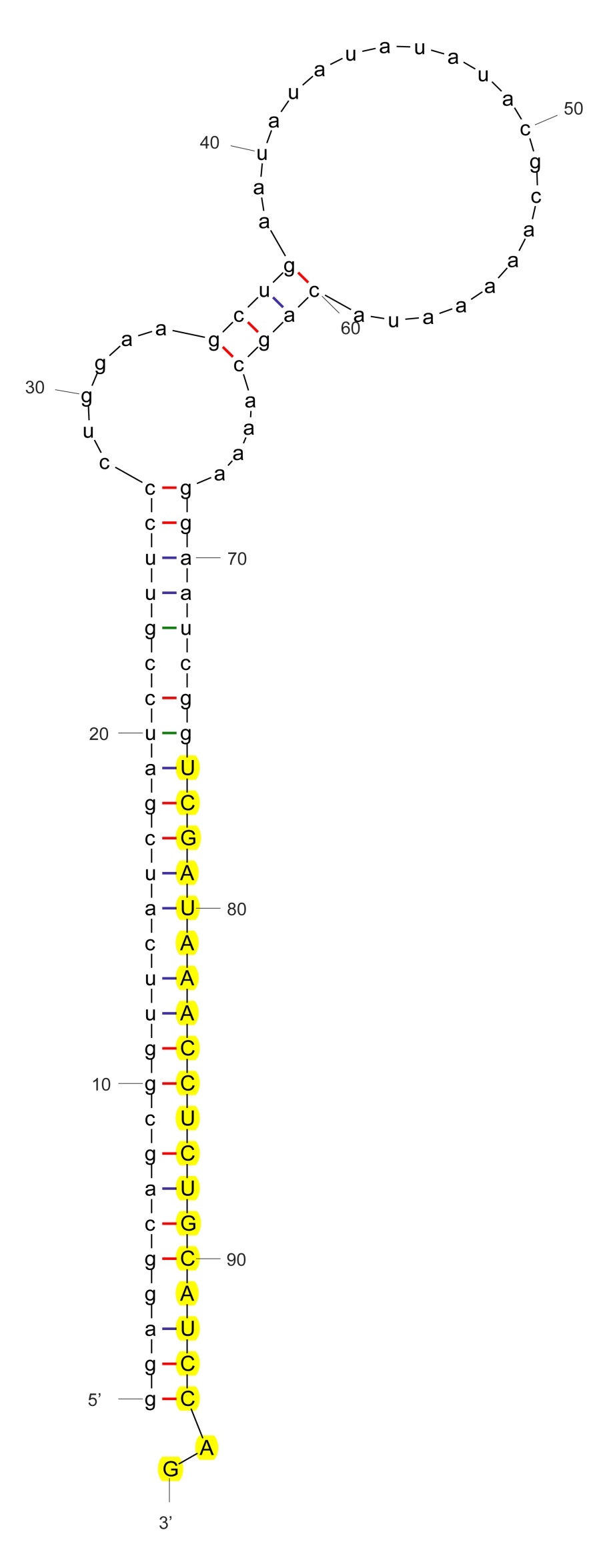


sha-miR162


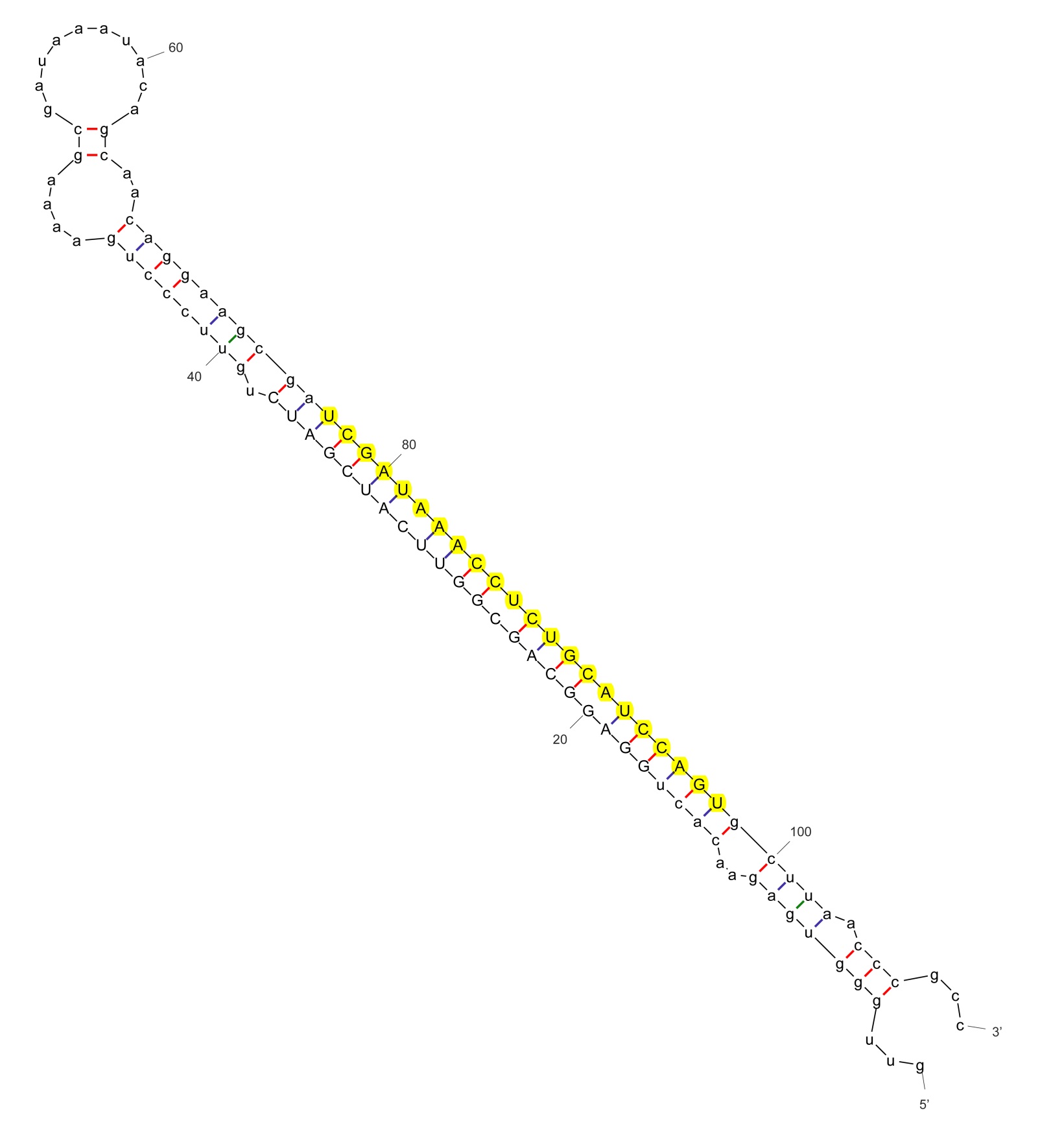


sha-miR162_vvi


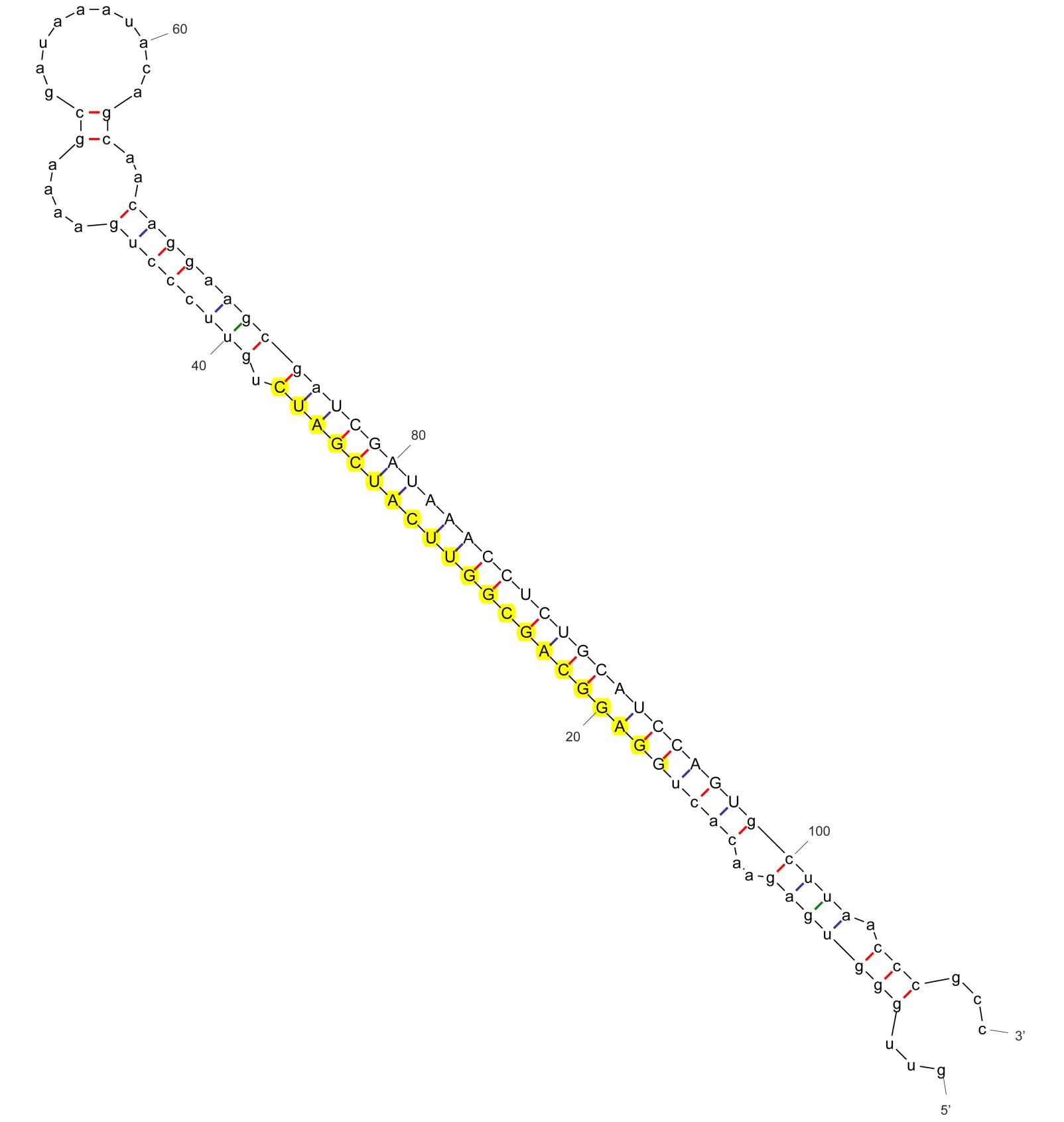


sha-miR162-p5_cme


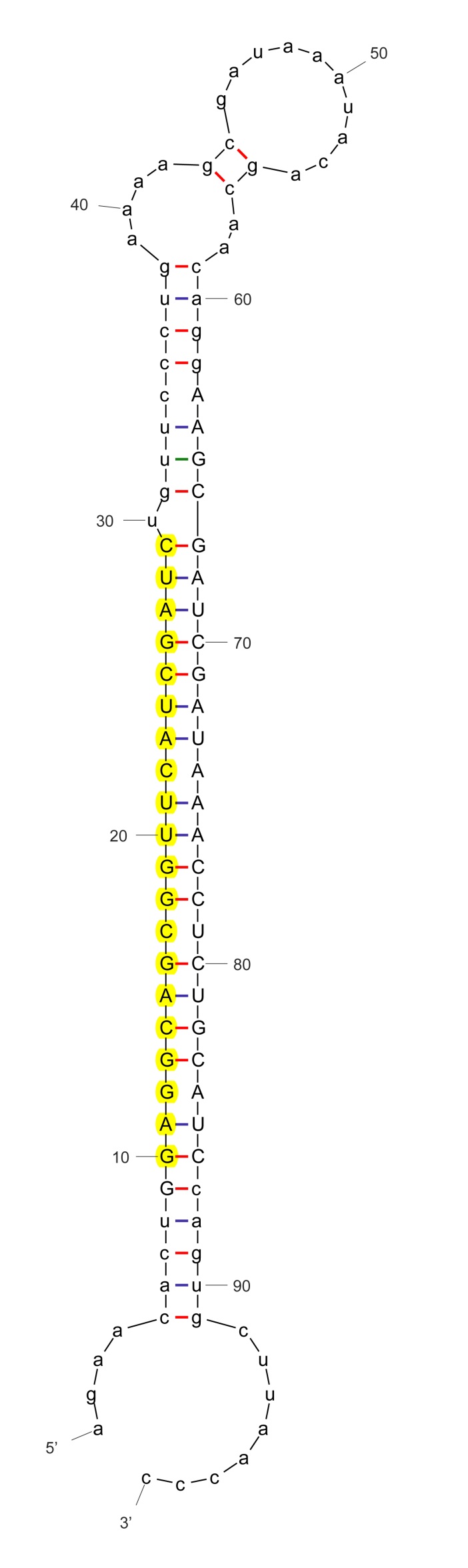


sha-miR162a-5p_stu


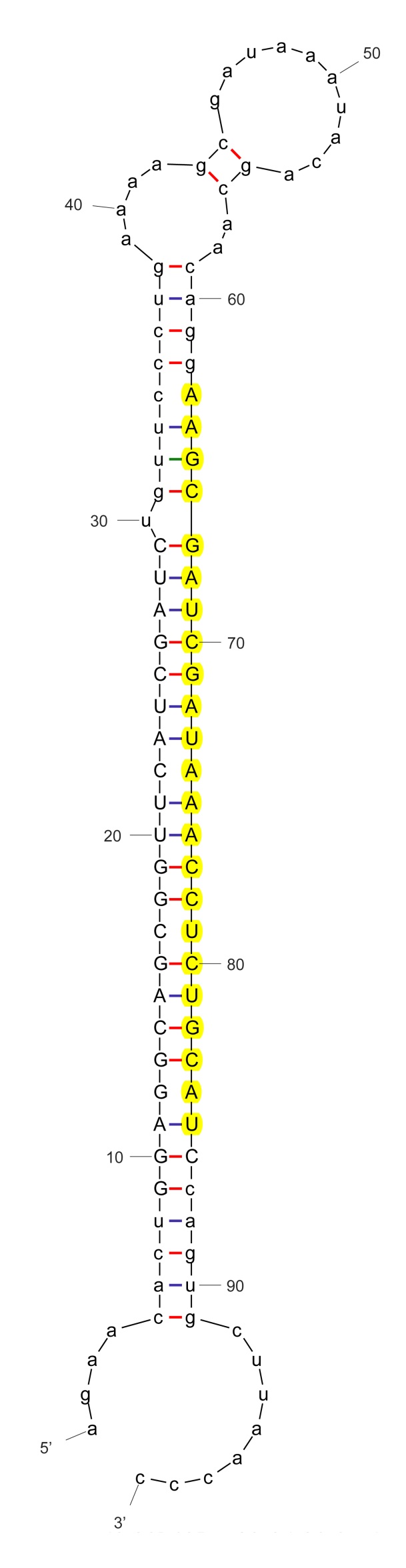


sha-miR162a-p3_stu


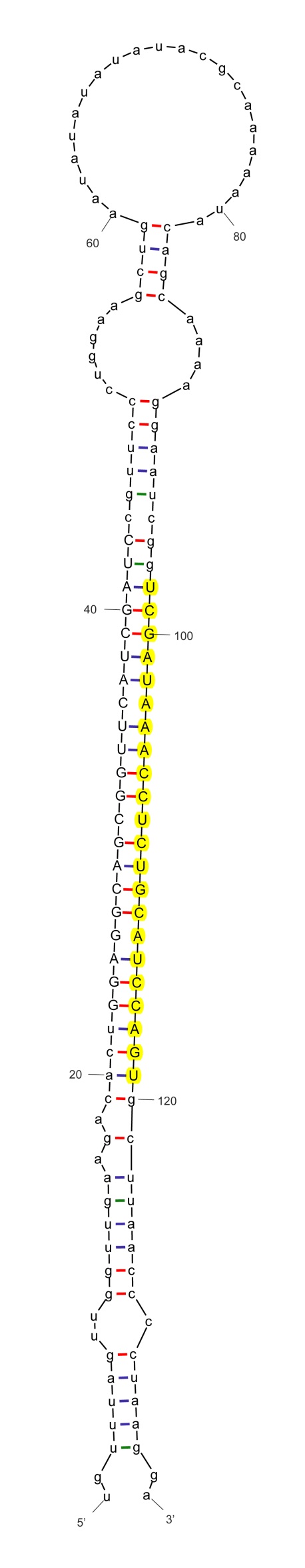


sha-miR162b-3p_stu


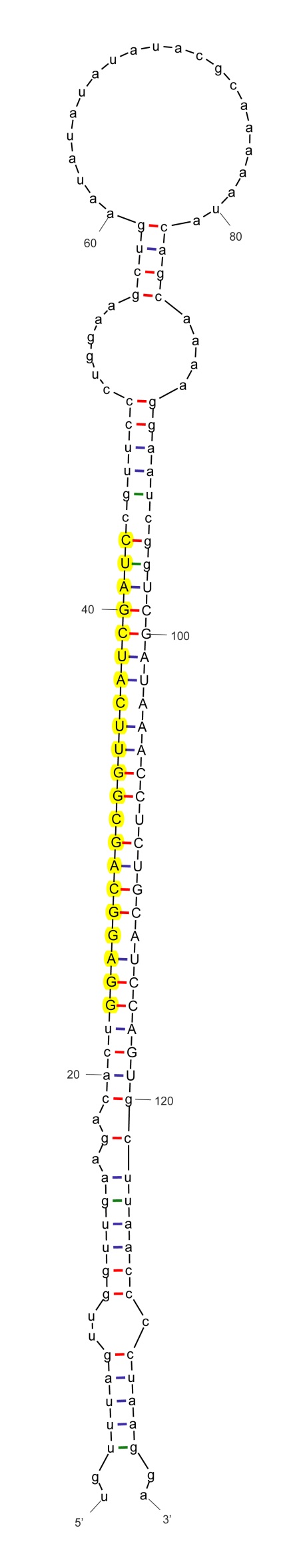


sha-miR162b-5p_stu


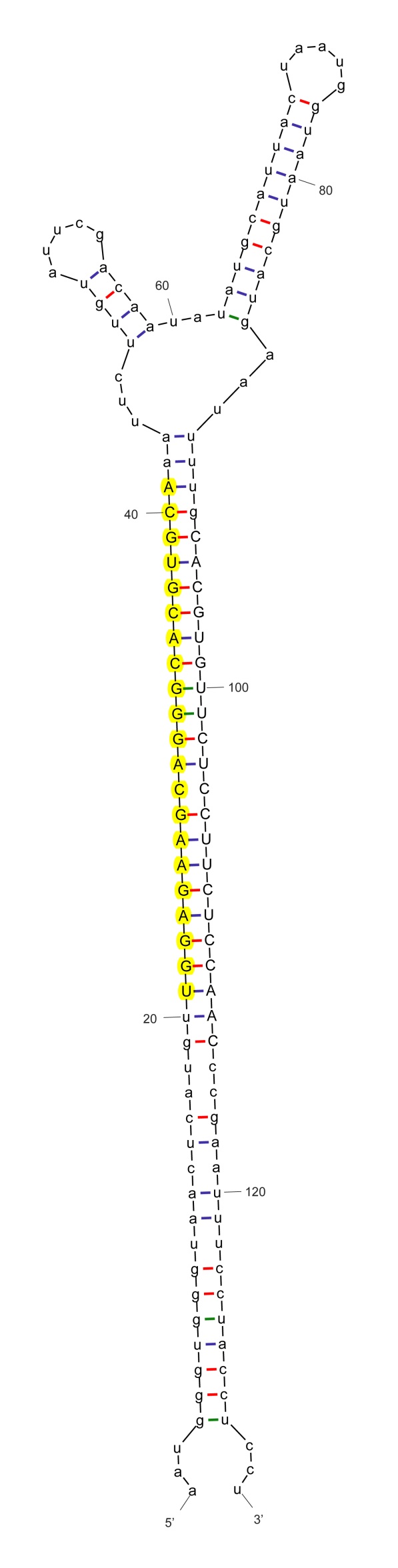


sha-miR164a_nta


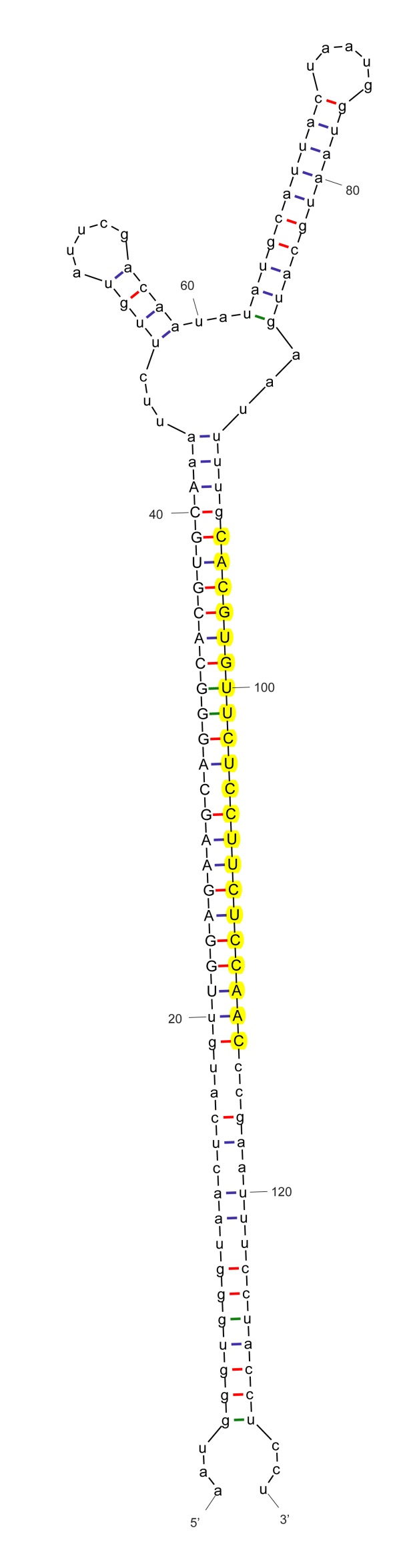


sha-miR164c-3p_aly


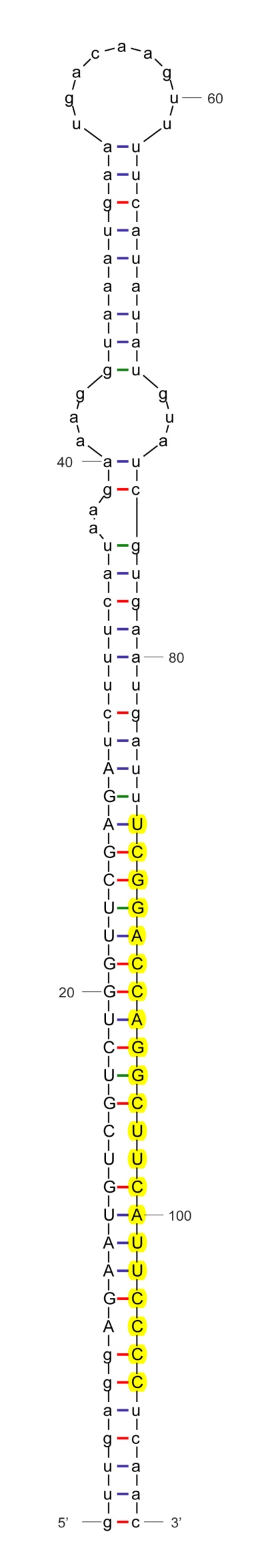


sha-miR166a


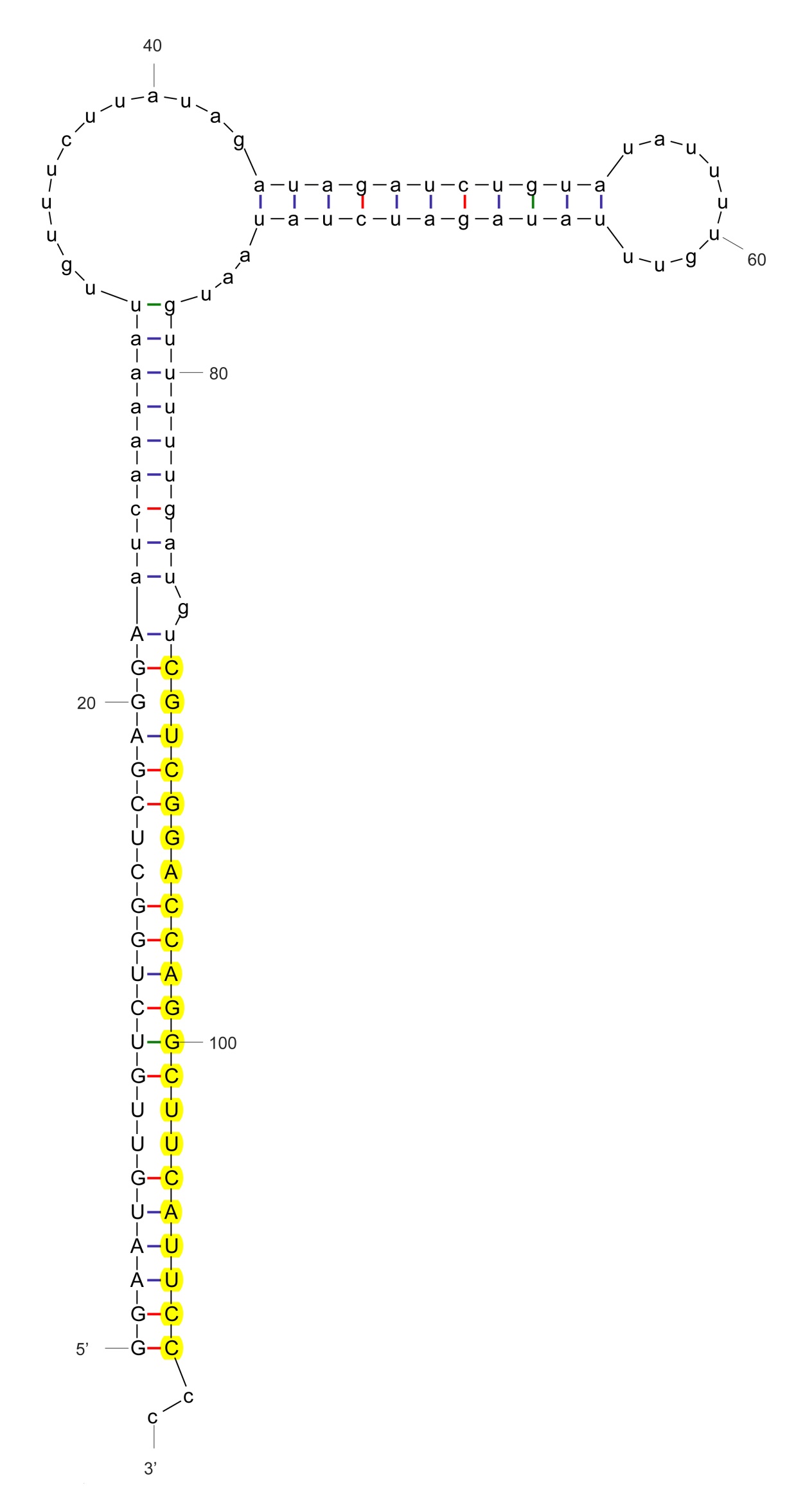


sha-miR166a-3p_stu


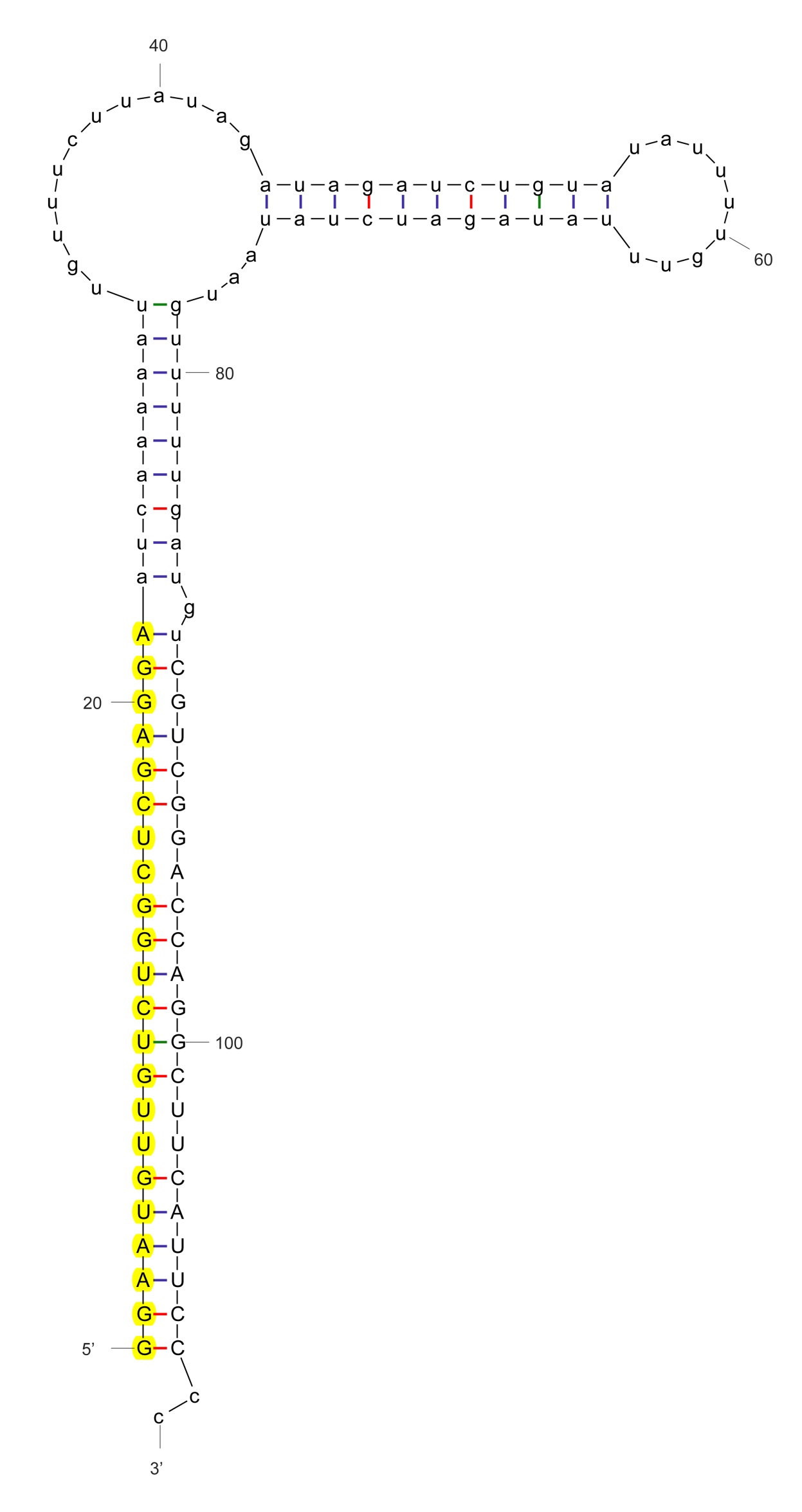


sha-miR166a-5p_stu


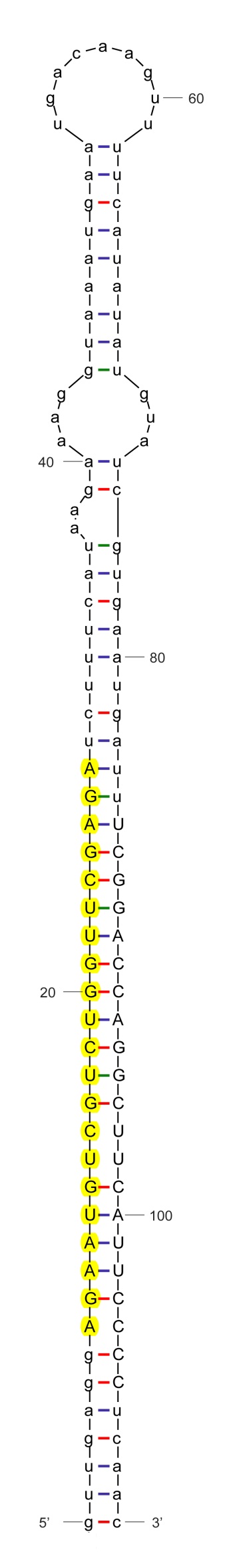


sha-miR166a-p5


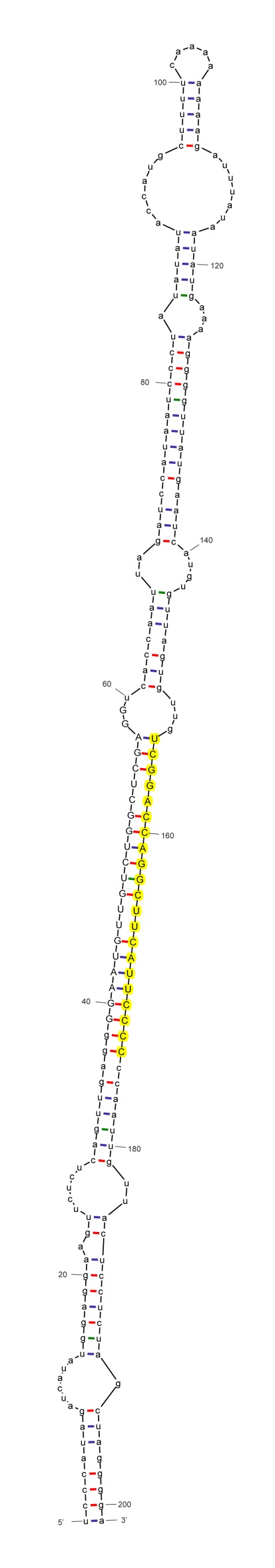


sha-miR166b


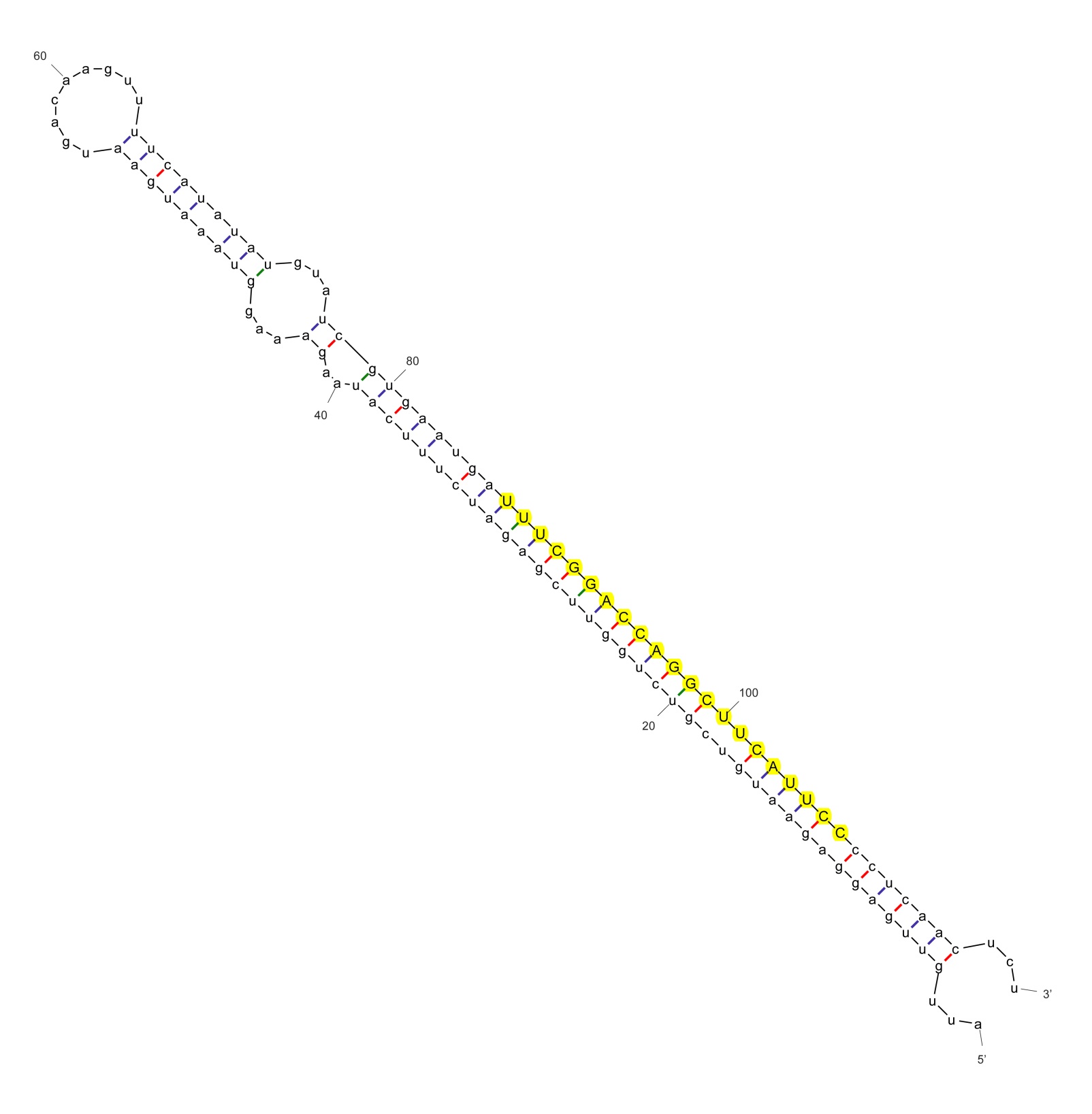


sha-miR166b_stu


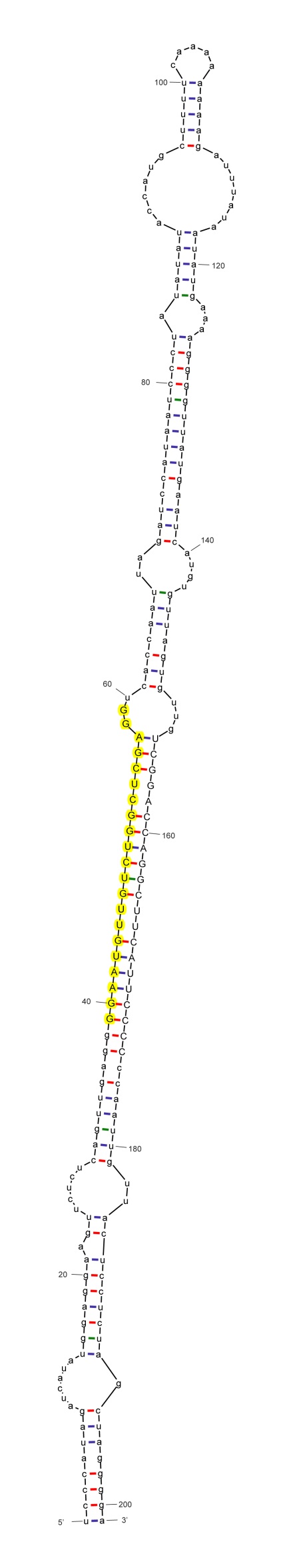


sha-miR166b-p5


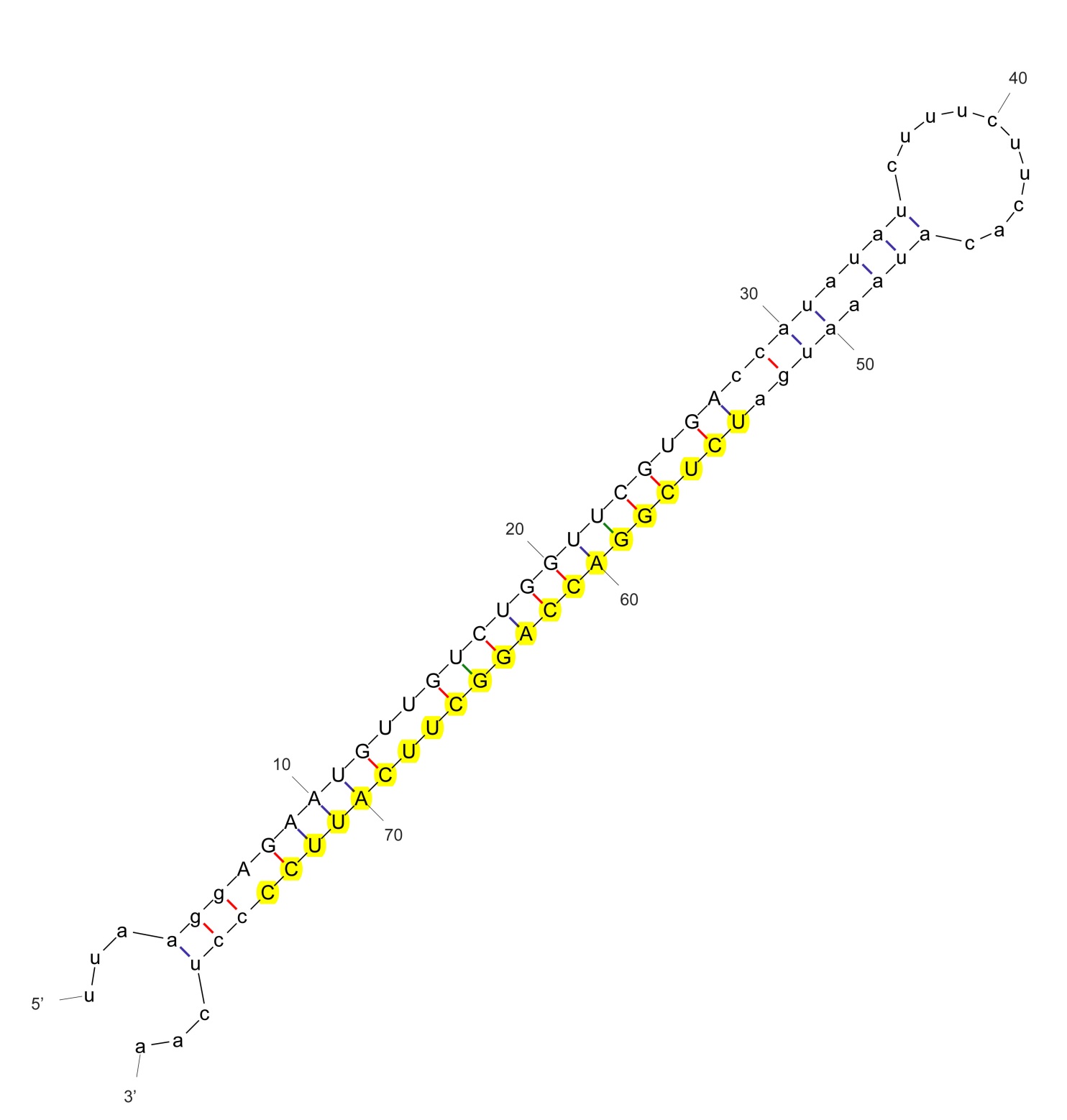


sha-miR166g_nta


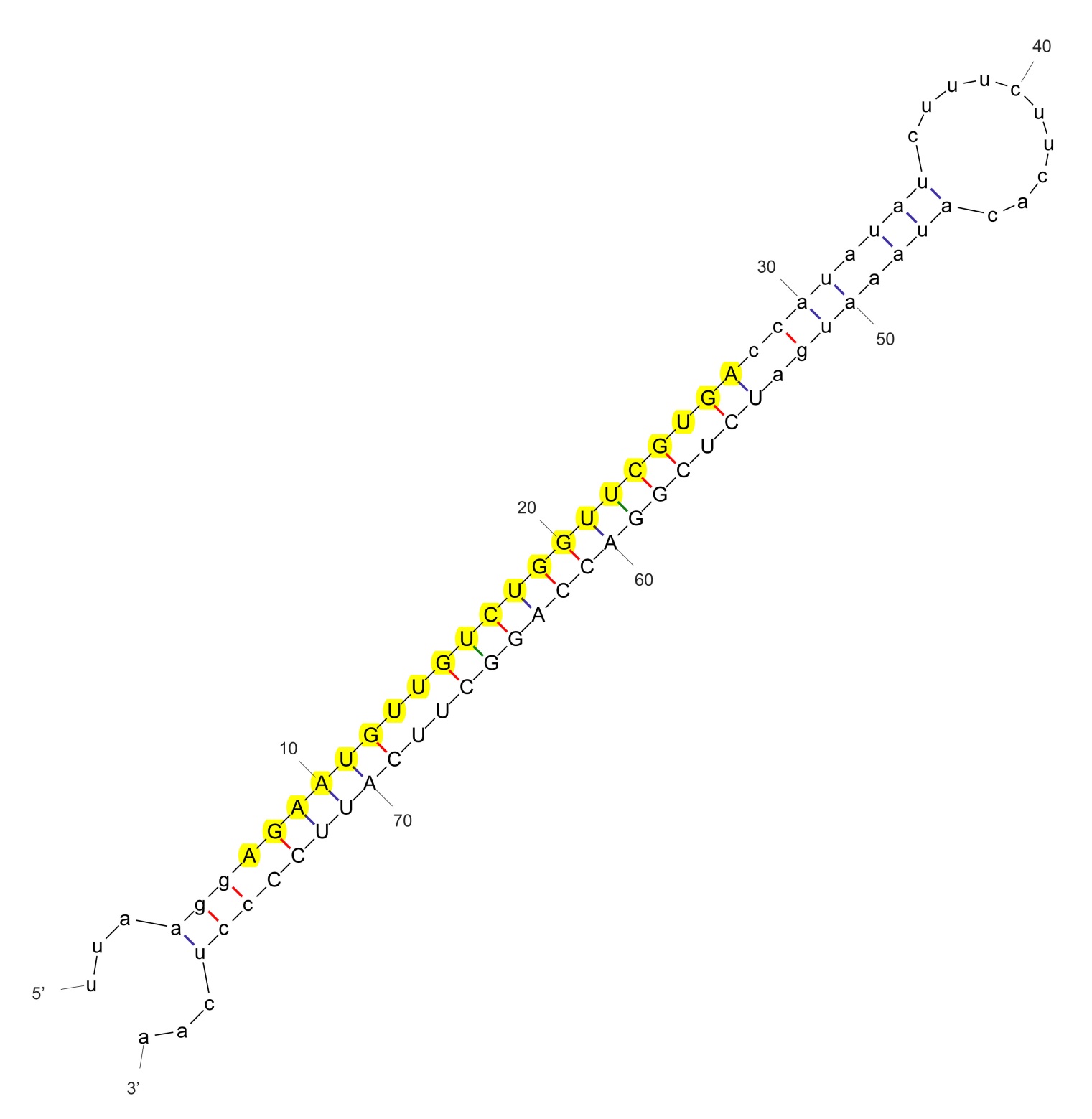


sha-miR166g-p5_nta


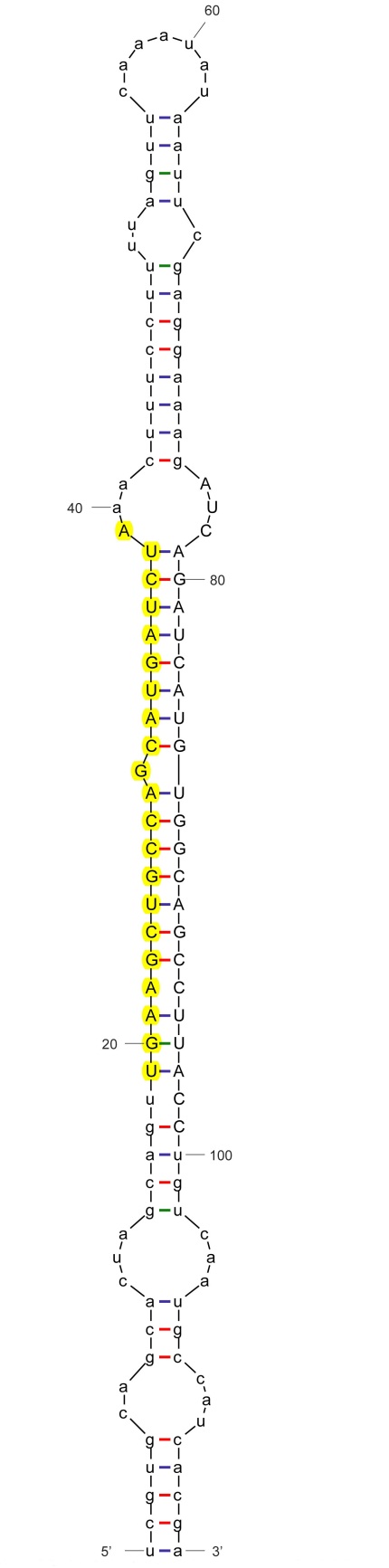


sha-miR167


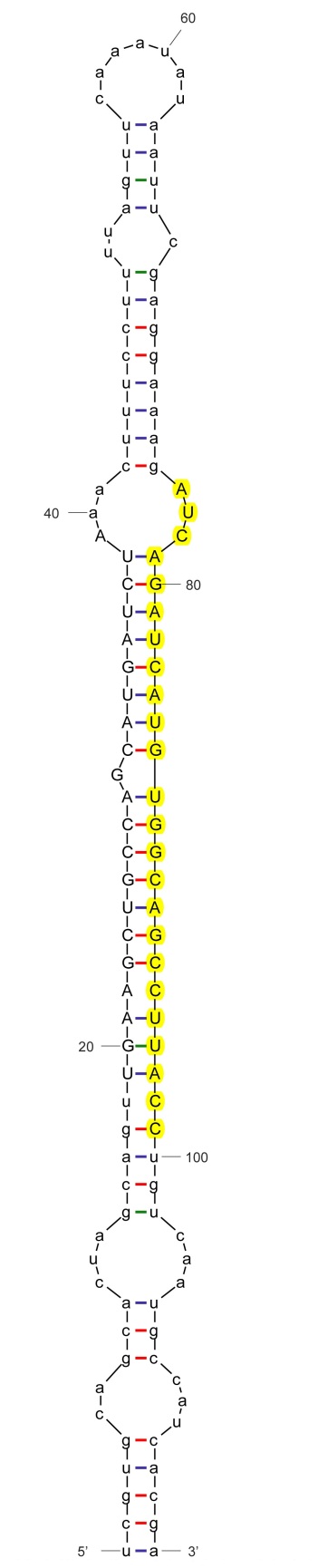


sha-miR167-p3


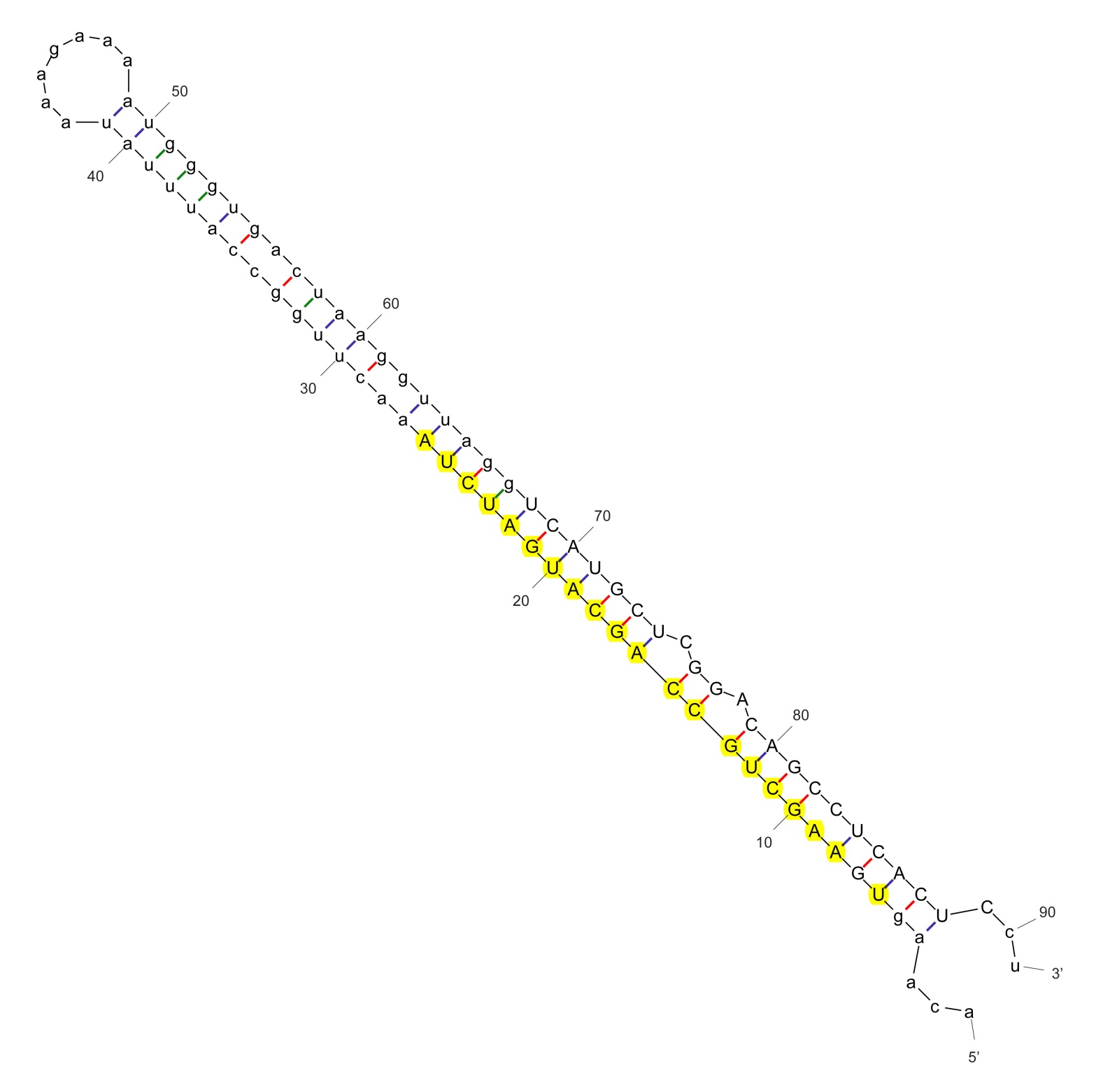


sha-miR167b_nta


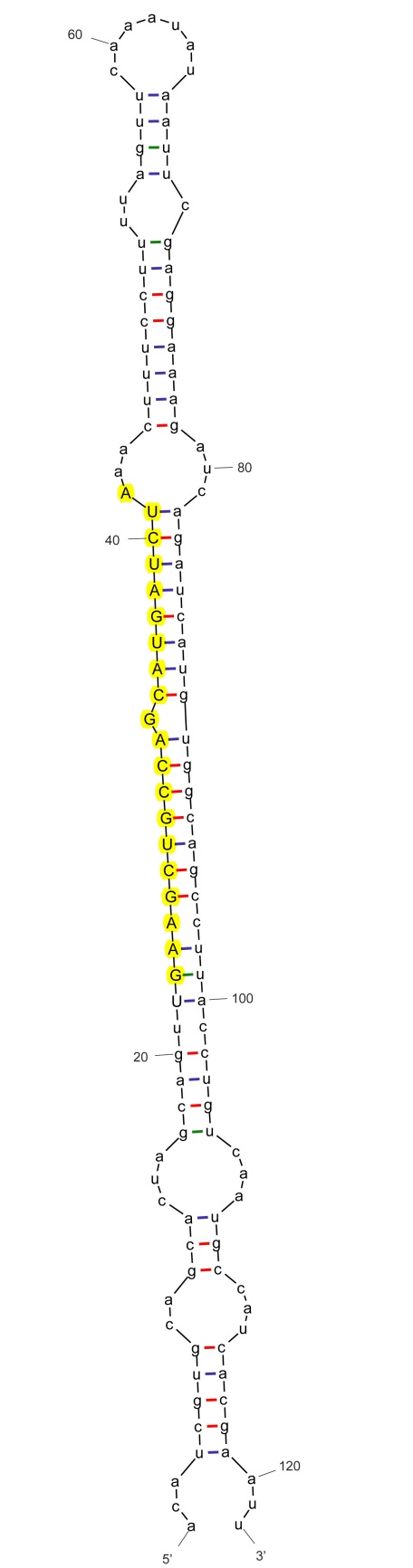


sha-miR167b_nta


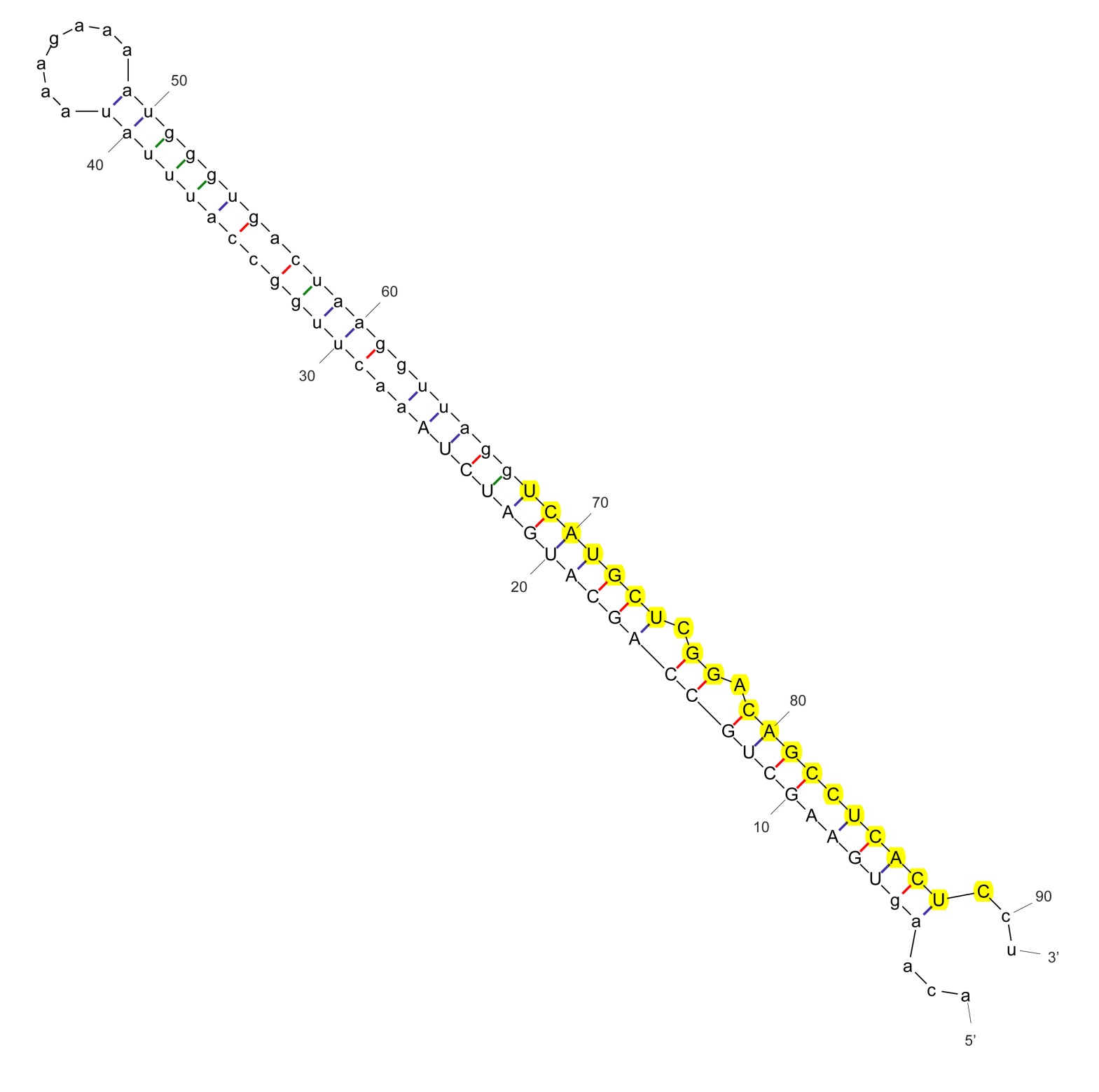


sha-miR167b-3p_aly


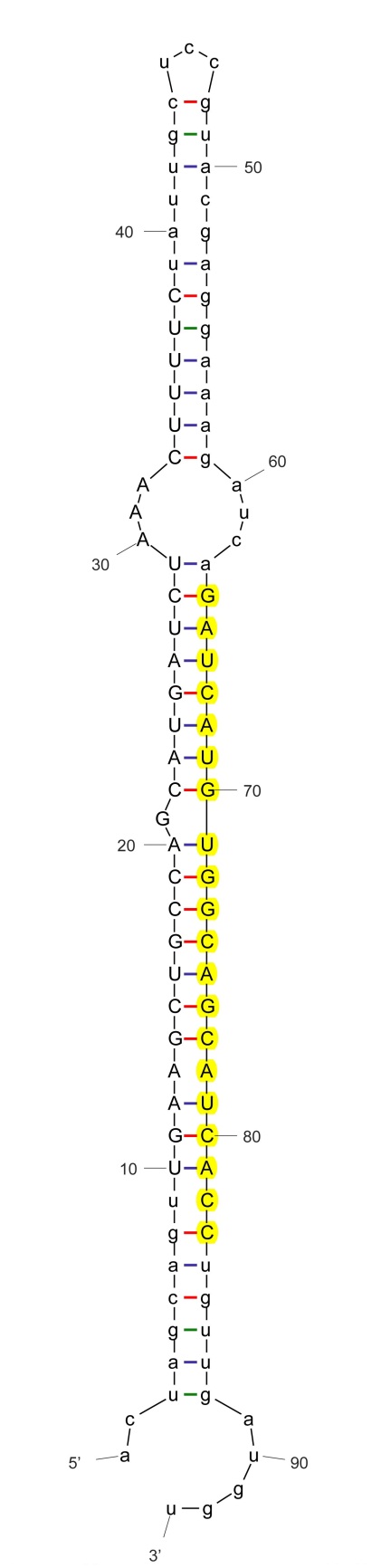


sha-miR167b-3p_stu


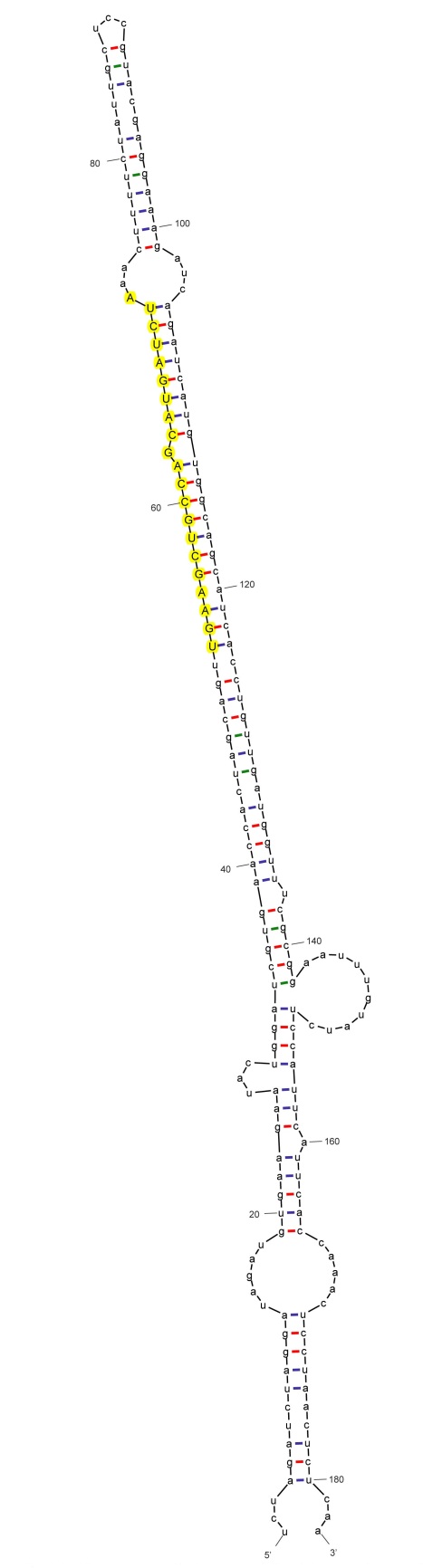


sha-miR167c_vvi


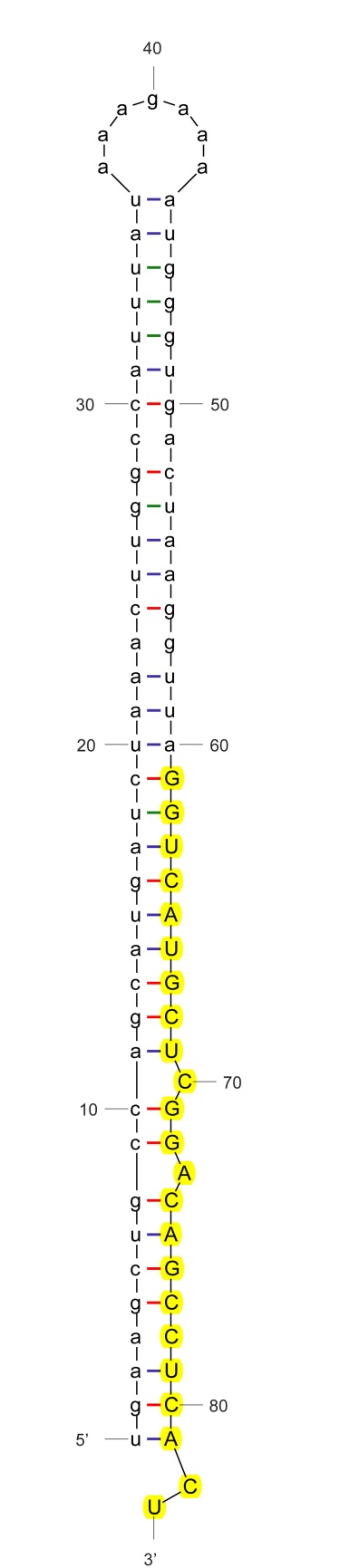


sha-miR167c-3p_stu


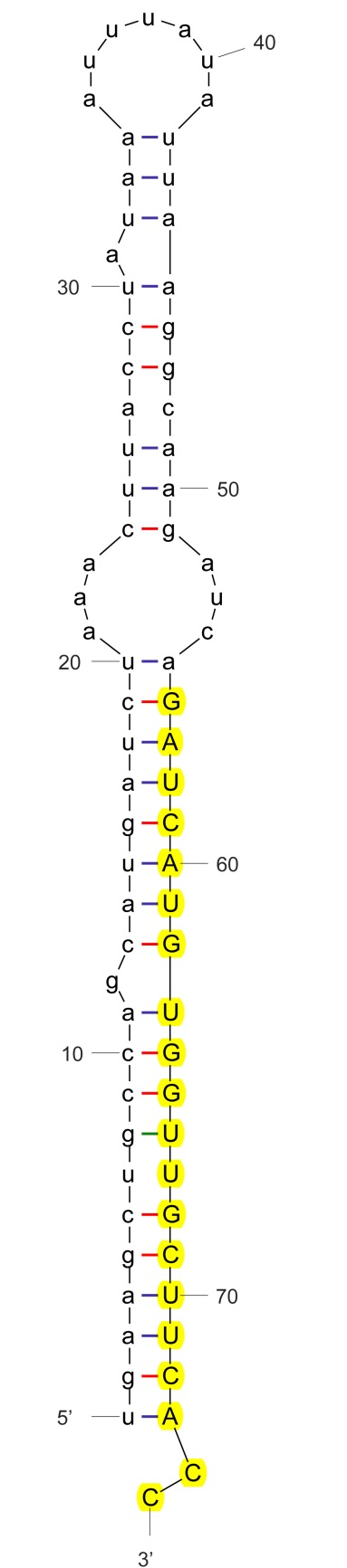


sha-miR167d-3p_stu


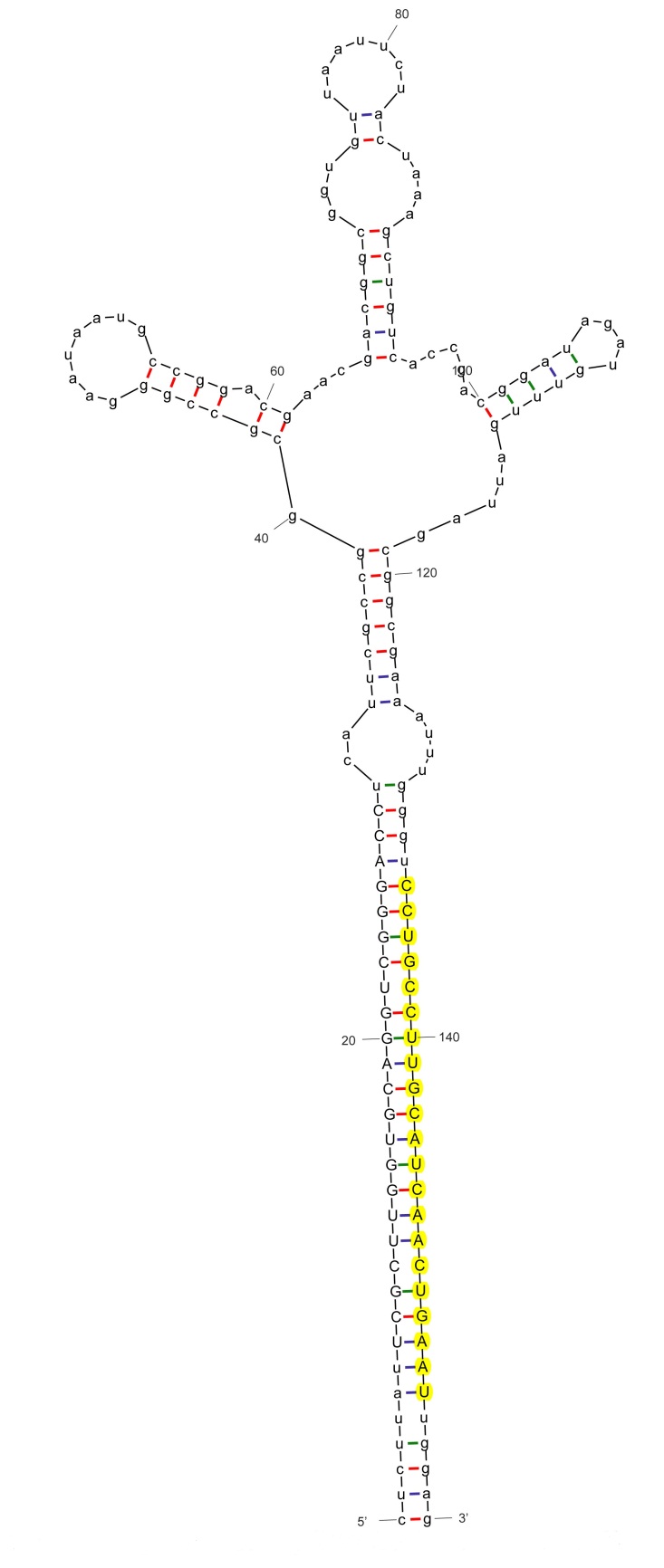


sha-miR168a-3p


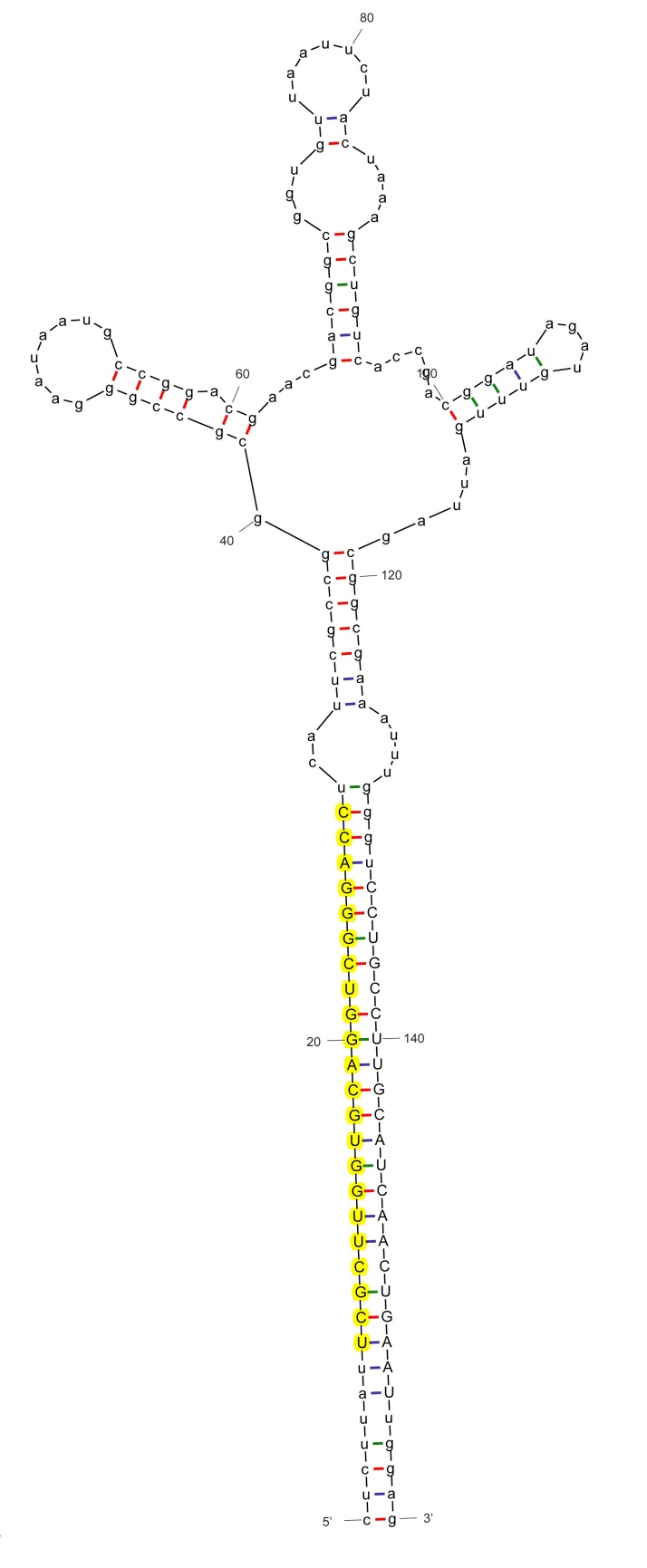


sha-miR168a-5p


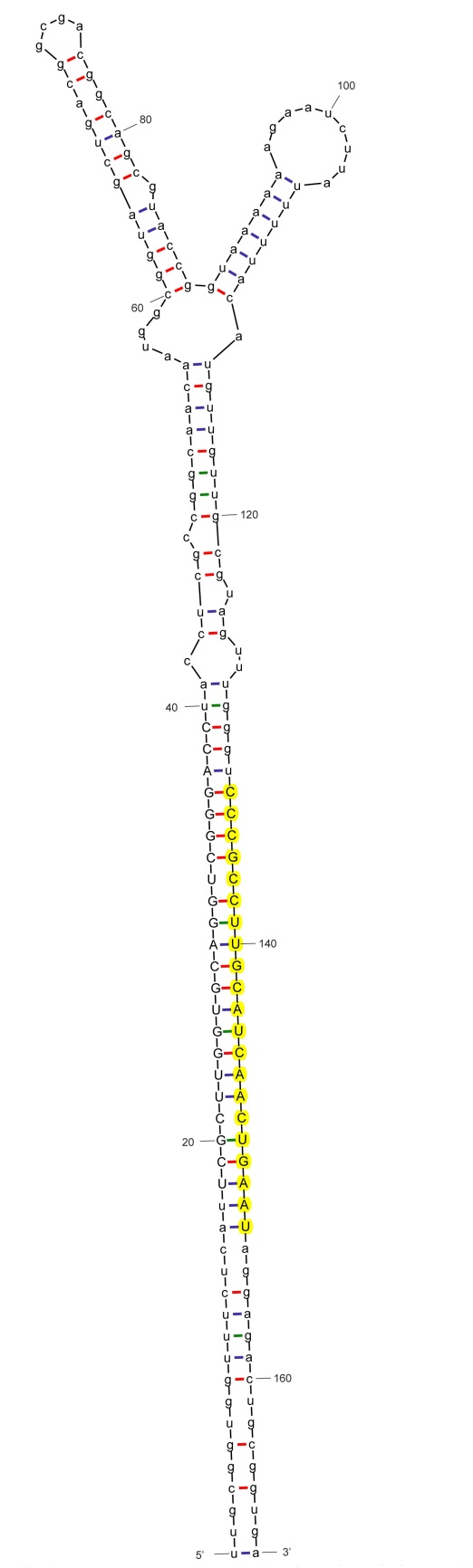


sha-miR168b-3p


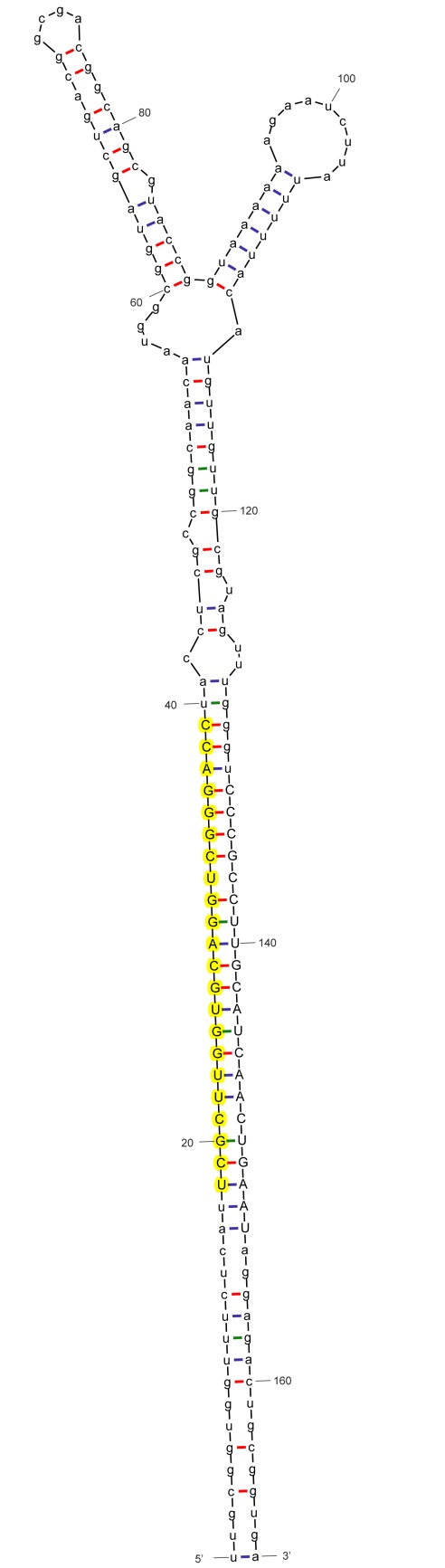


sha-miR168b-5p


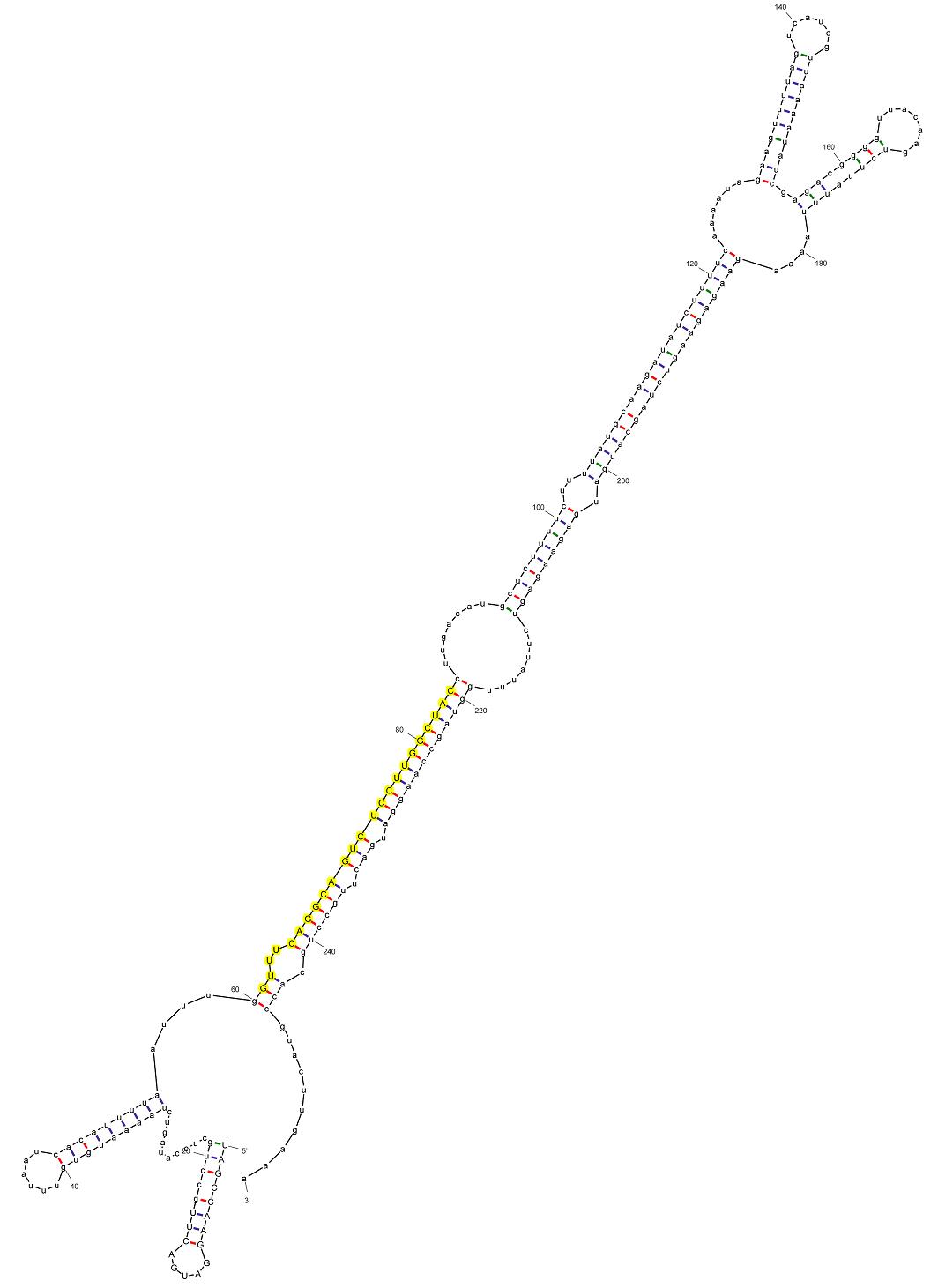


sha-miR169c-3p_stu


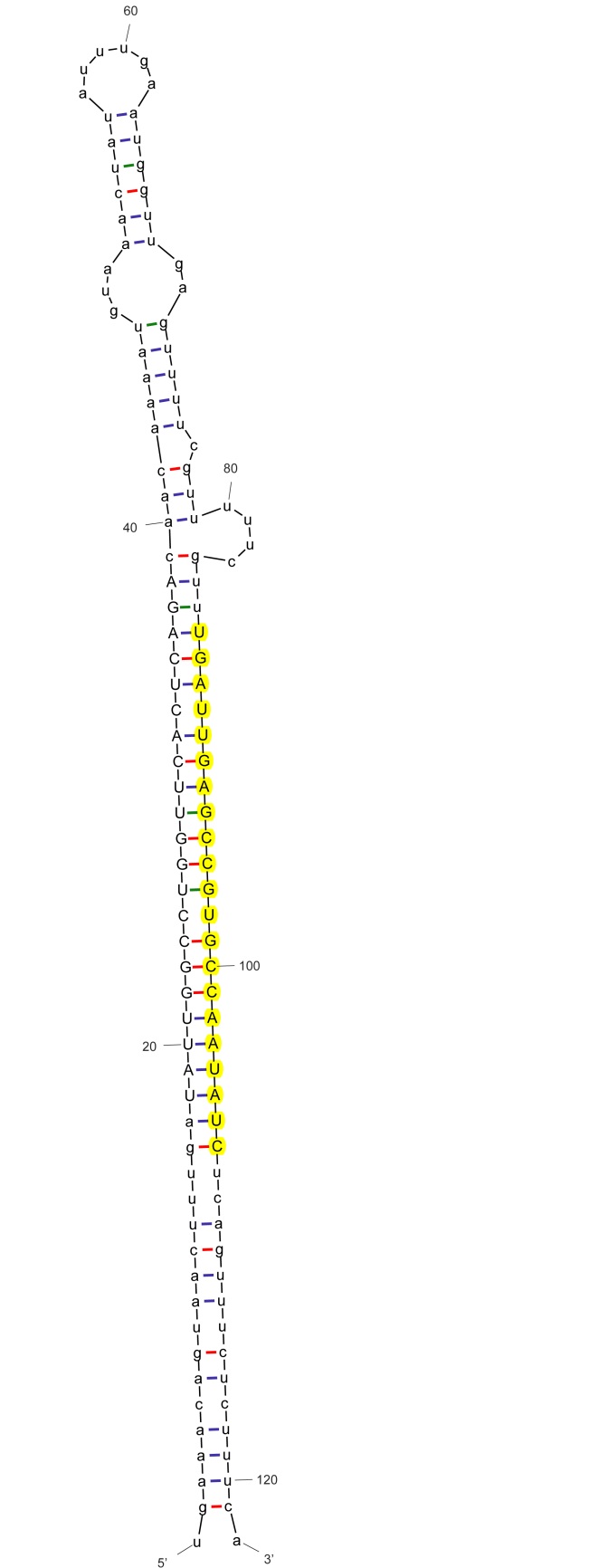


sha-miR171a


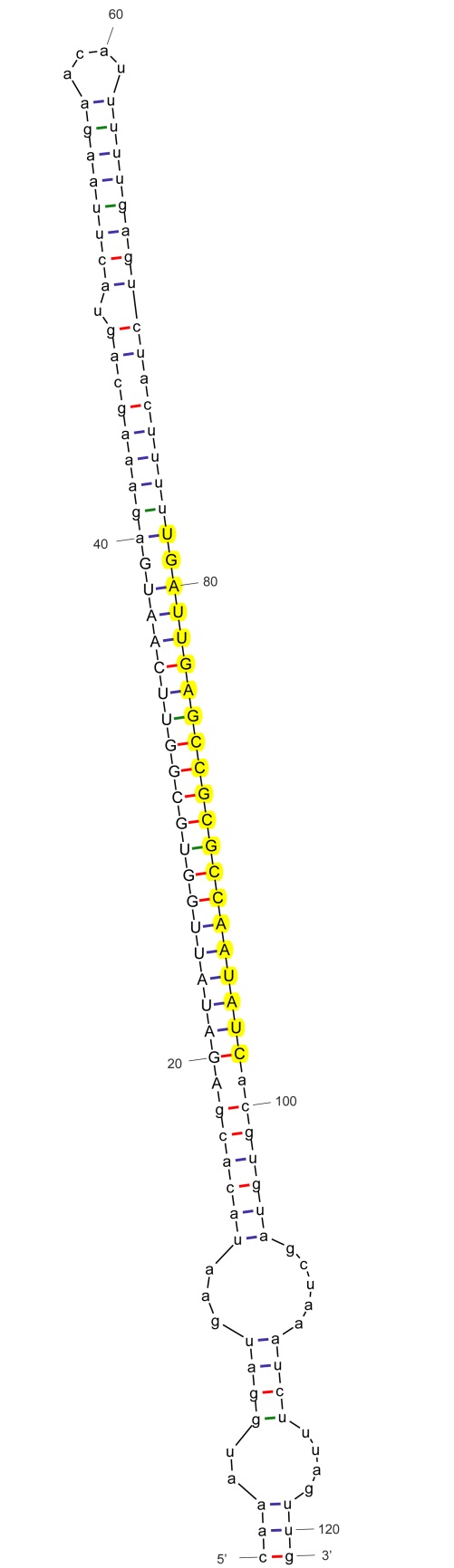


sha-miR171a_nta


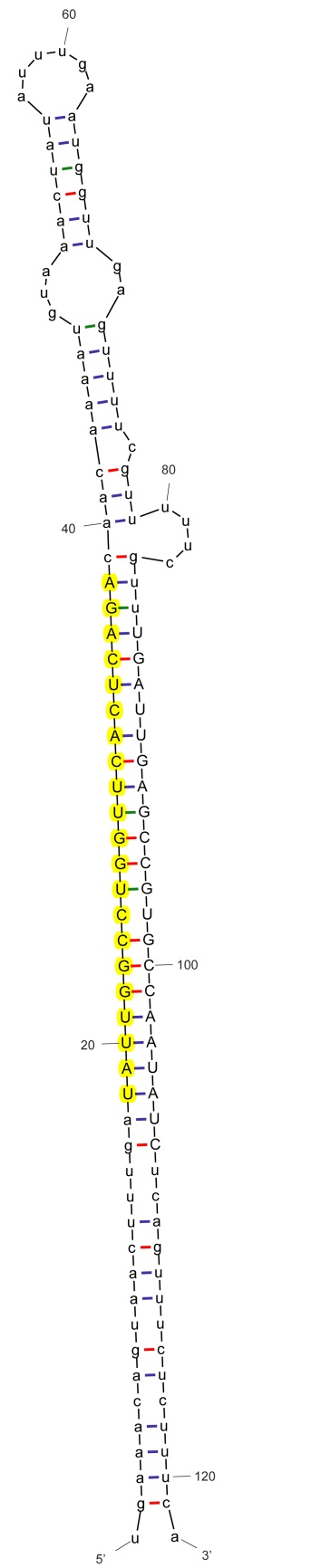


sha-miR171a-5p_stu


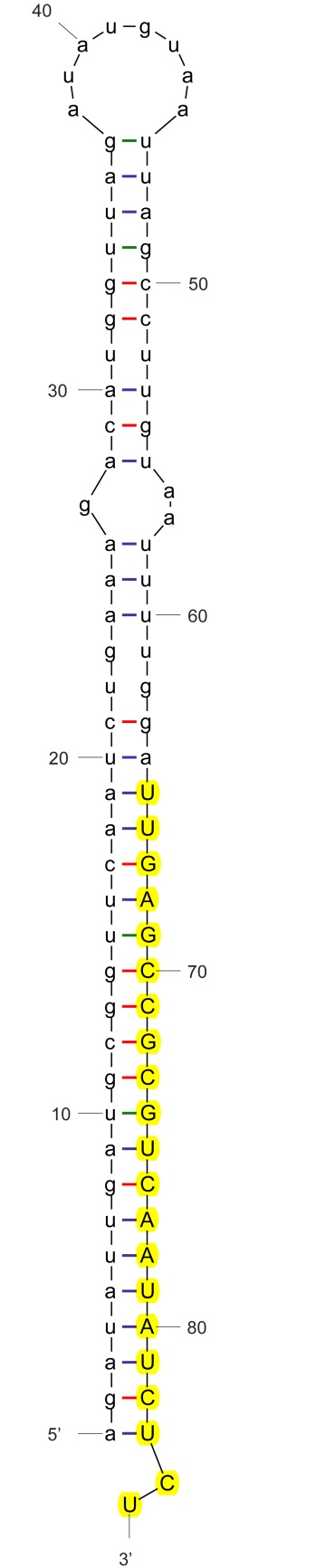


sha-miR171b-3p_stu


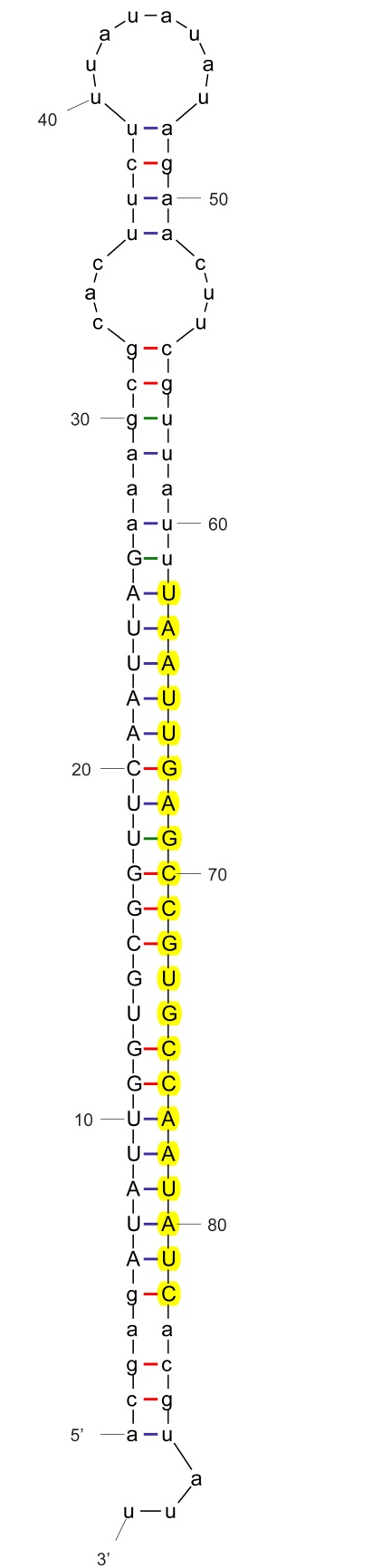


sha-miR171b-p3_mes


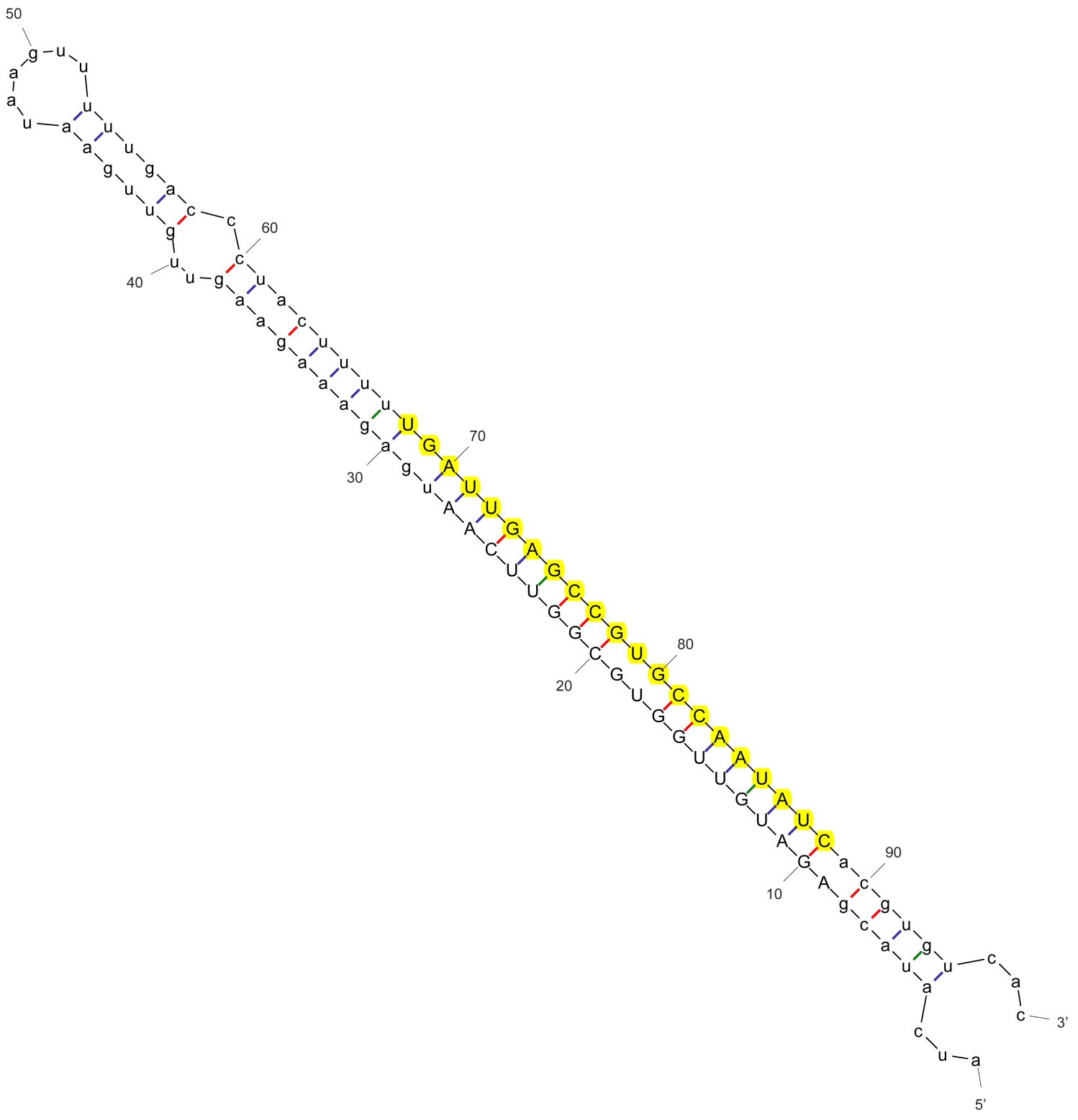


sha-miR171c_mtr


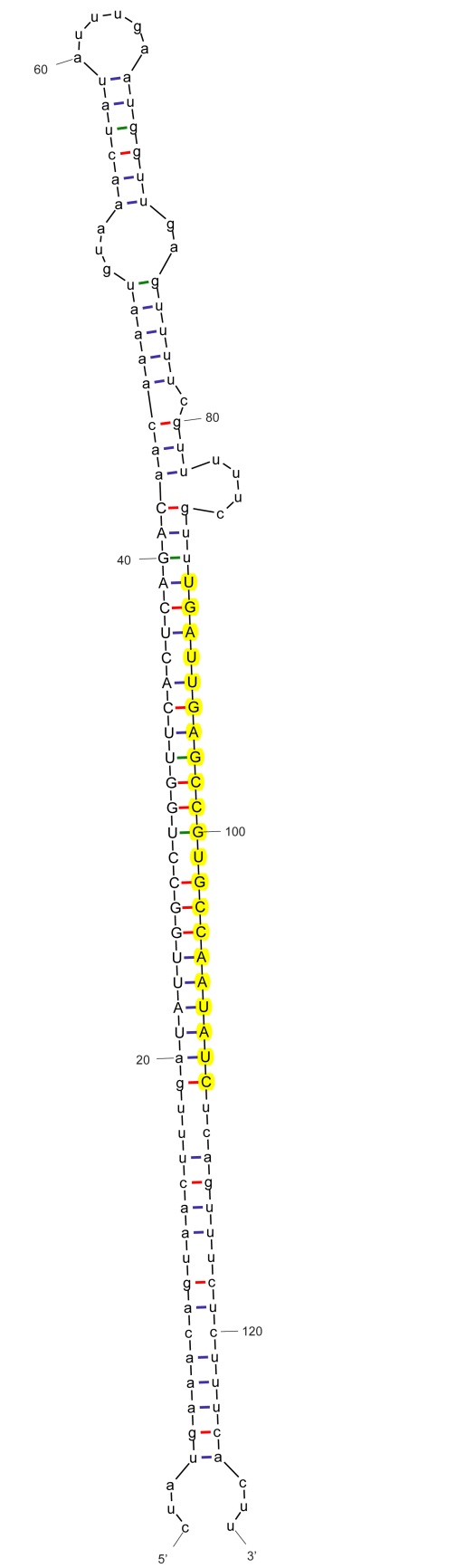


sha-miR171c-3p_stu


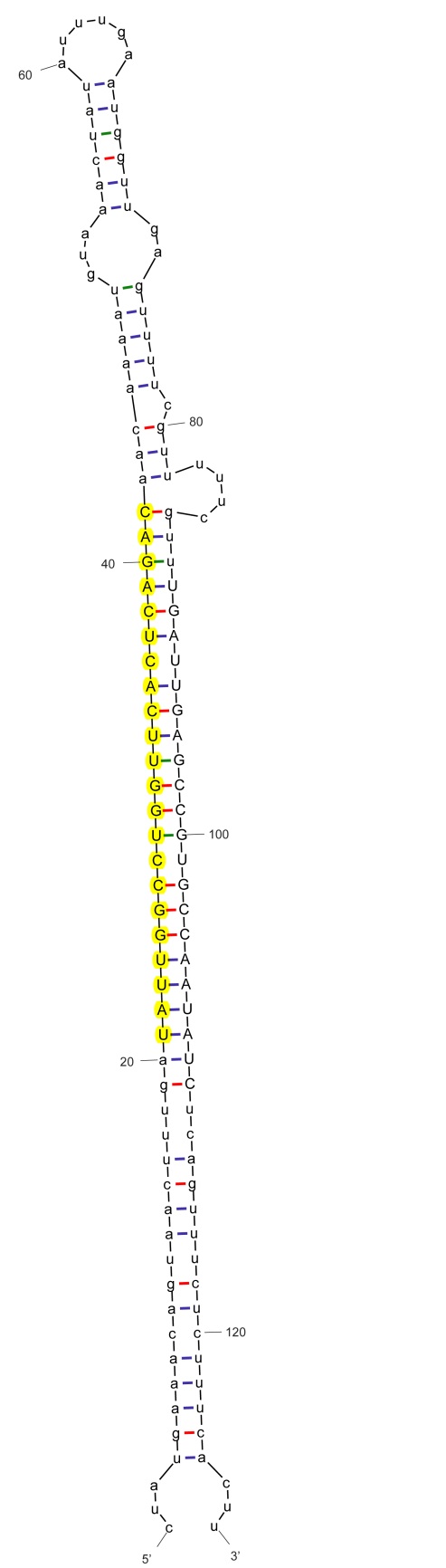


sha-miR171c-5p_stu


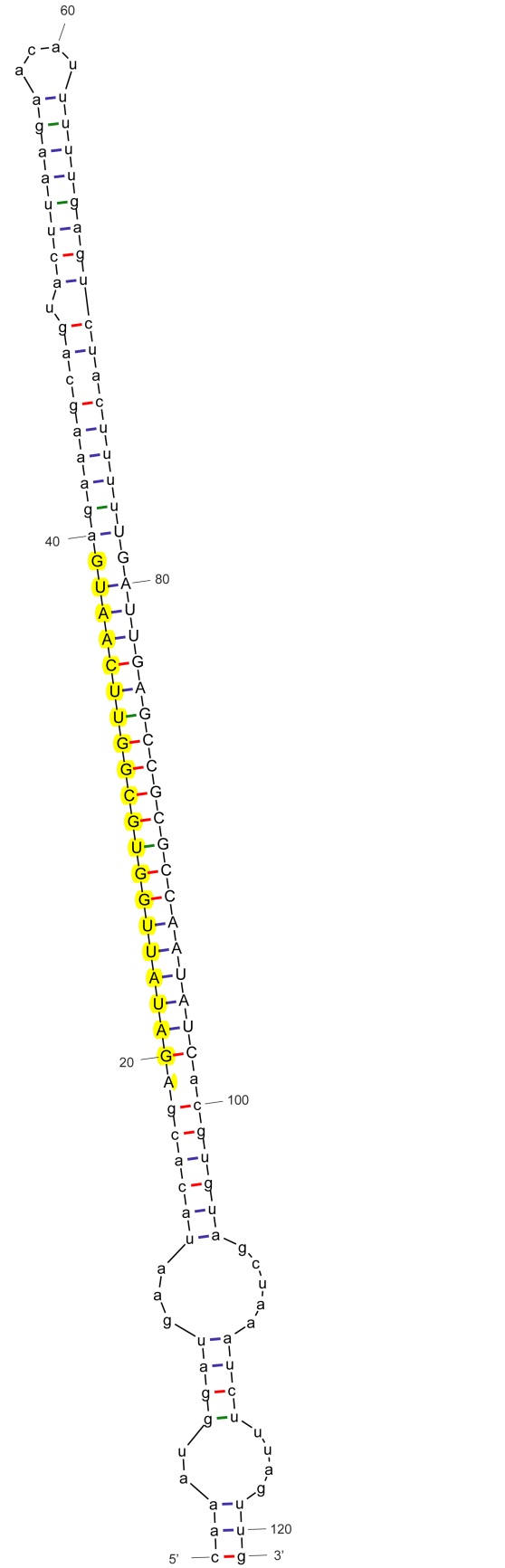


sha-miR171c-p5


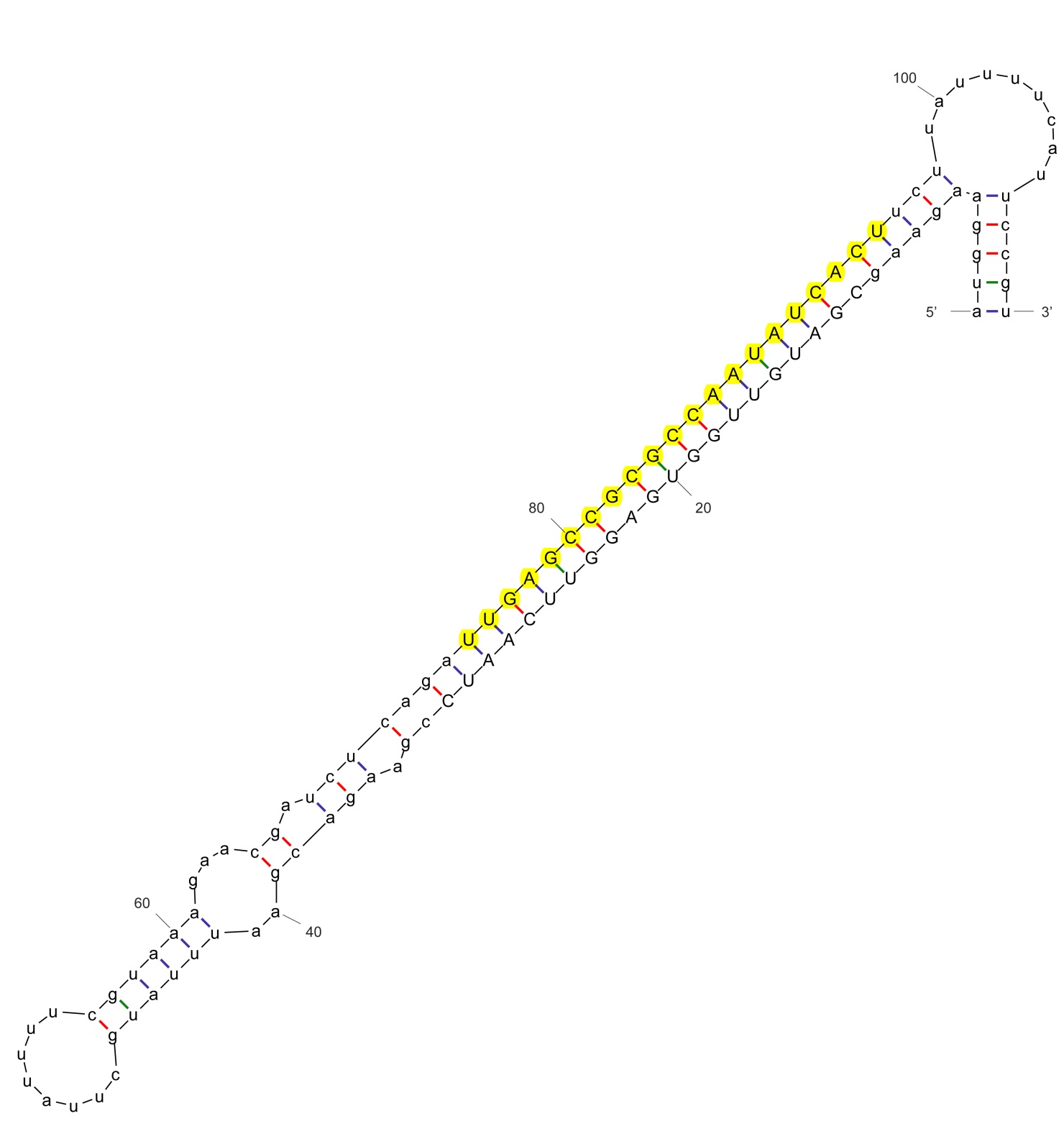


sha-miR171d


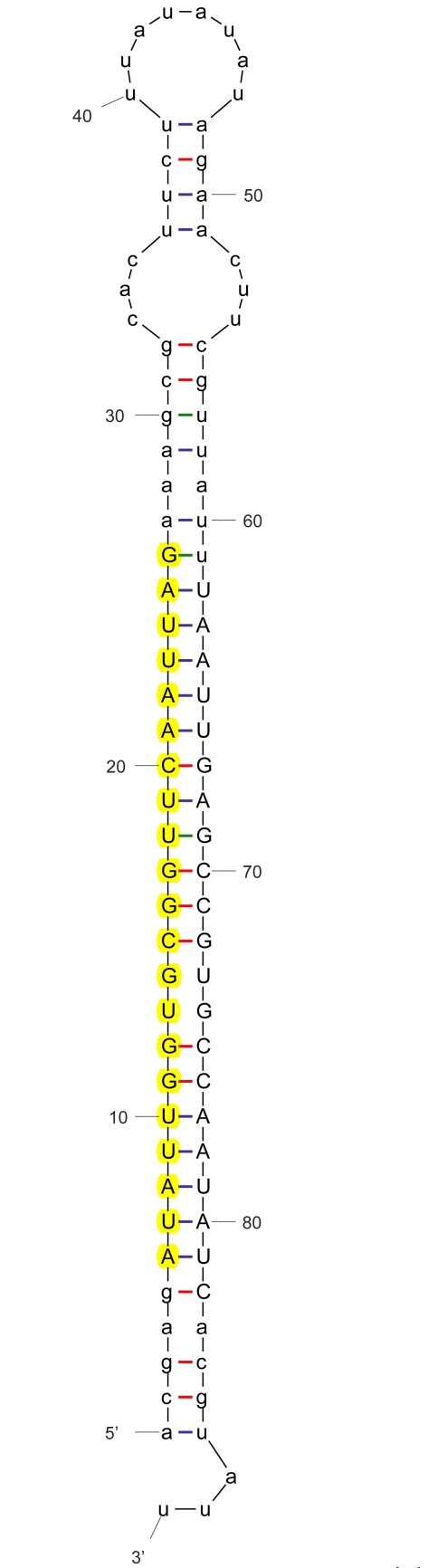


sha-miR171d-5p_stu


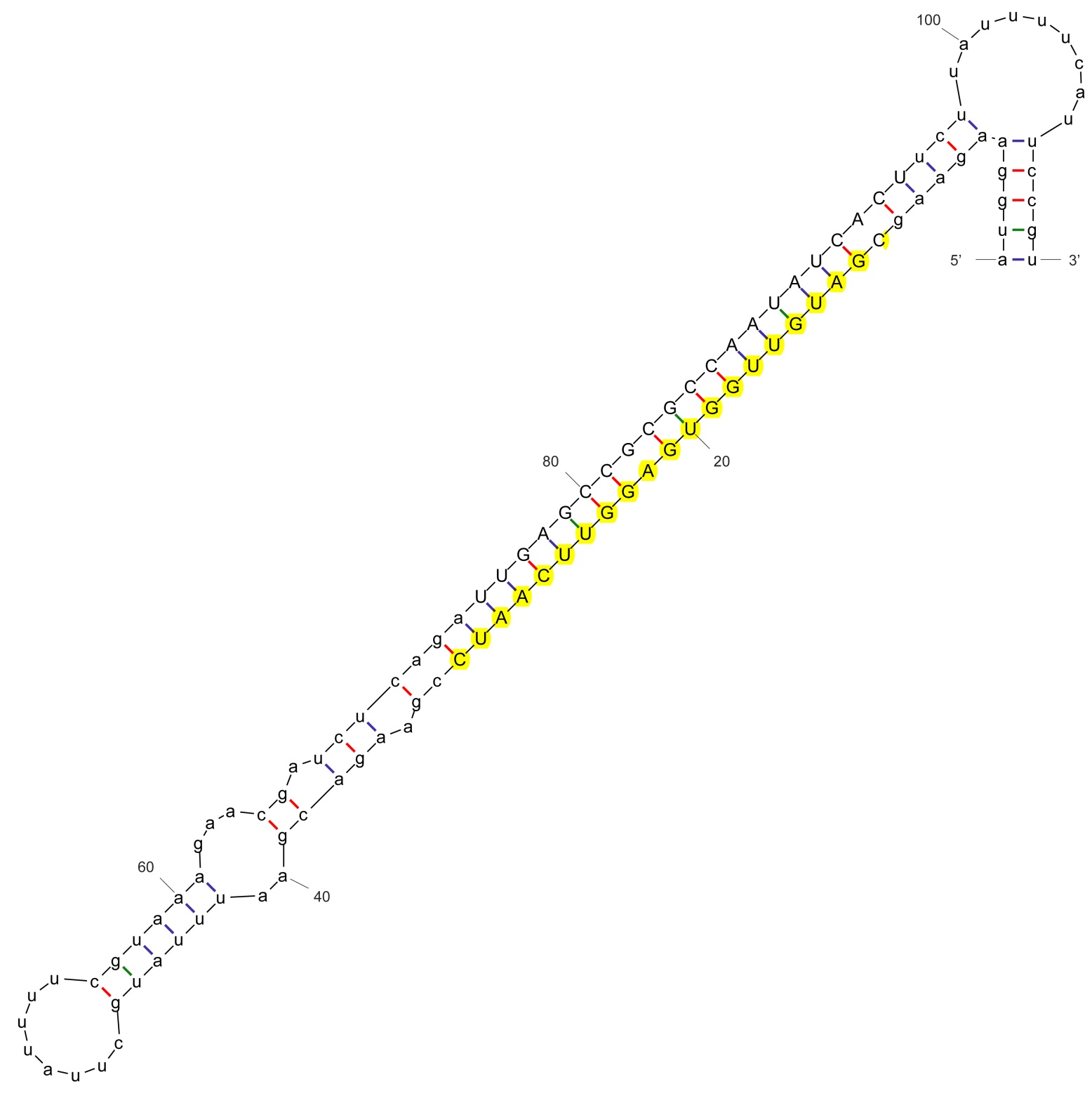


sha-miR171e-5p_mtr


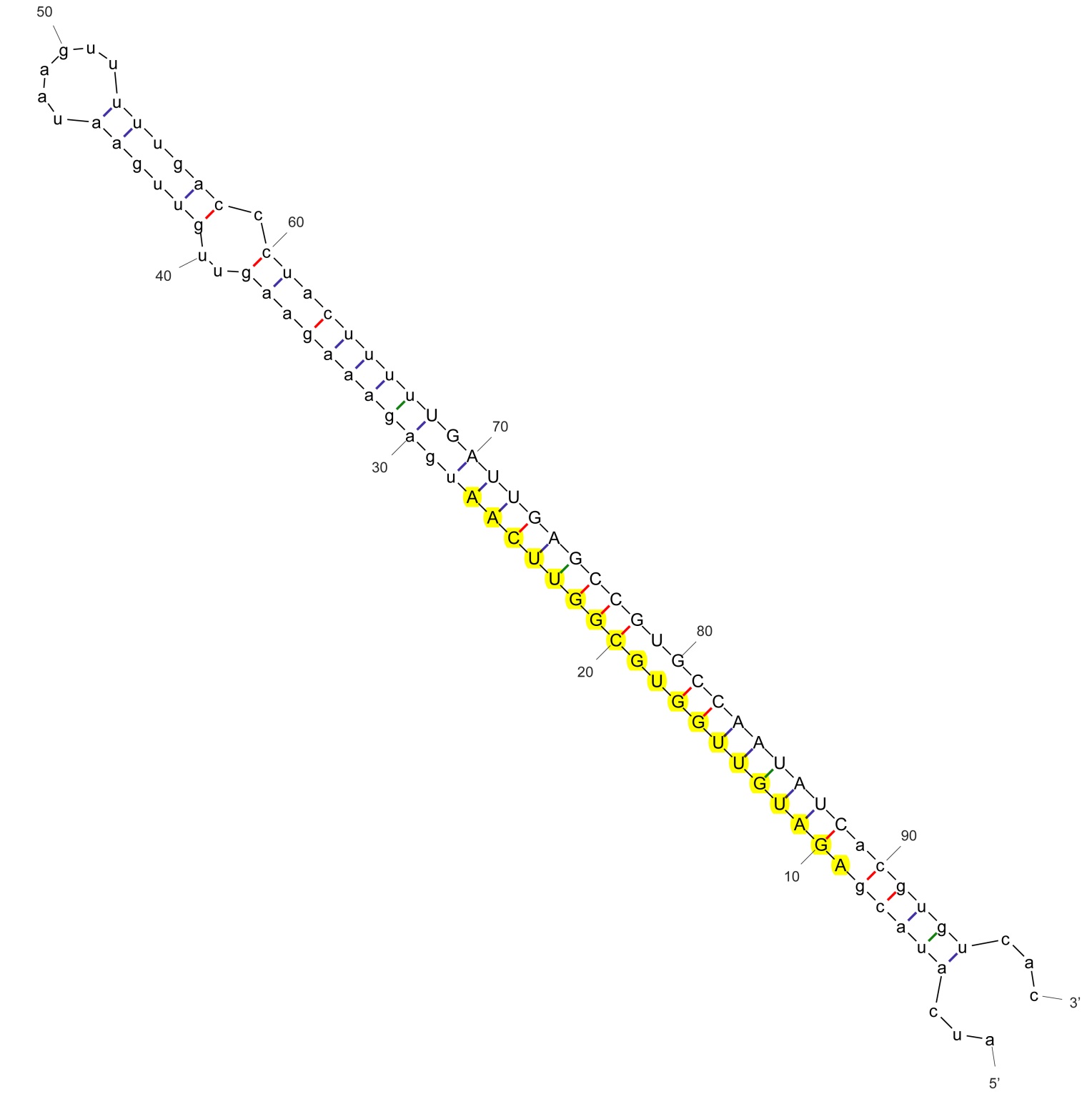


sha-miR171h-p5_vvi


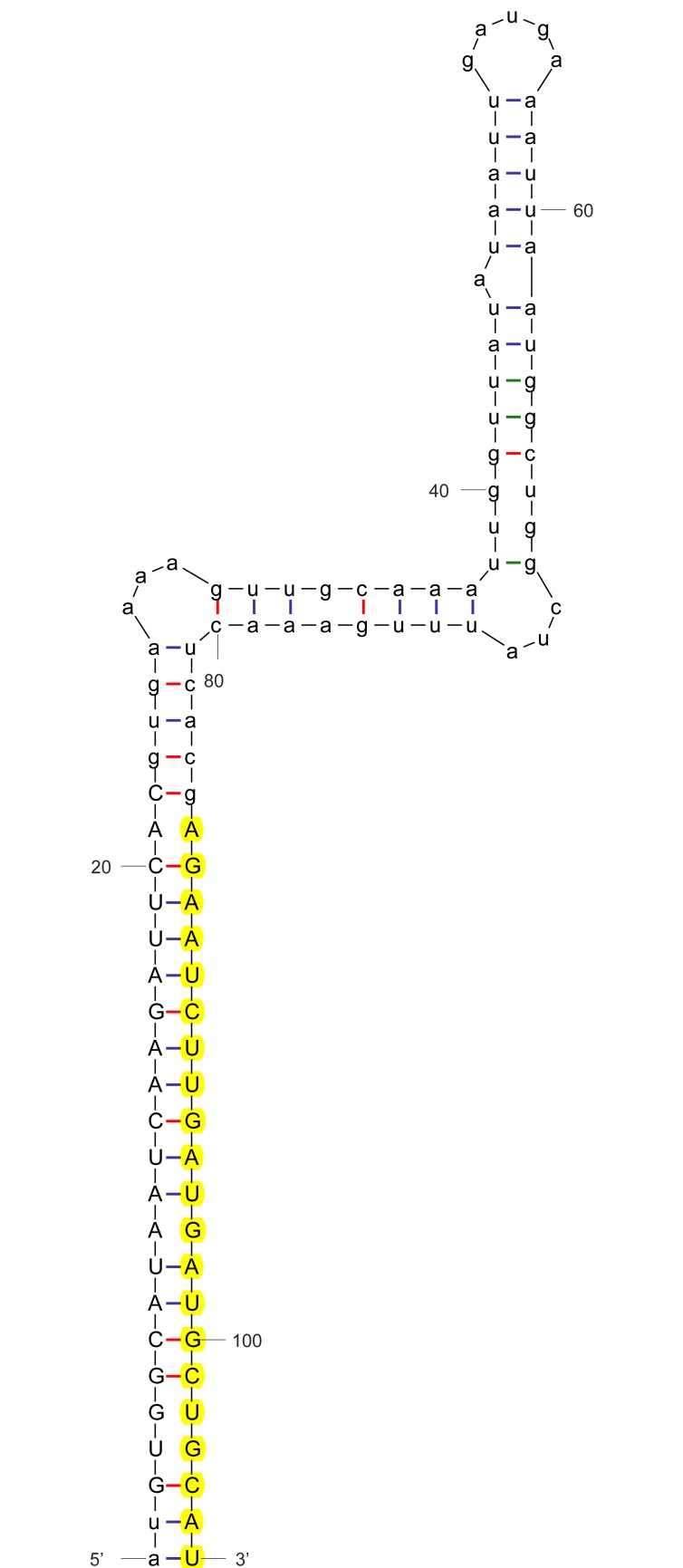


sha-miR172a


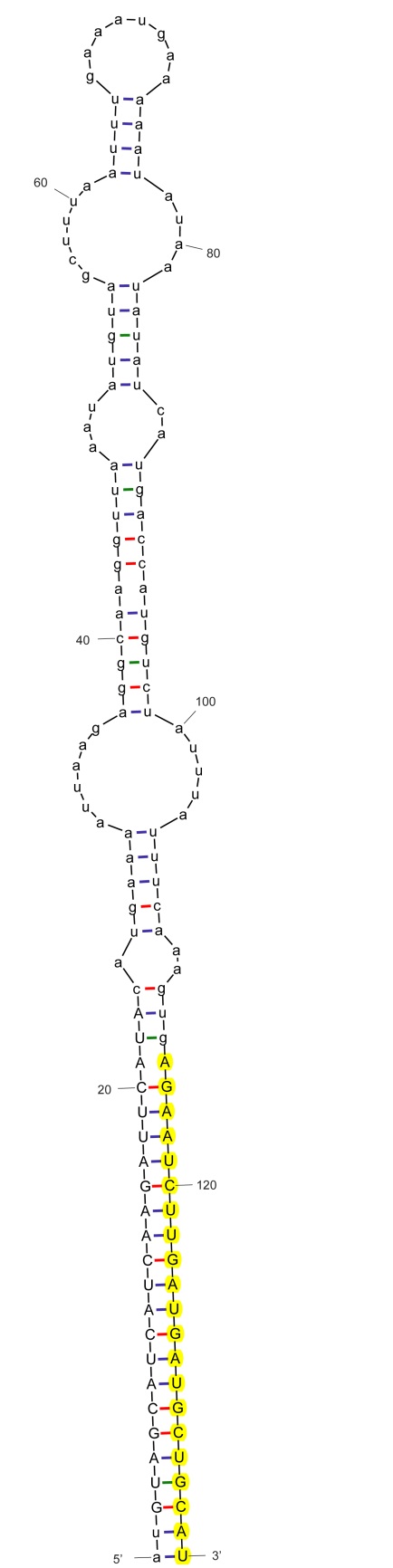


sha-miR172b


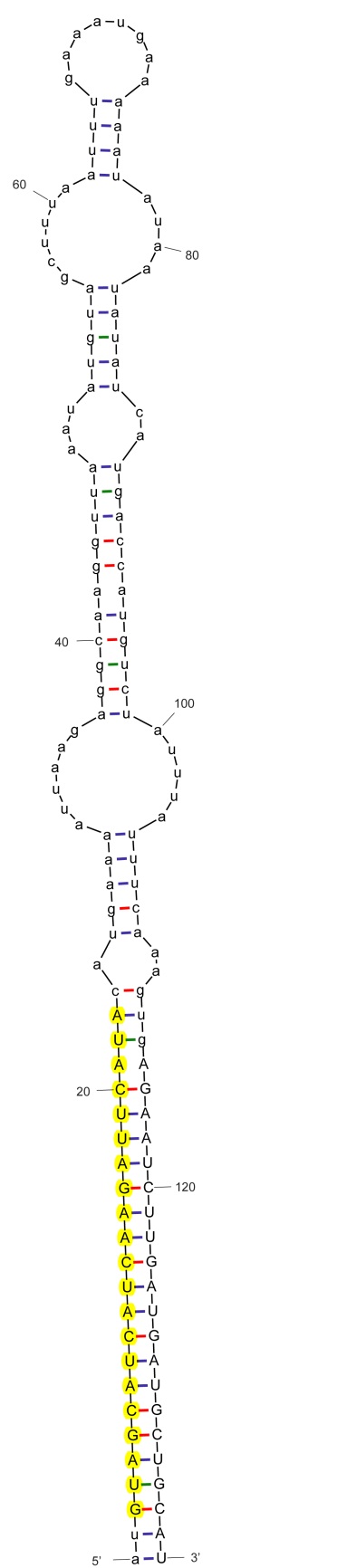


sha-miR172b-p5


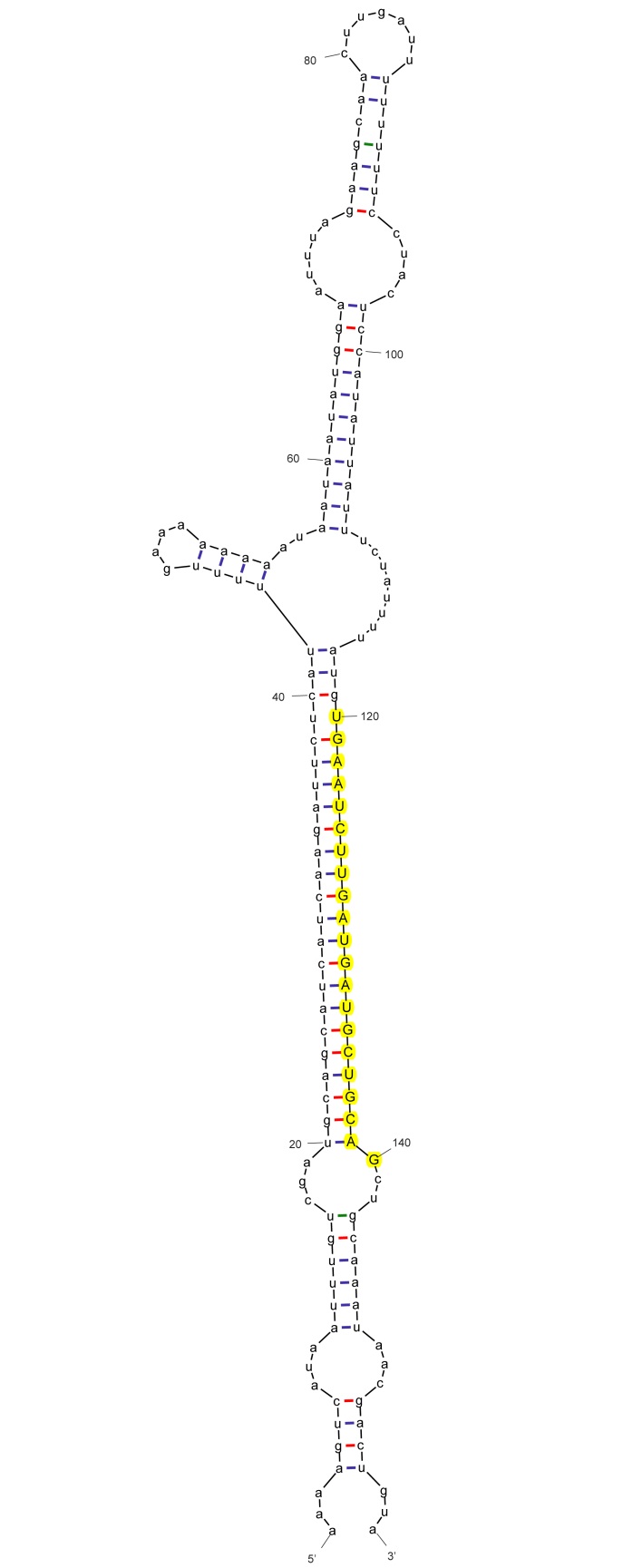


sha-miR172c-3p_aly


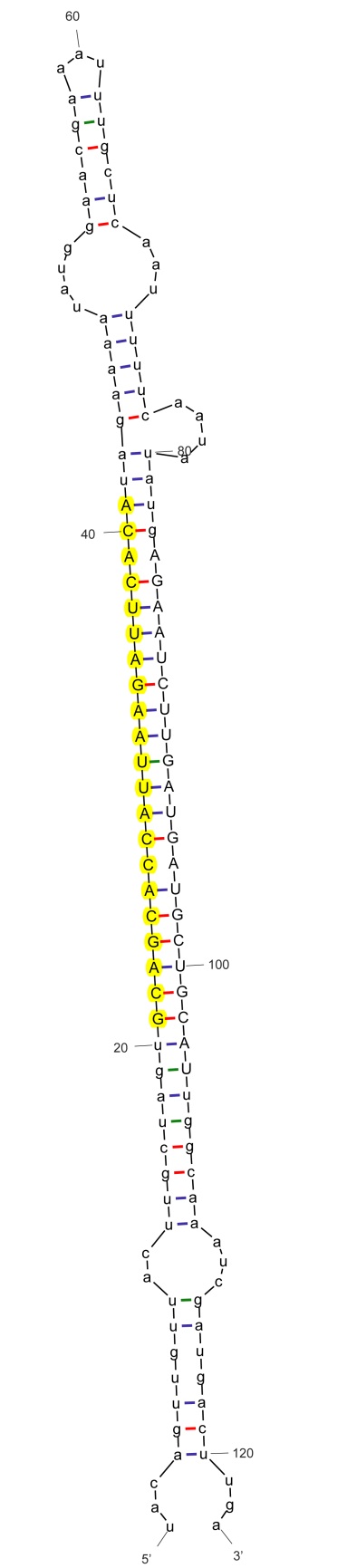


sha-miR172e-5p_aly


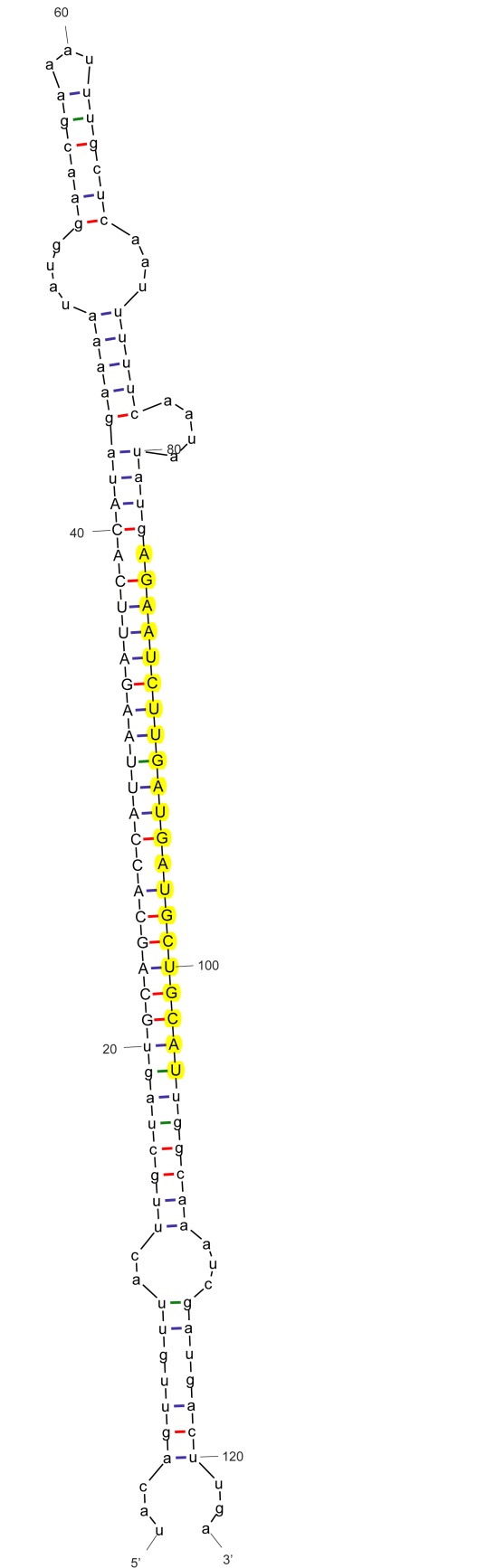


sha-miR172i_nta


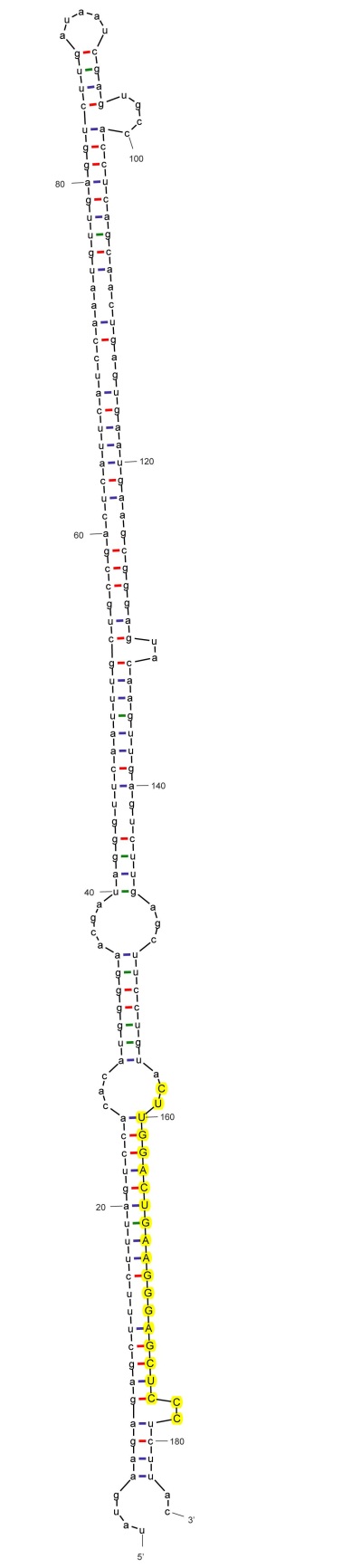


sha-miR319


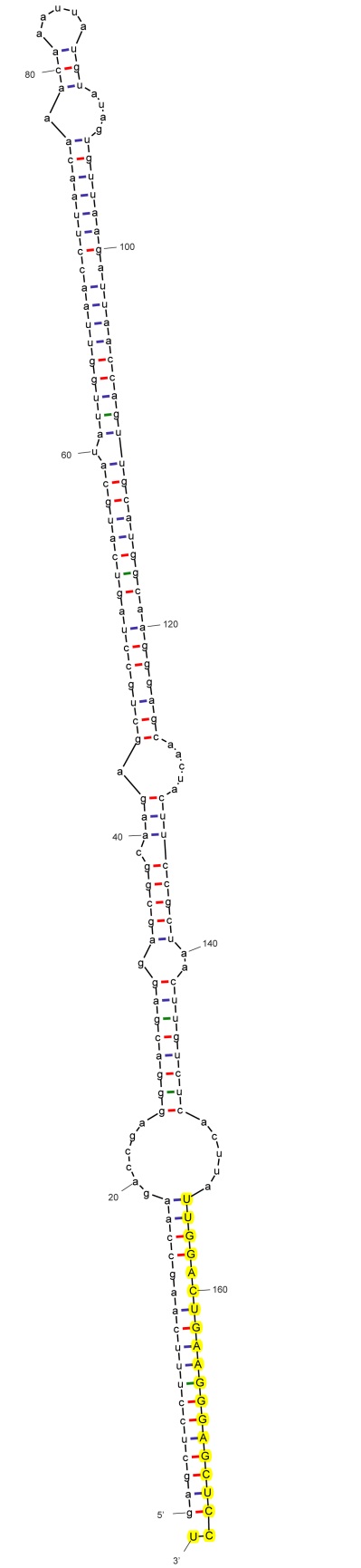


sha-miR319b_stu


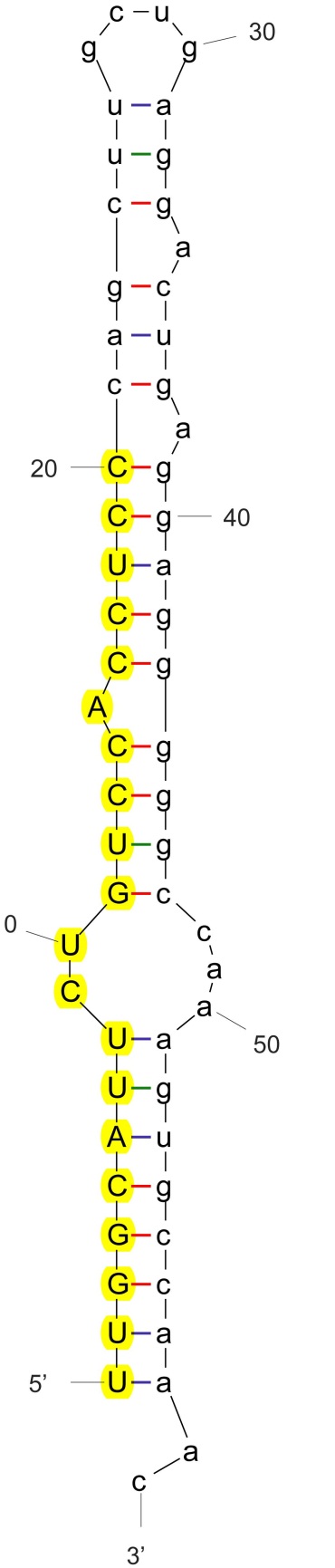


sha-miR384-5p_stu


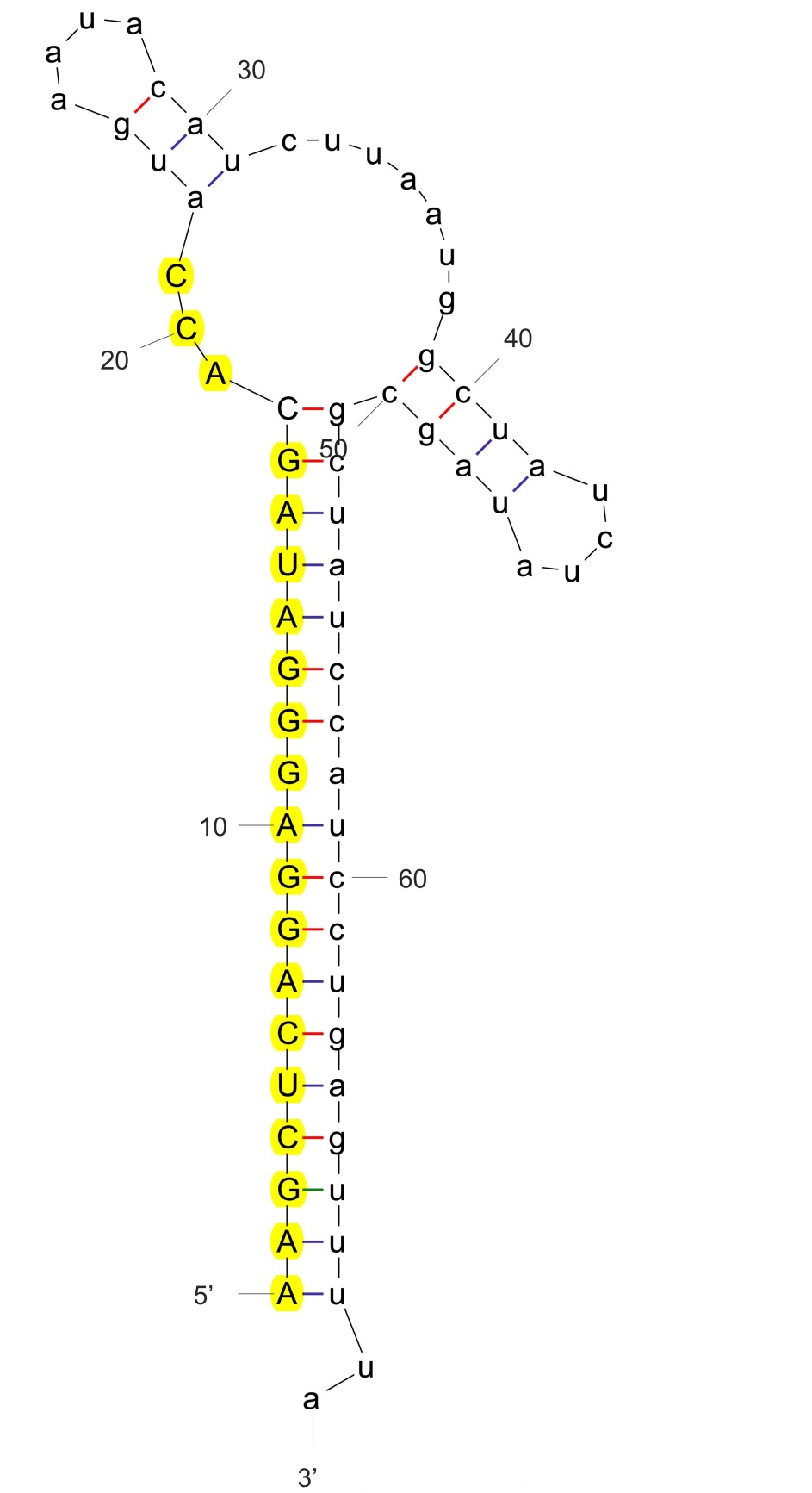


sha-miR390-5p_stus


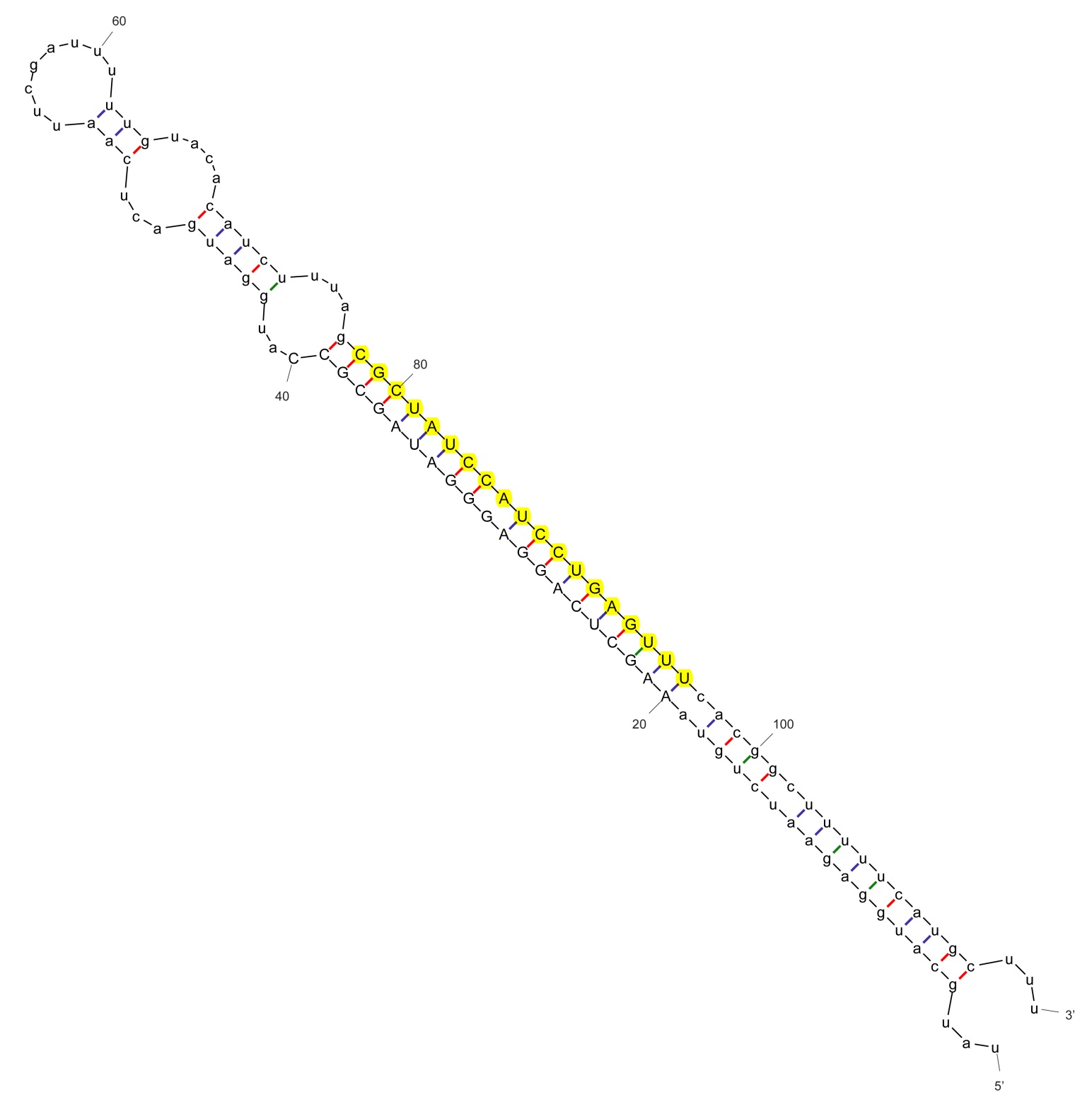


sha-miR390b-3p_aly


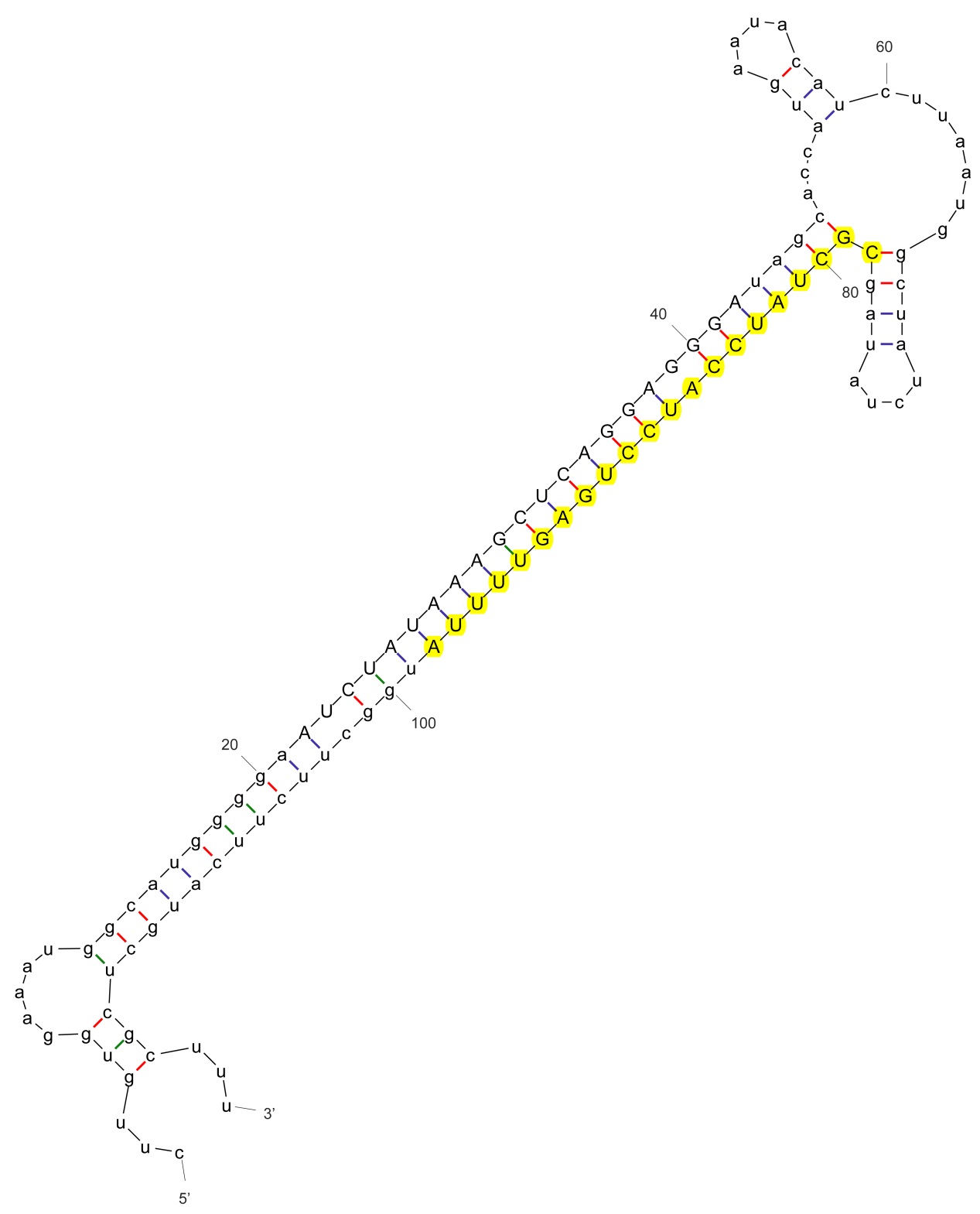


sha-miR390b-3p_aly


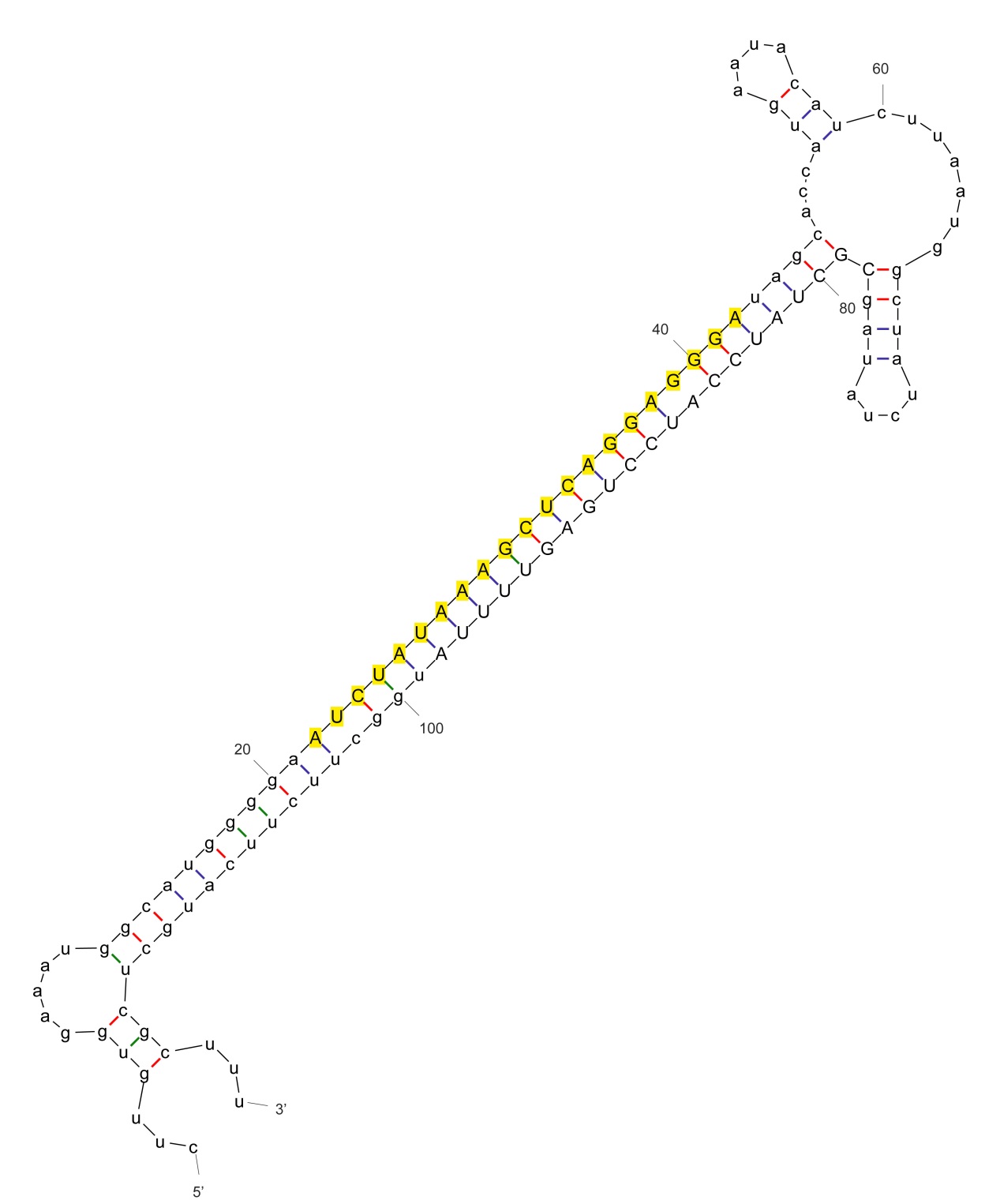


sha-miR390b-p5_nta

sha-miR390c_nta


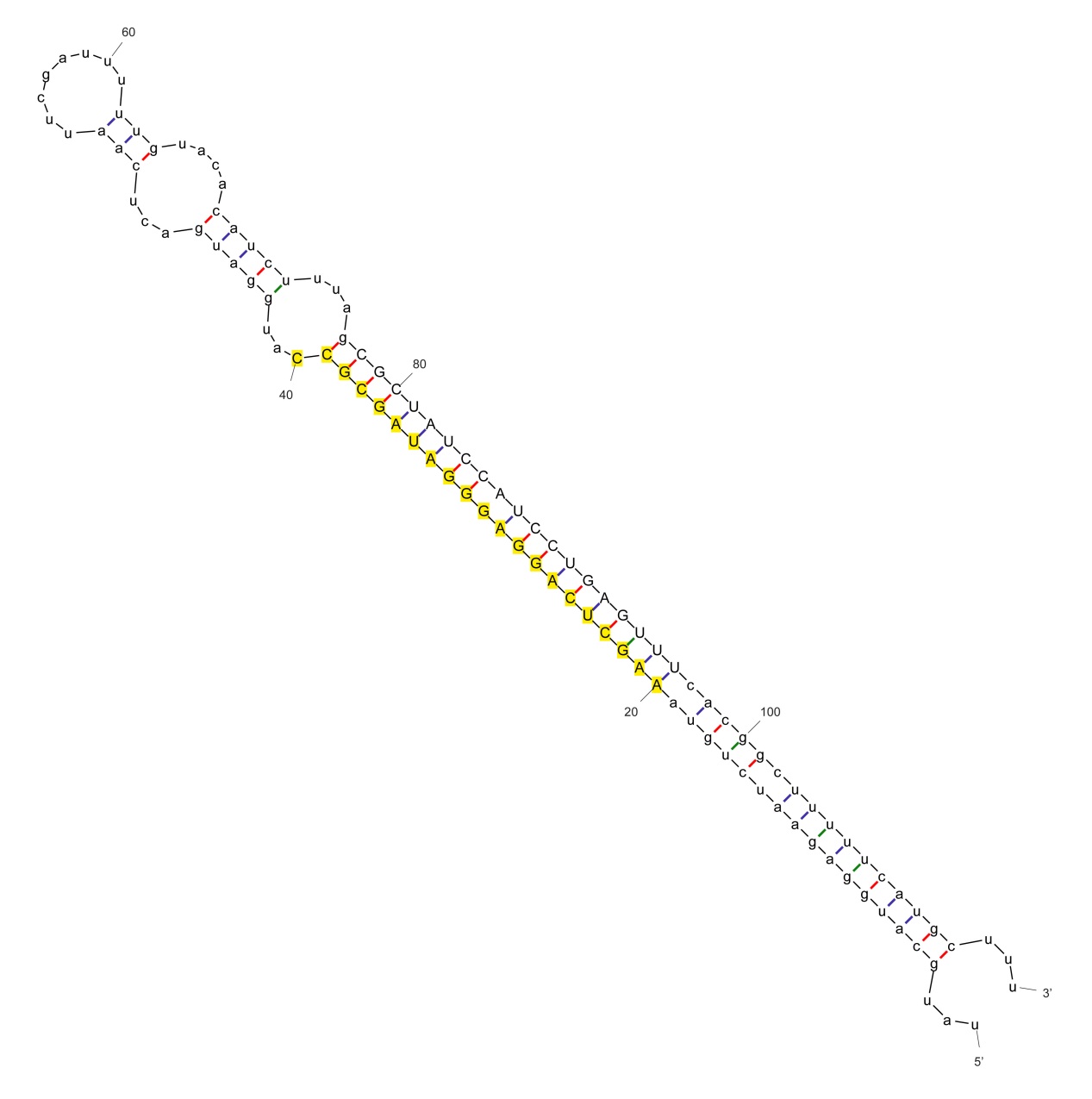

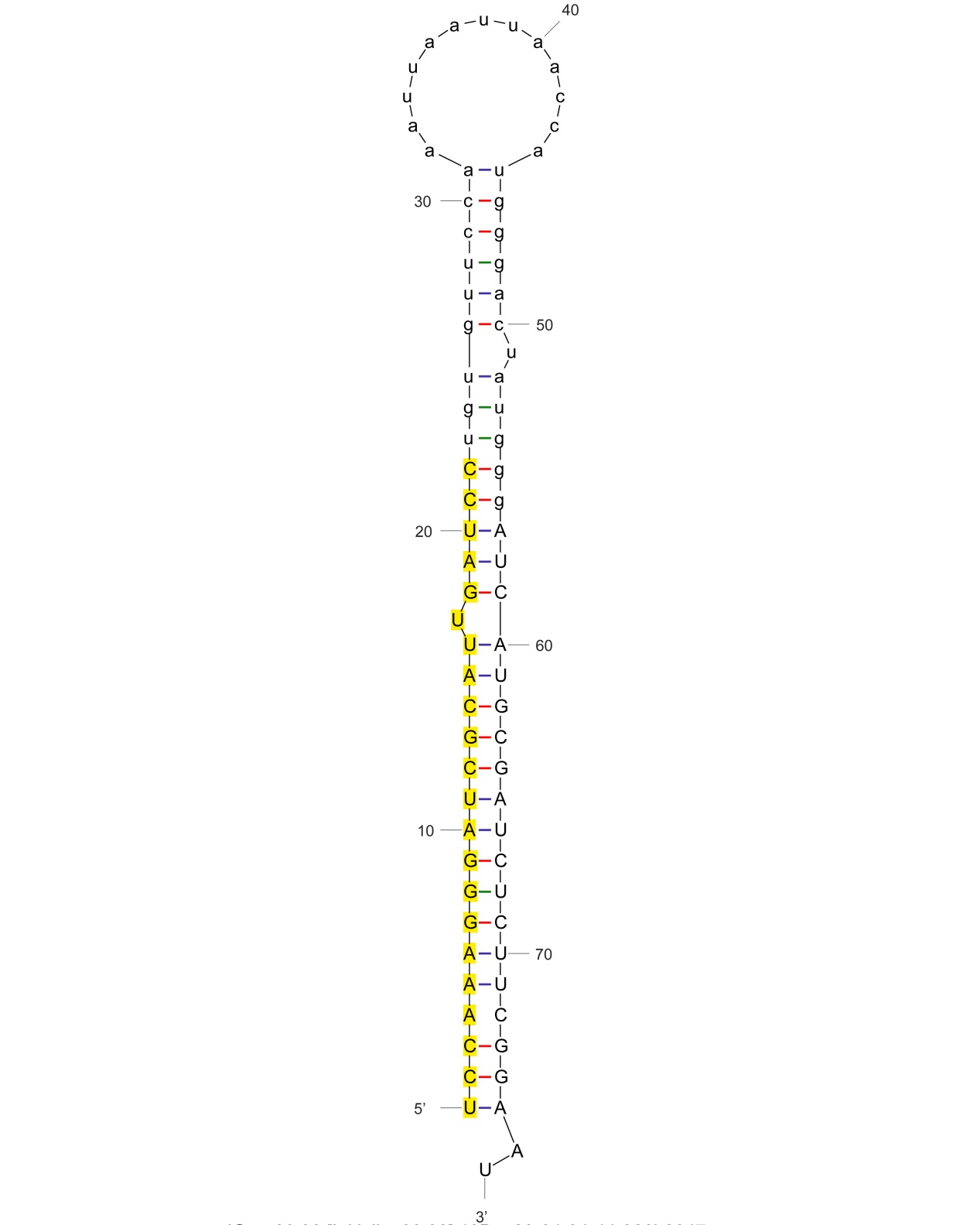


sha-miR393-5p_stu


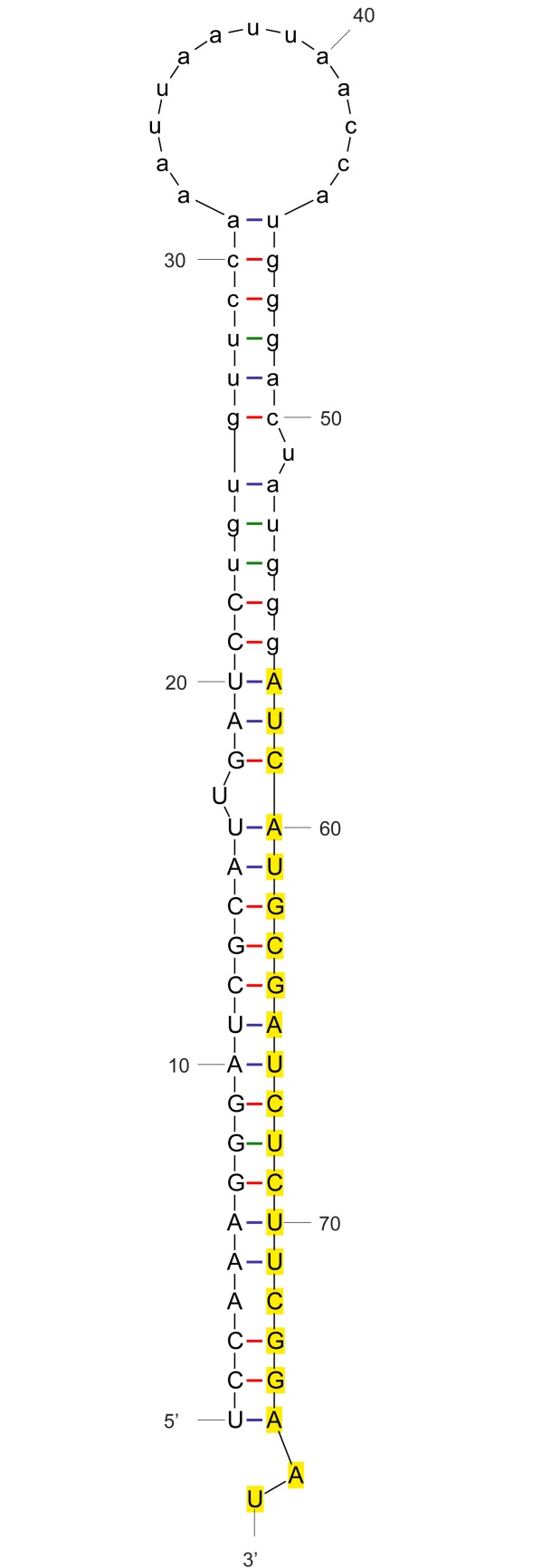


sha-miR393-3p_stu


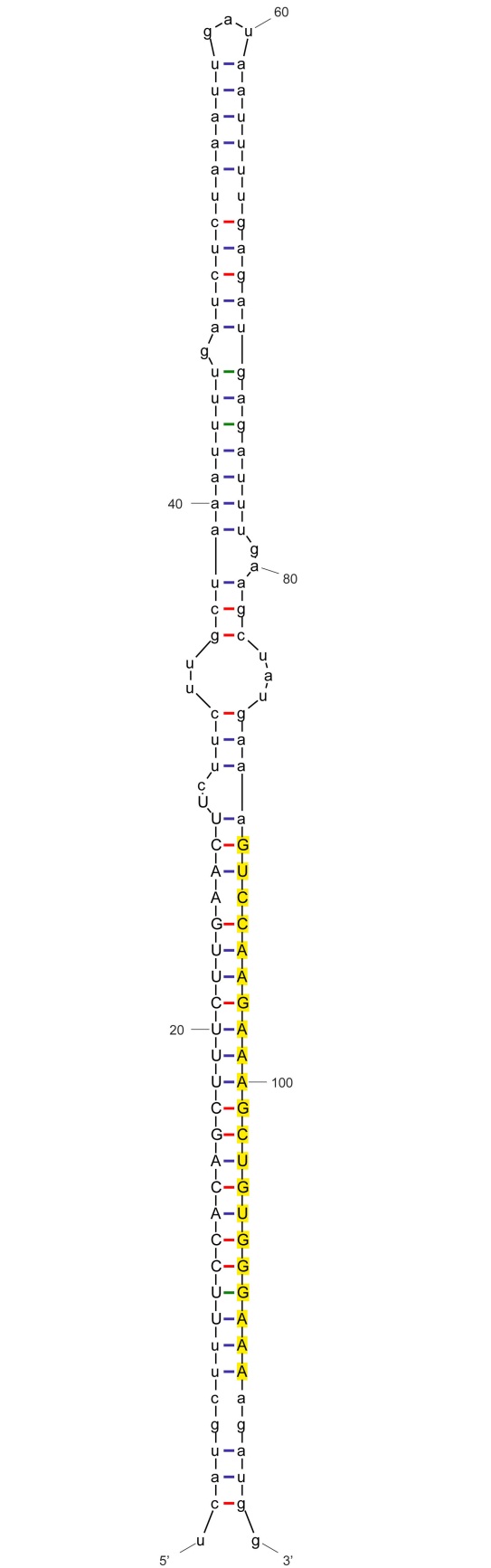


sha-miR396-3p_stu


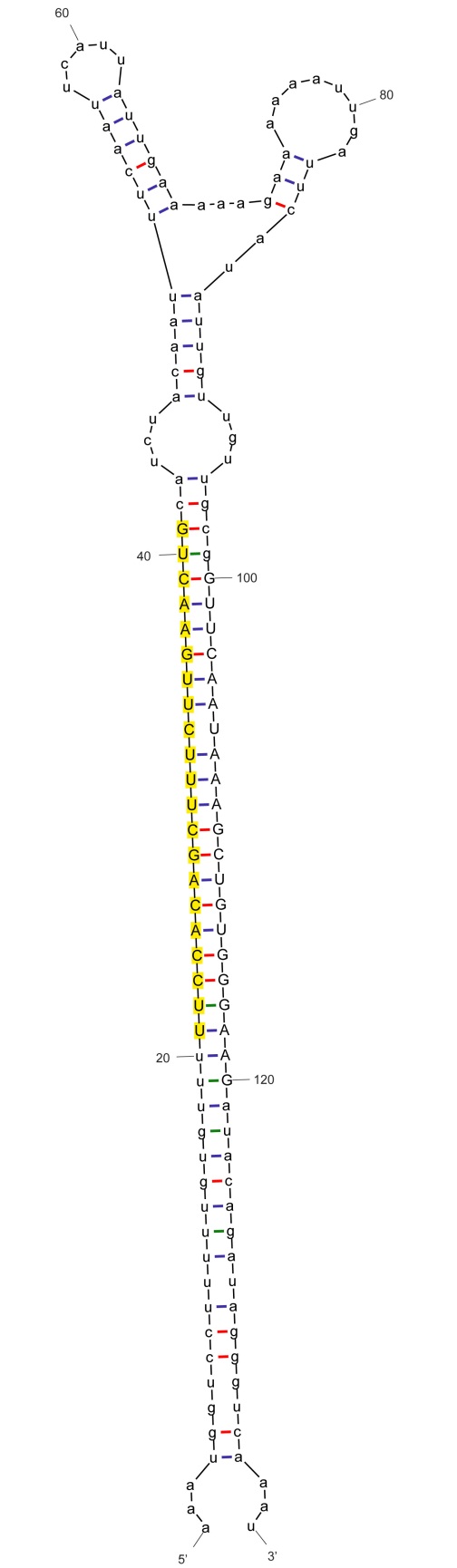


sha-miR396a_nta


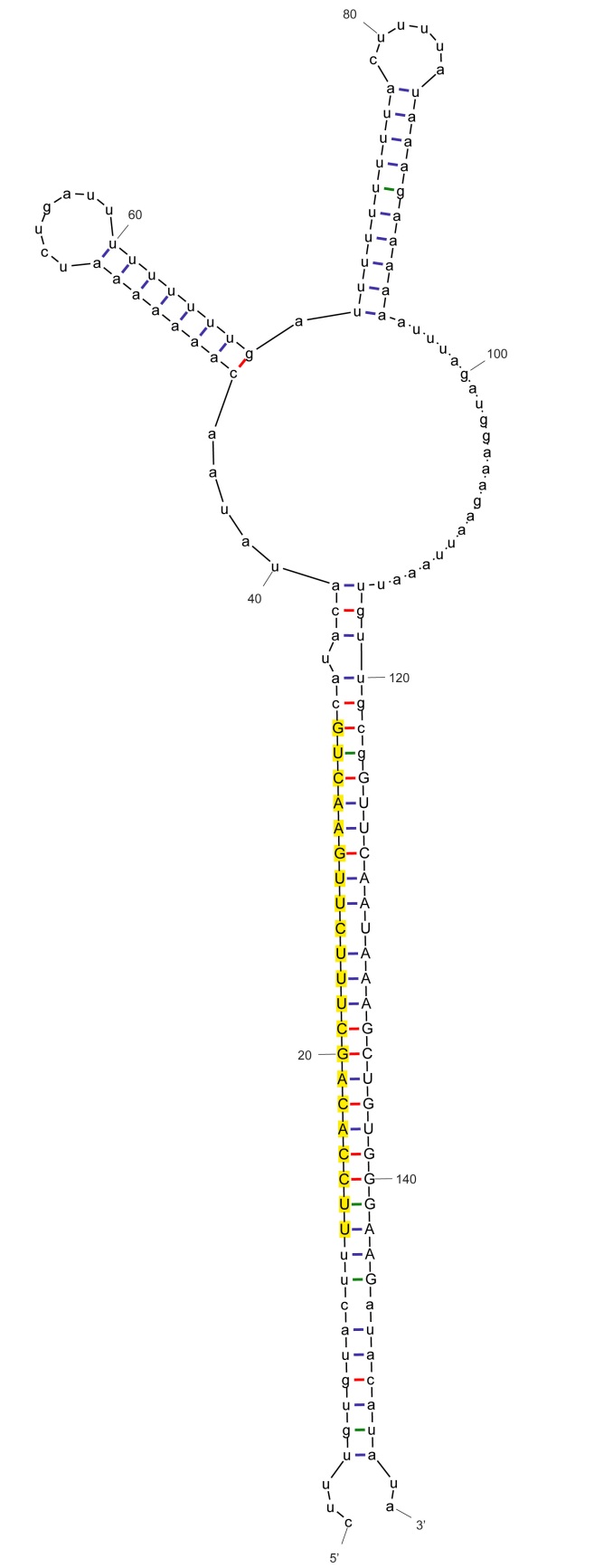


sha-miR396a_nta


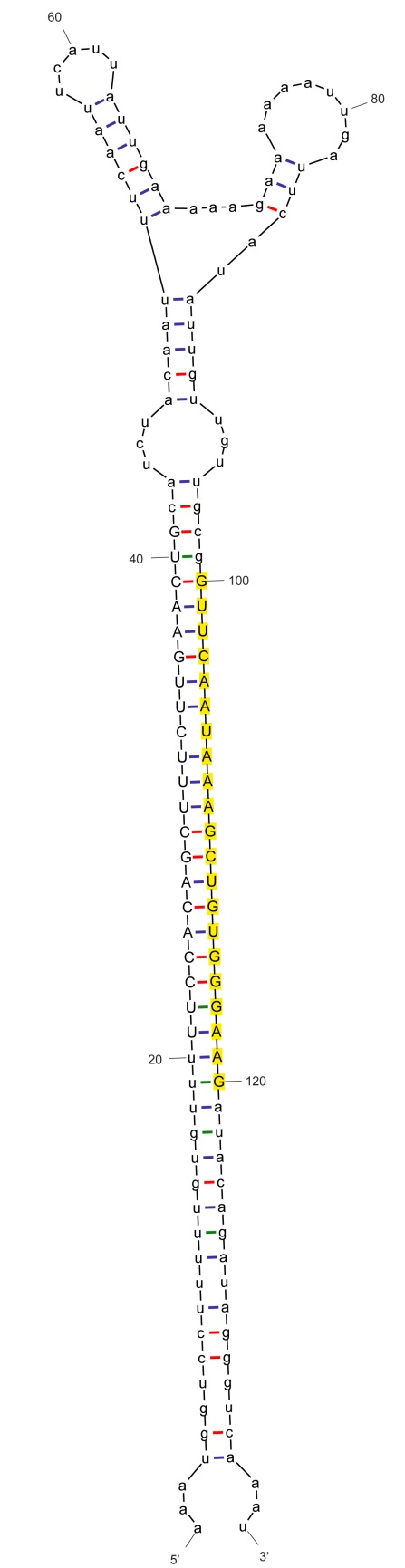


sha-miR396a-3p_aly


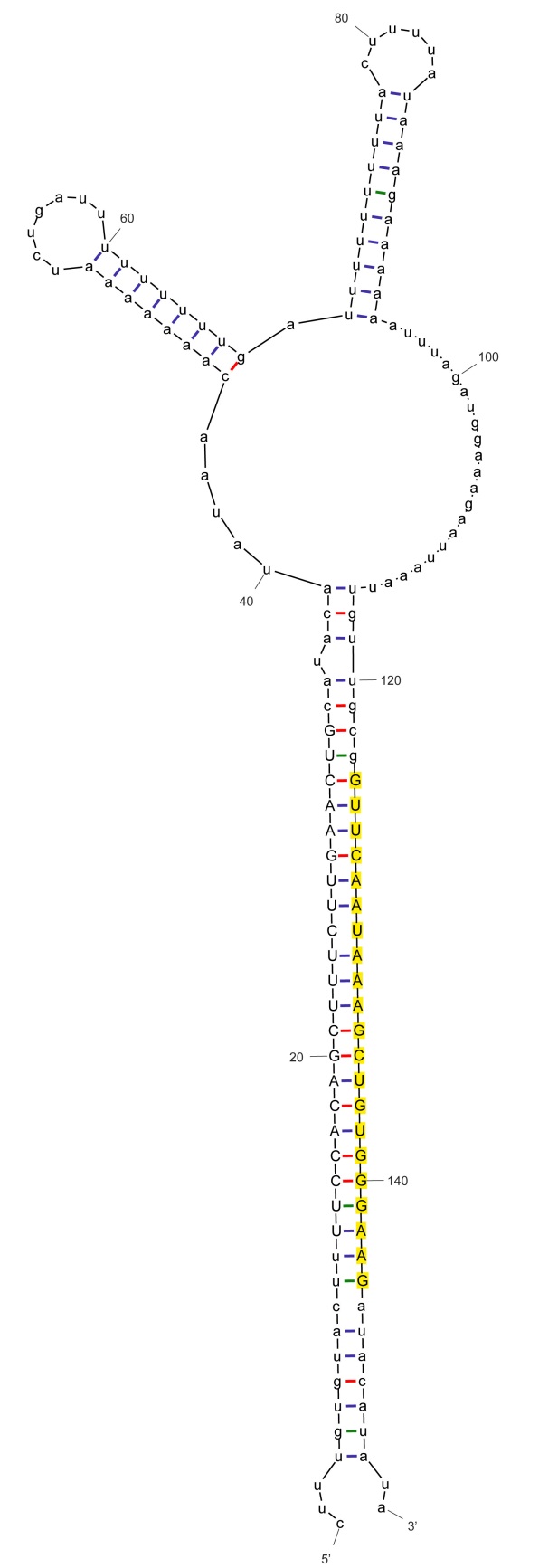


sha-miR396a-3p_aly


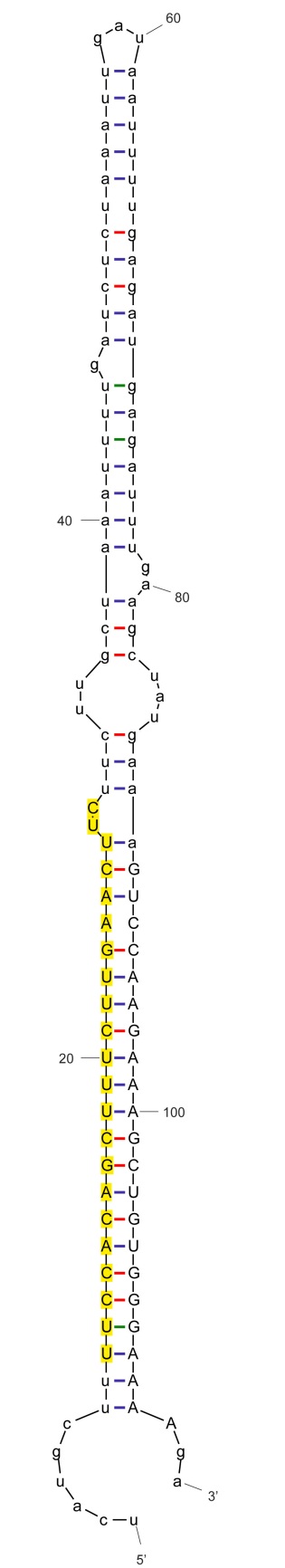


sha-miR396b_nta


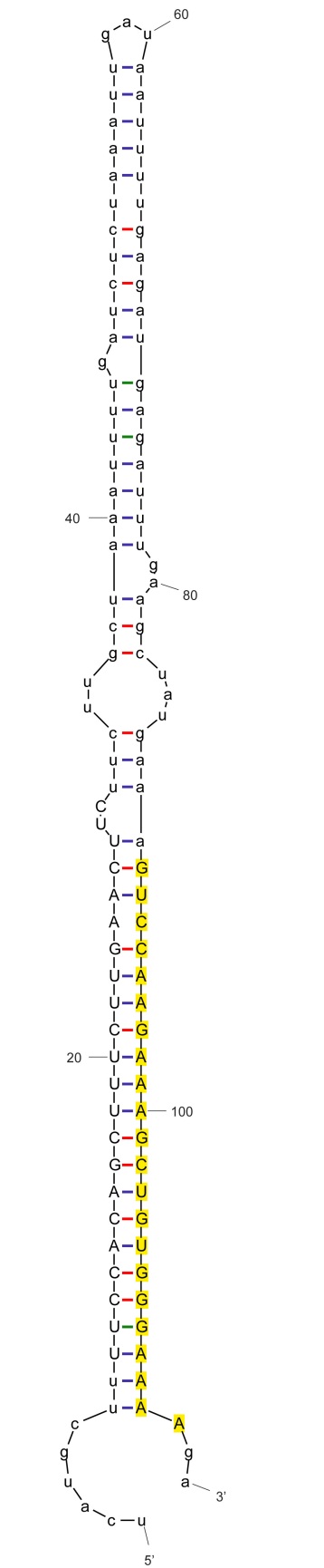


sha-miR396b-3p_aly


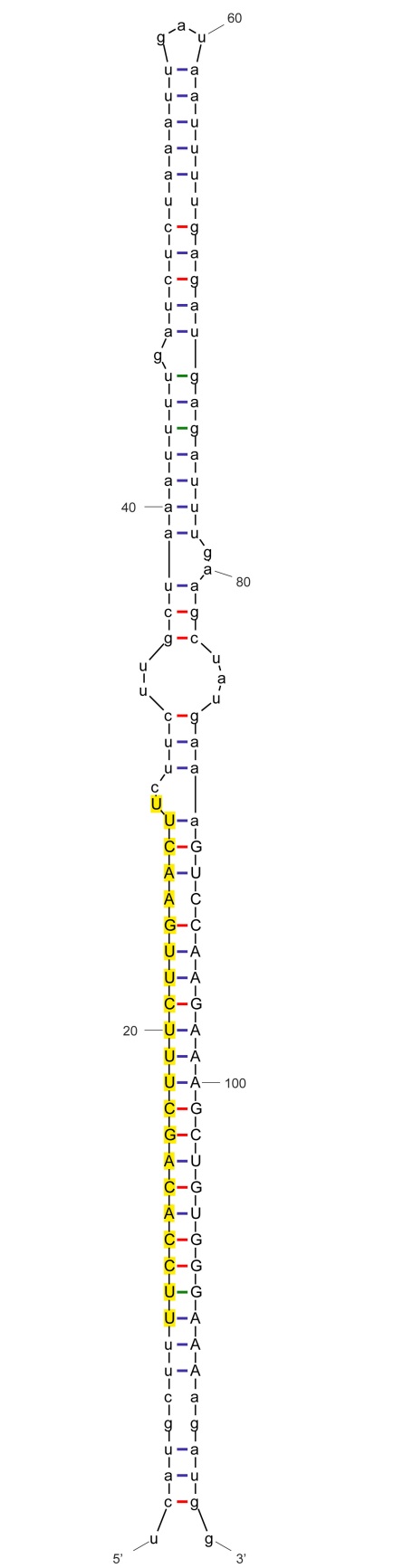


sha-miR396c_nta


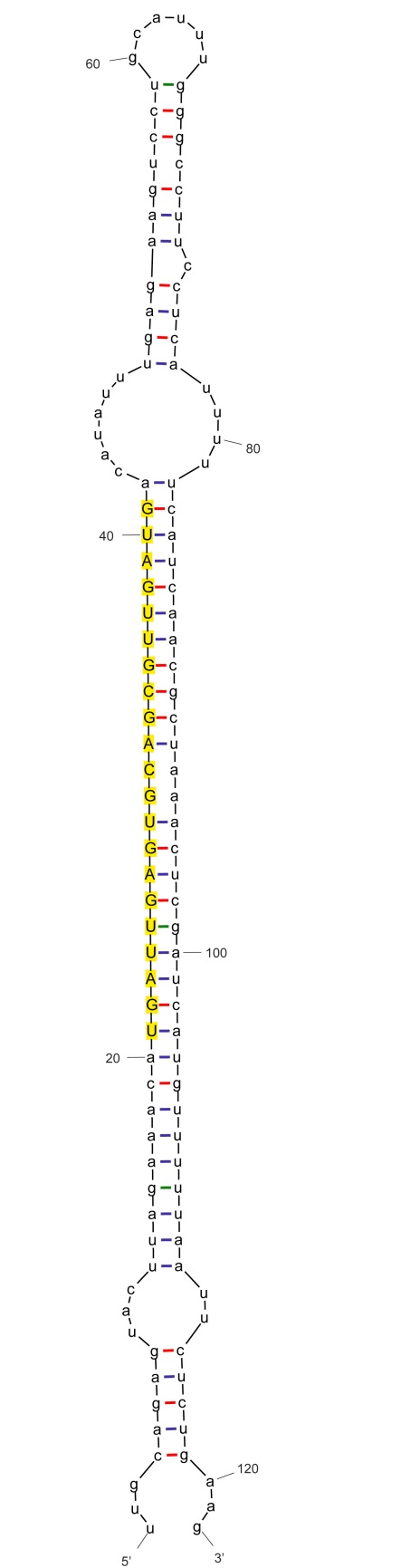


sha-miR397_nta


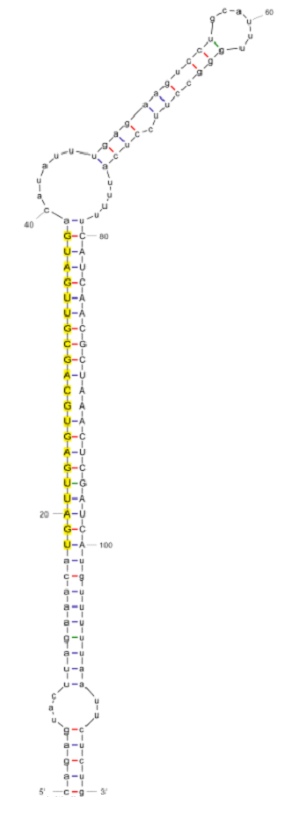


sha-miR397-p5


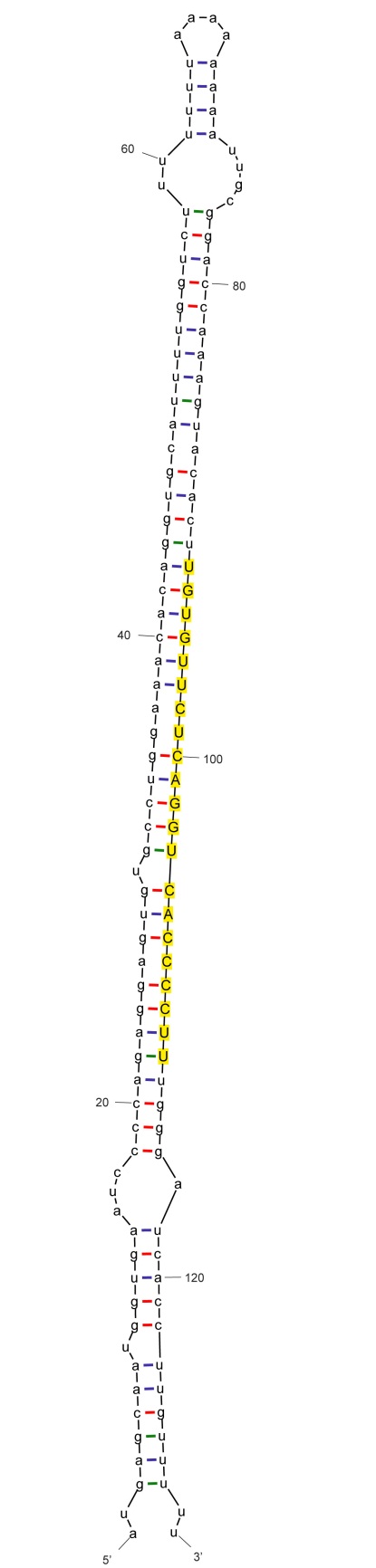


sha-miR398_nta


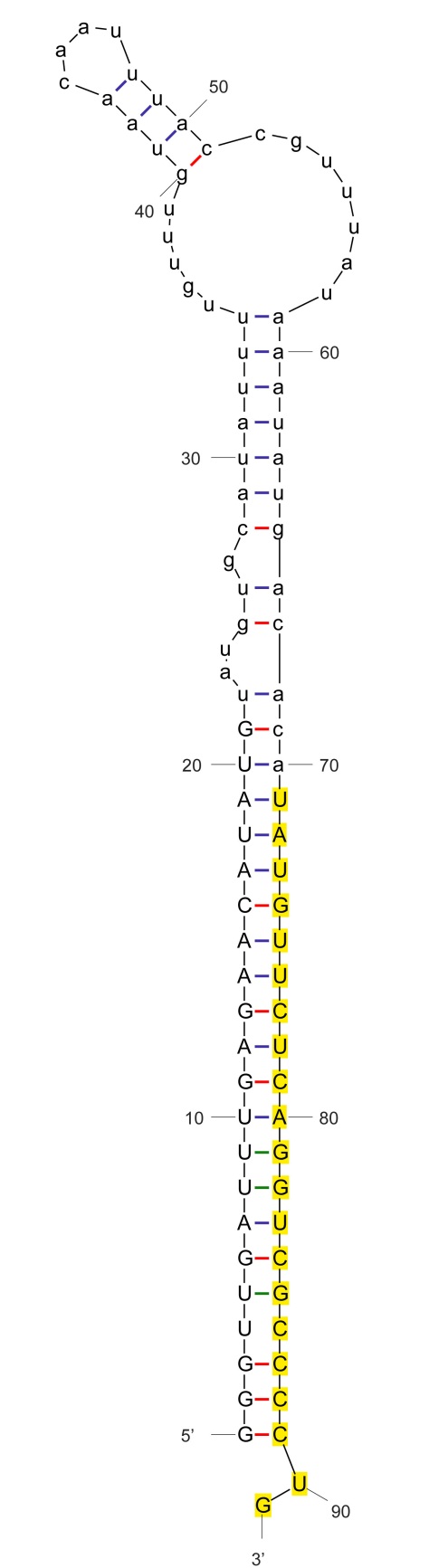


sha-miR398a-3p_stu


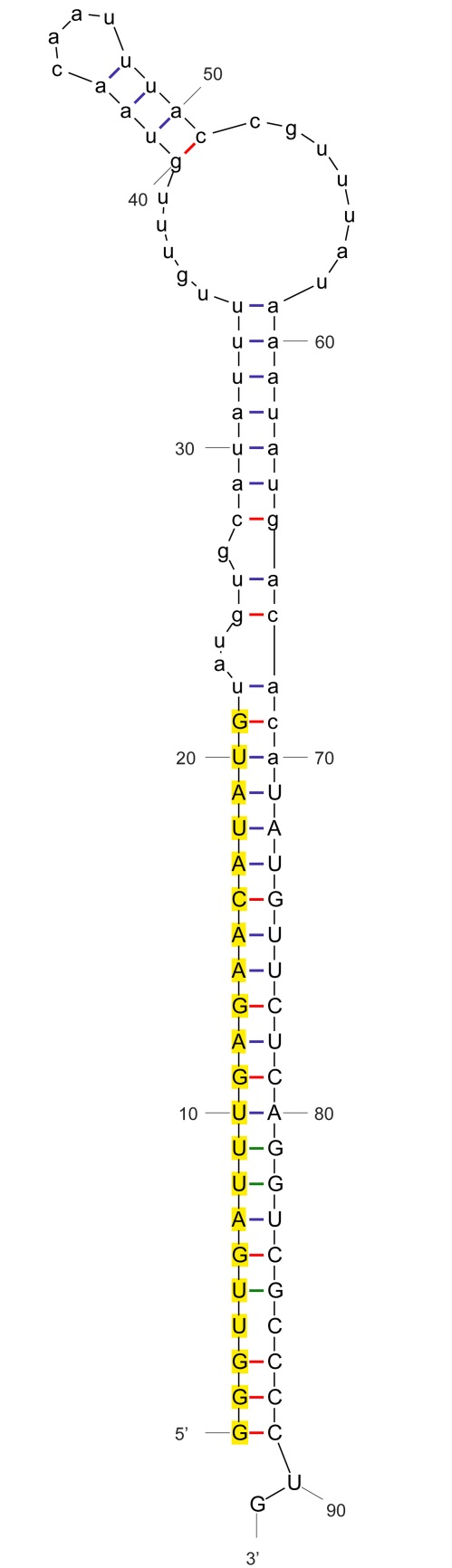


sha-miR398a-5p_stu


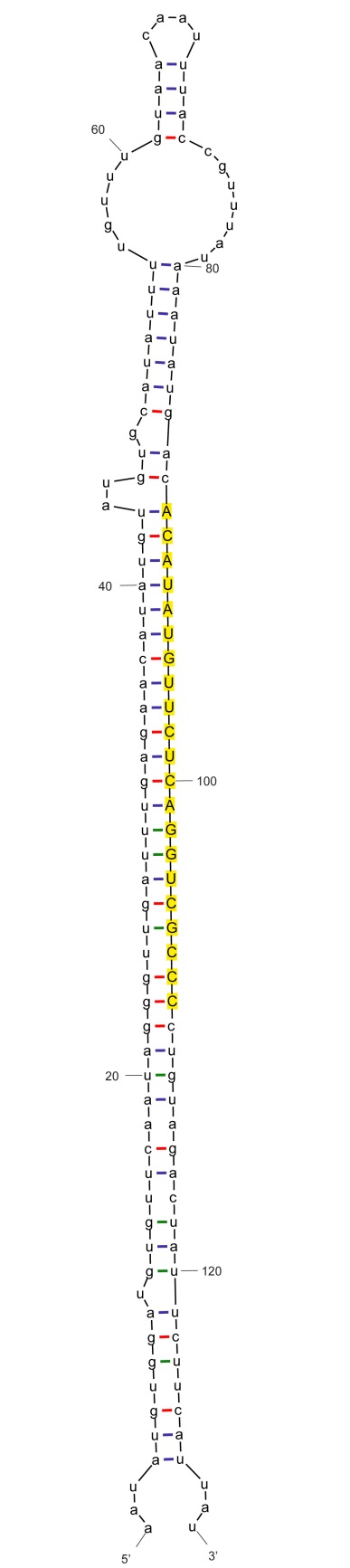


sha-miR398a-p3_cme

sha-miR399

sha-miR399-p5

sha-miR399b_nta

sha-miR399b-p5_nta

sha-miR399g_nta

sha-miR399n-5p_stu

sha-miR408_nta

sha-miR408a-3p_stu

sha-miR408a-5p_stu

sha-miR408b-5p_stu

sha-miR482a

sha-miR482a_ghr

sha-miR482a-5p_stu

sha-miR482b

sha-miR482c_stu

sha-miR482c-p5_stu

sha-miR482d-5p_stu

sha-miR530_stu

sha-miR530a_cme

sha-miR827_nta

sha-miR827-5p_stu

sha-miR1886a-p3_stu

sha-miR1886a-p5_stu

sha-miR1916-p3

sha-miR1916-p5

sha-miR1917-p3

sha-miR1917-p5

sha-miR1919a

sha-miR1919a-p5

sha-miR1919b

sha-miR1919b-p5

sha-miR1919c

sha-miR1919c-p5

sha-miR3627-3p_stu

sha-miR3627-5p_stu

sha-miR4376

sha-miR4376-p3

sha-miR5300

sha-miR5300-p3

sha-miR5301

sha-miR5301-p3

sha-miR6022

sha-miR6022-p5

sha-miR3627-5p_stu

sha-miR3627-5p_stu

sha-miR6023-p3

sha-miR3627-5p_stu

sha-miR3627-5p_stu

sha-miR6022-p5

sha-miR6023-p5

sha-miR6024

sha-miR6024-5p_stu

sha-miR6025_stu

sha-miR6025-p3_stu

sha-miR6026

sha-miR6026-5p_stu

sha-miR6027

sha-miR6027-p5

sha-miR7981-3p_stu

sha-miR7981-p5_stu

sha-miR7982a-p3_stus

sha-miR7982a-p5_stu

sha-miR7983-p3_stu

sha-miR7983-p5_stu

sha-miR8007a-5p_stu

sha-miR8007a-p3_stu
