# Supplementary material for: Identification of chilling stress-responsive tomato microRNAs and their target genes by high-throughput sequencing and degradome analysis
Source: BMC Genomics. 2014 Dec 17;15(1):1130. doi: 10.1186/1471-2164-15-1130 (PMC4377850; doi:10.1186/1471-2164-15-1130)
Supplement: Supplementary file 4 — Additional file 4: Figure S2: - Secondary structures of 236 putative novel miRNAs identified in the CT and NT libraries. The mature miRNAs sequences are underlined in yellow. (DOCX 19 MB) [file 12864_2014_6877_MOESM4_ESM.docx]

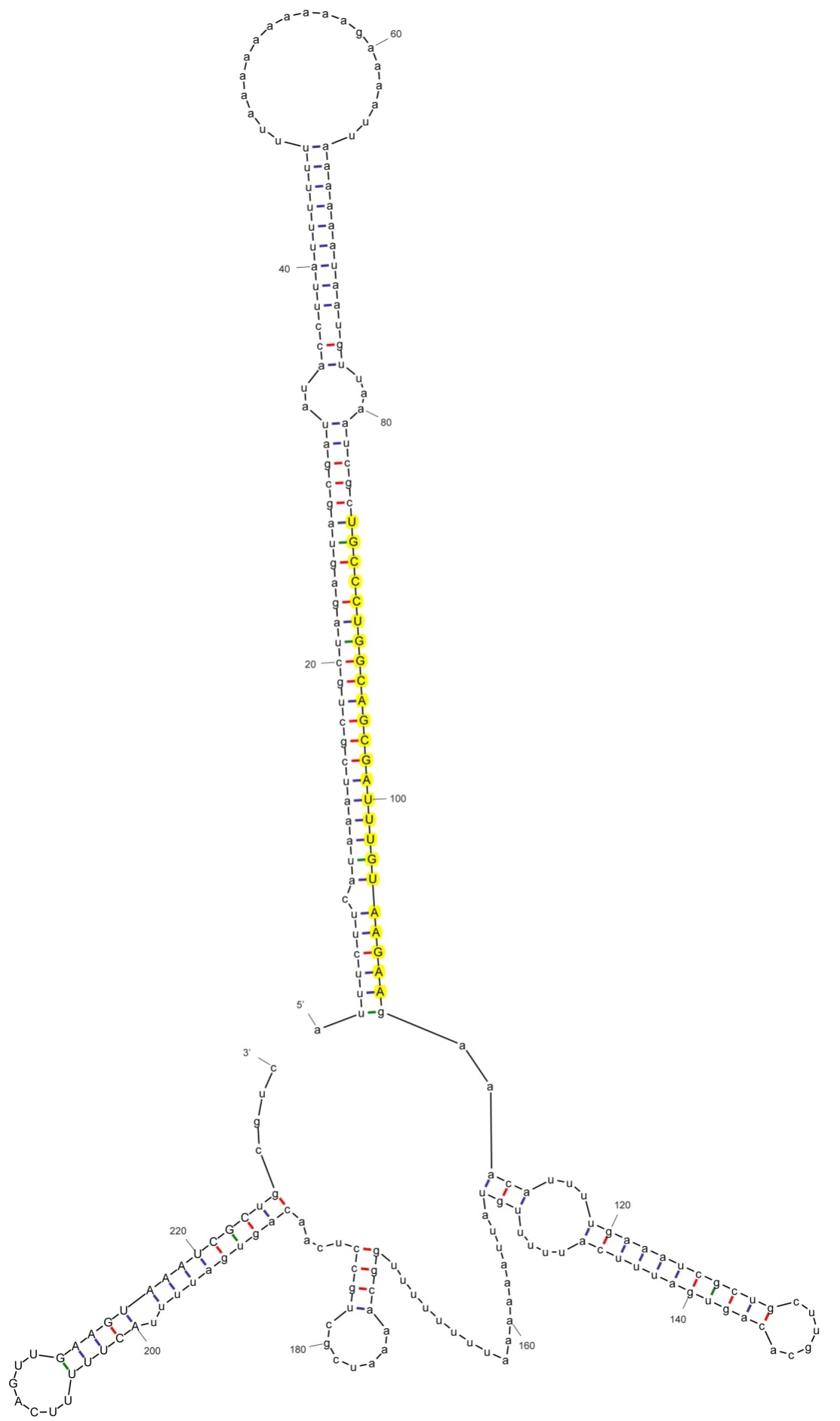


PC-1-5p

PC-2-5p


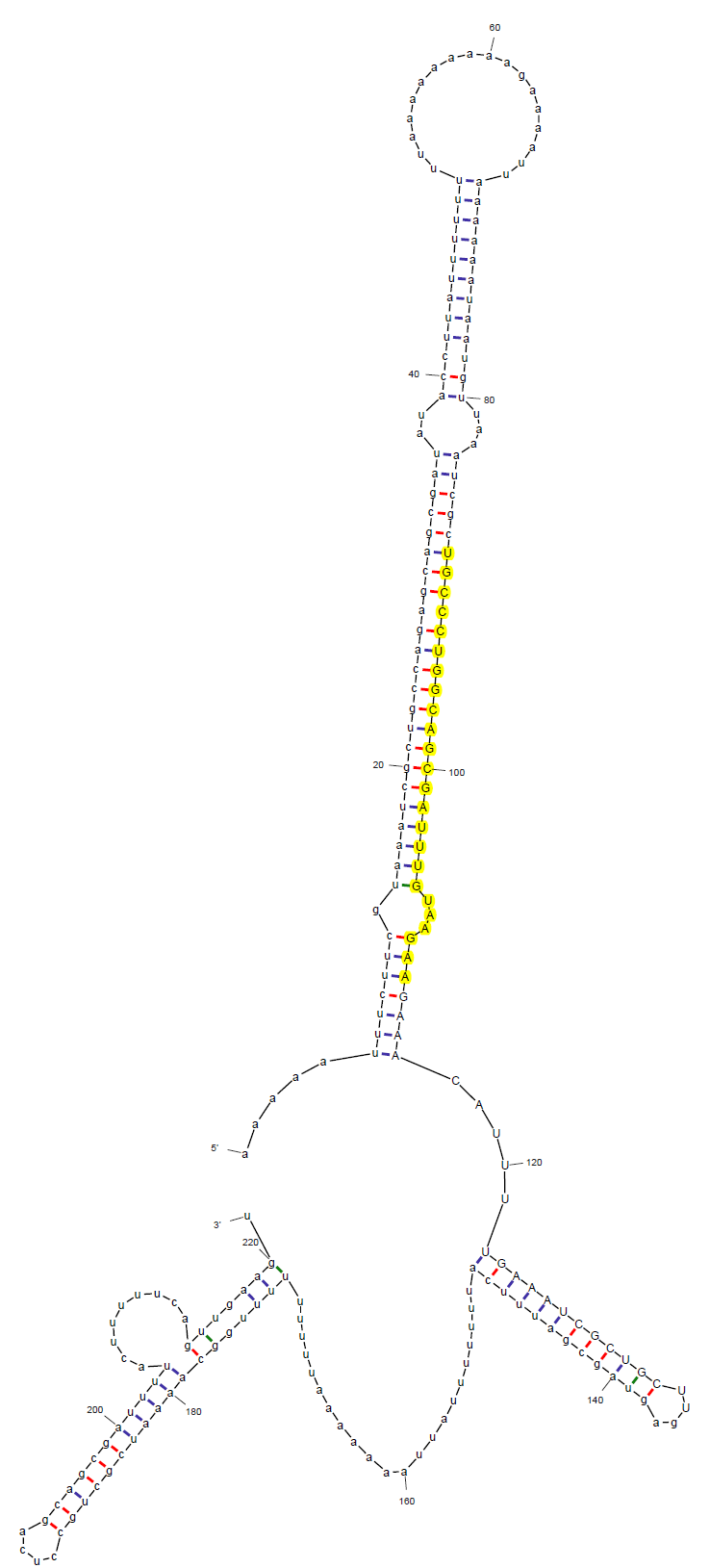


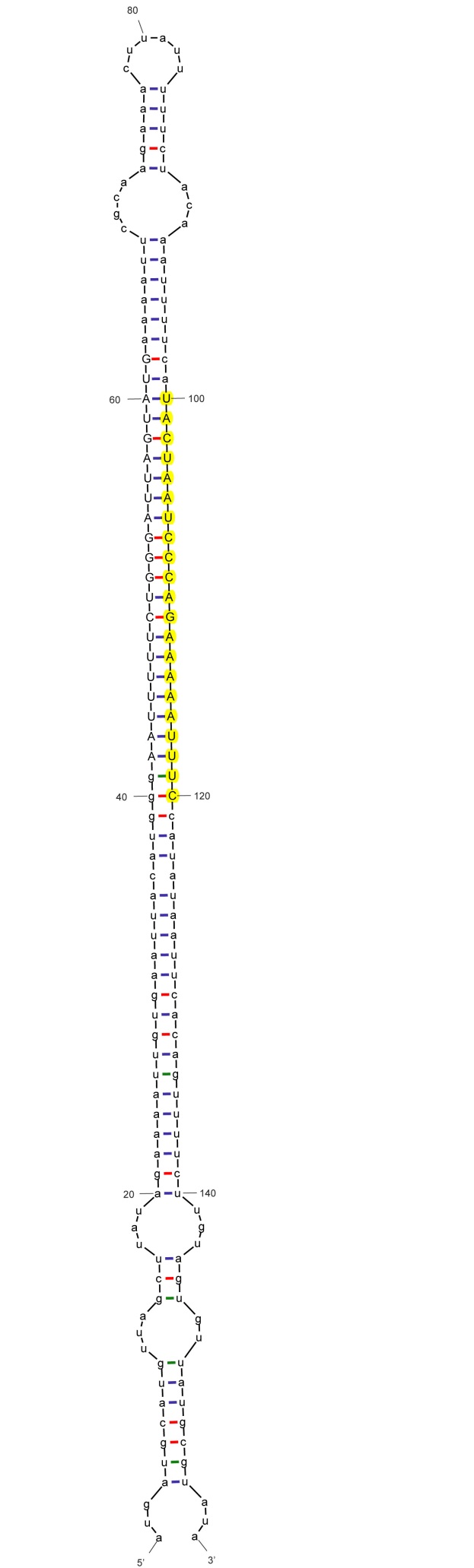


PC-3-3p


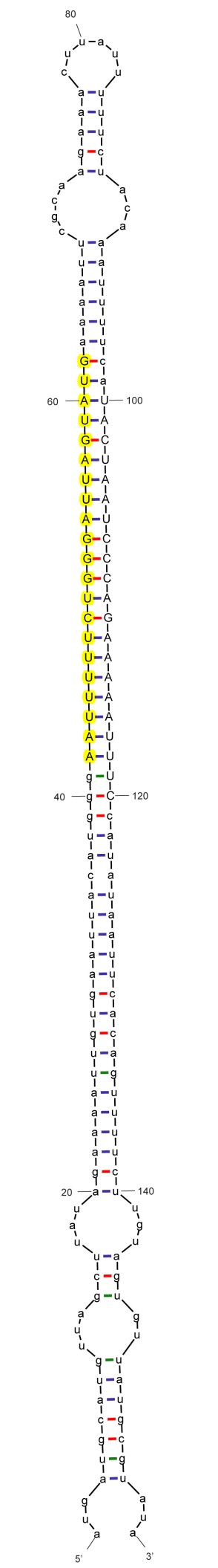


PC-3-5p


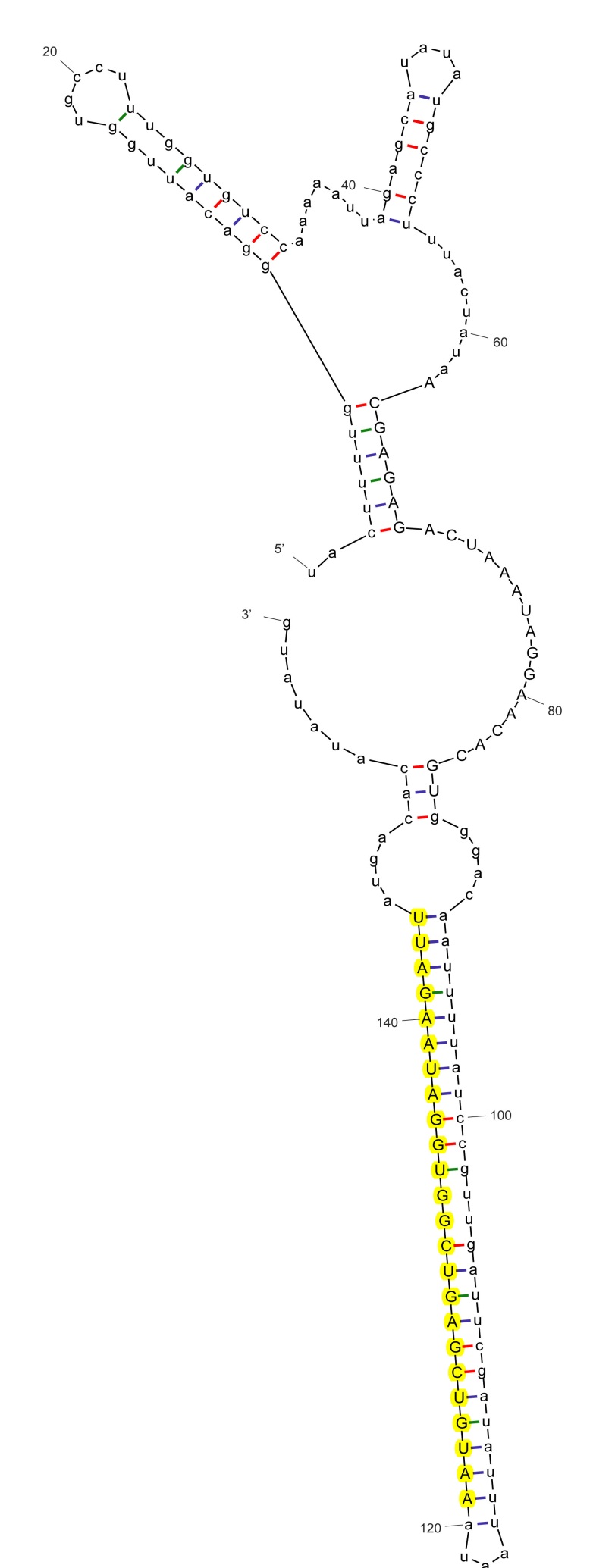


PC-4-3p


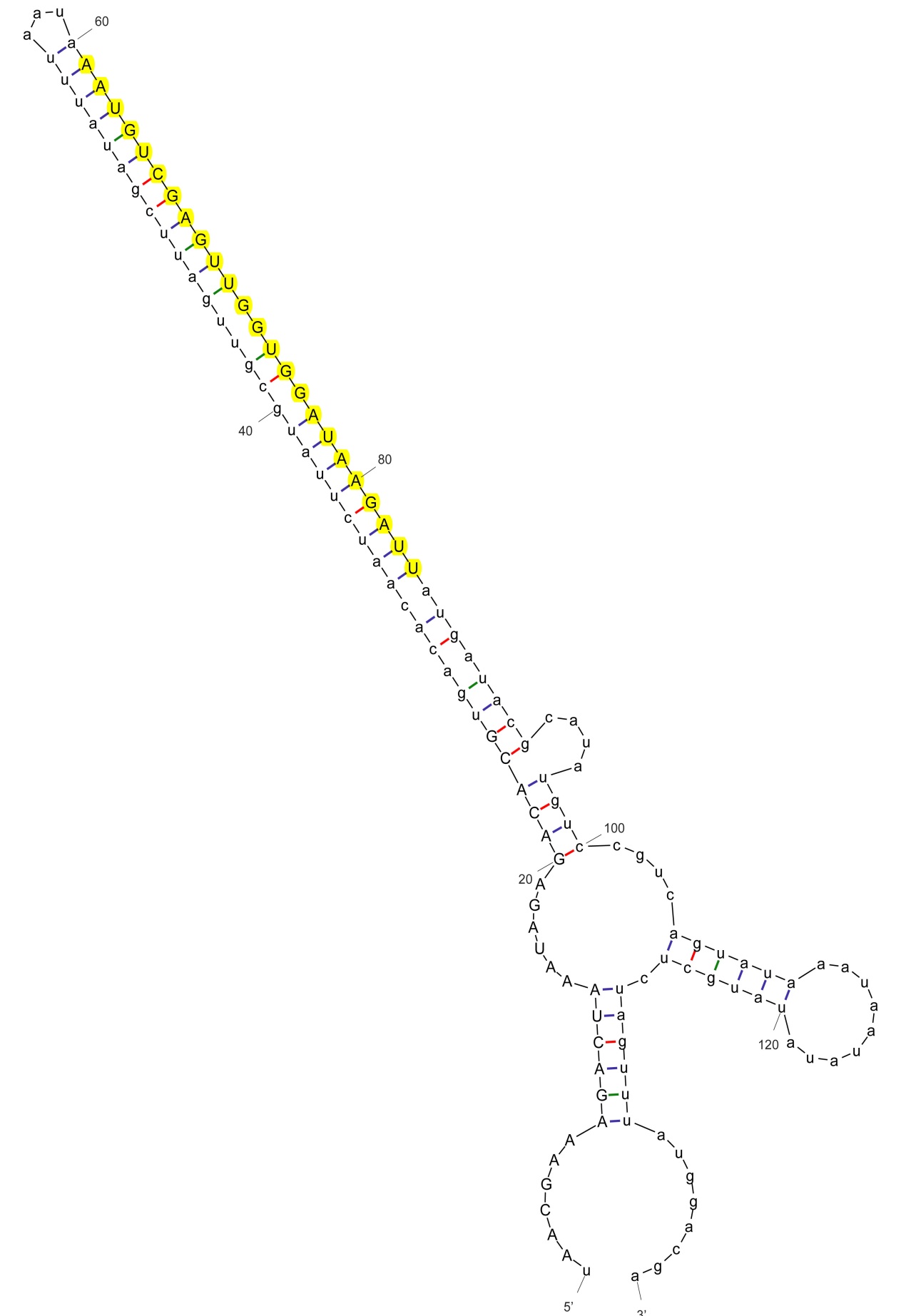


PC-5-3p


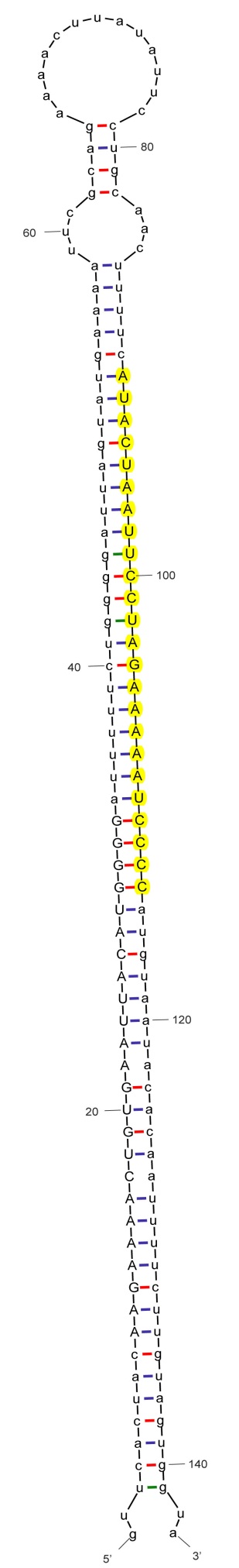


PC-6-3p


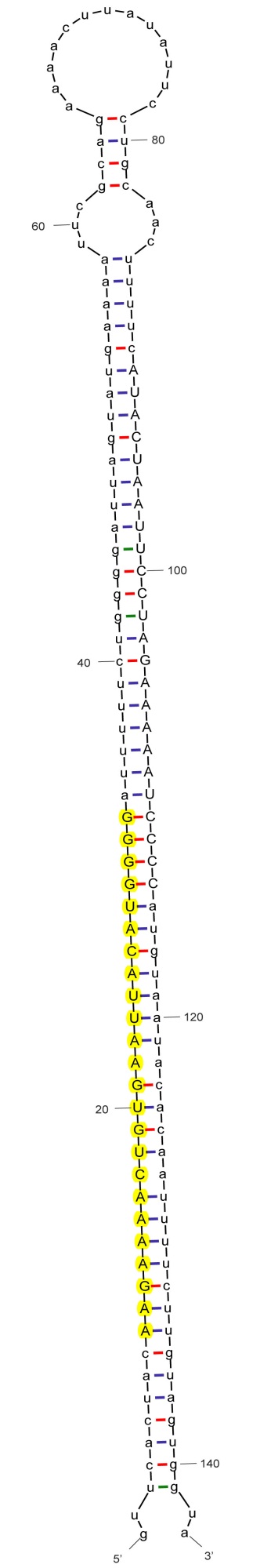


PC-6-5p


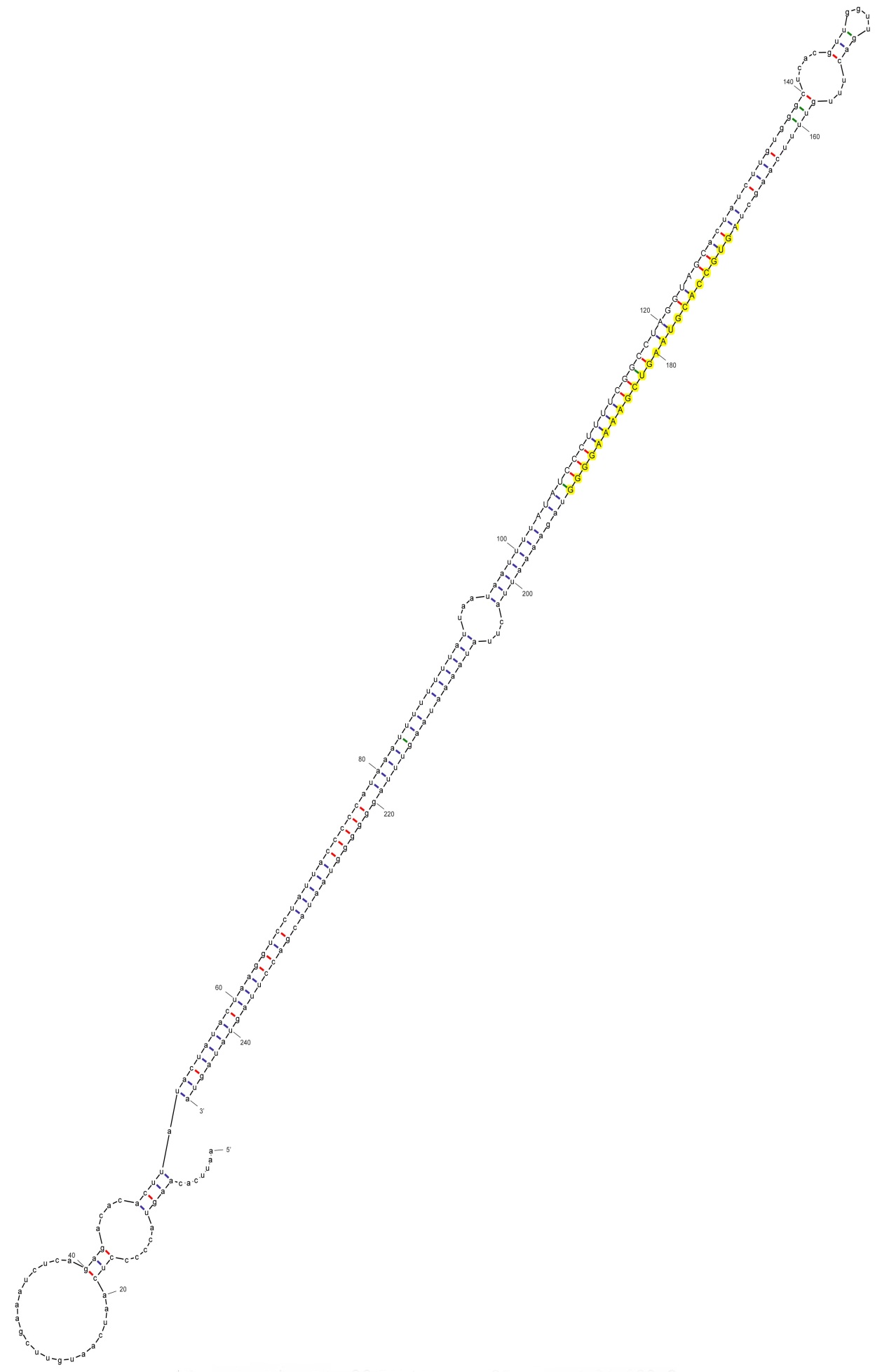


PC-7-3p


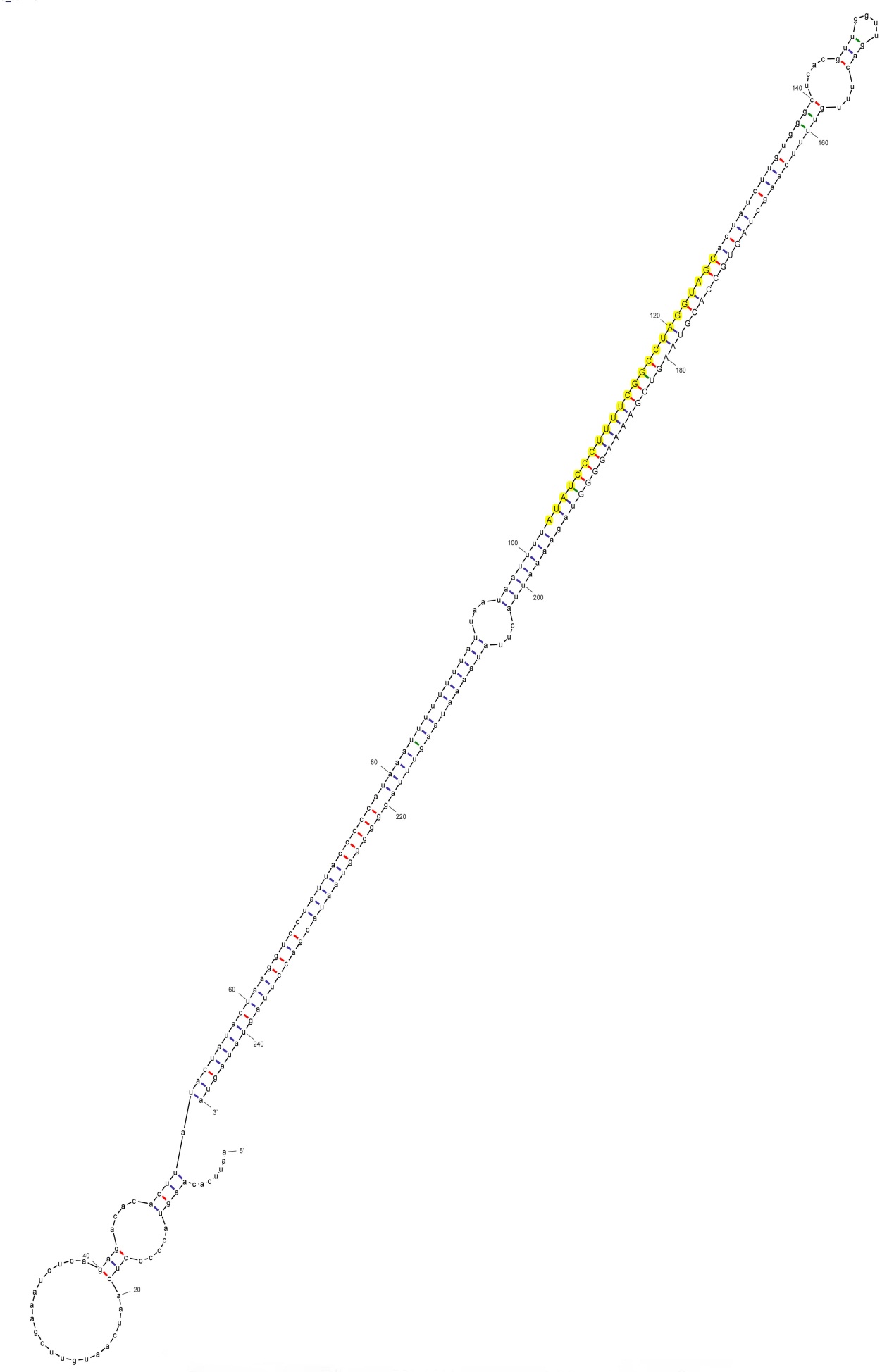


PC-7-5p


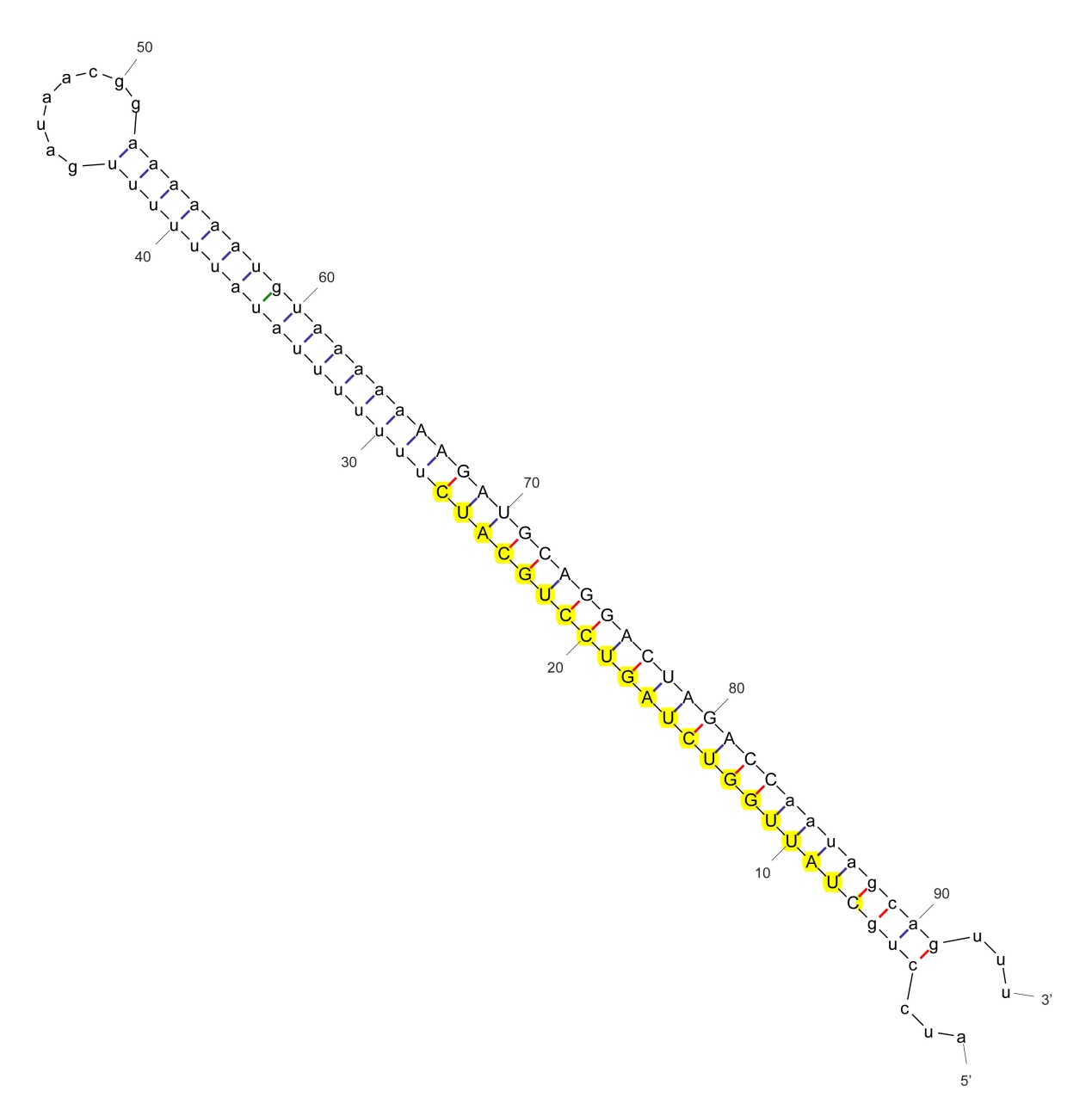


PC-8-5p


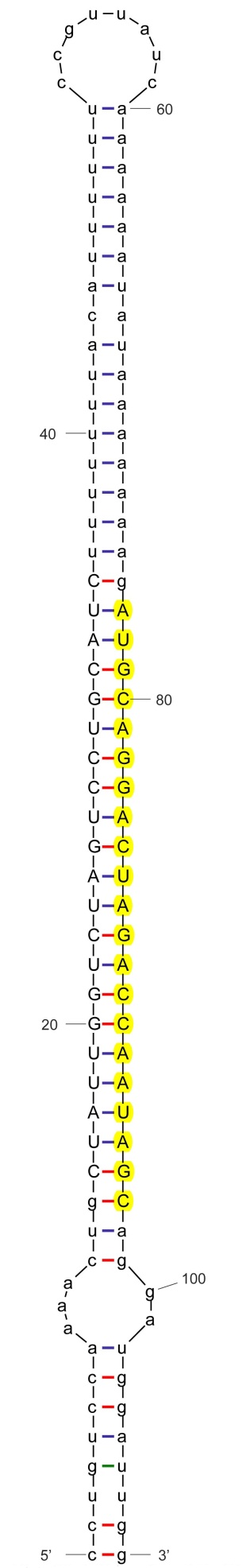


PC-9-3p


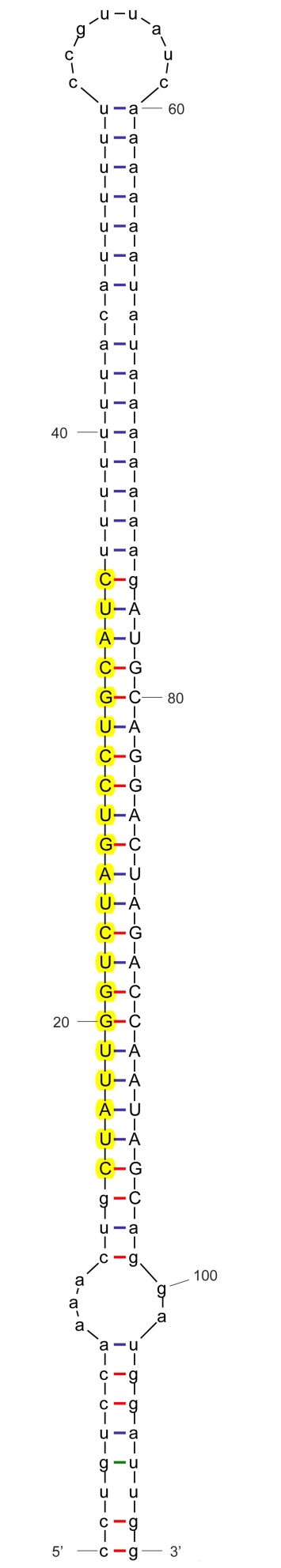


PC-9-5p


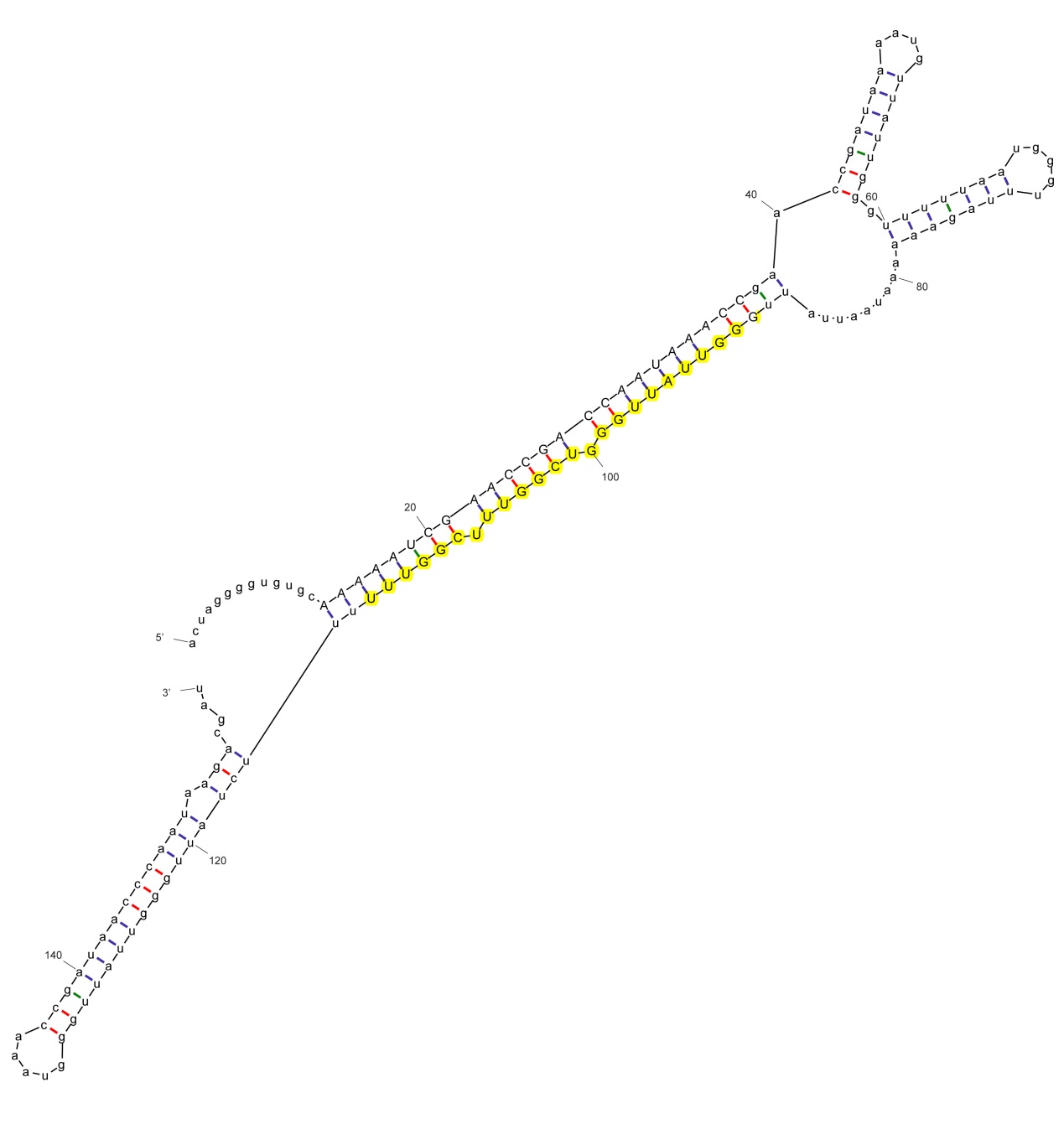


PC-10-3p


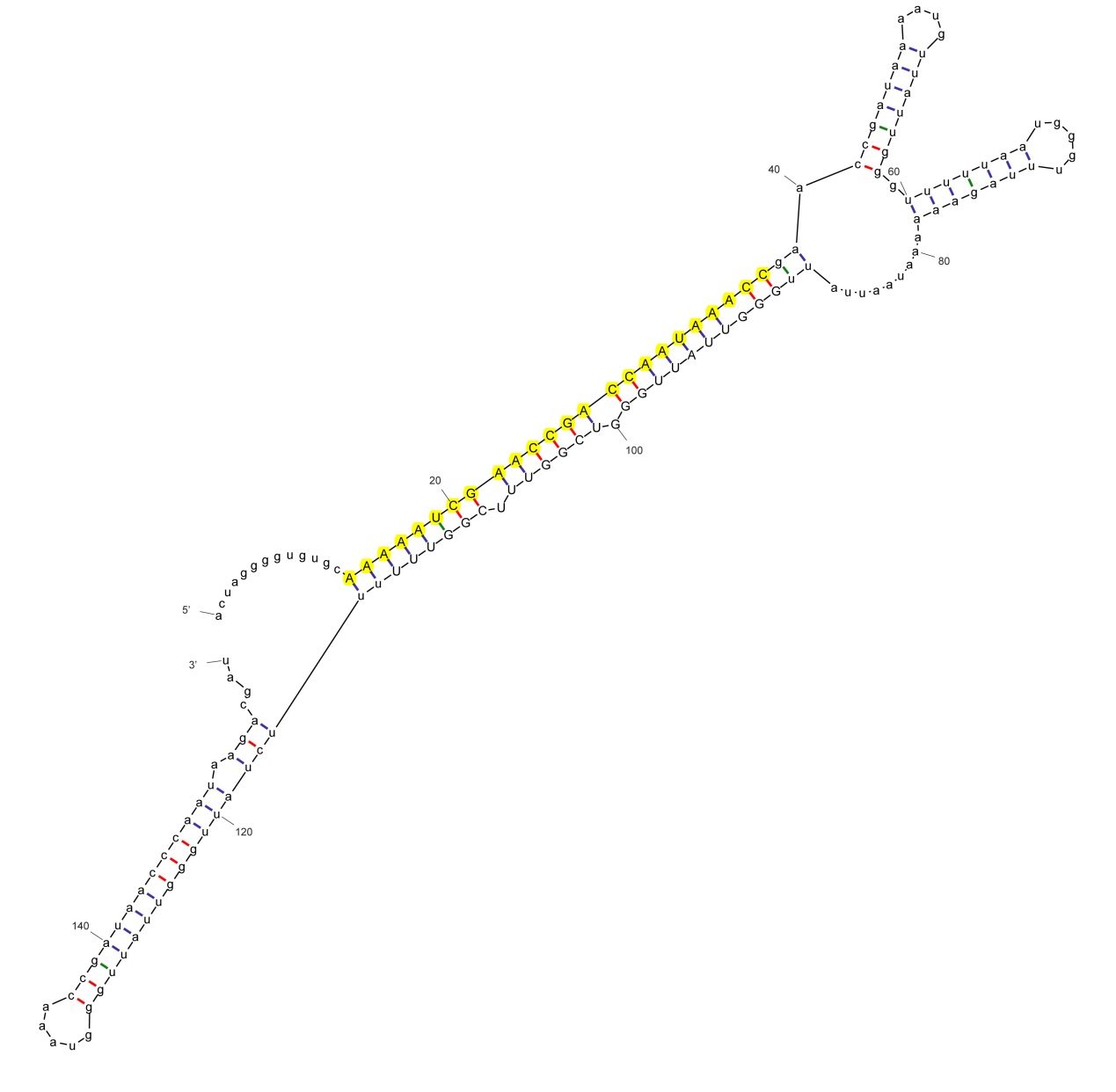


PC-10-5p

PC-10-3p


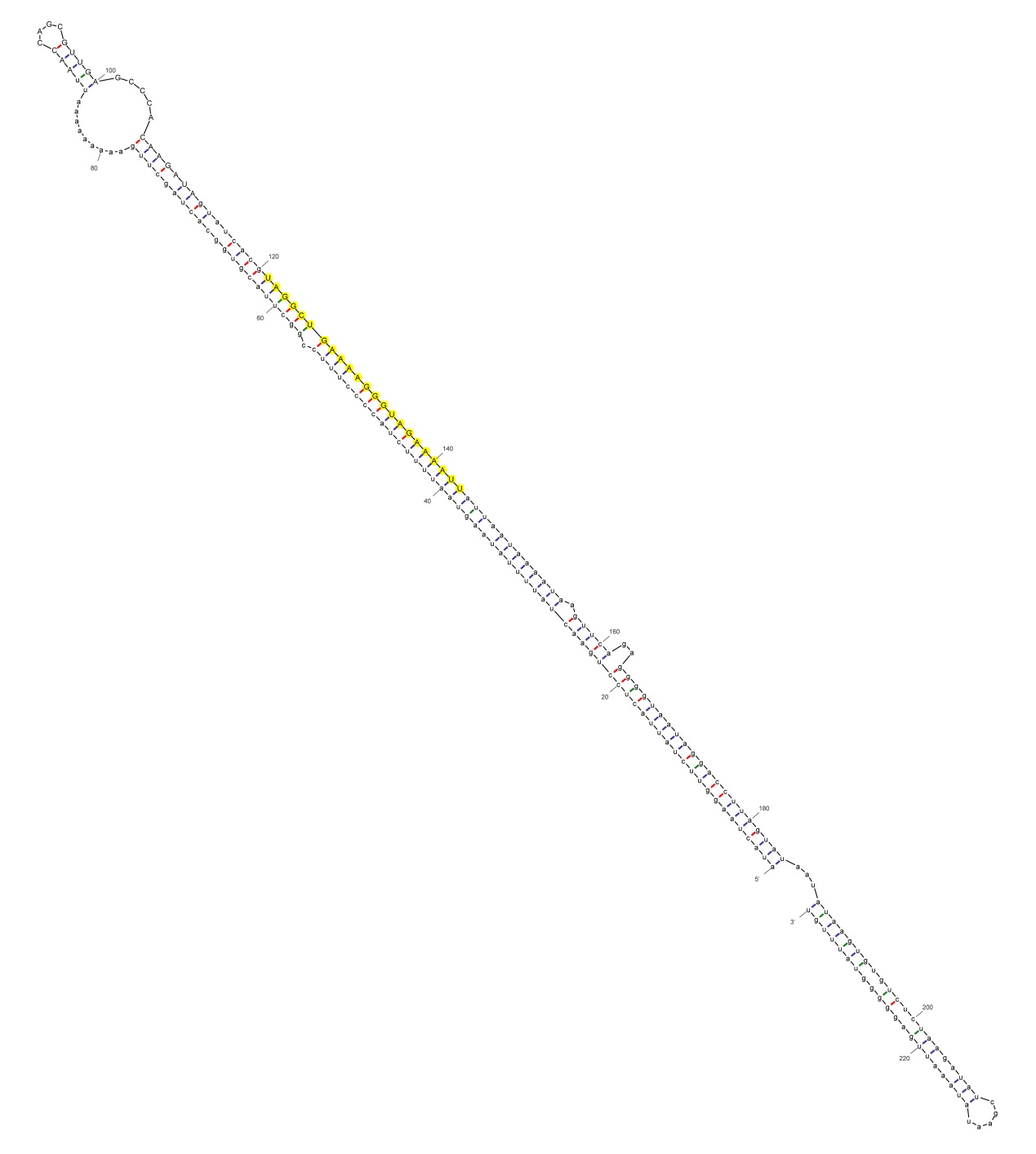


PC-11-3p


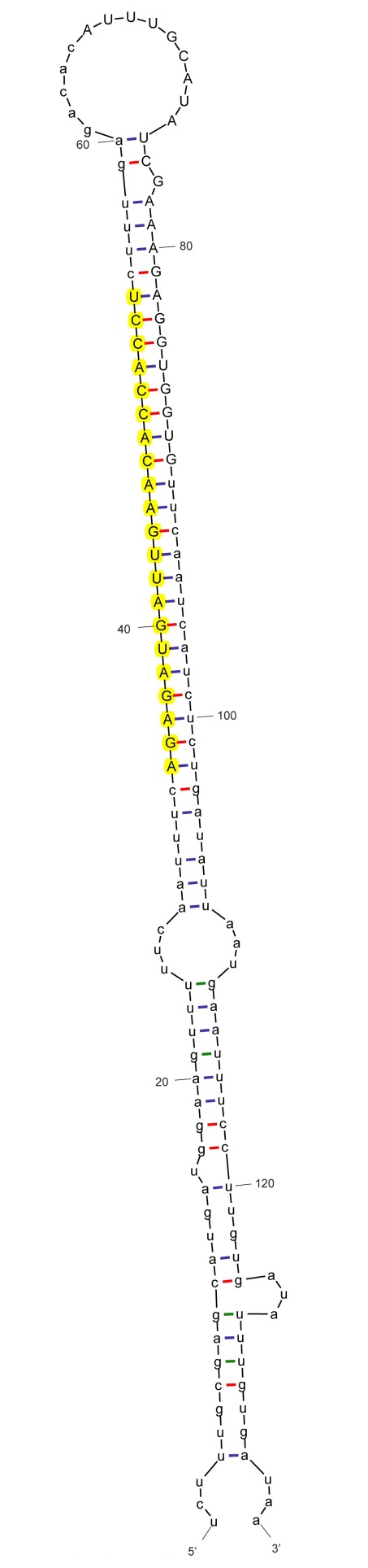


PC-12-5p


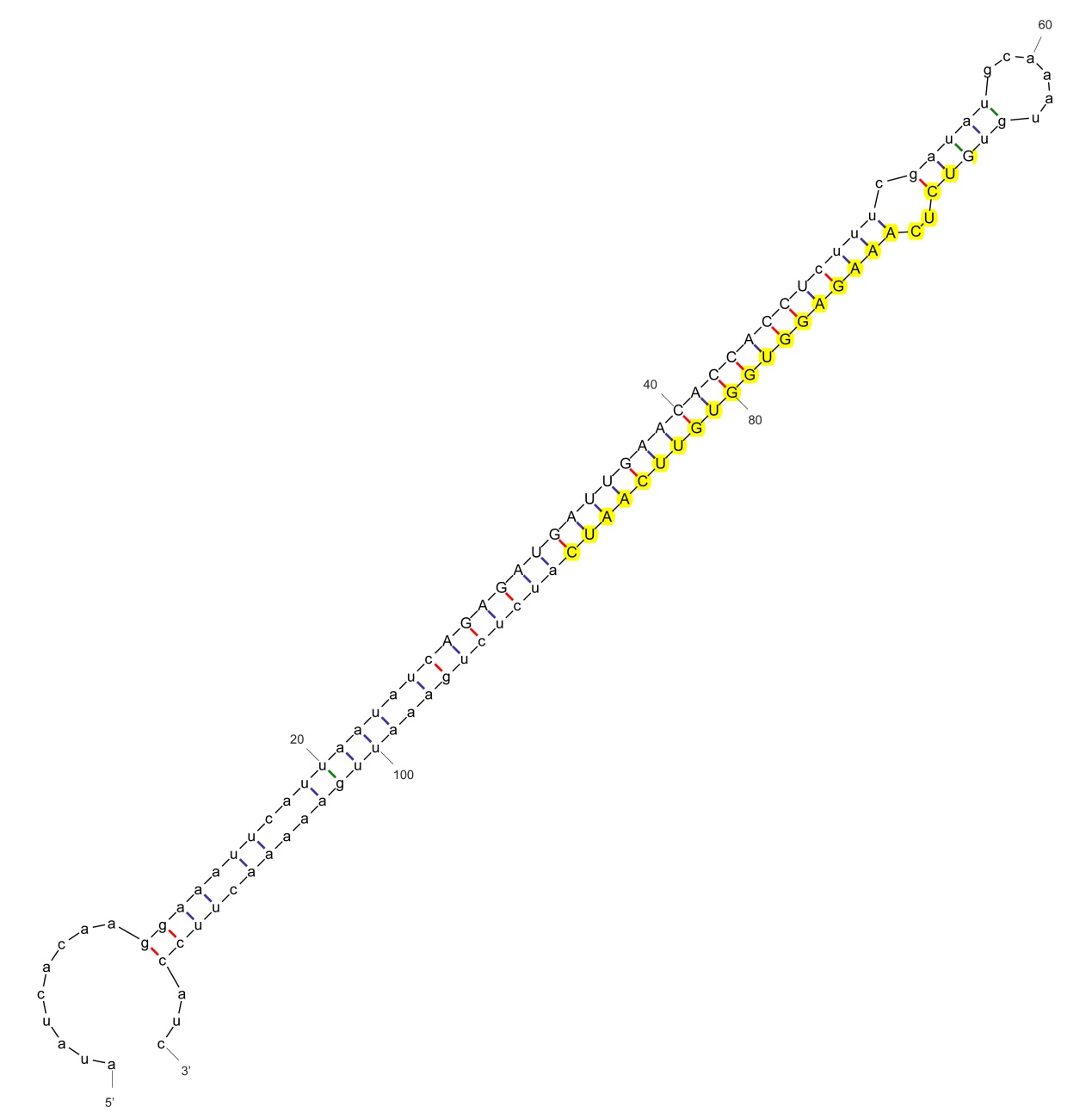


PC-13-3p


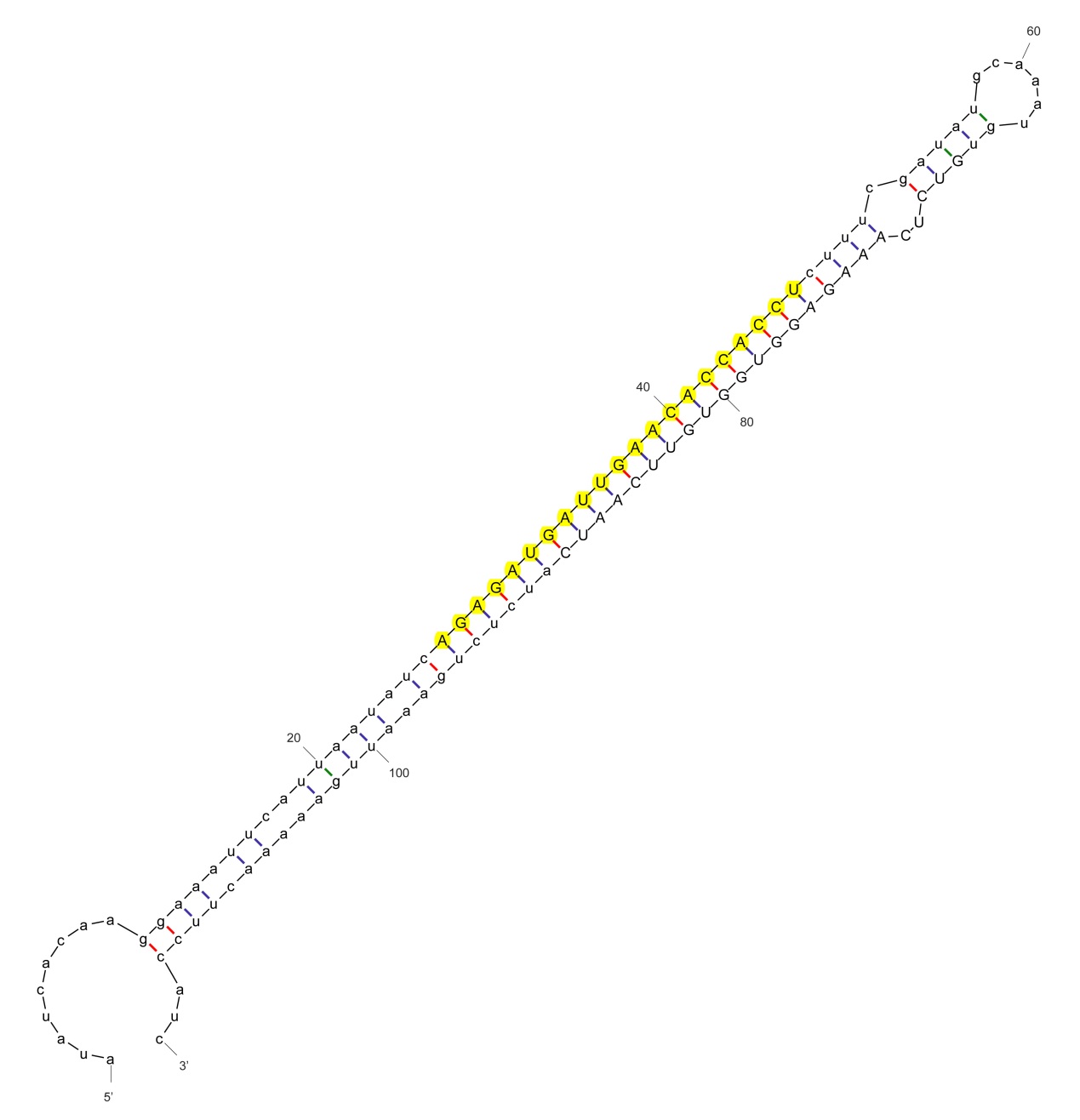


PC-13-5p


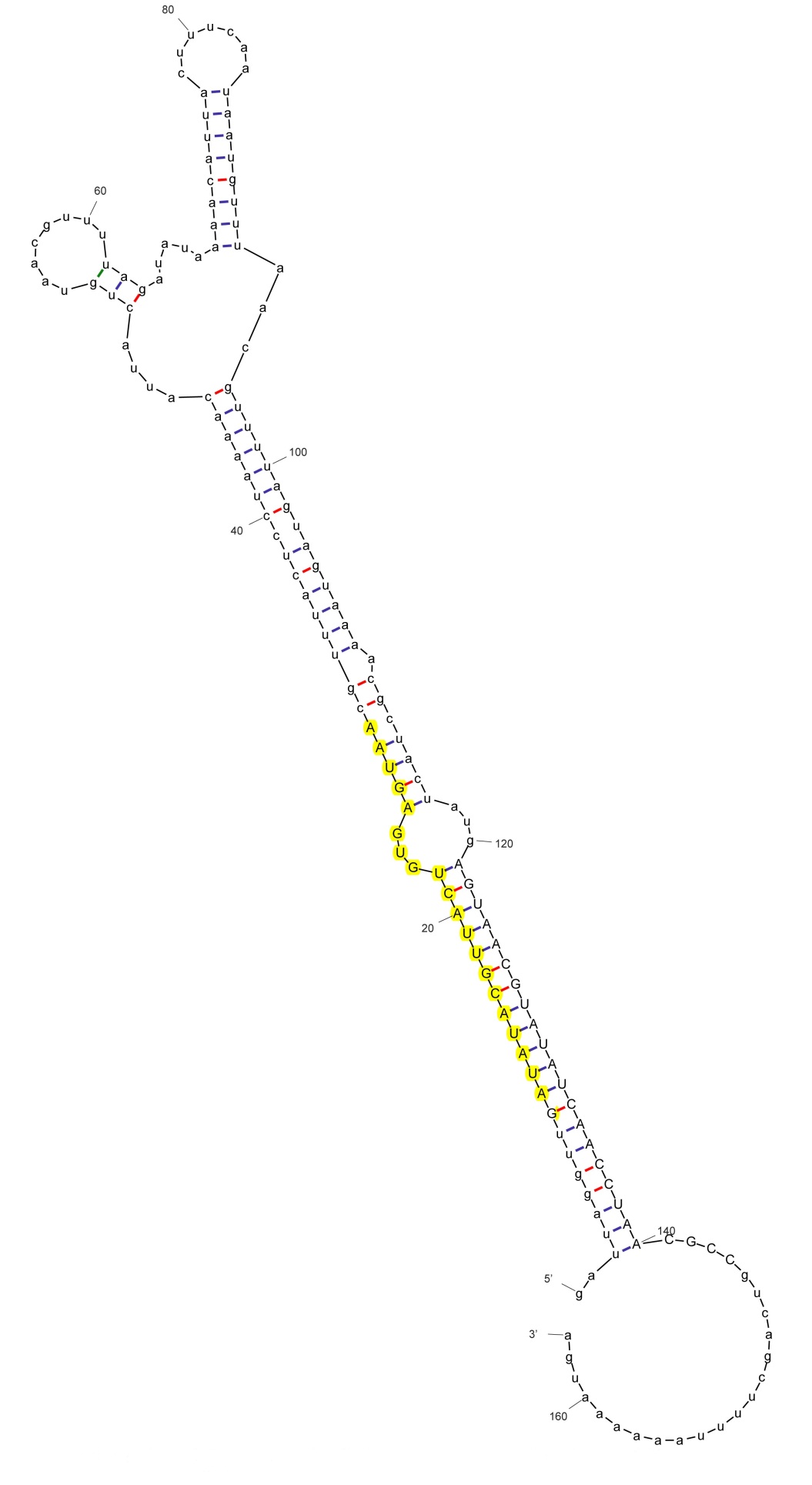


PC-14-5p


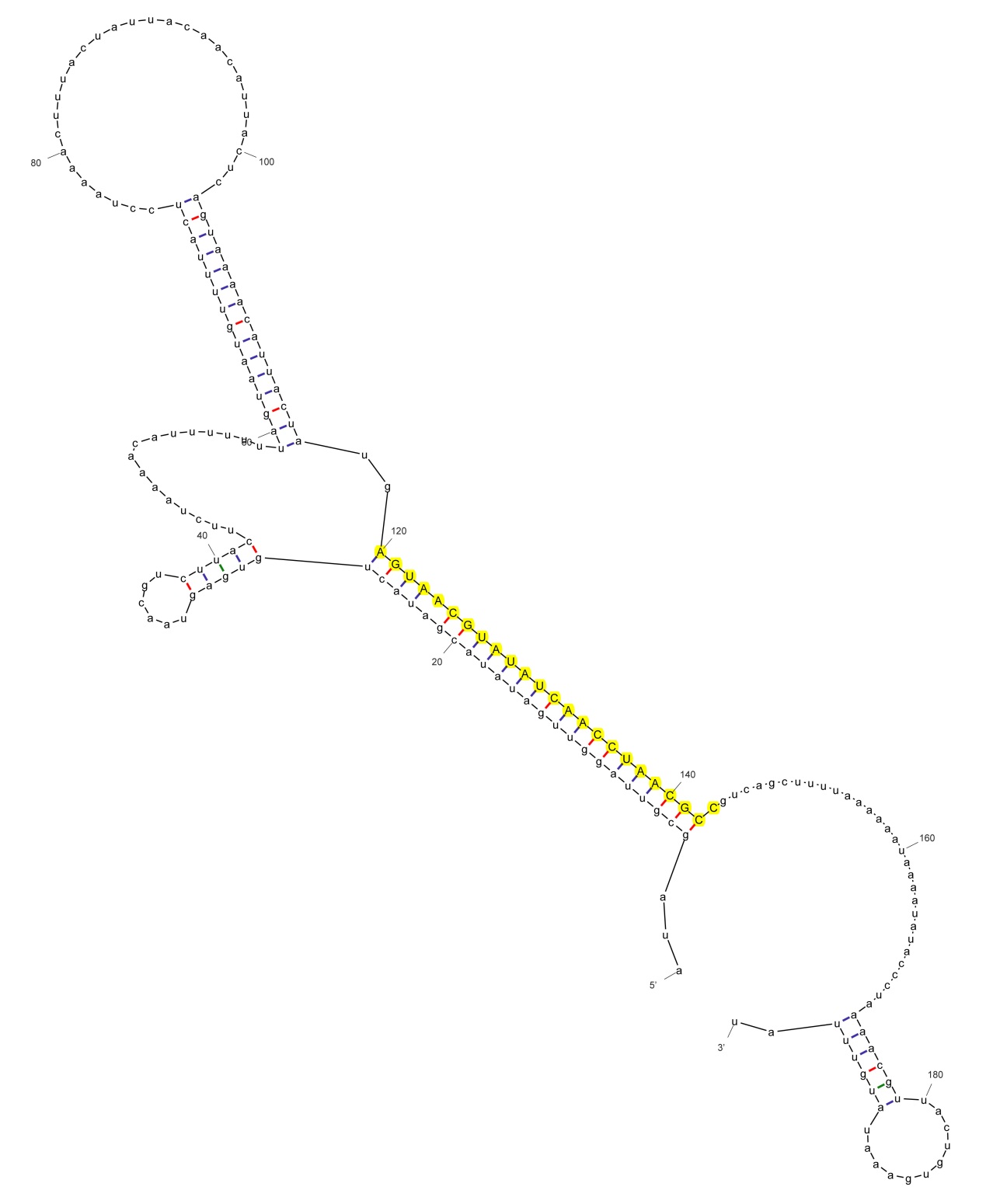


PC-15-3p


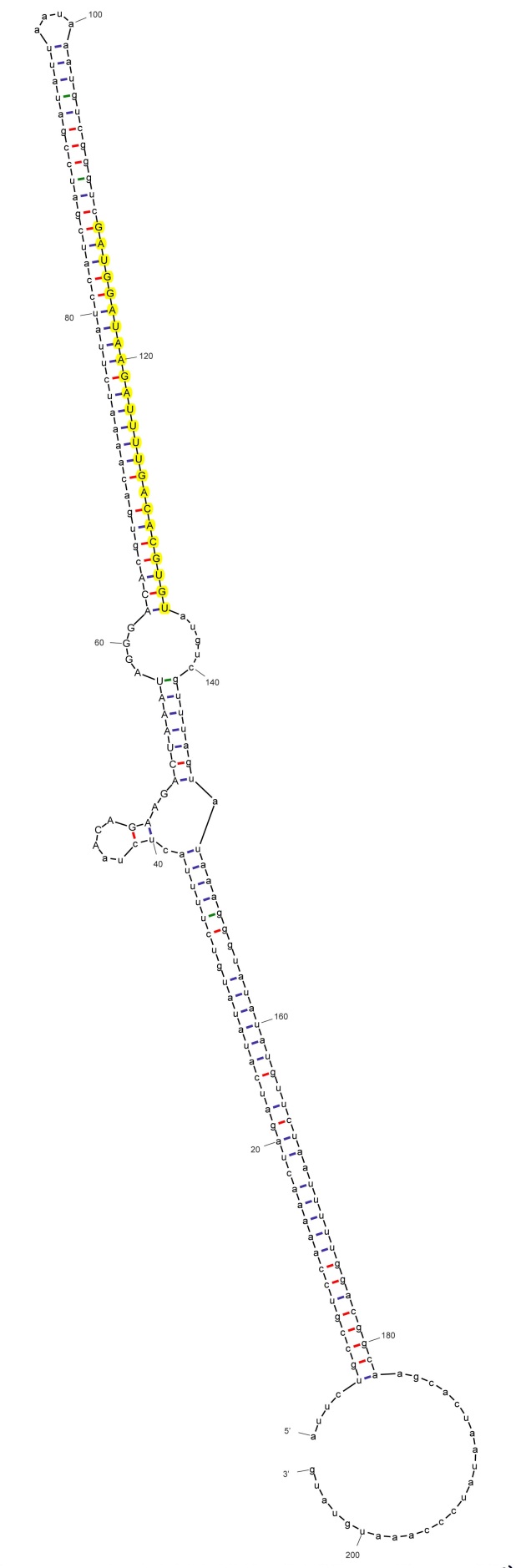


PC-16-3p


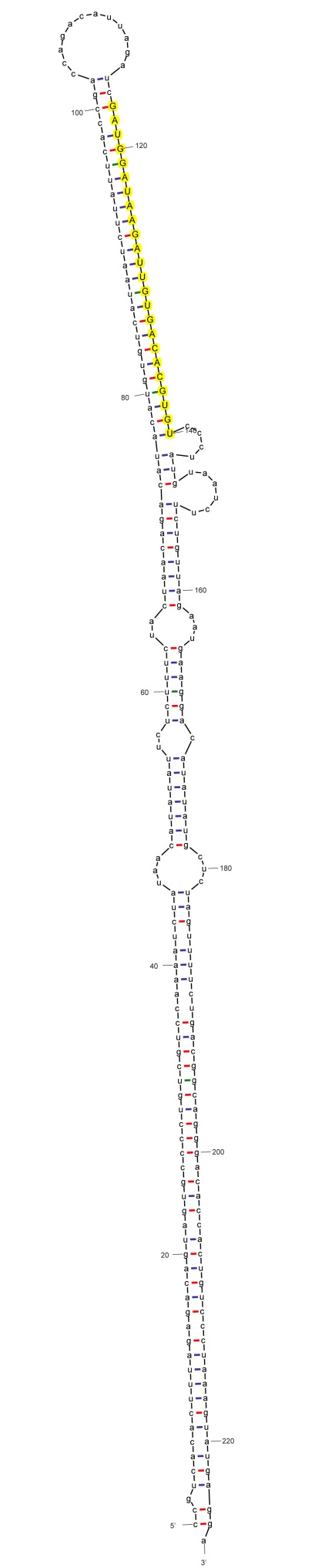


PC-17-3p


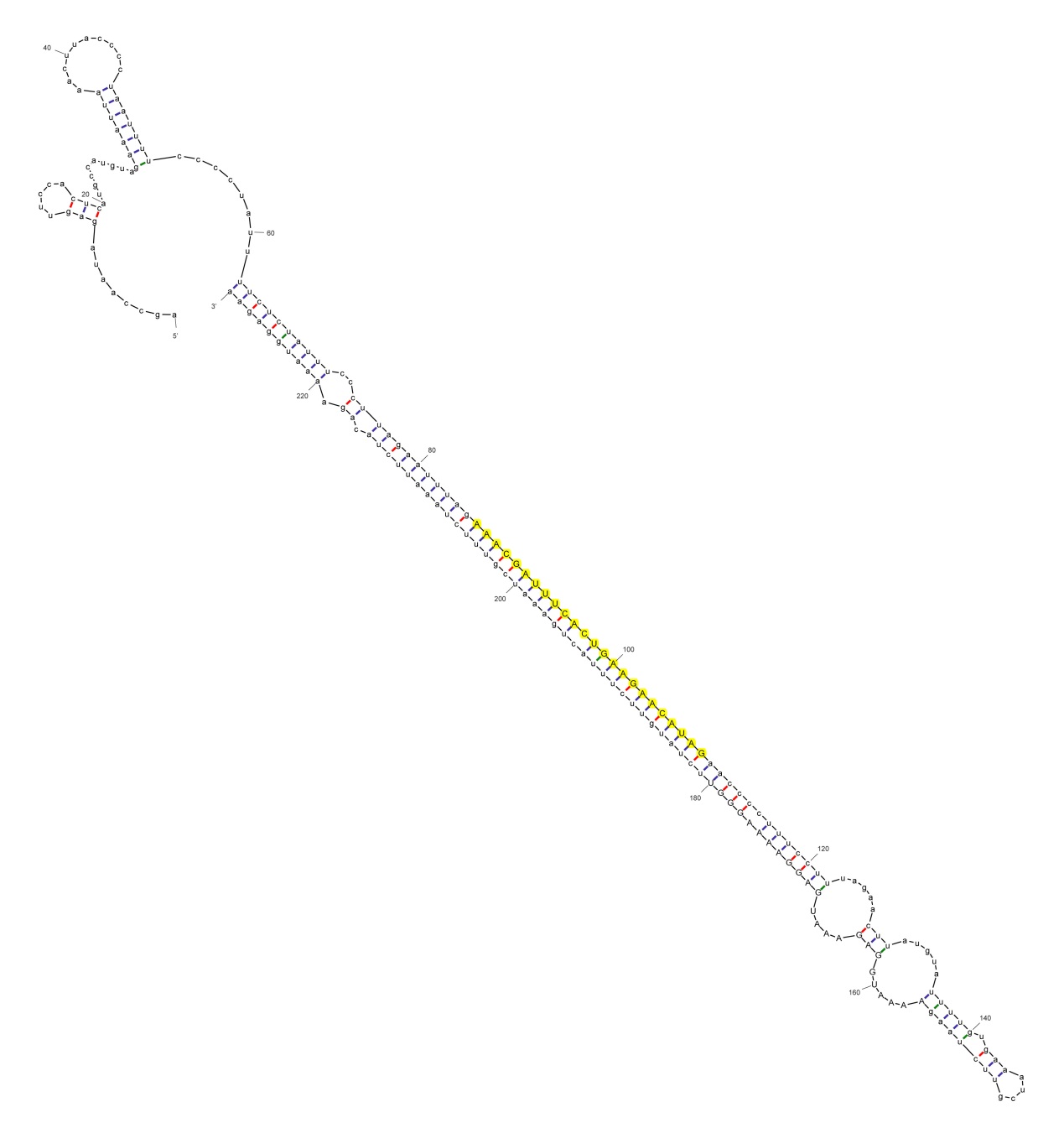


PC-18-5p


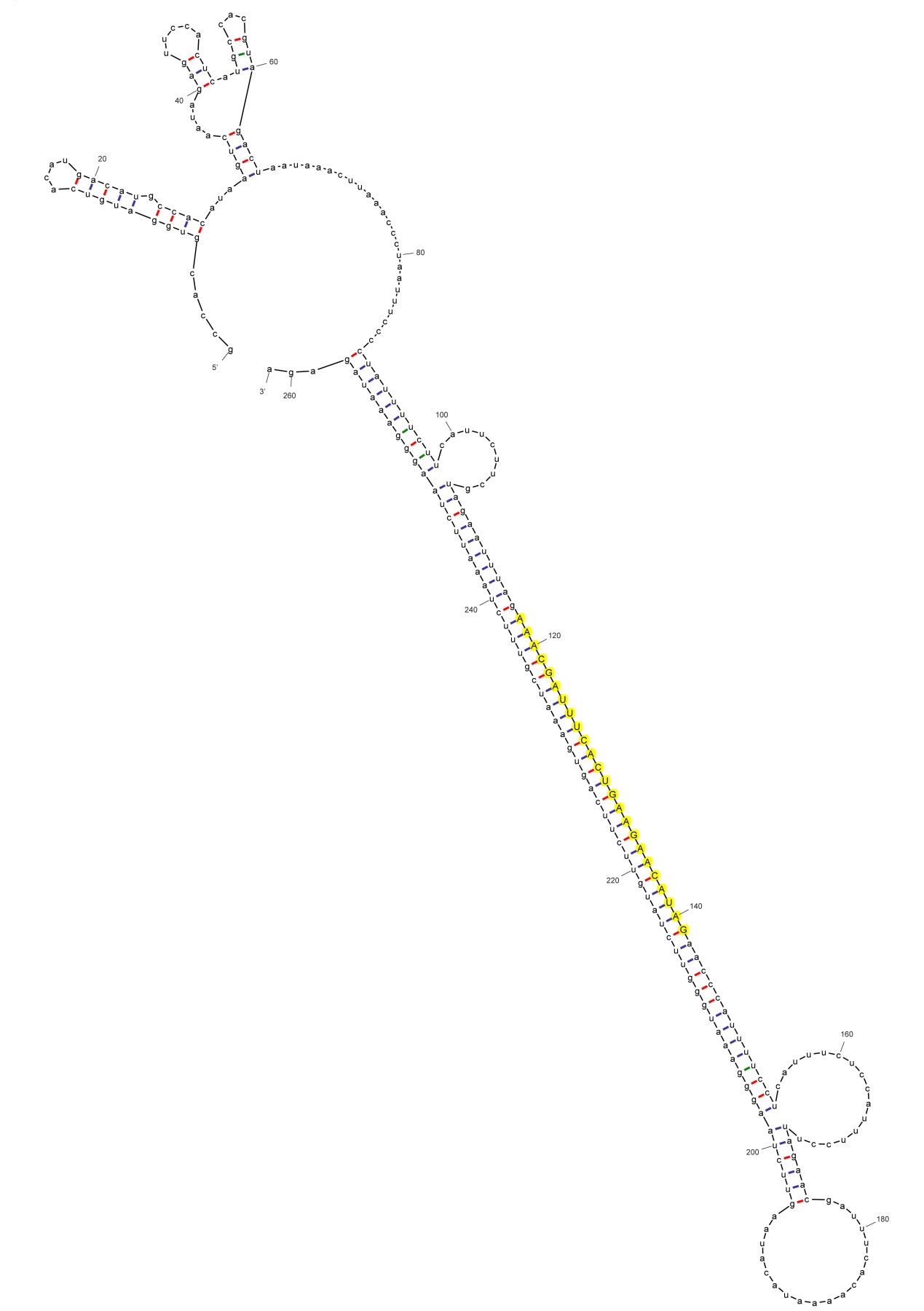


PC-19-5p


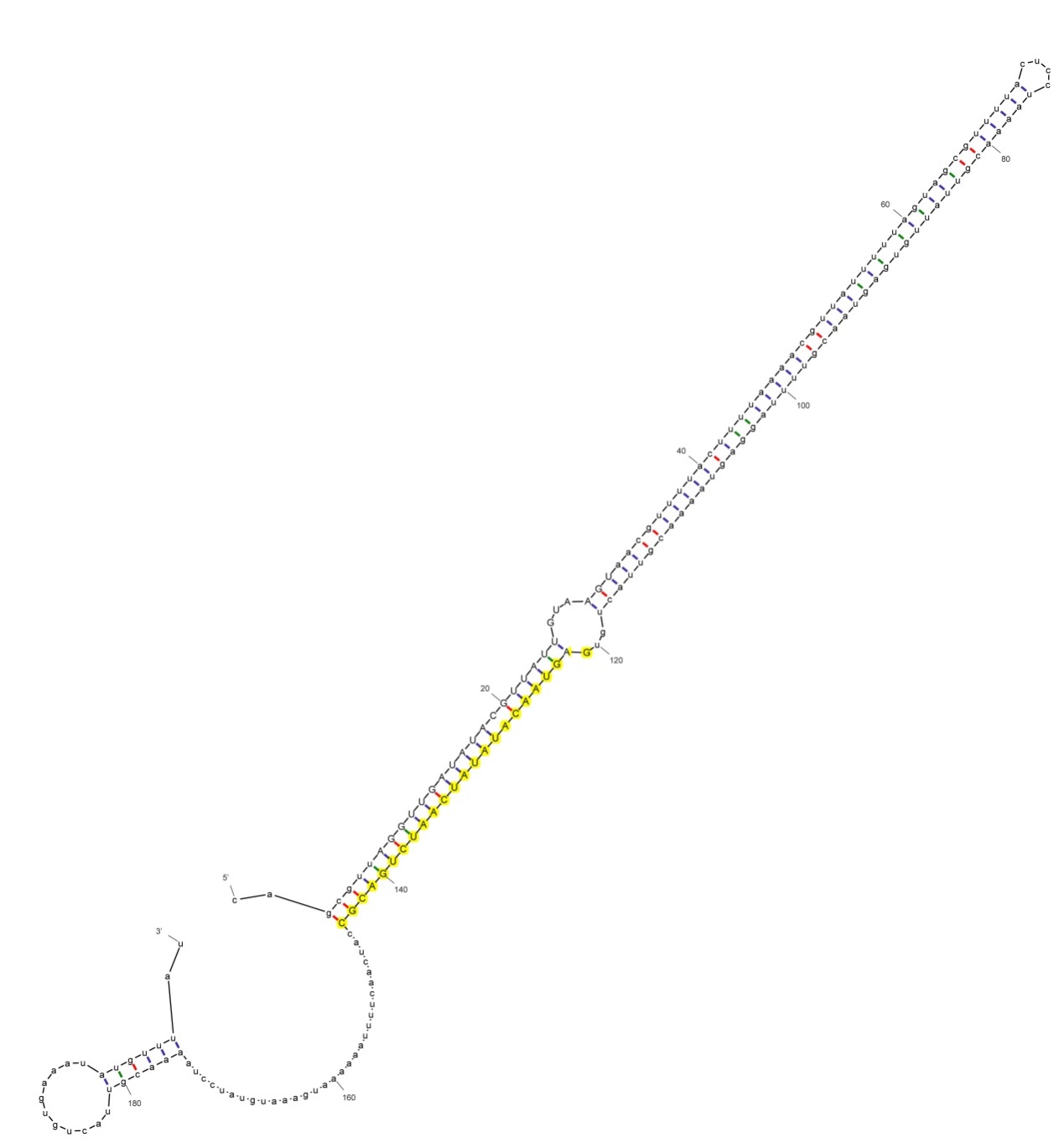


PC-20-3p

**
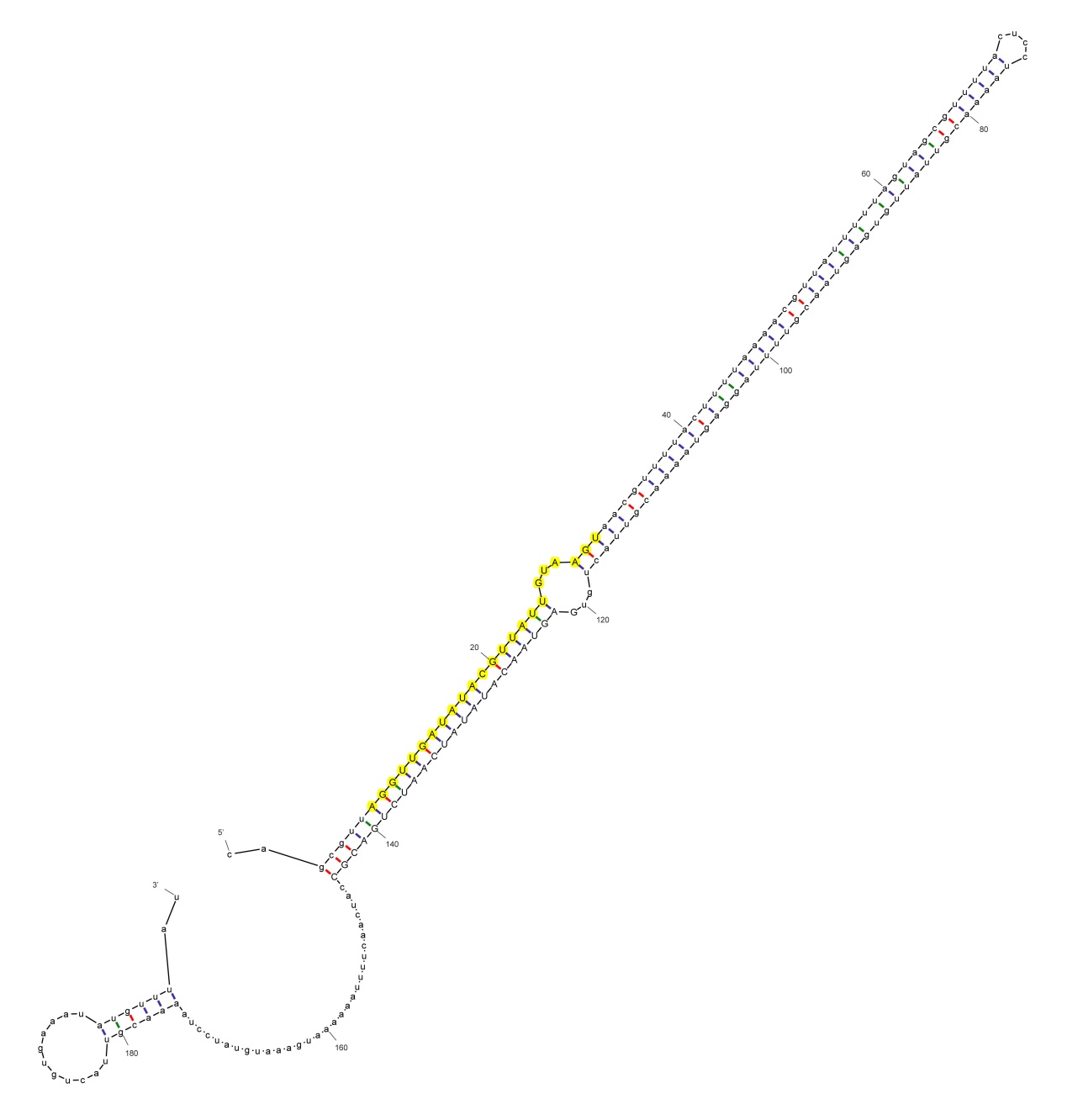
**

PC-20-5p


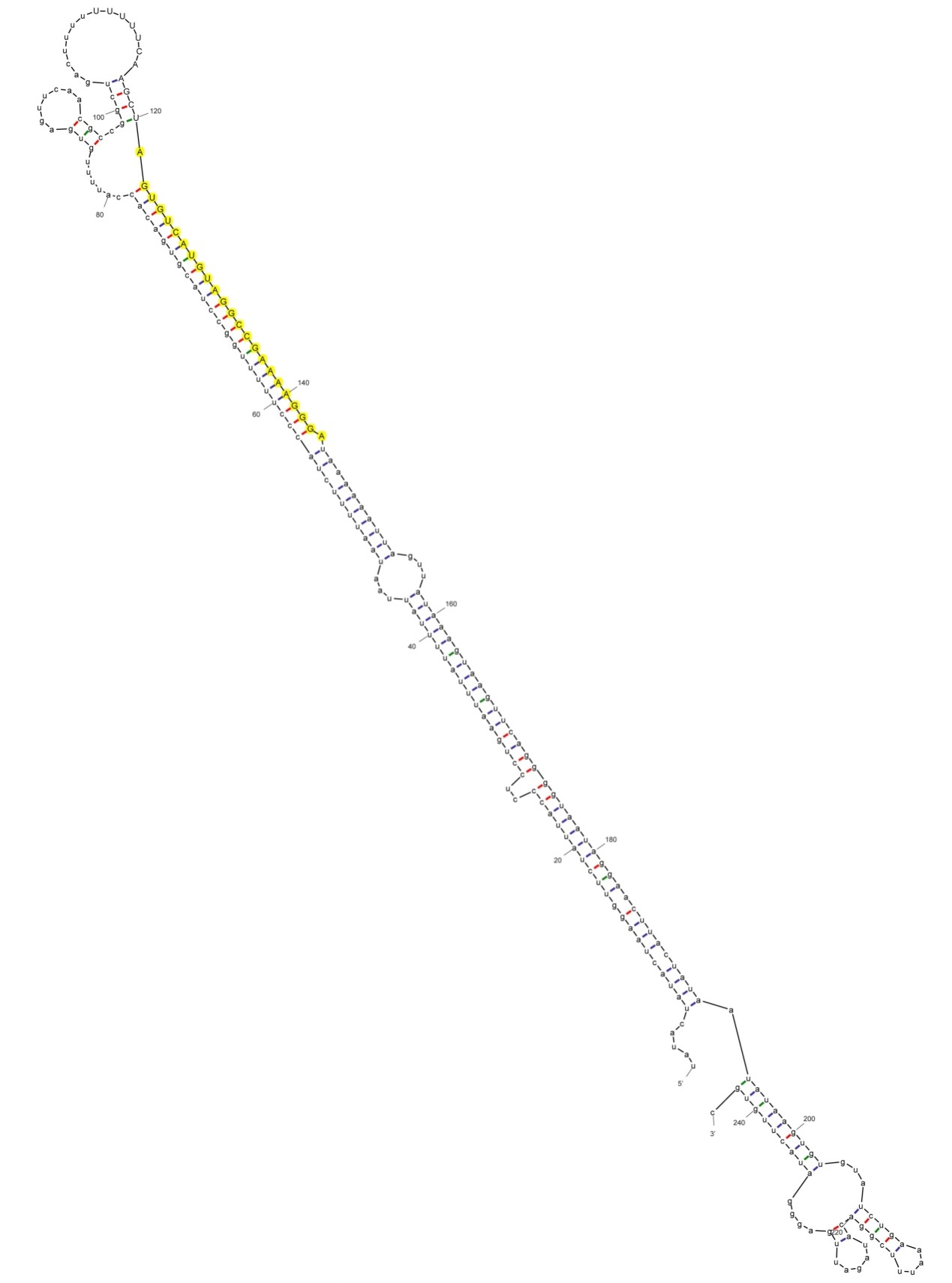


PC-21-3p


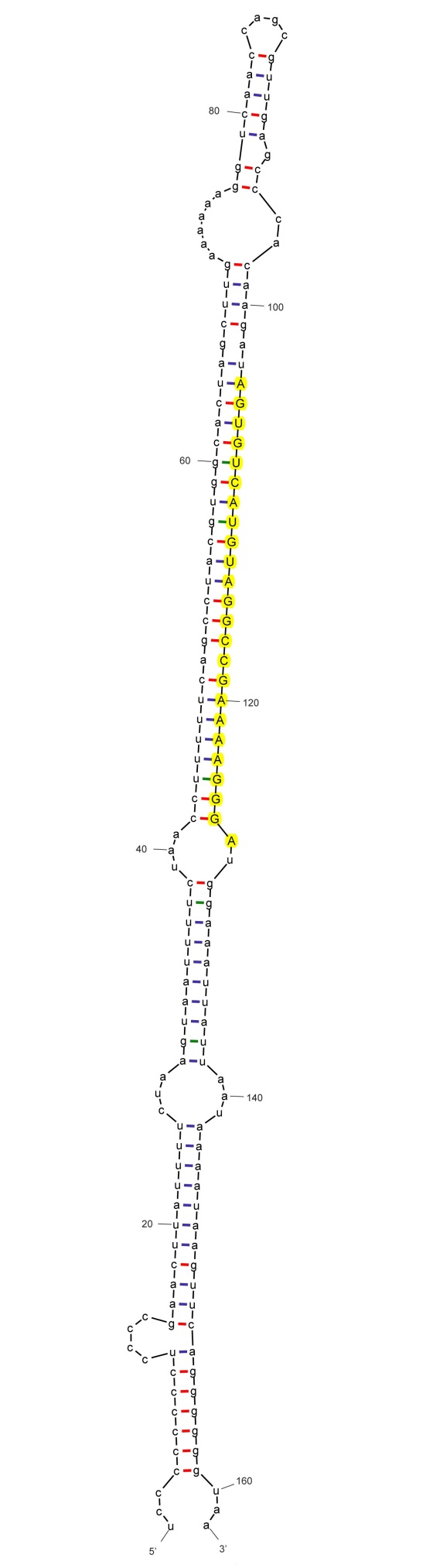


PC-22-3p


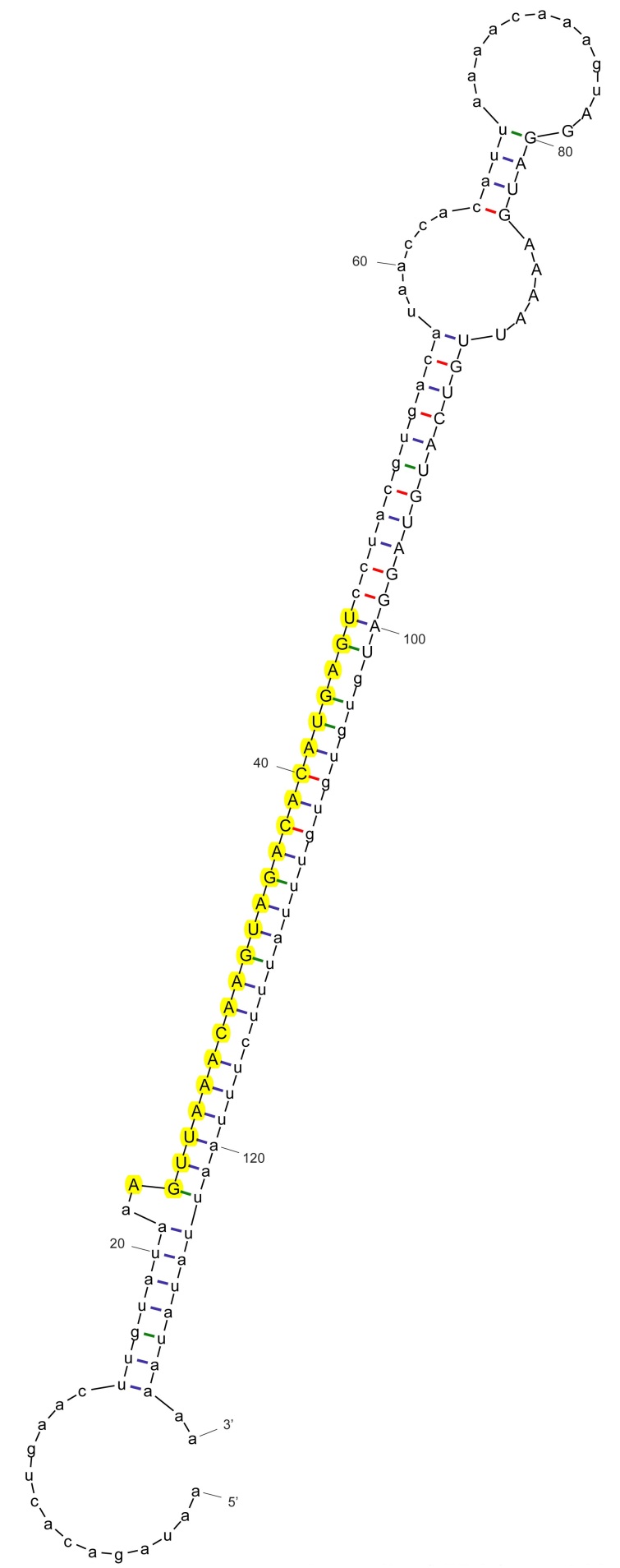


PC-23-5p


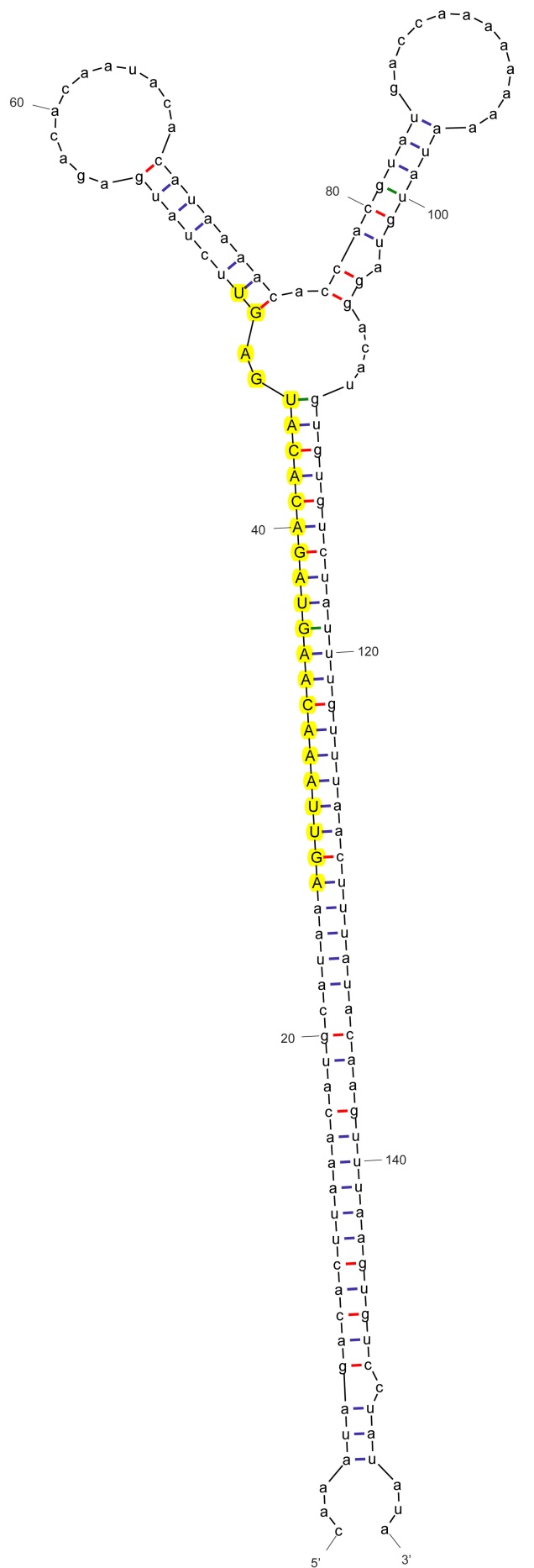


PC-24-5p


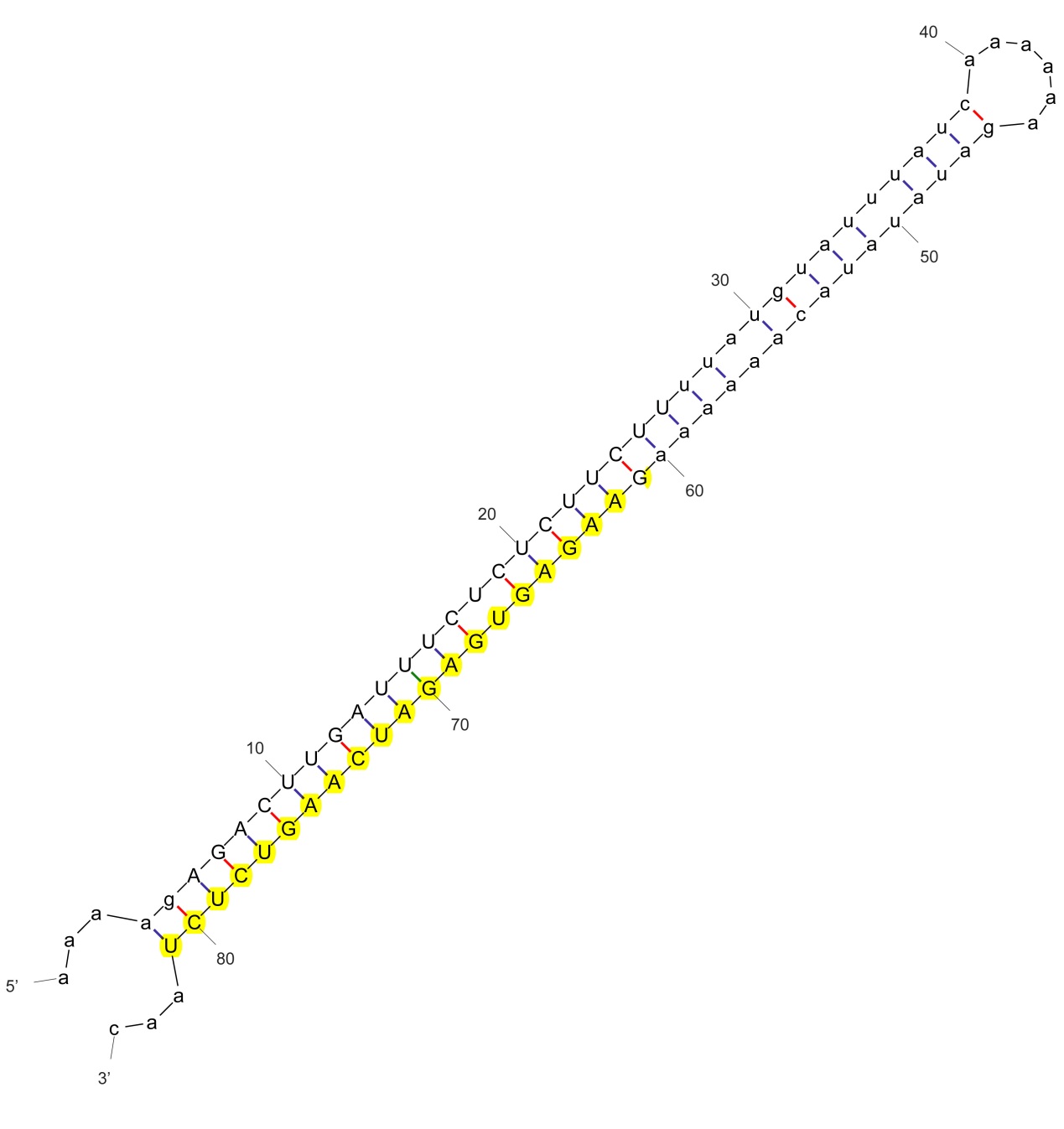


PC-25-3p


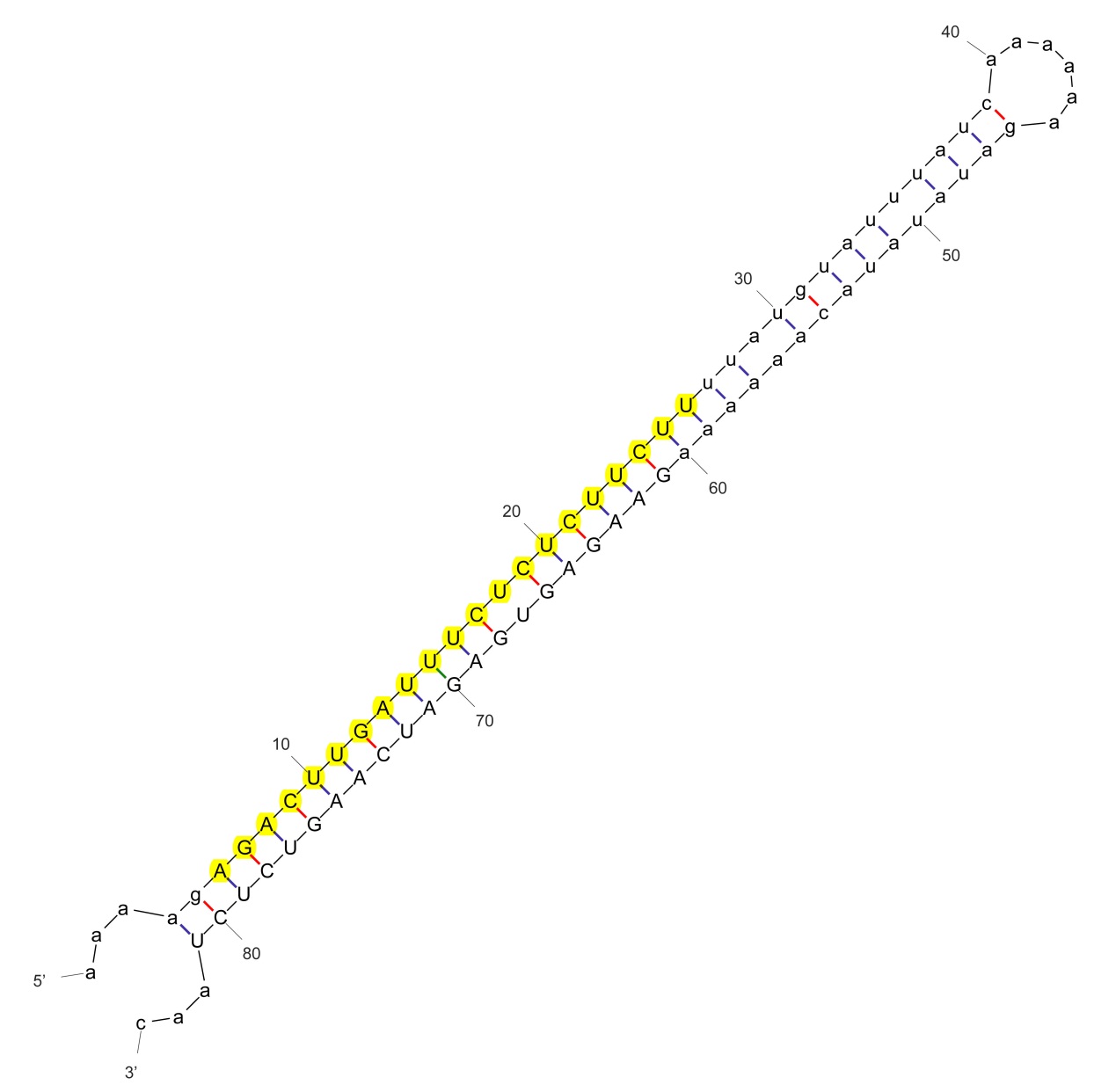


PC-25-5p


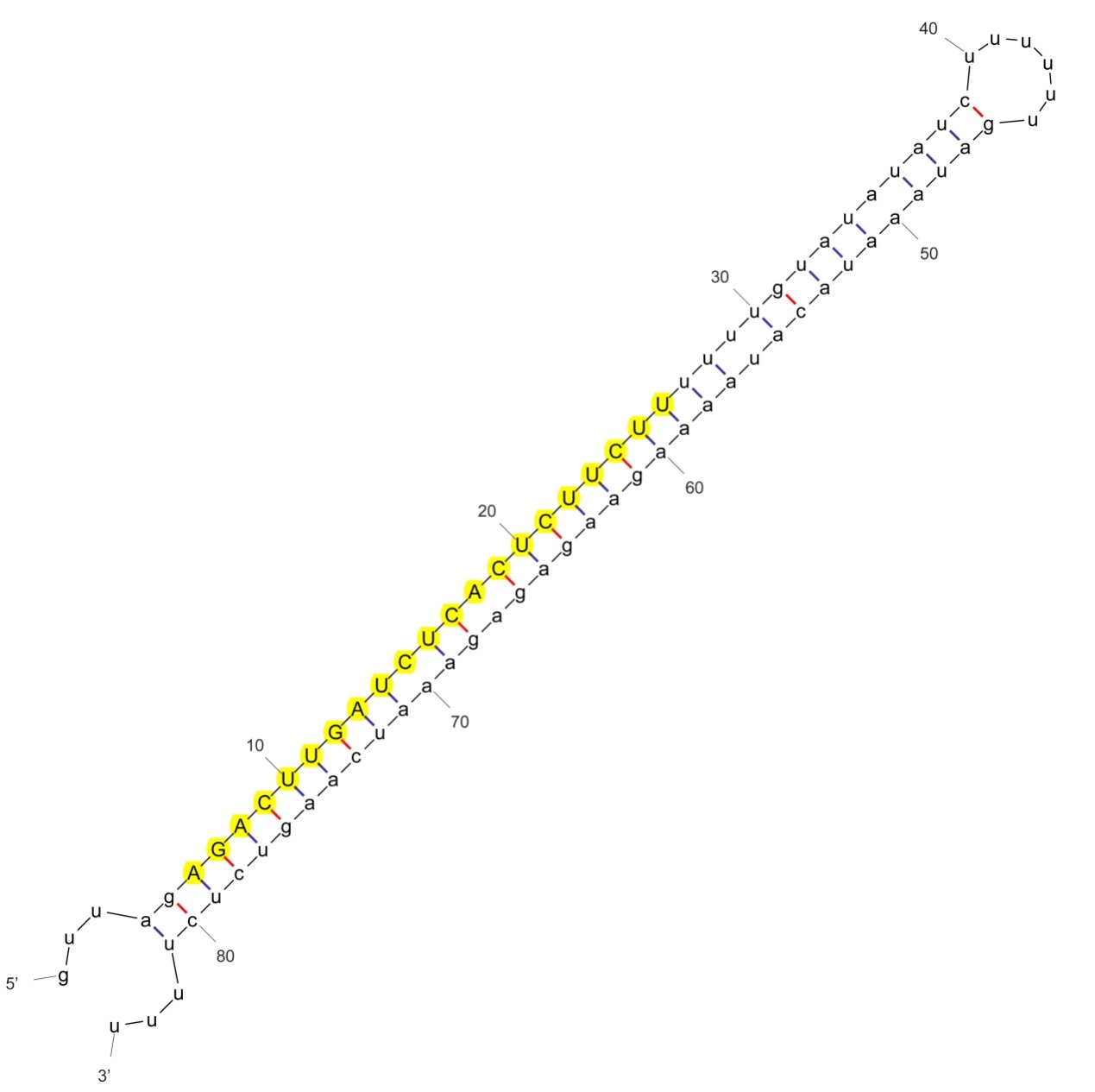


PC-26-5p


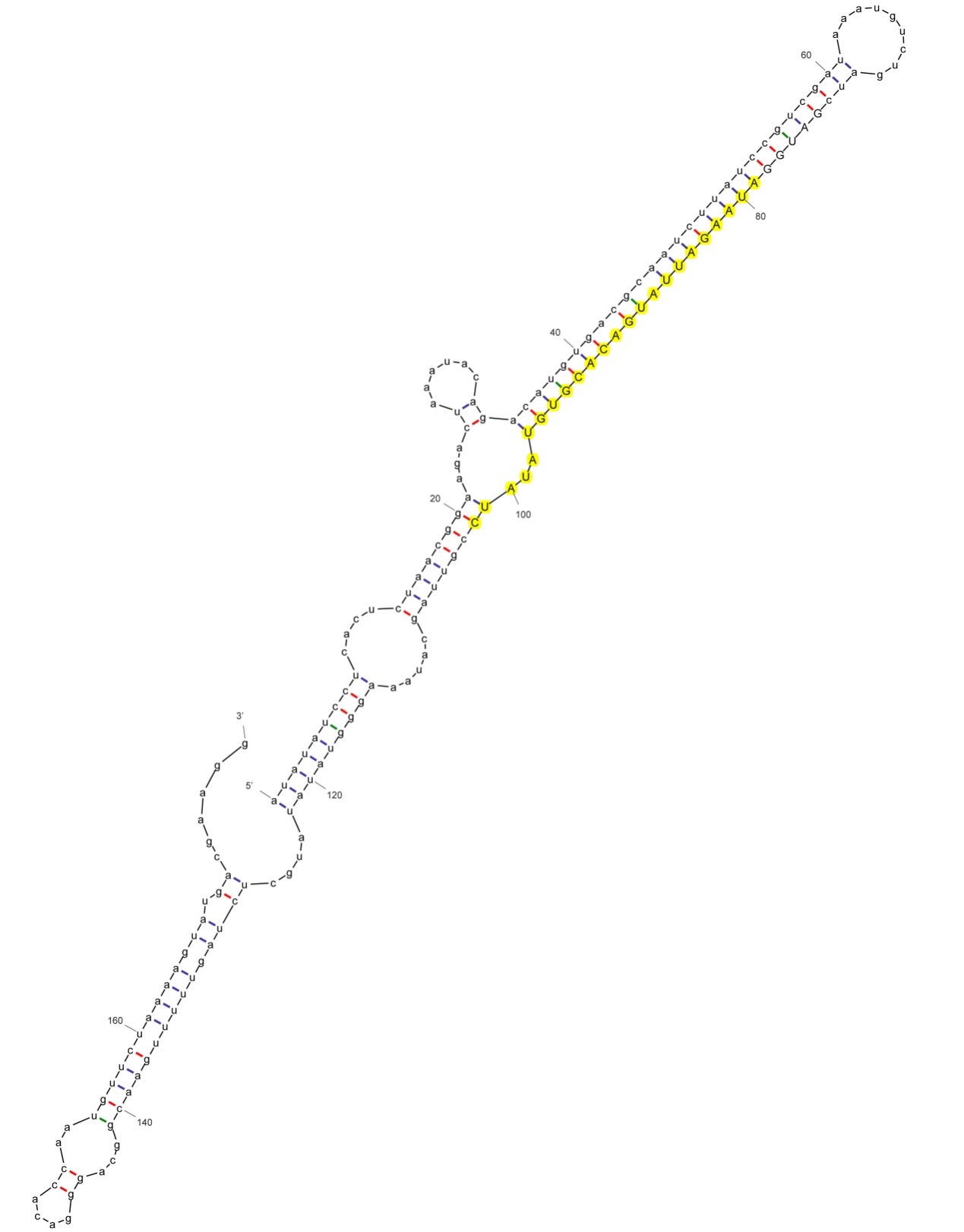


PC-27-3p


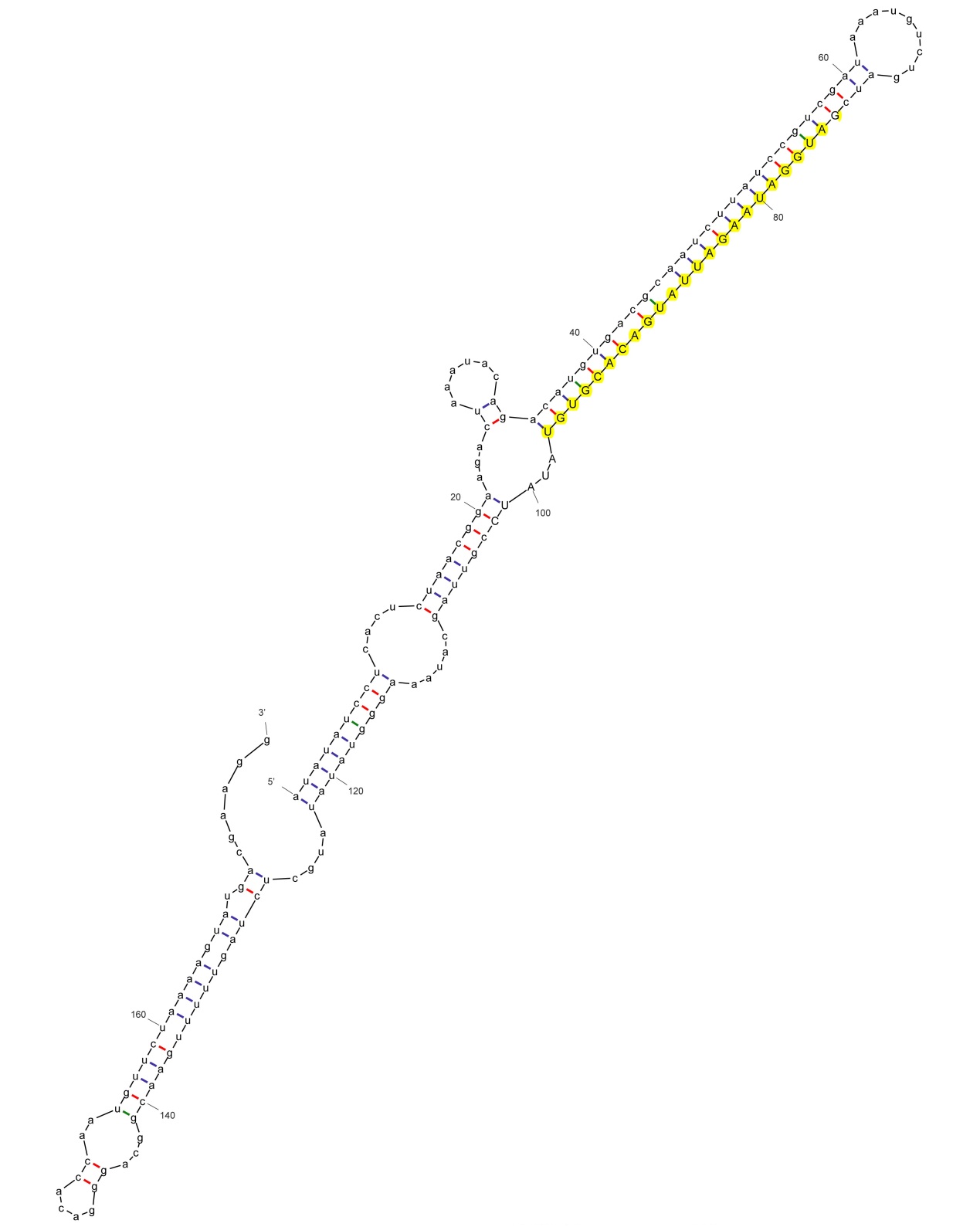


PC-27-5p


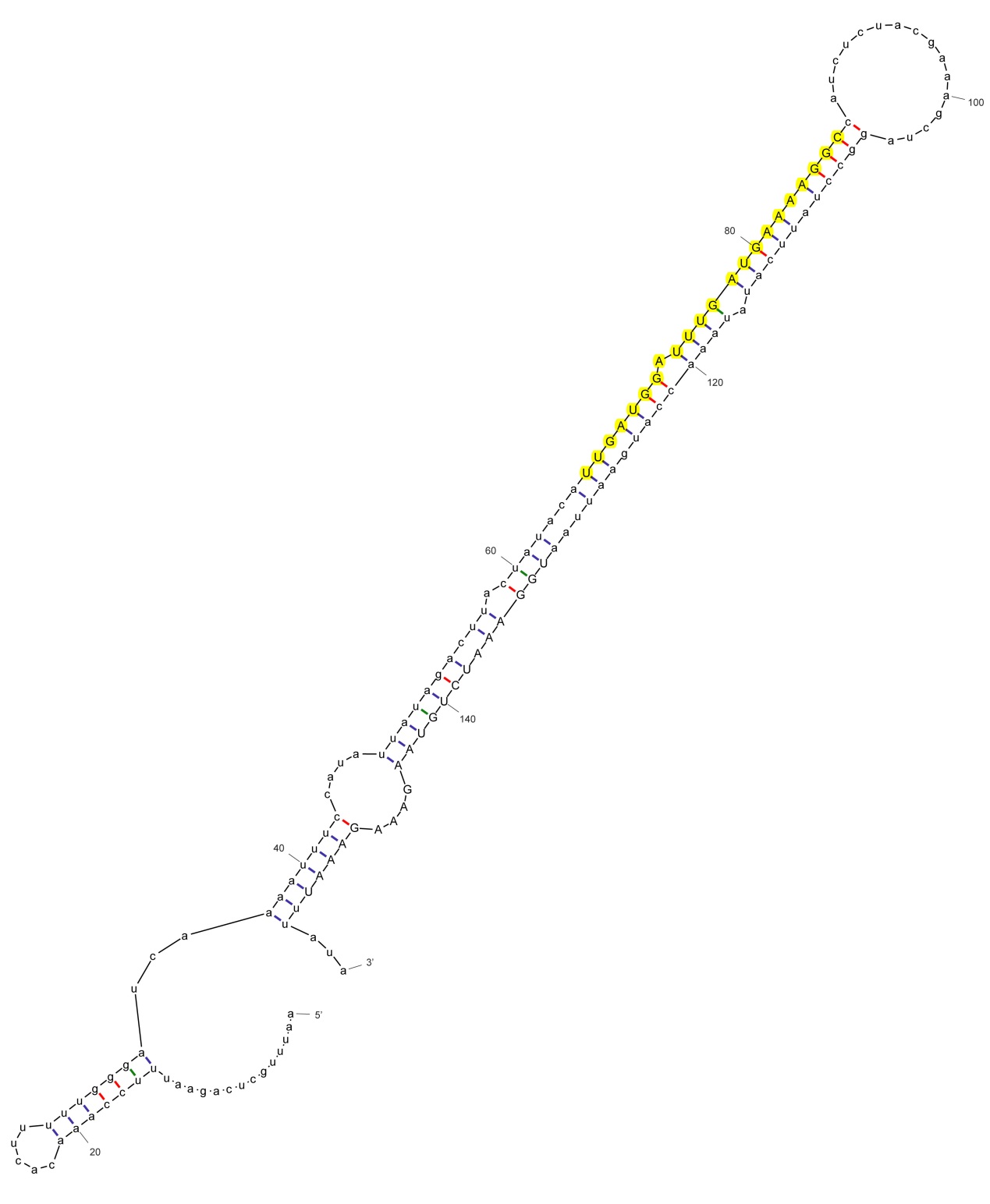


PC-28-5p


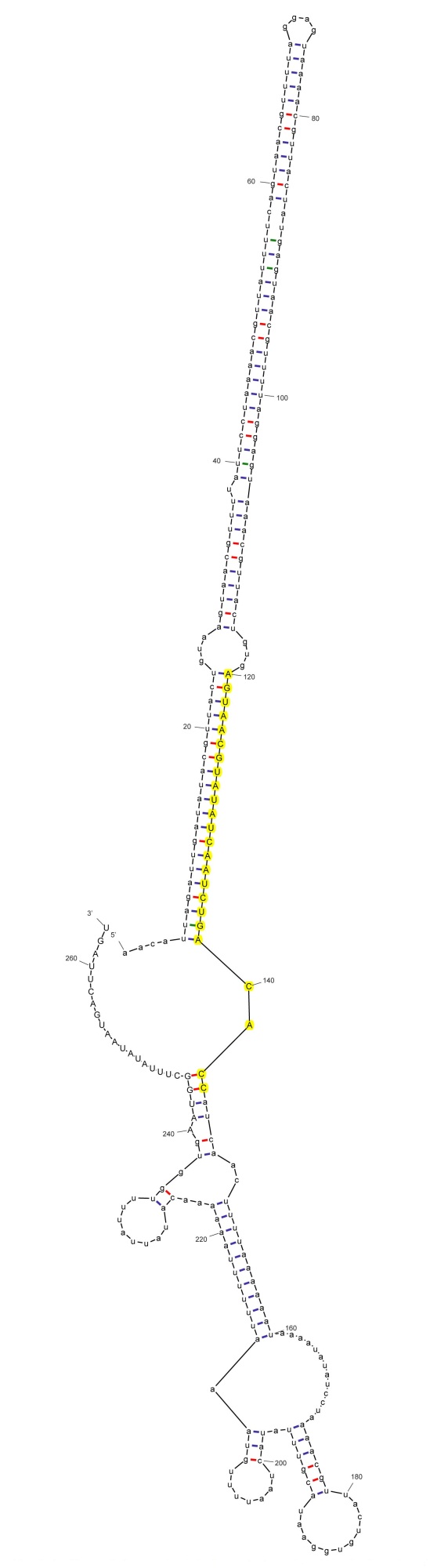


PC-29-5p


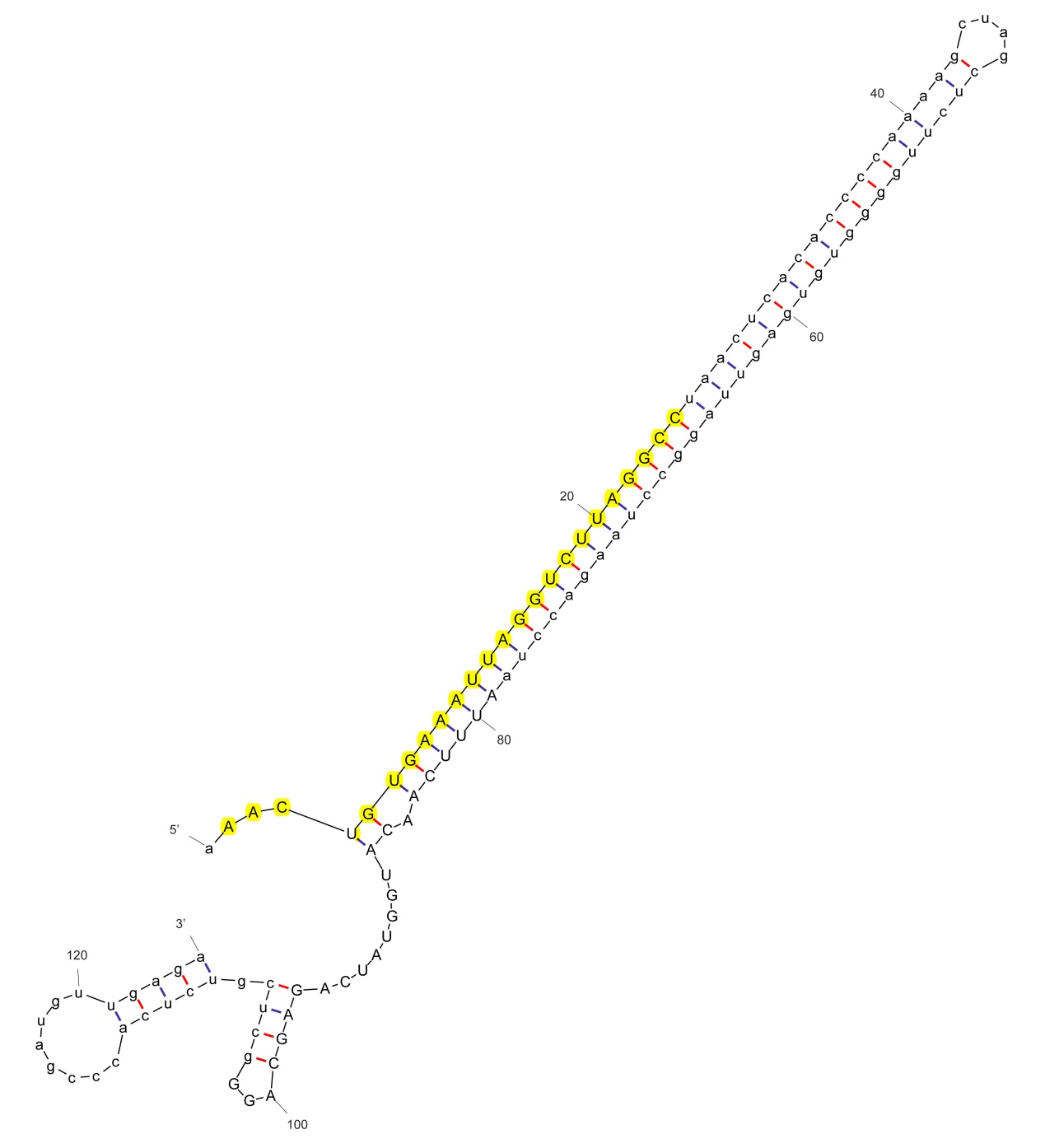


PC-30-5p


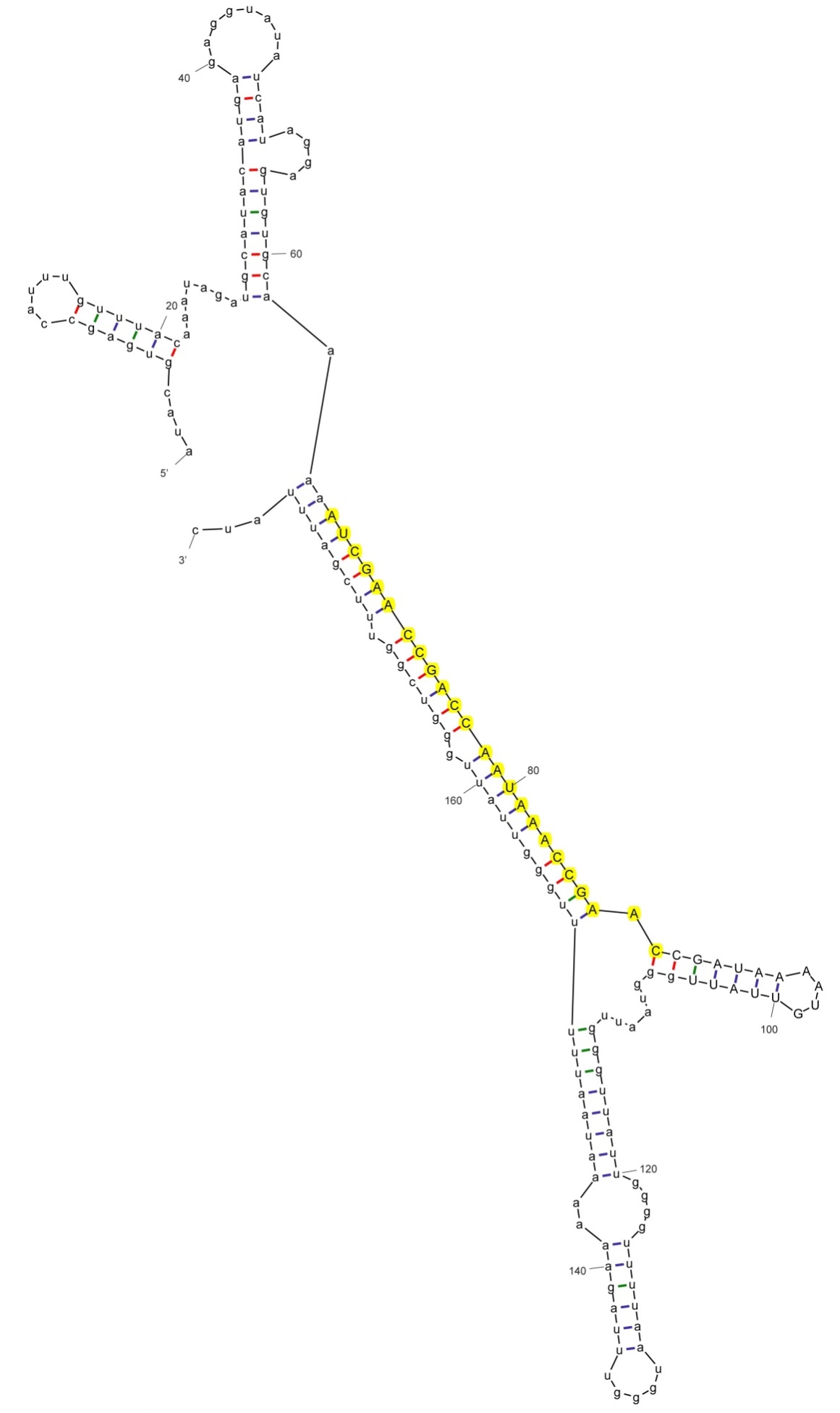


PC-31-5p


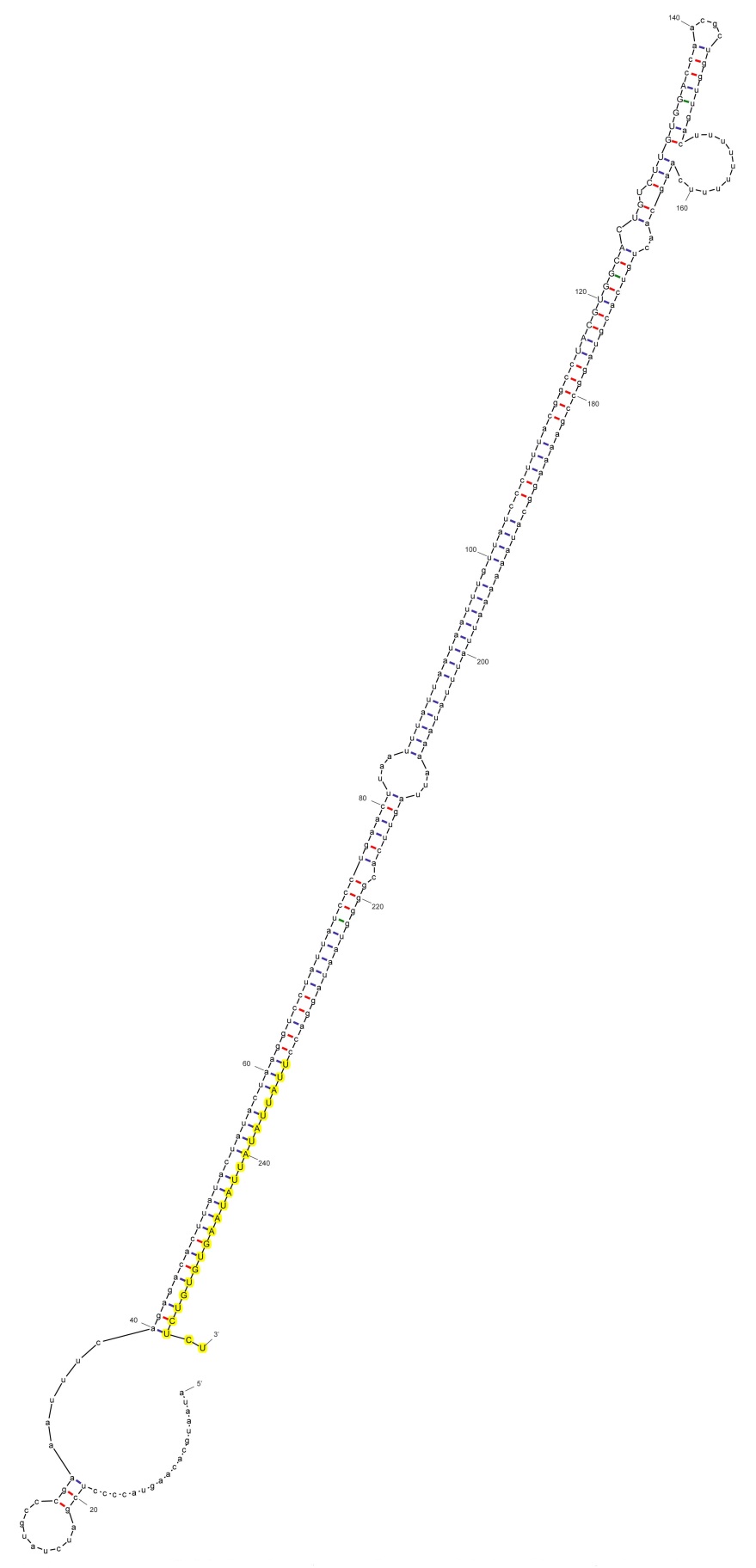


PC-32-3p


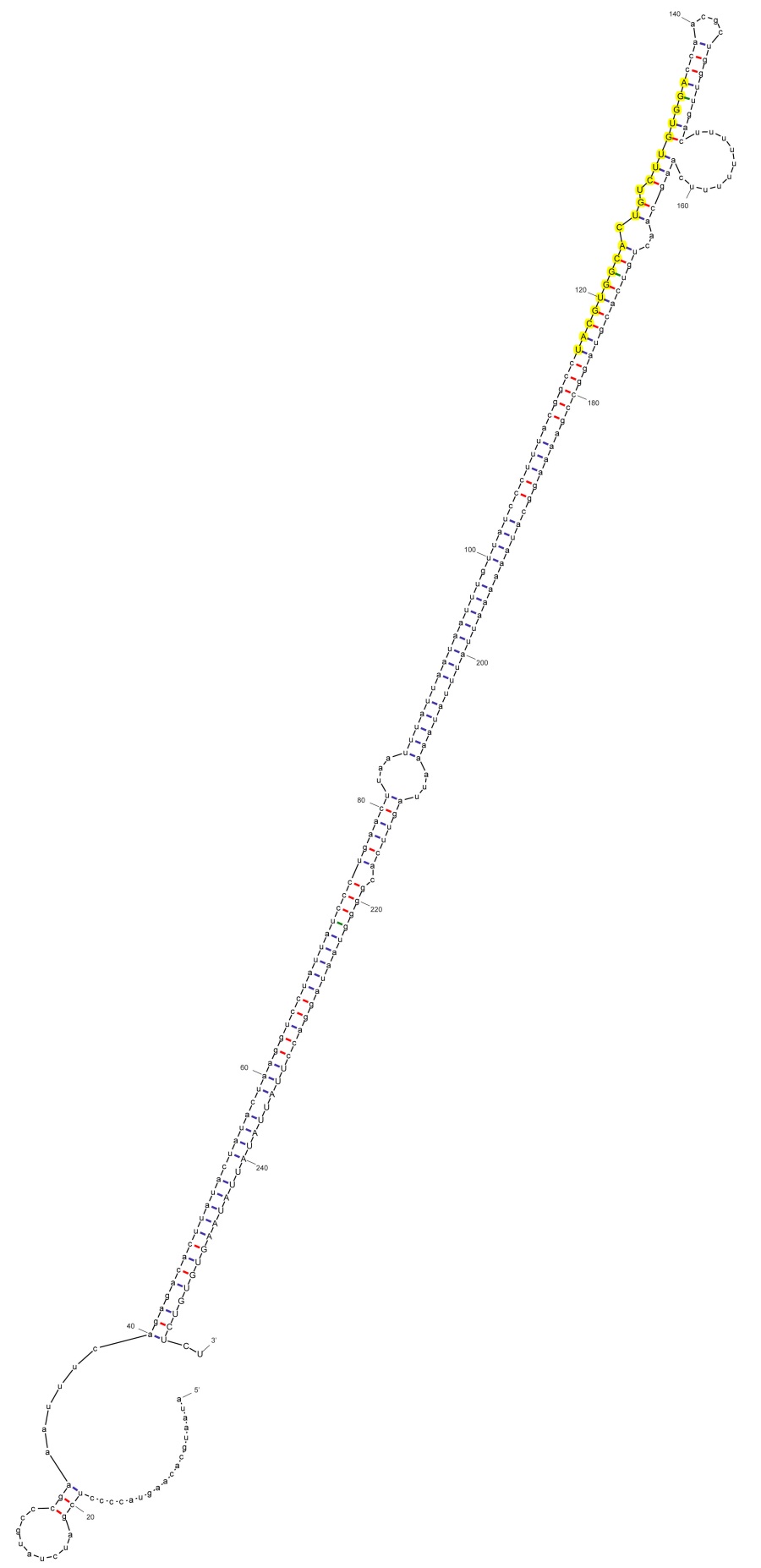


PC-32-5p


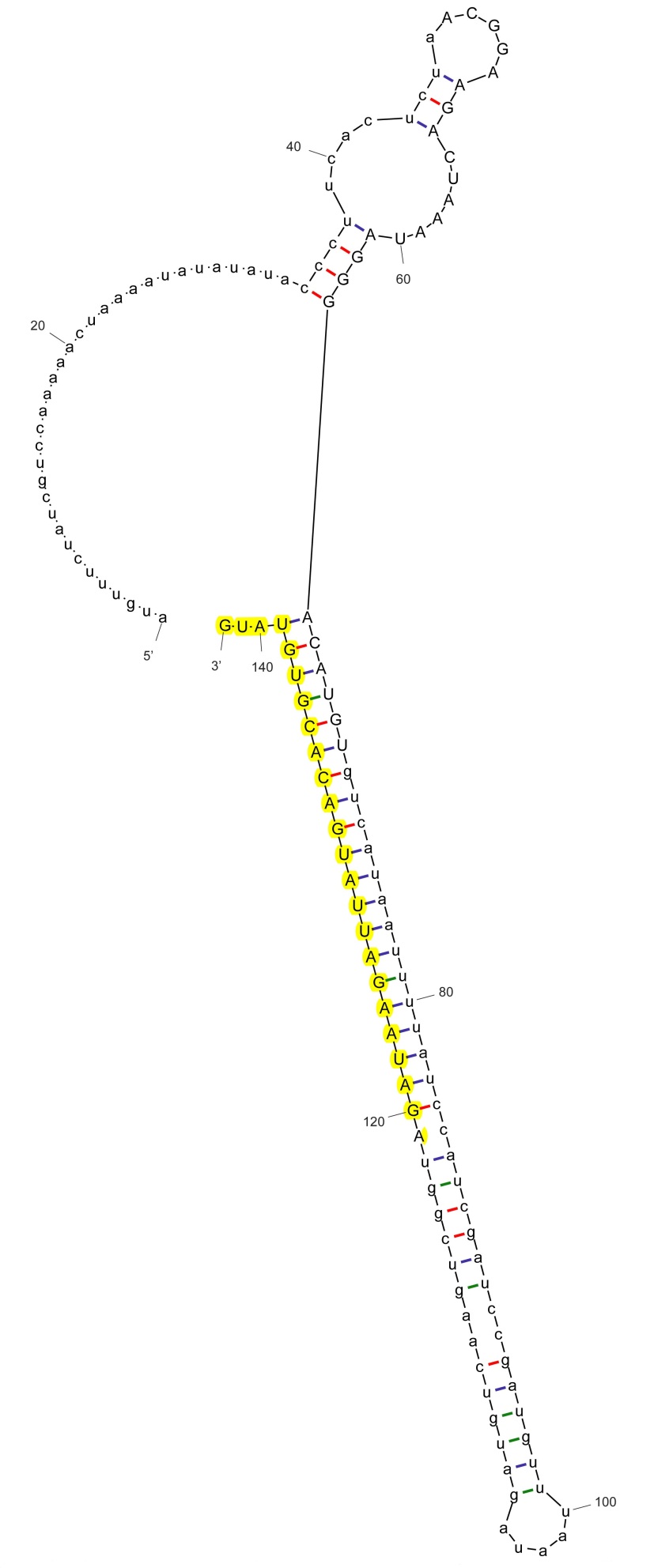


PC-33-3p


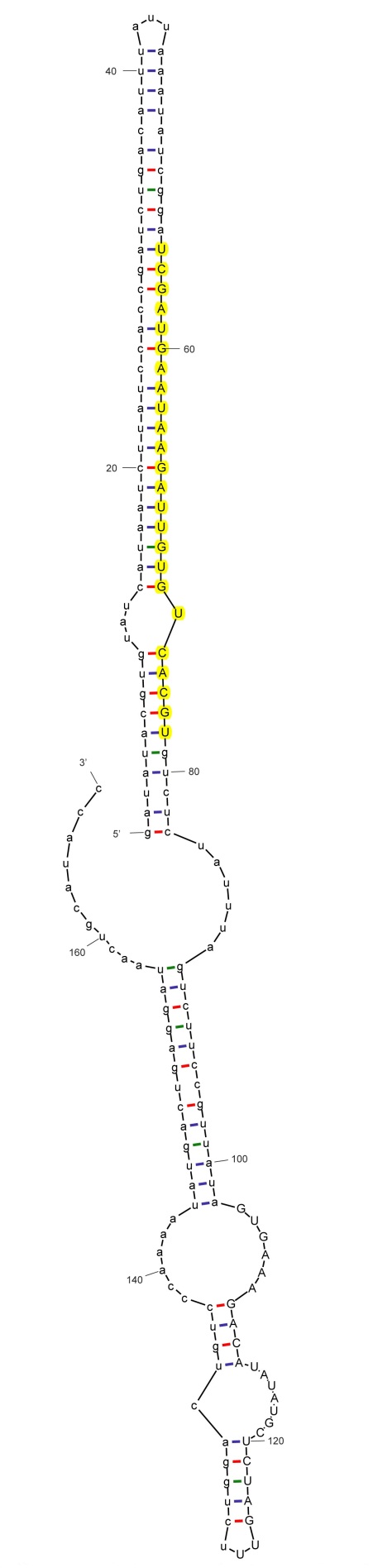


PC-34-5p


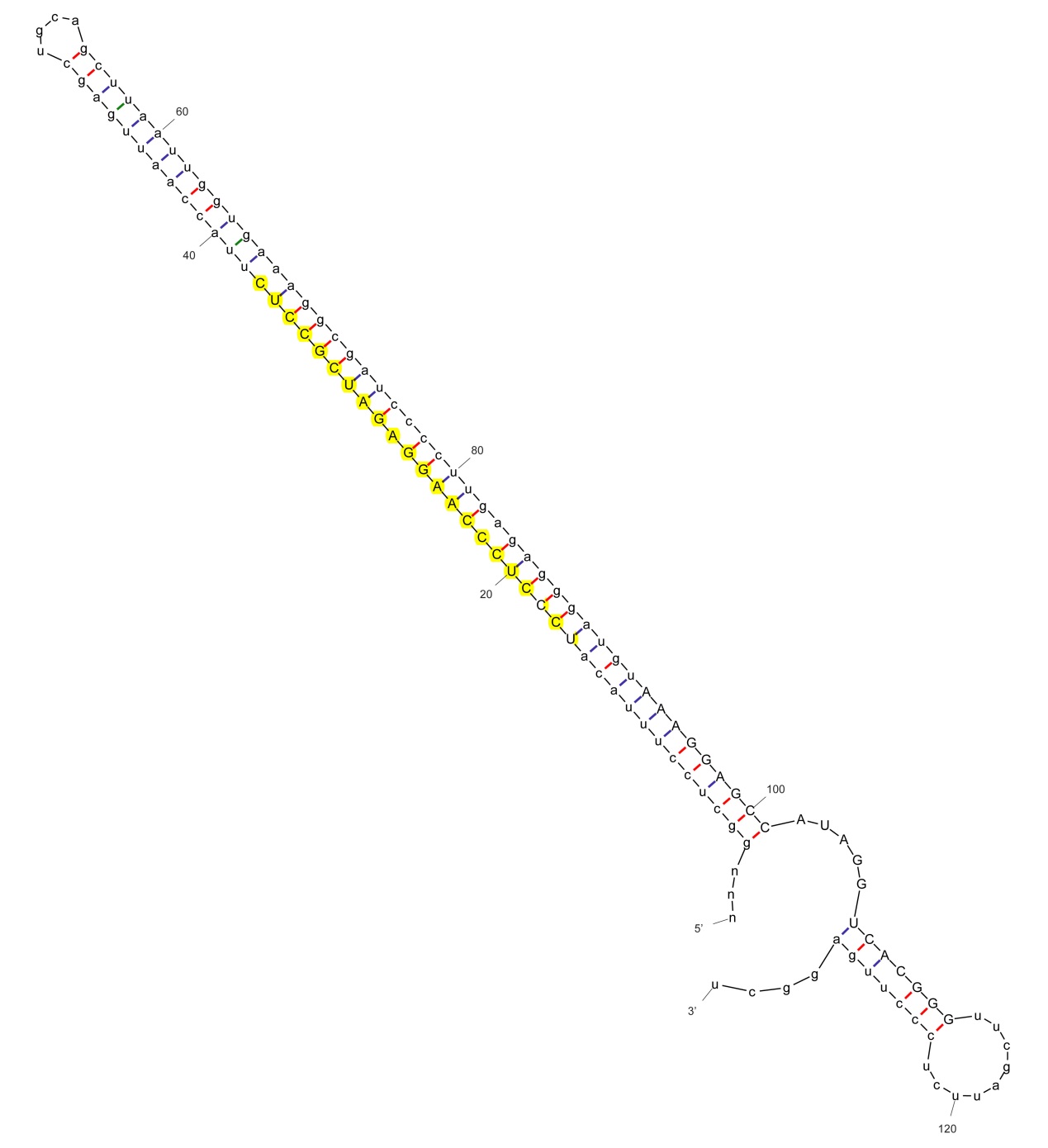


PC-35-5p

PC-36-5p


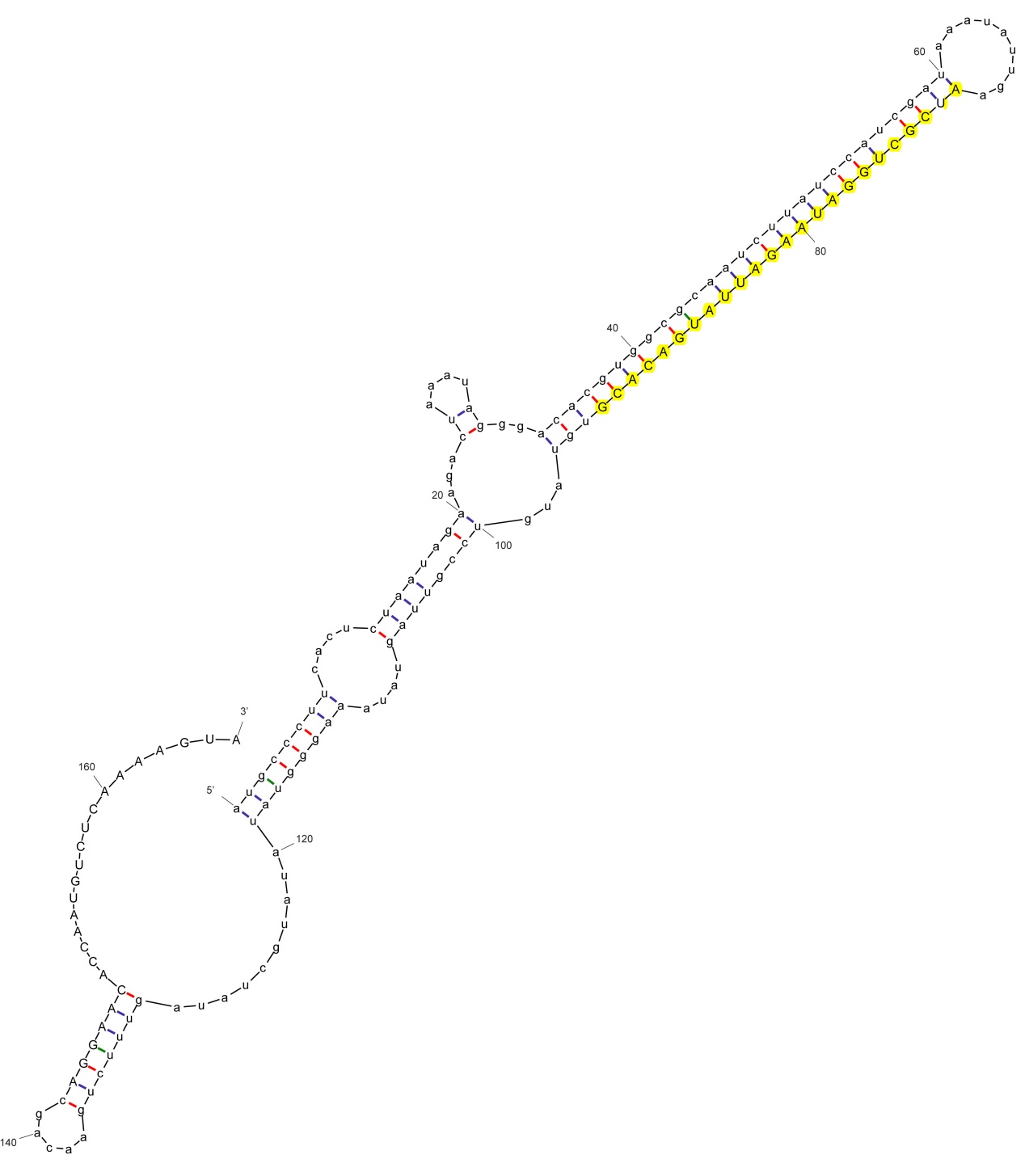


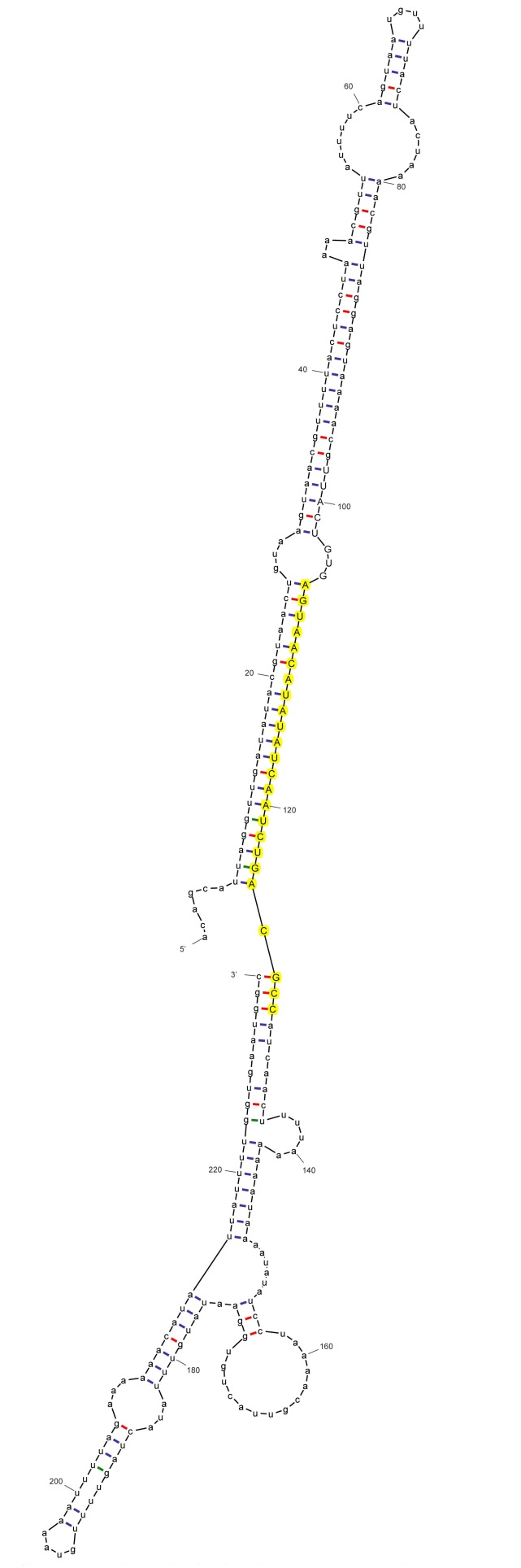


PC-37-3p


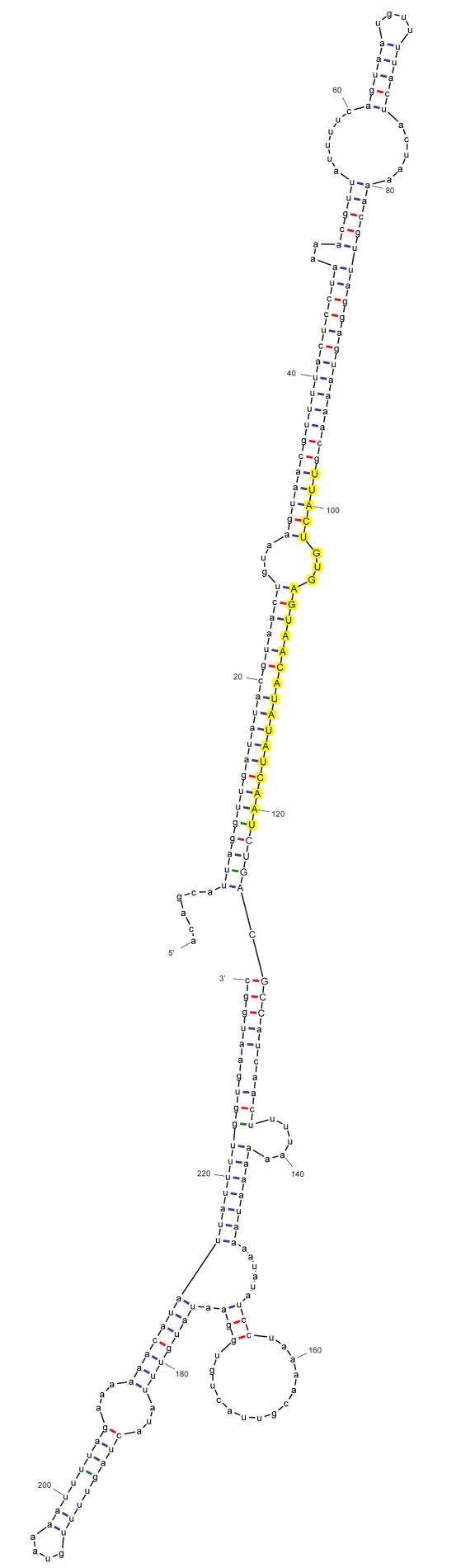


PC-37-5p


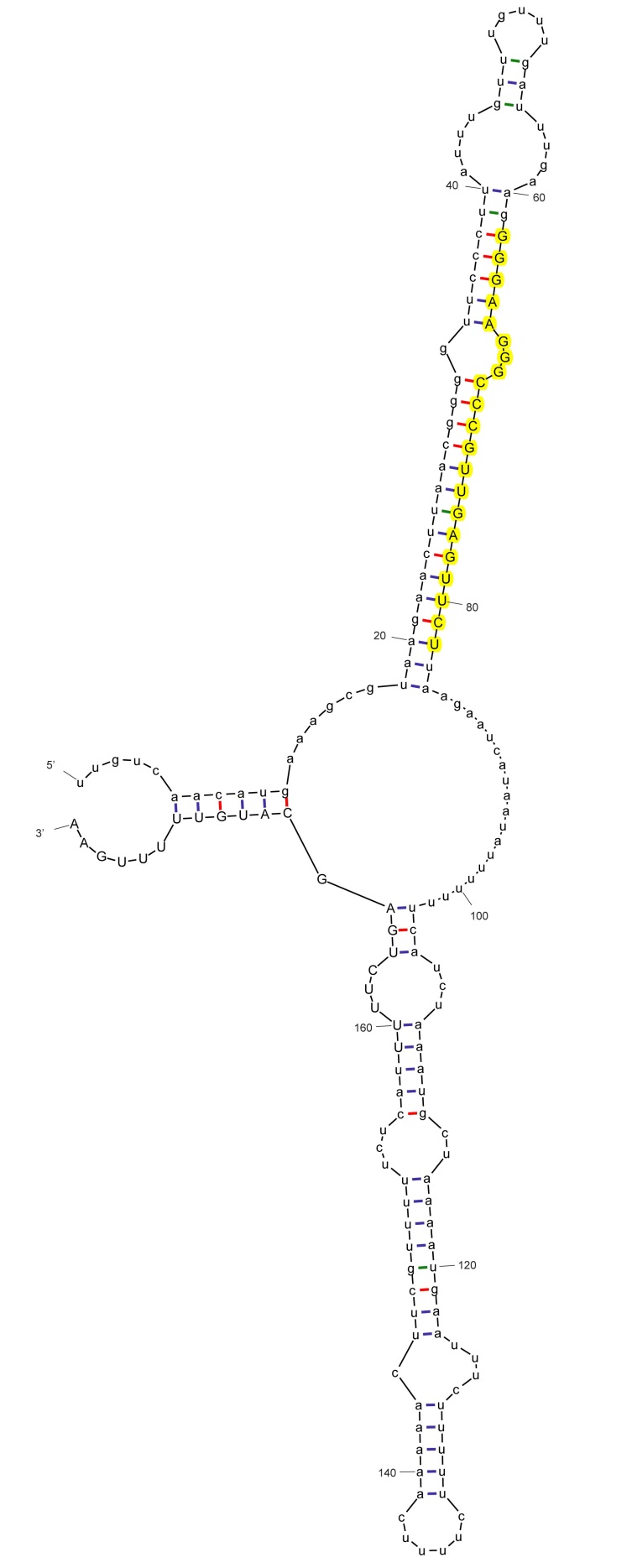


PC-38-5p


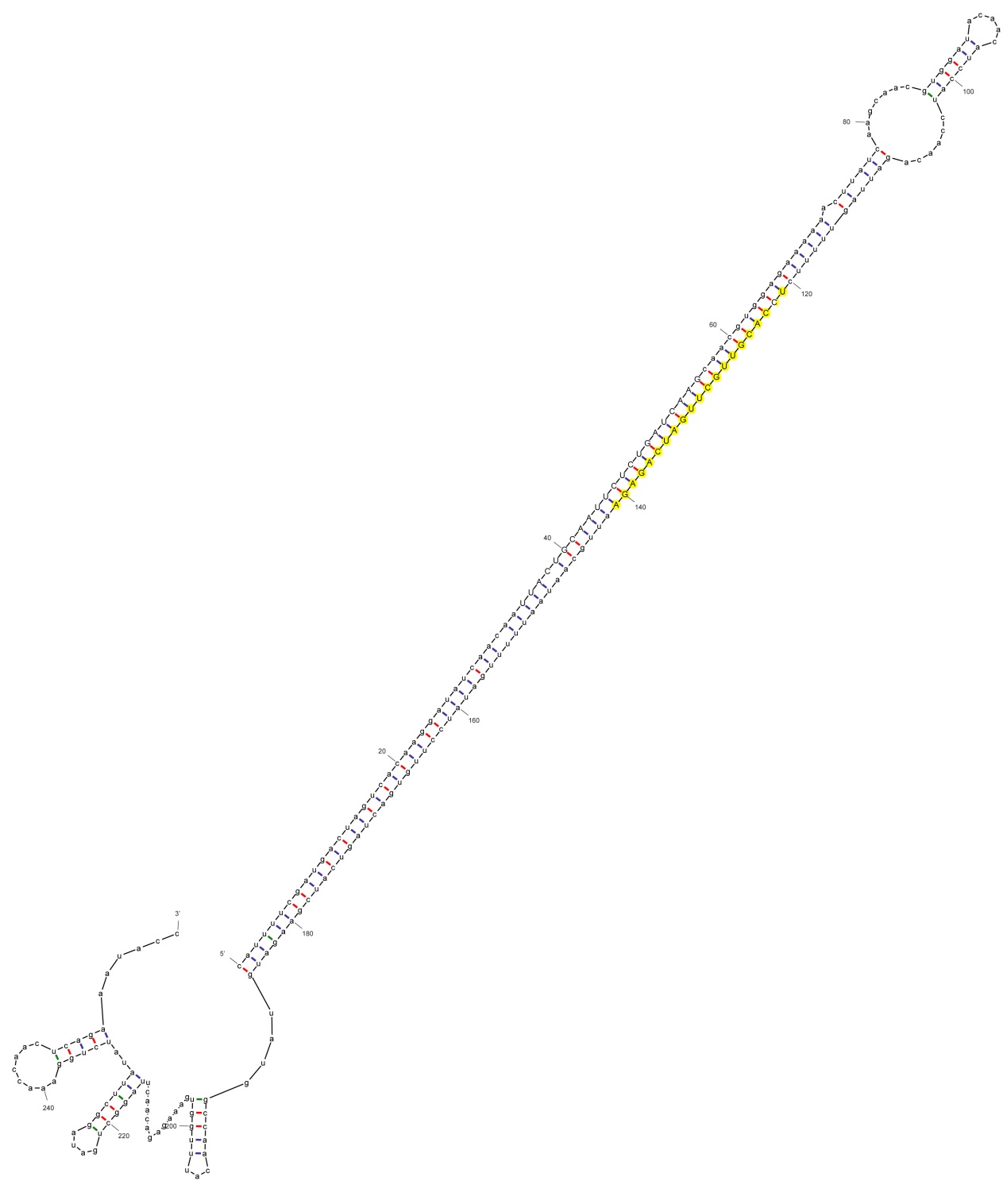


PC-39-3p


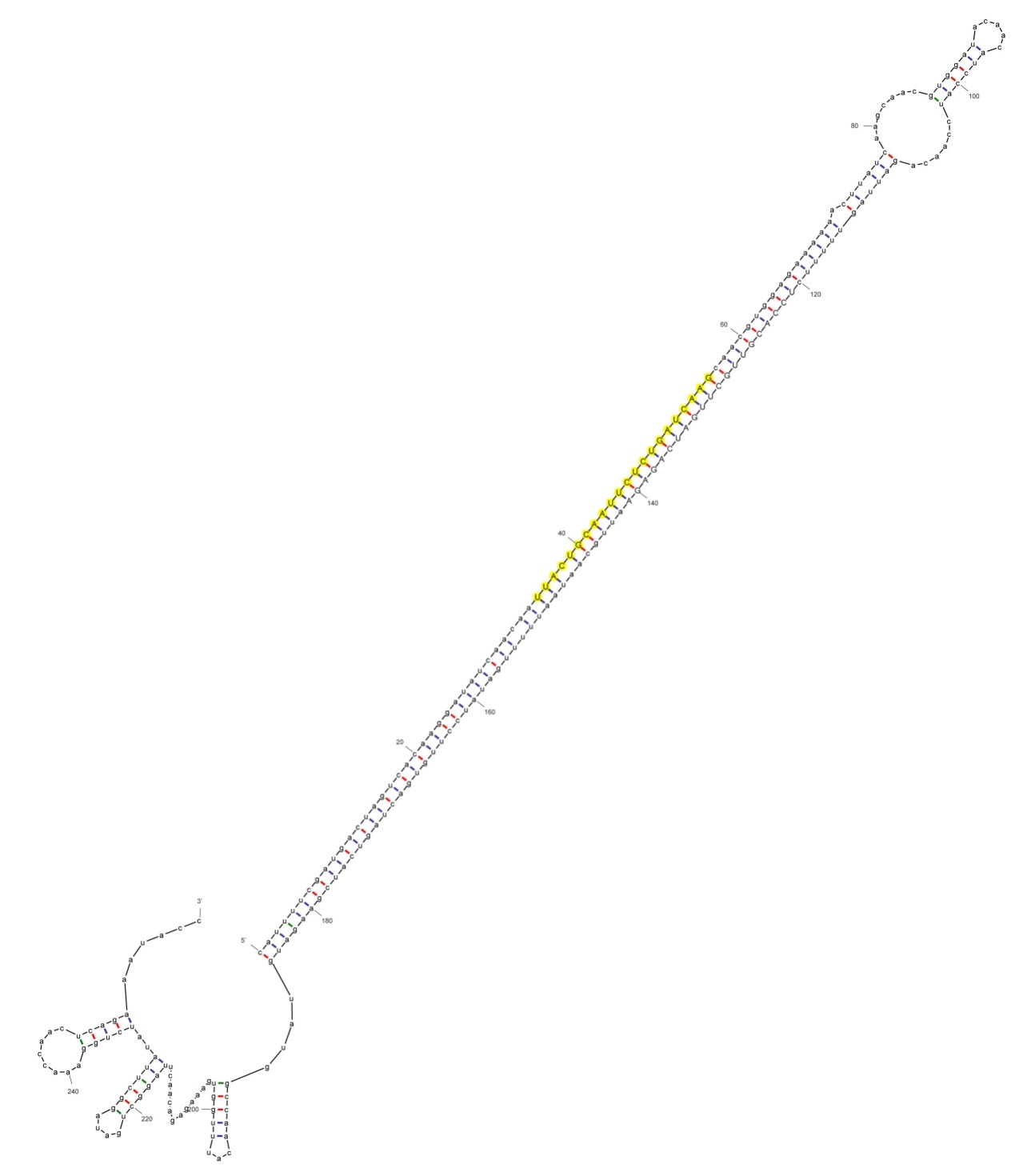


PC-39-5p


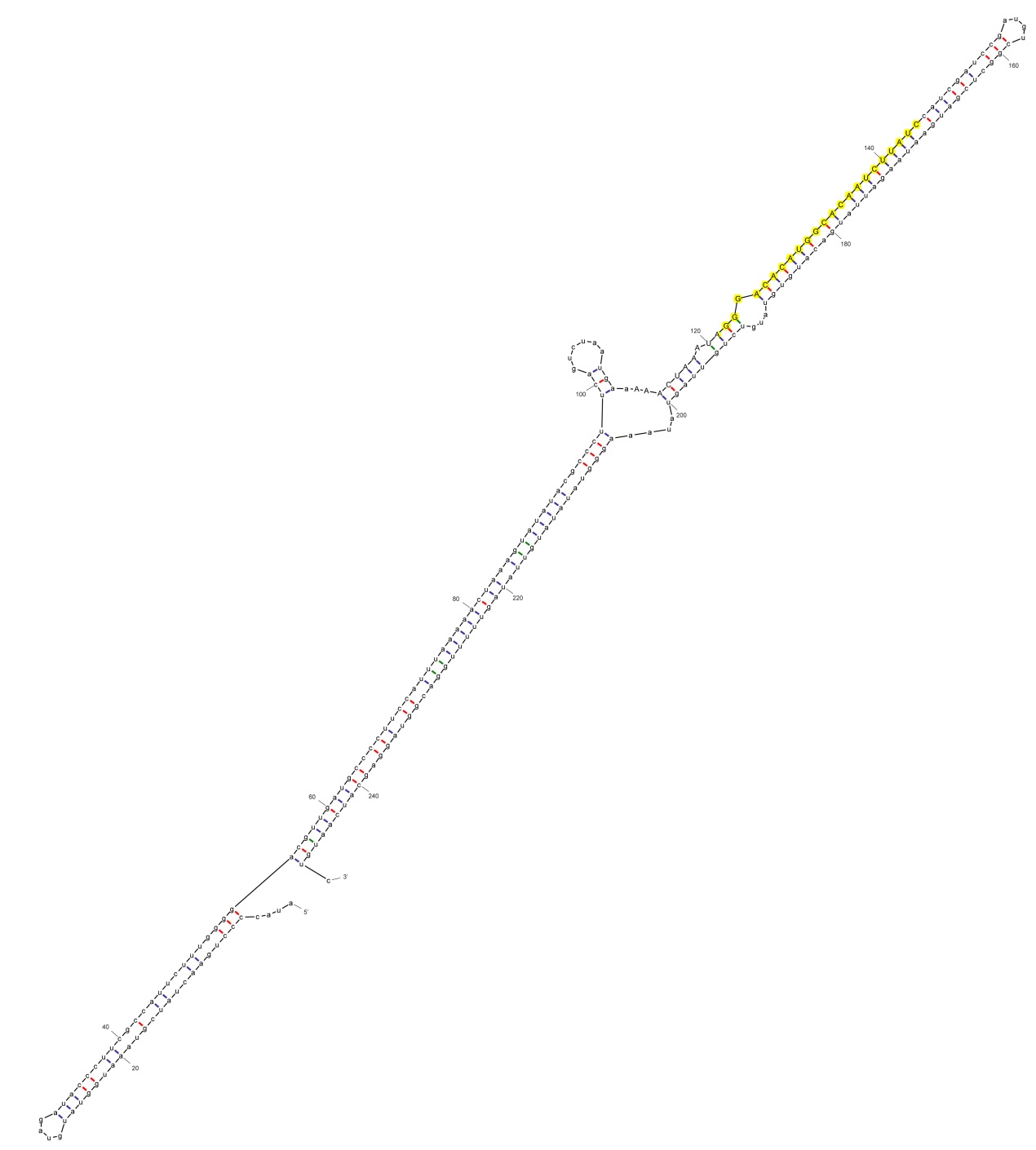


PC-40-3p


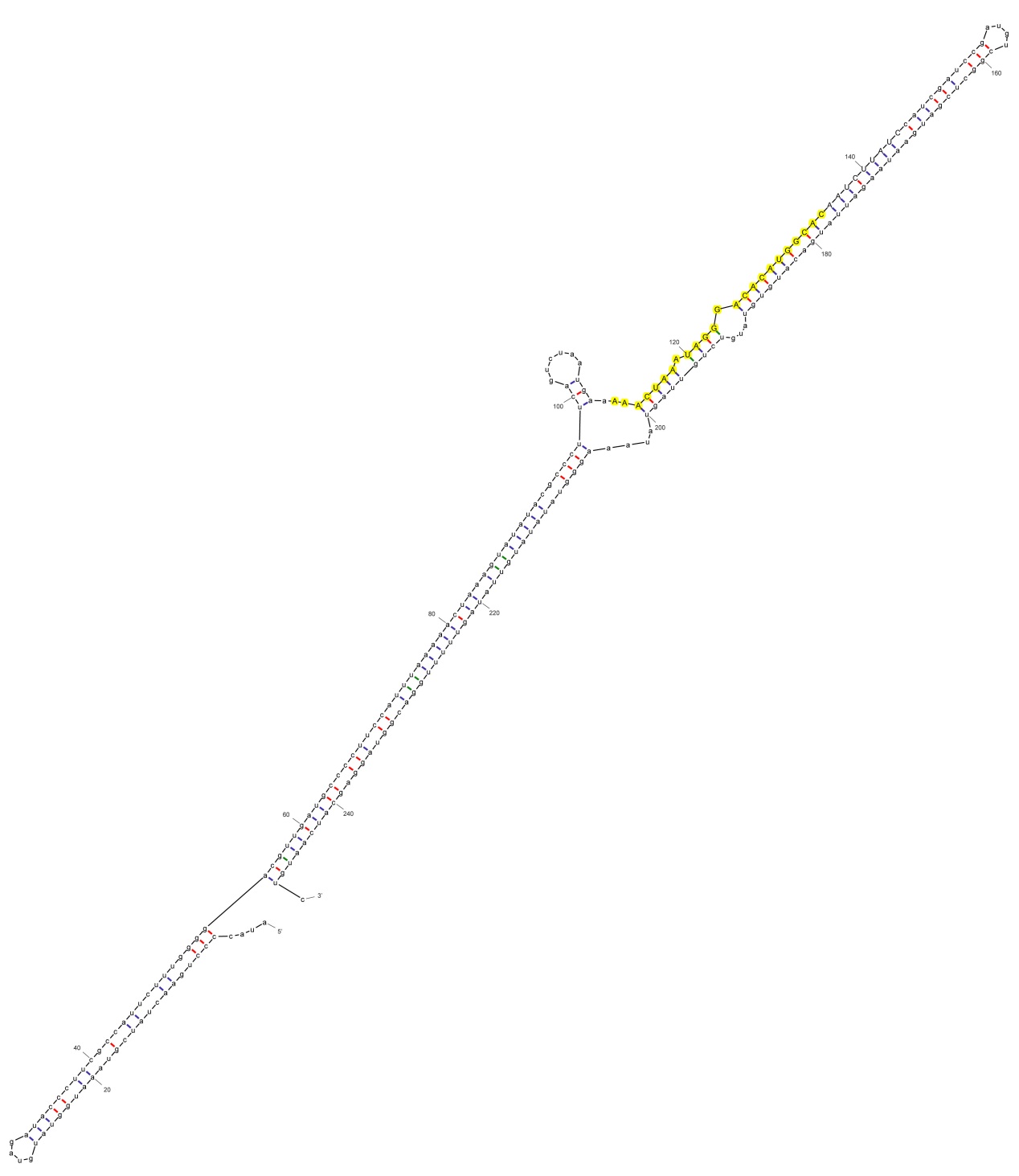


PC-40-5p


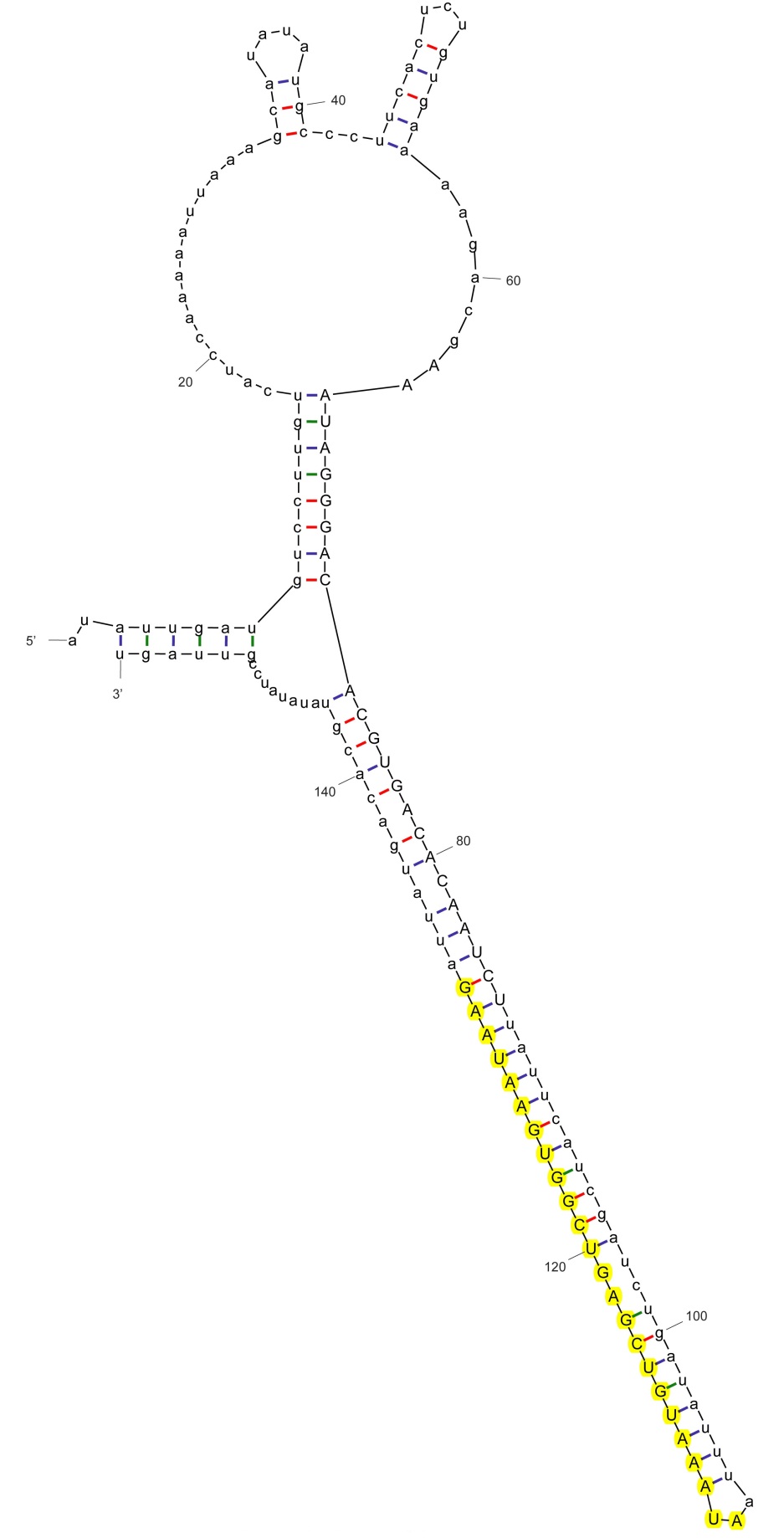


PC-41-3p


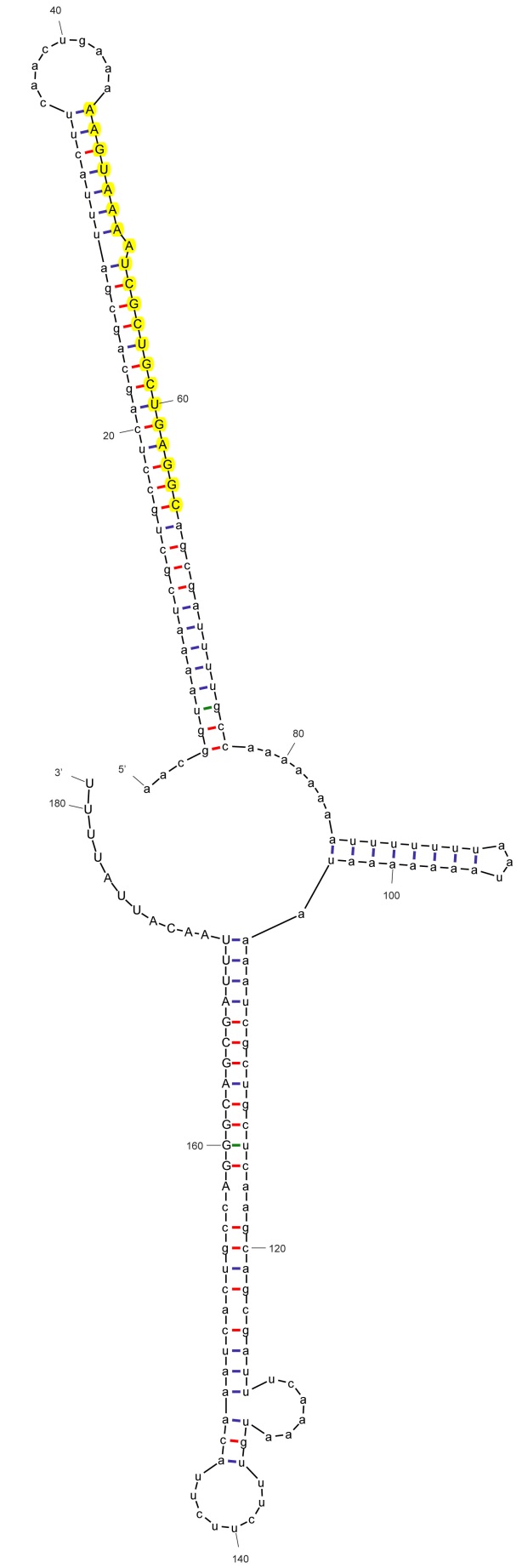


PC-42-5p


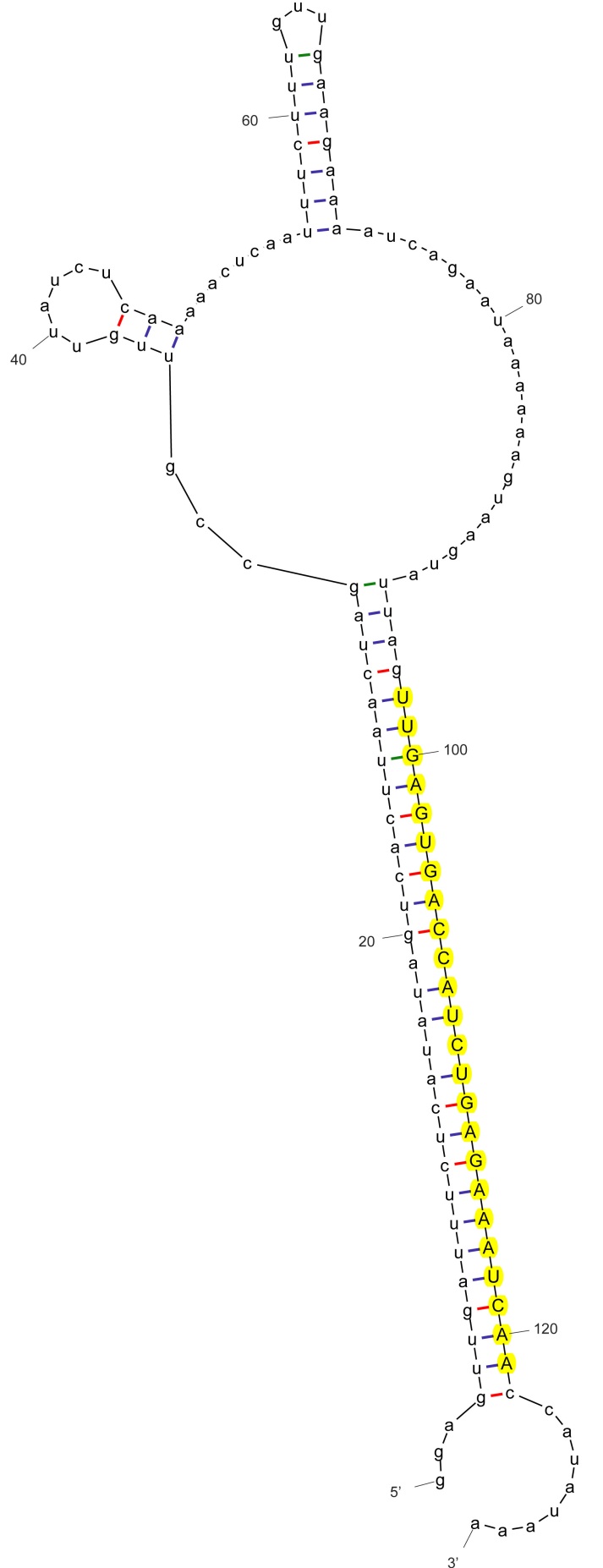


PC-43-3p


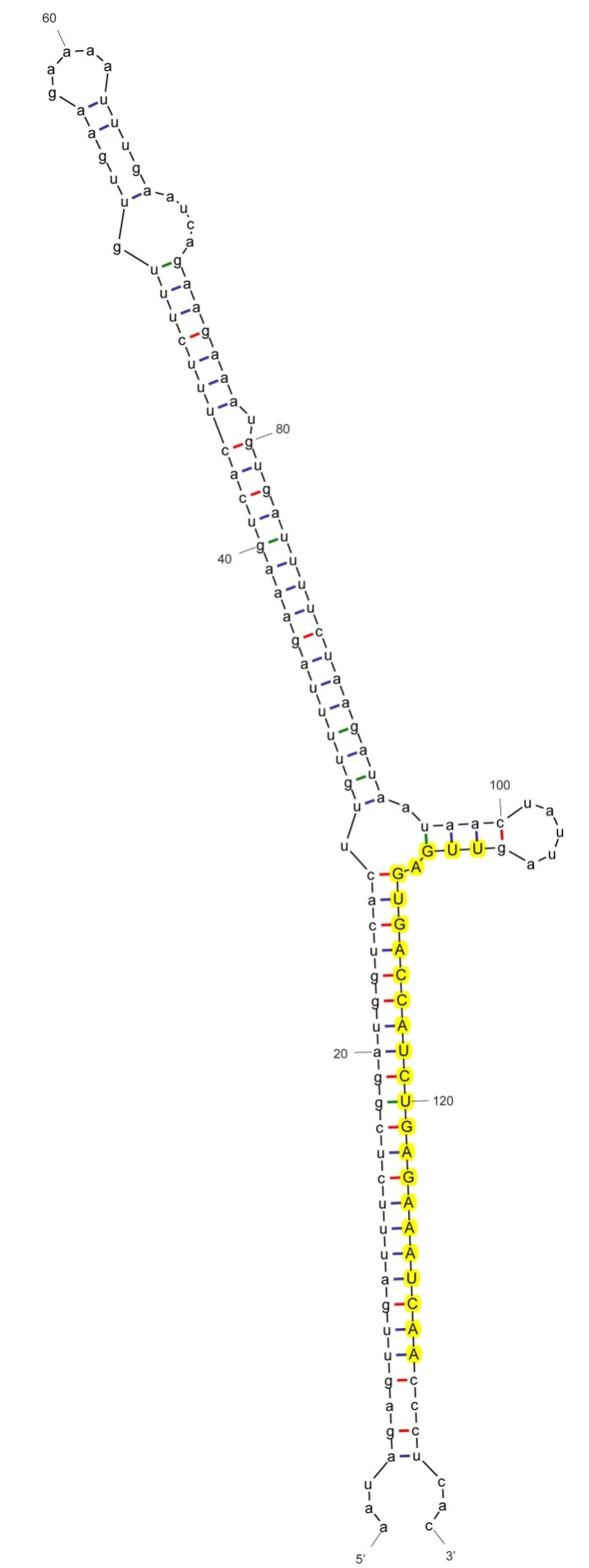


PC-44-3p


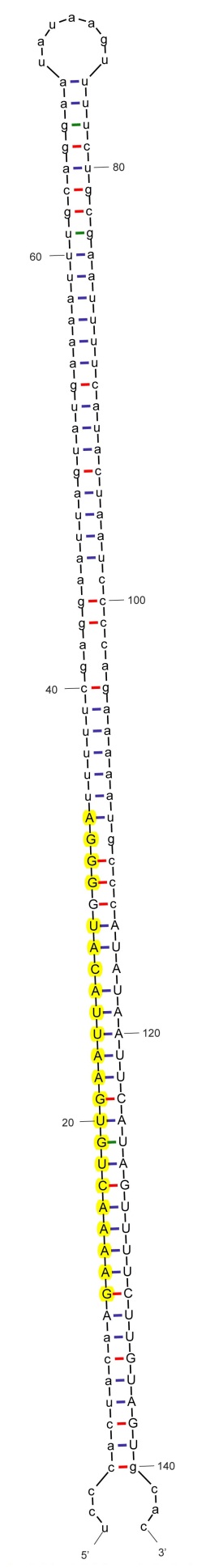


PC-45-5p


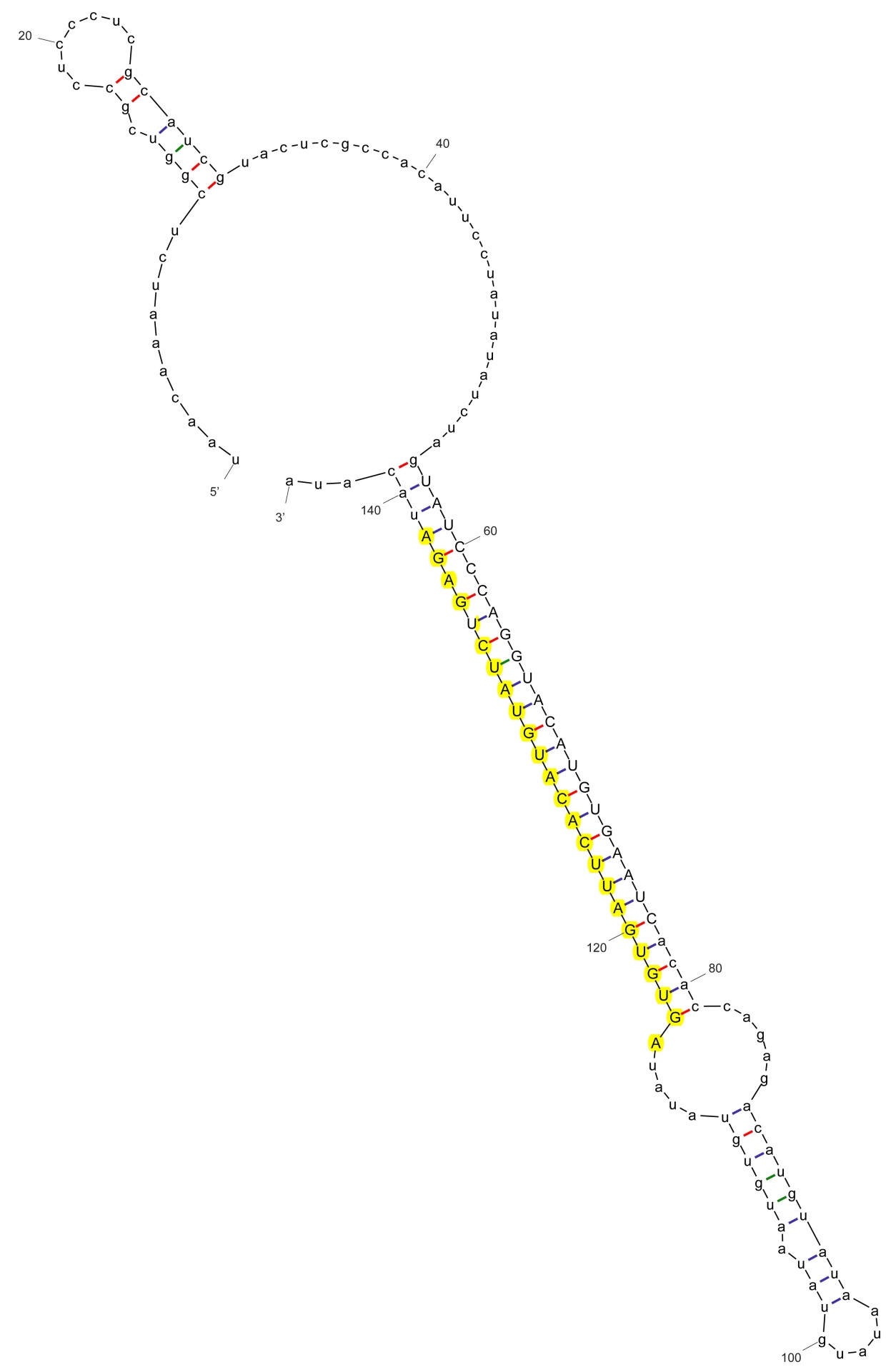


PC-46-3p


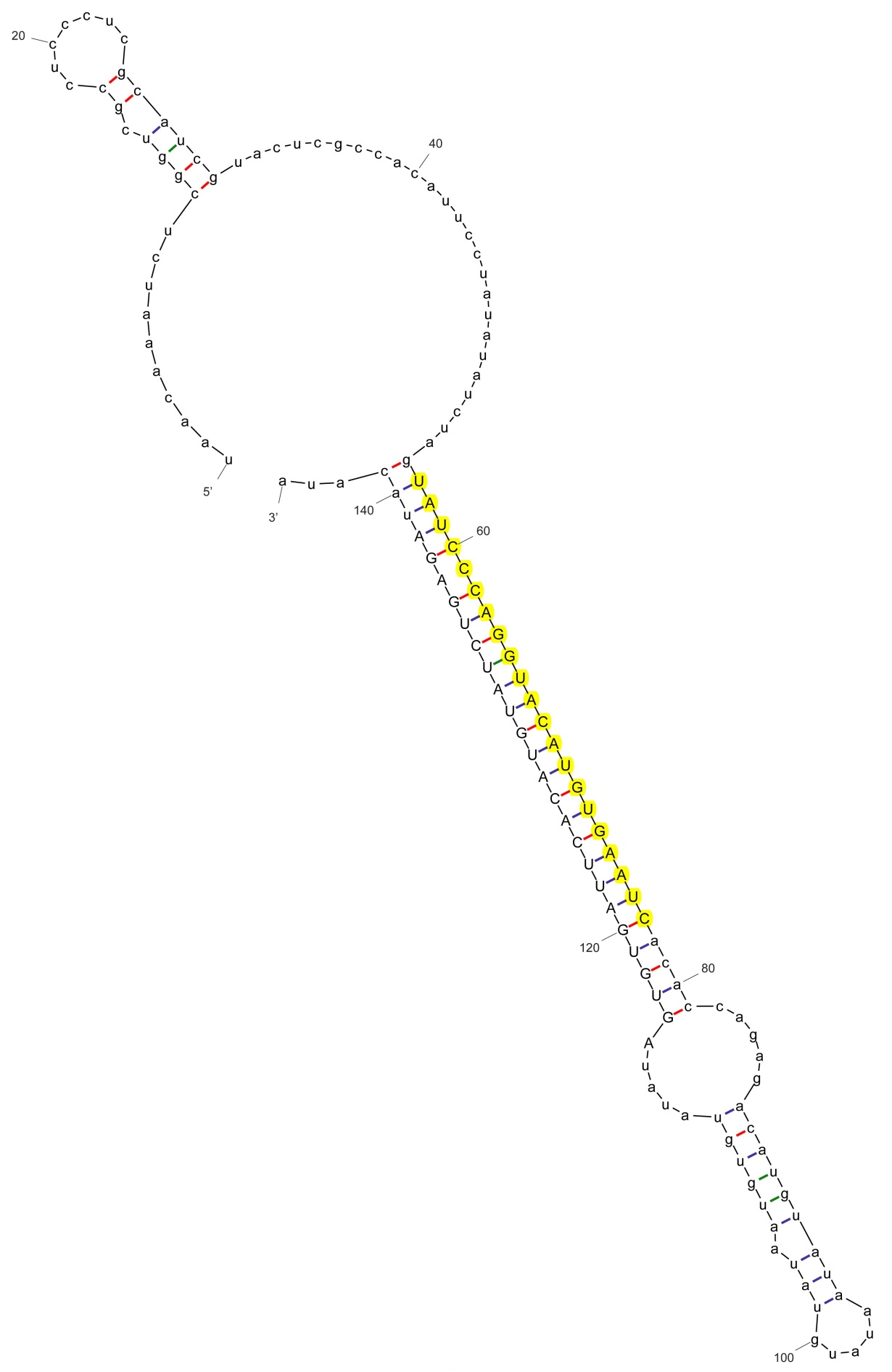


PC-46-5p


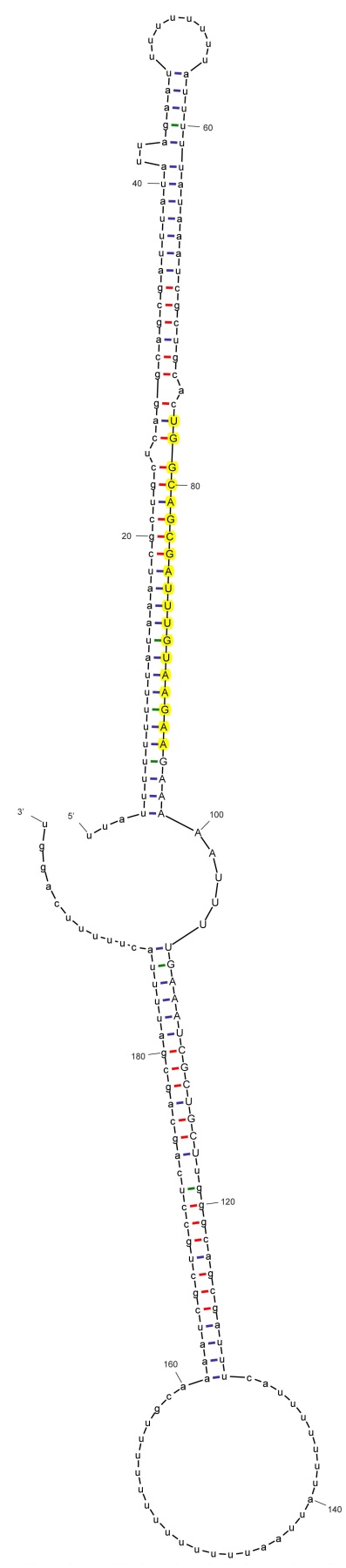


PC-47-5p


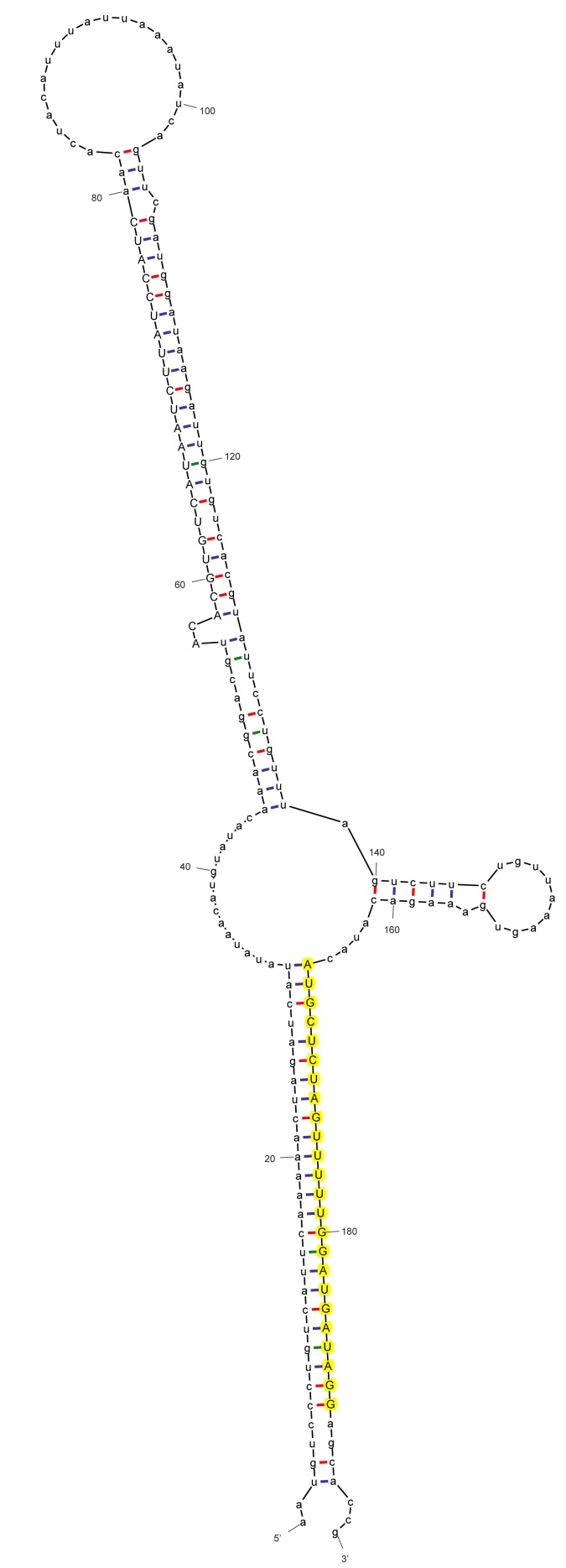


PC-48-3p


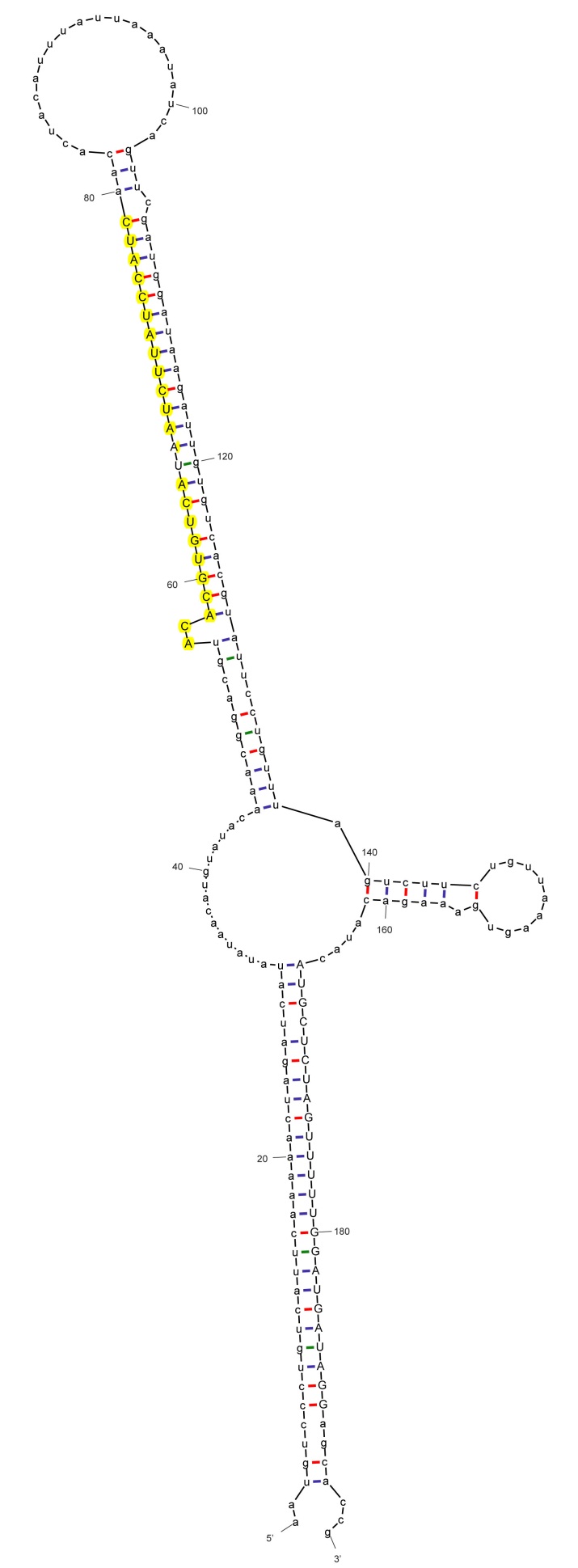


PC-48-5p


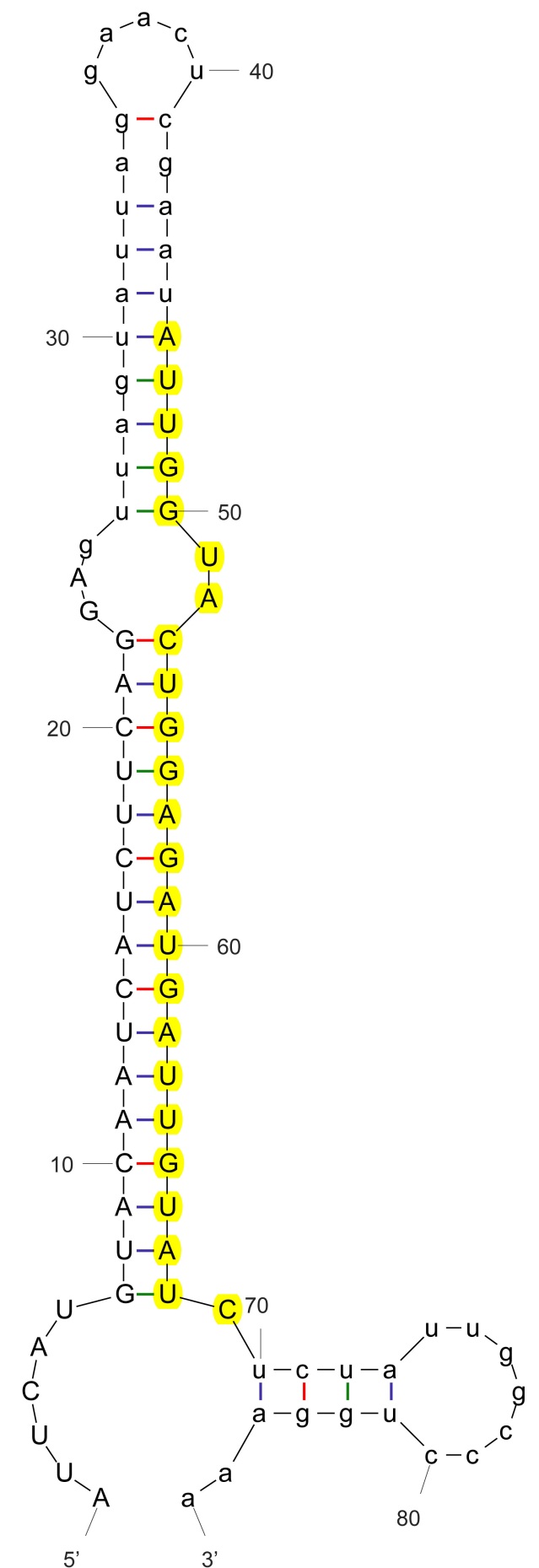


PC-49-3p


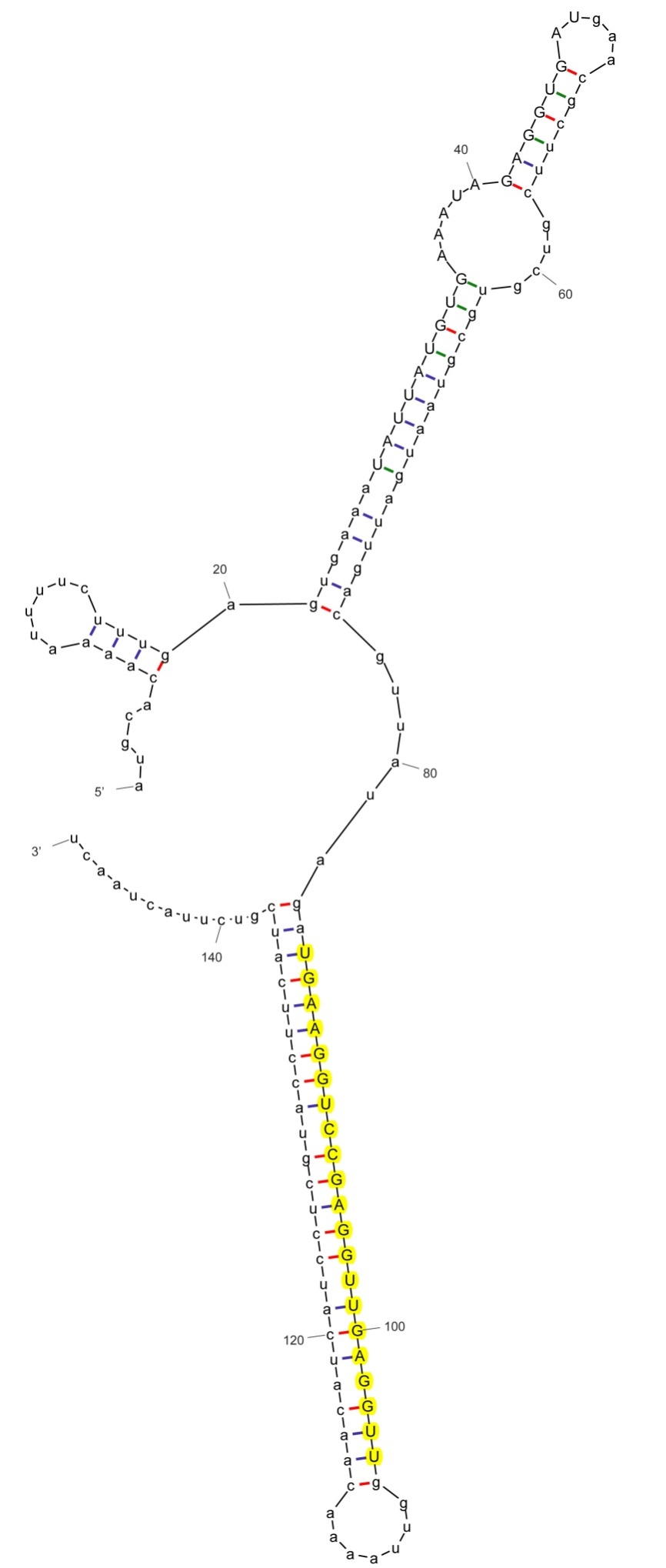


PC-50-3p


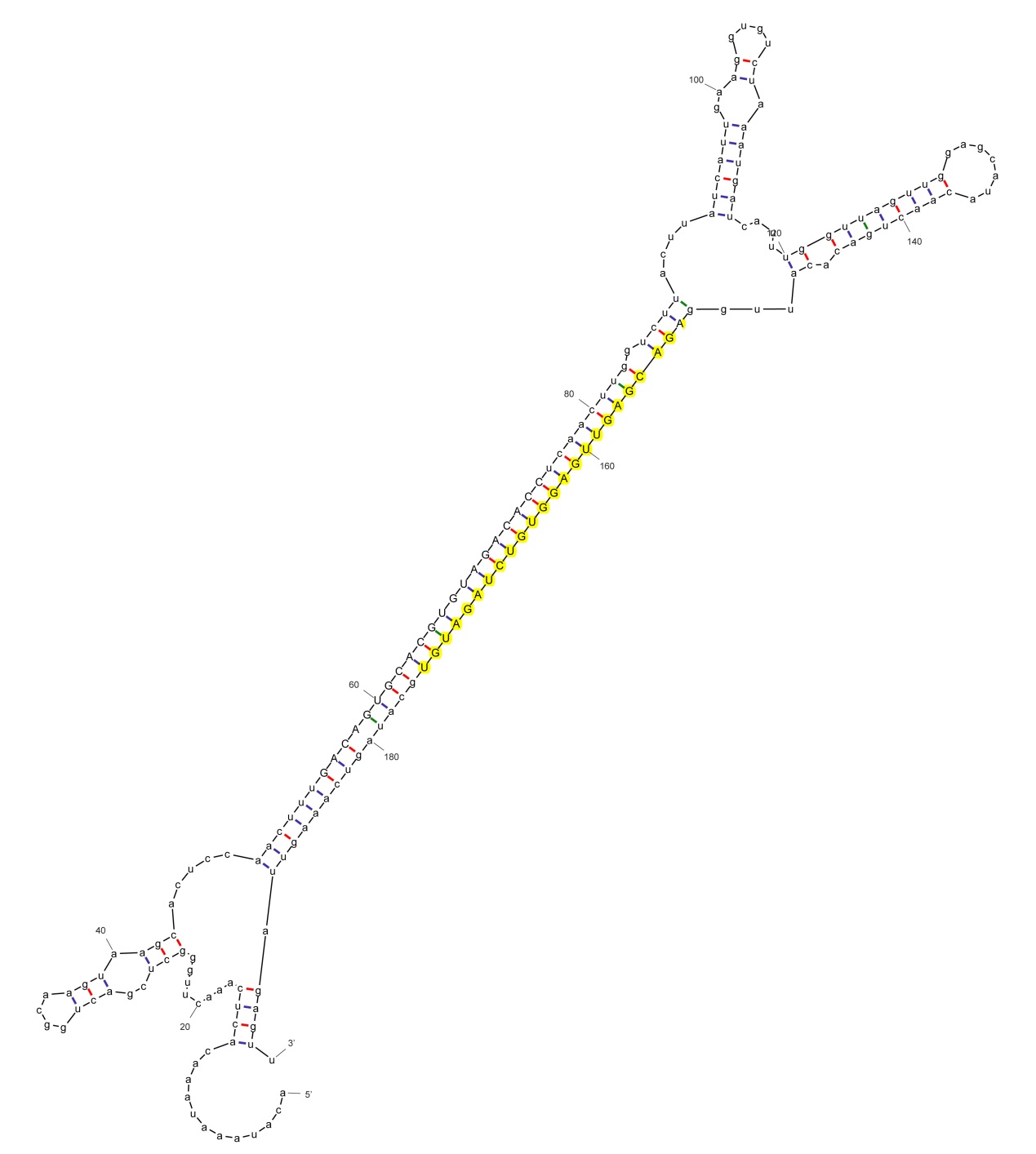


PC-51-3p


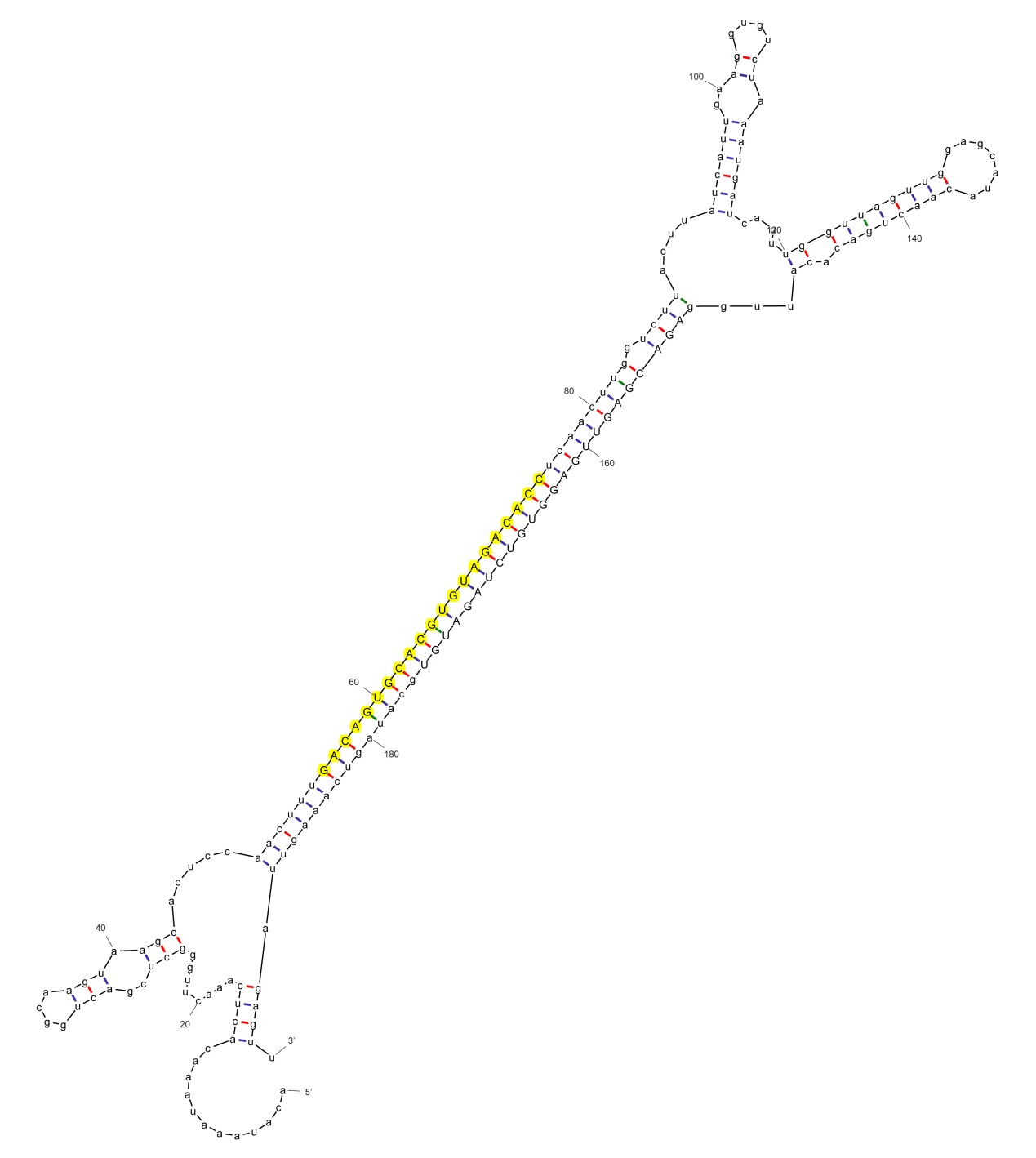


PC-51-5p


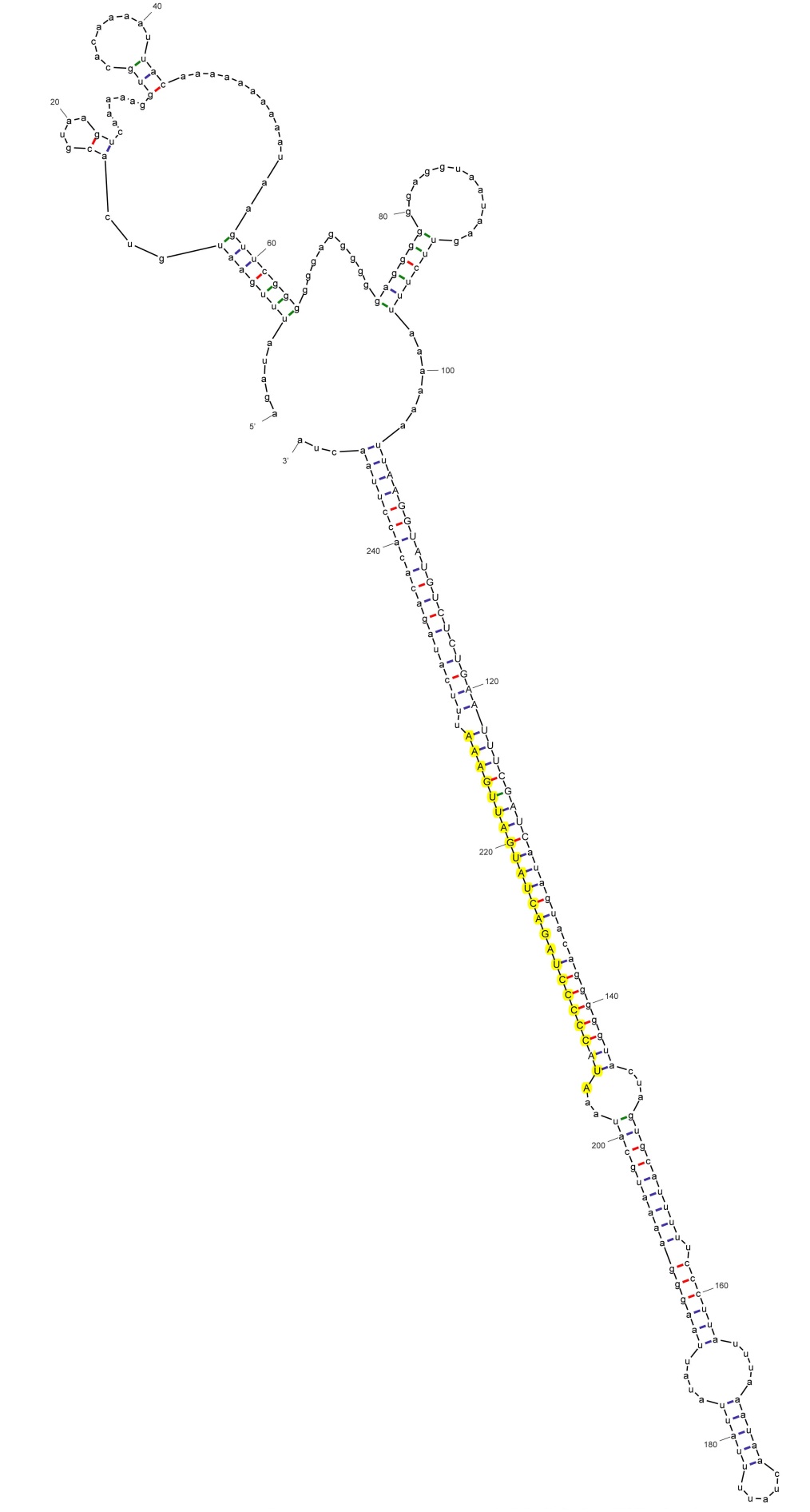


PC-52-3p


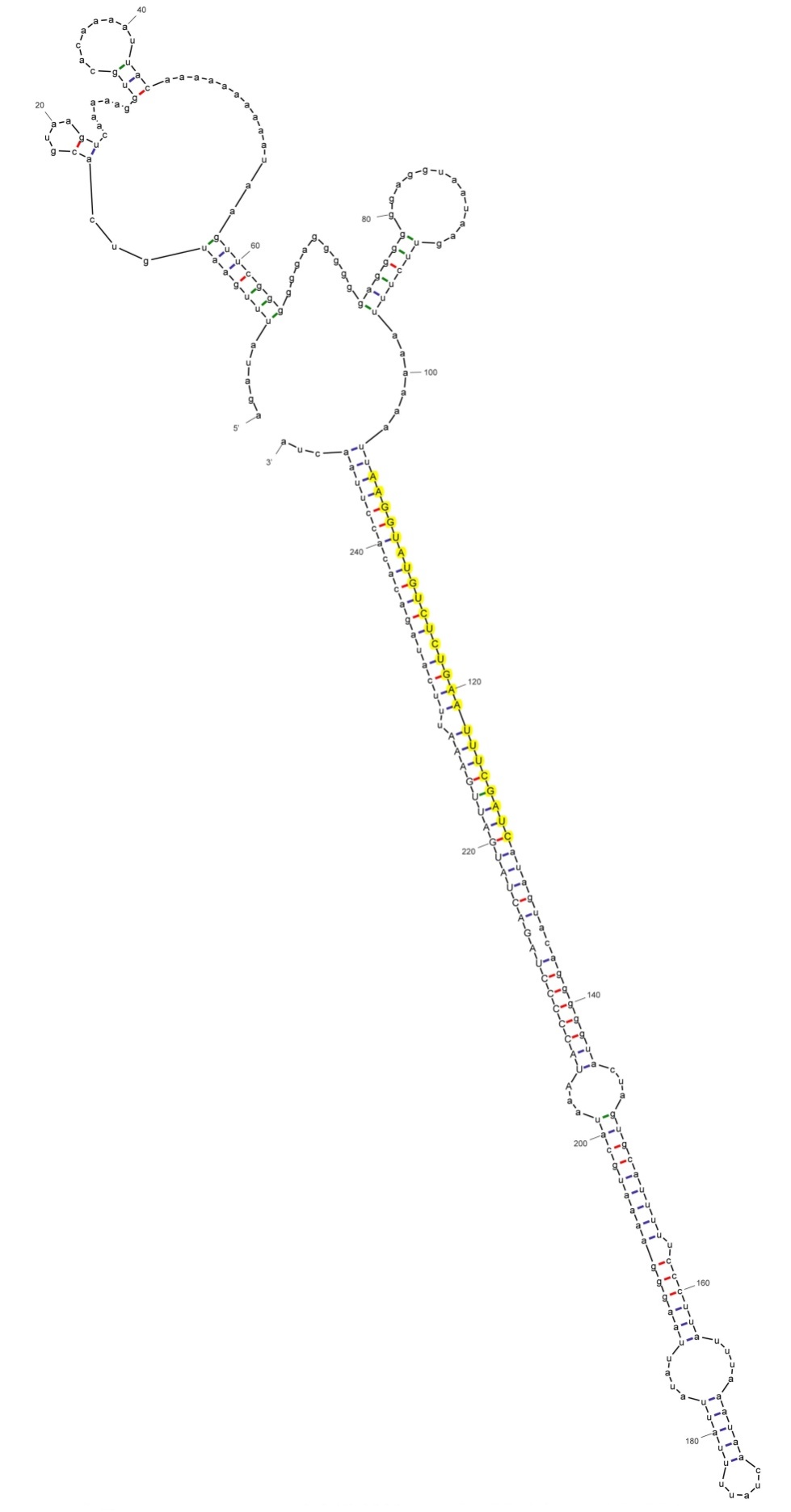


PC-52-5p


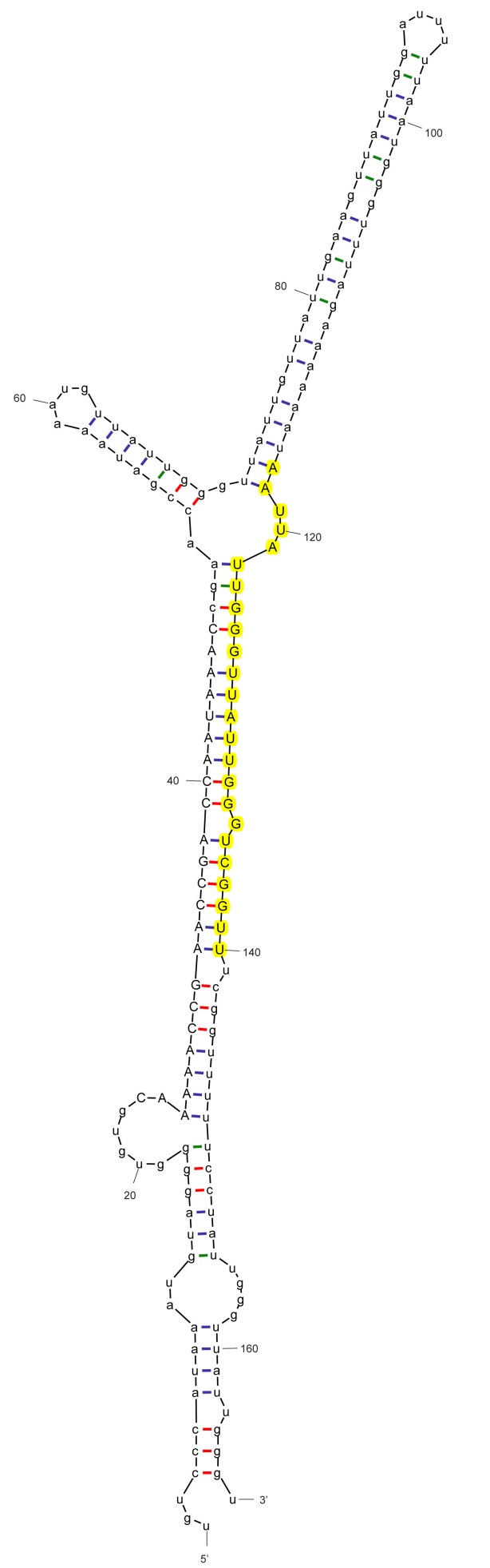


PC-53-3p


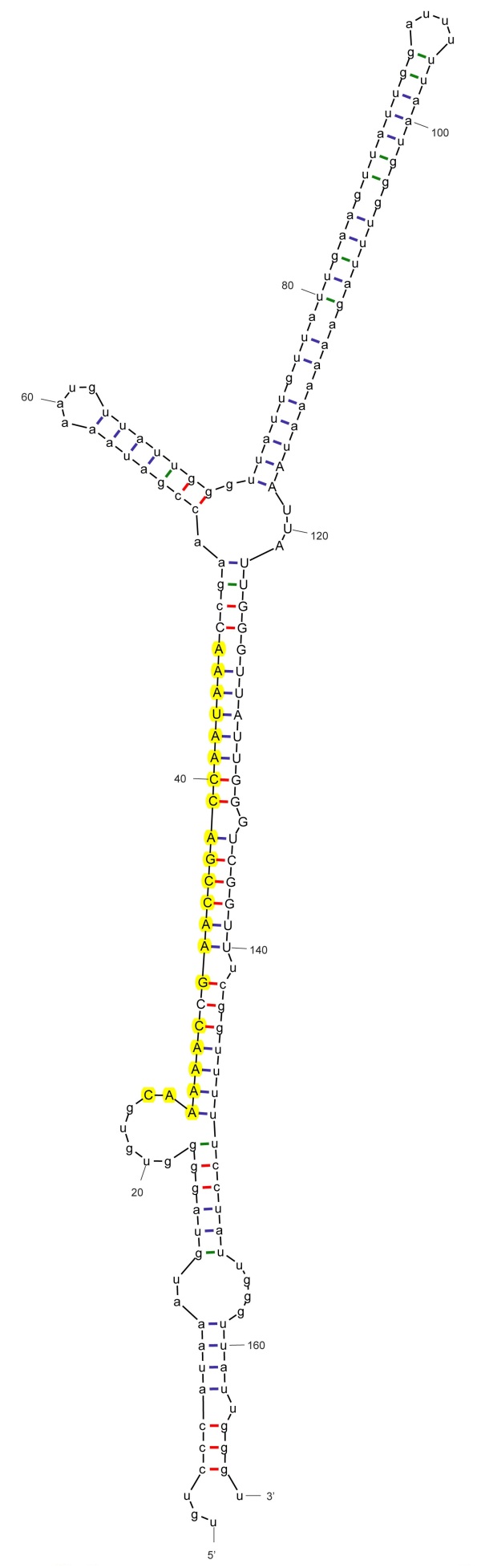


PC-53-5p


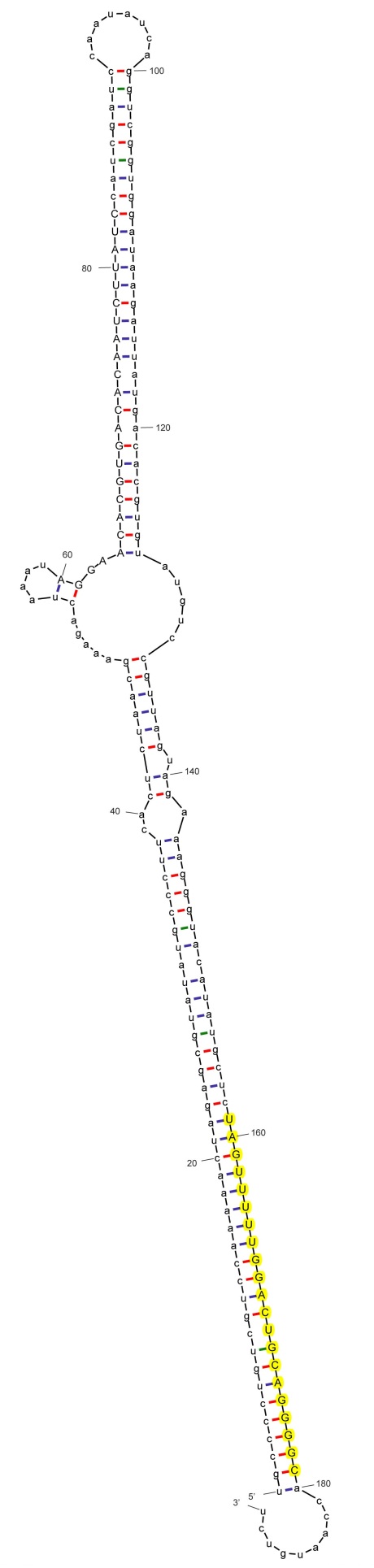


PC-54-3p


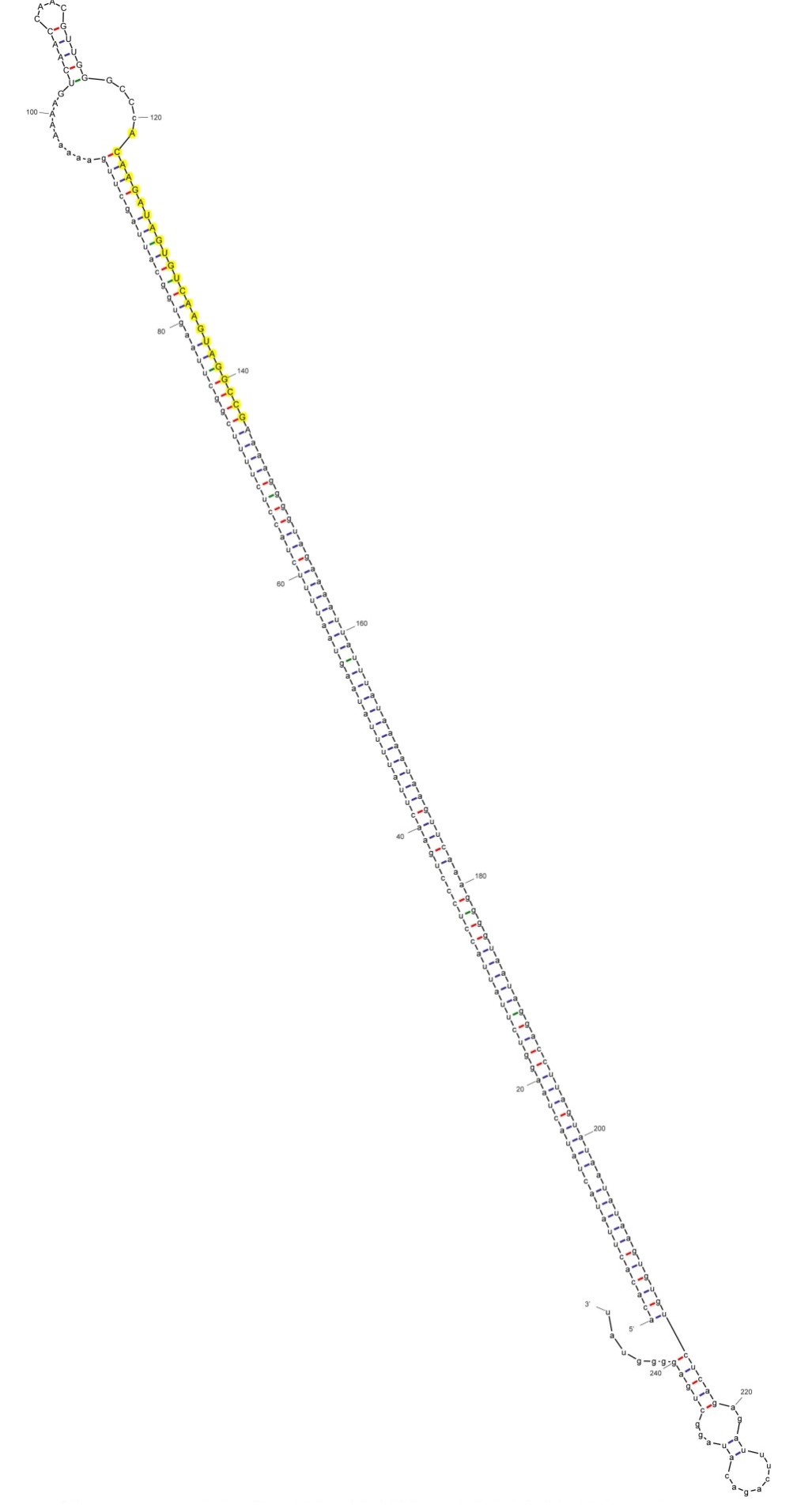


PC-55-3p


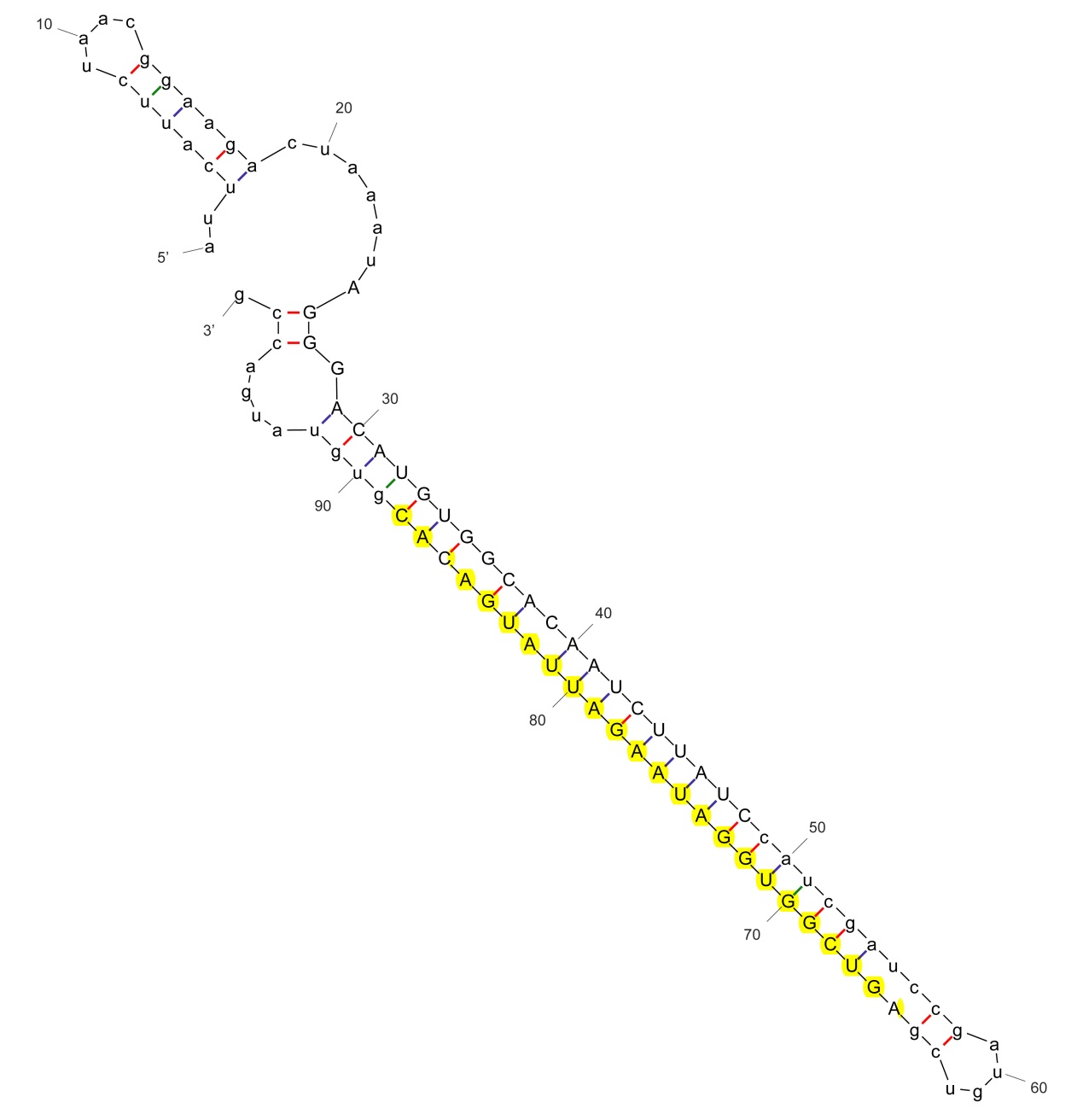


PC-56-3p


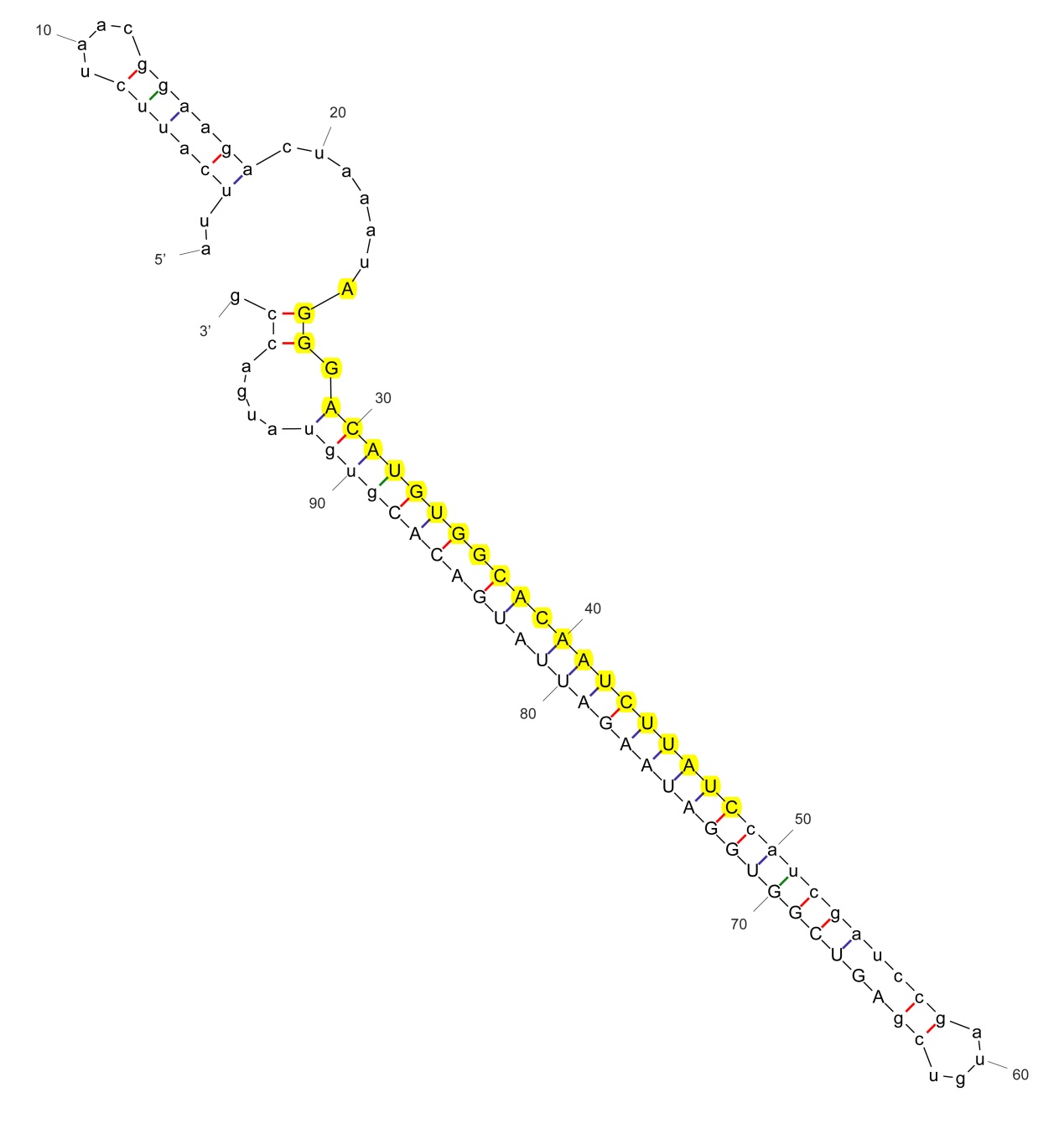


PC-56-5p


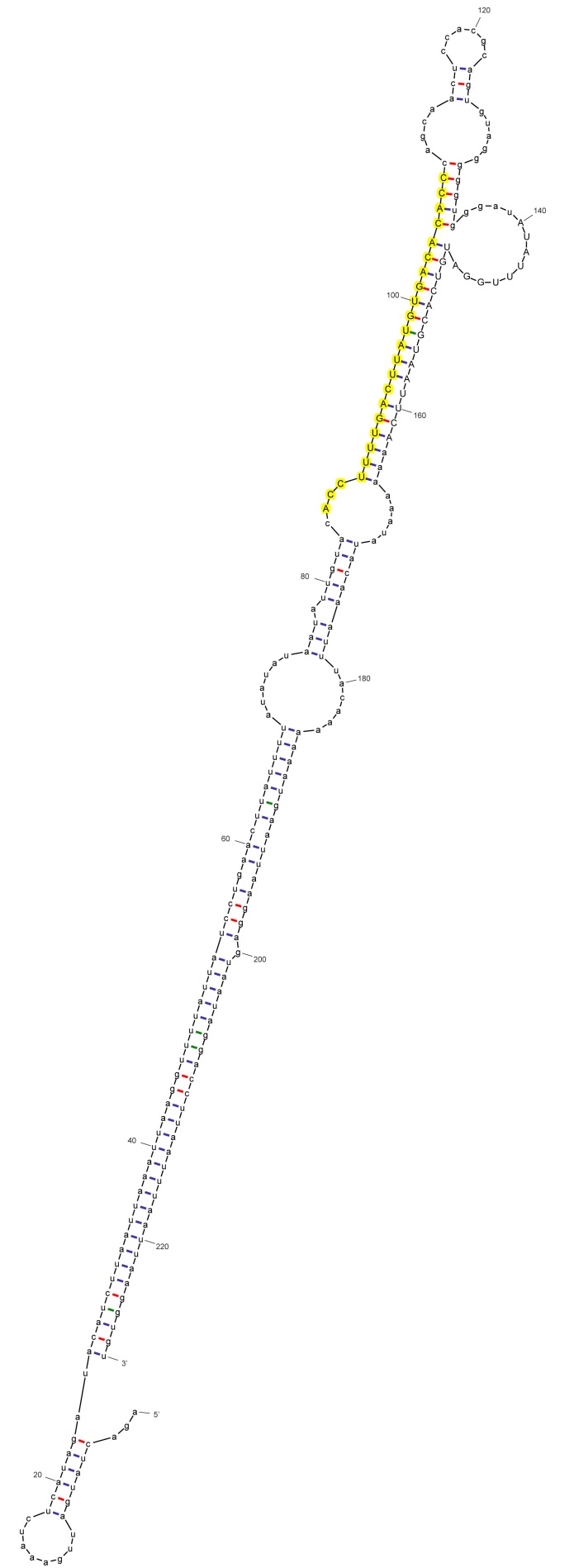


PC-57-5p


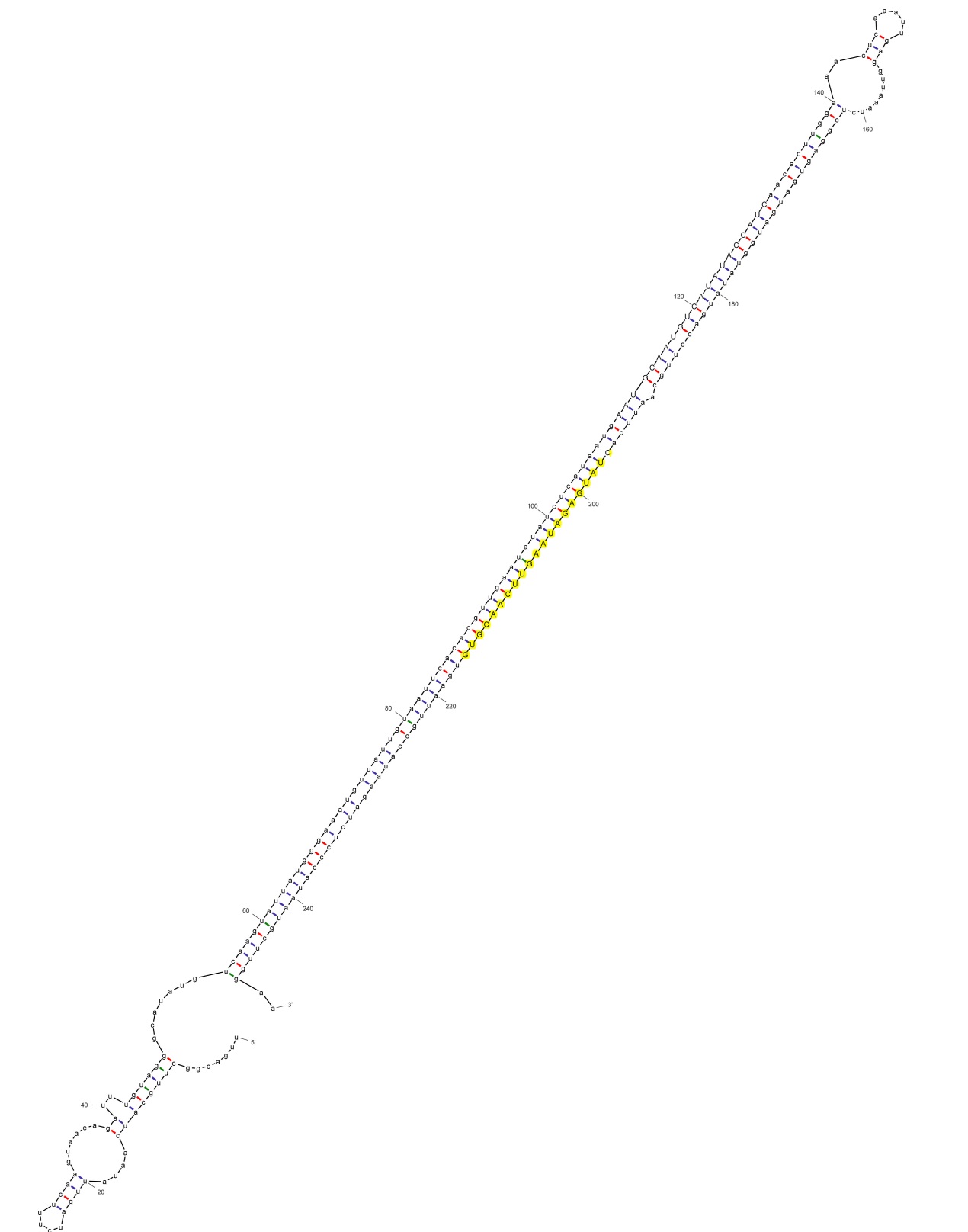


PC-58-3p


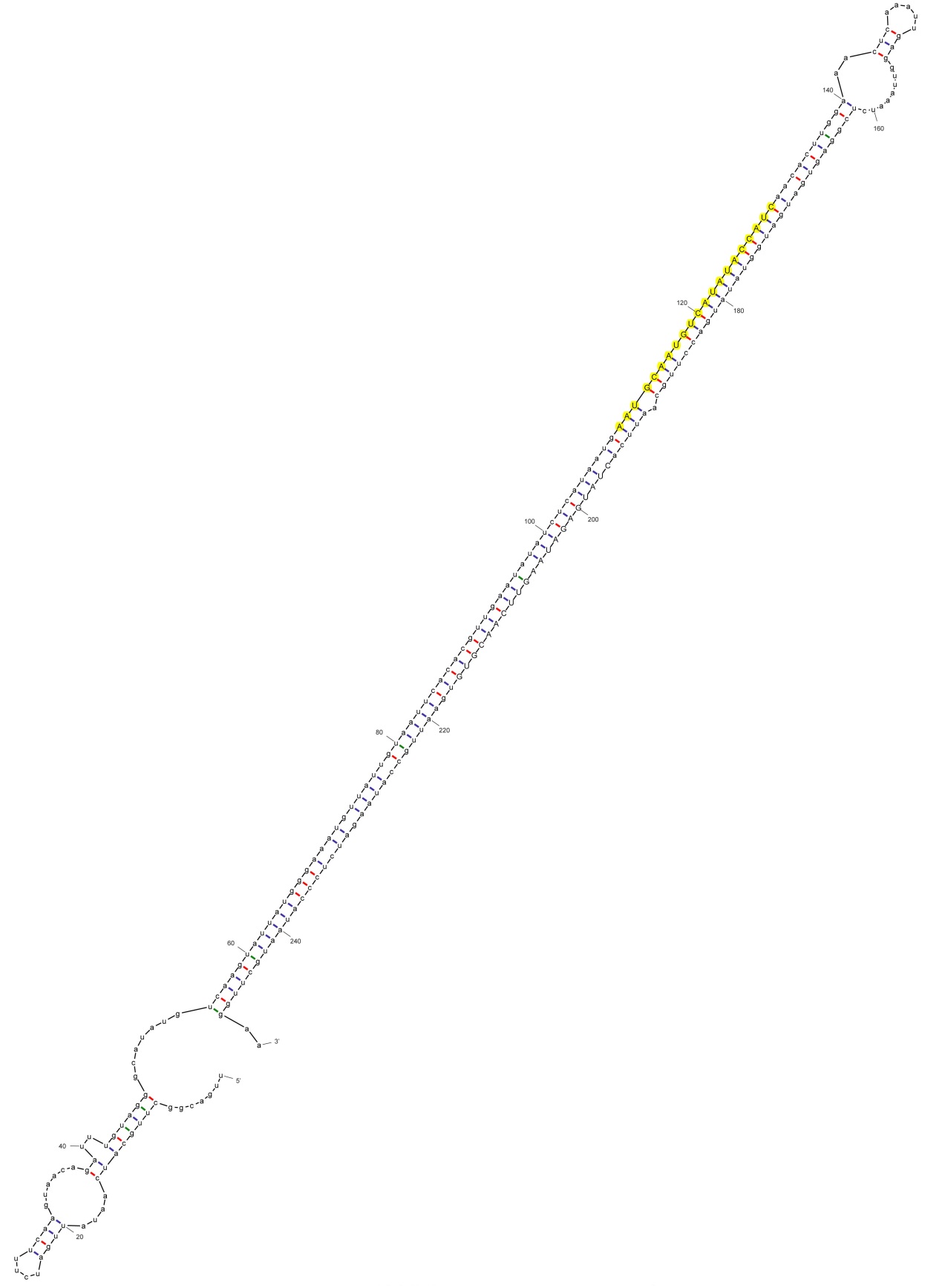


PC-58-5p


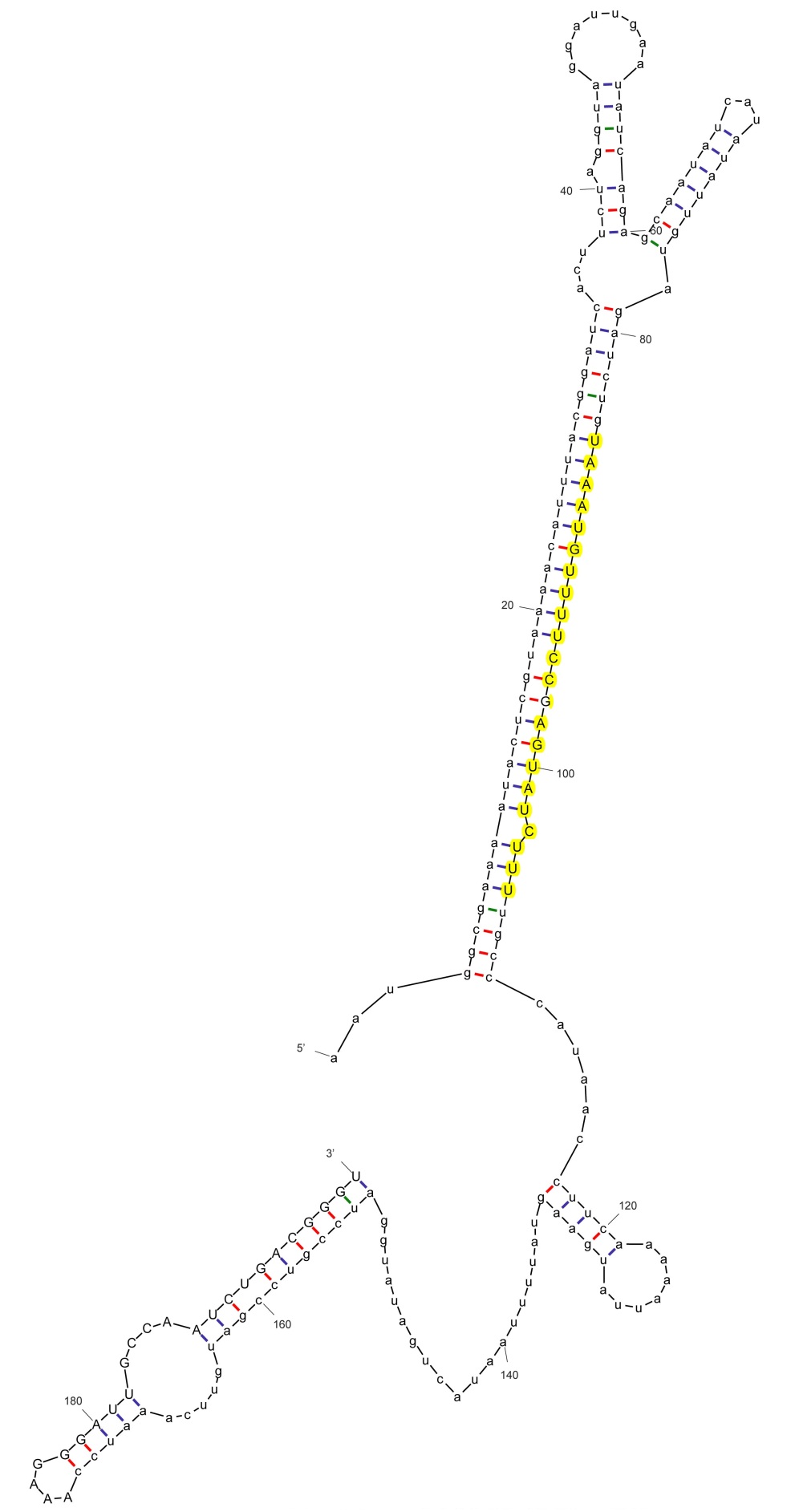


PC-59-5p


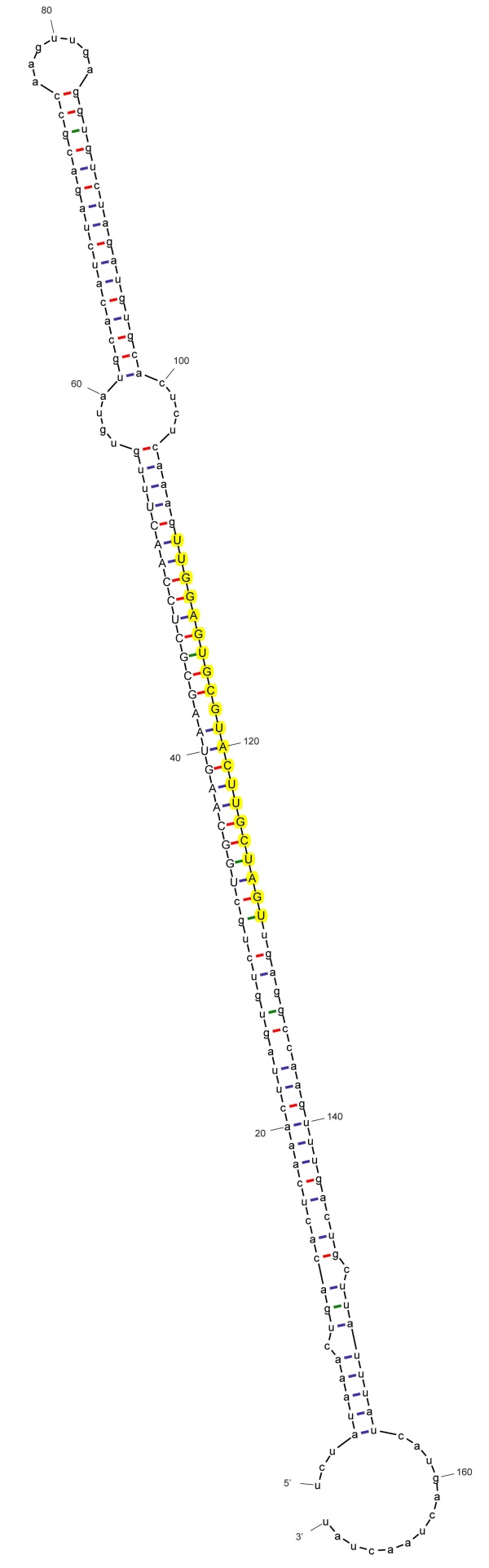


PC-60-3p


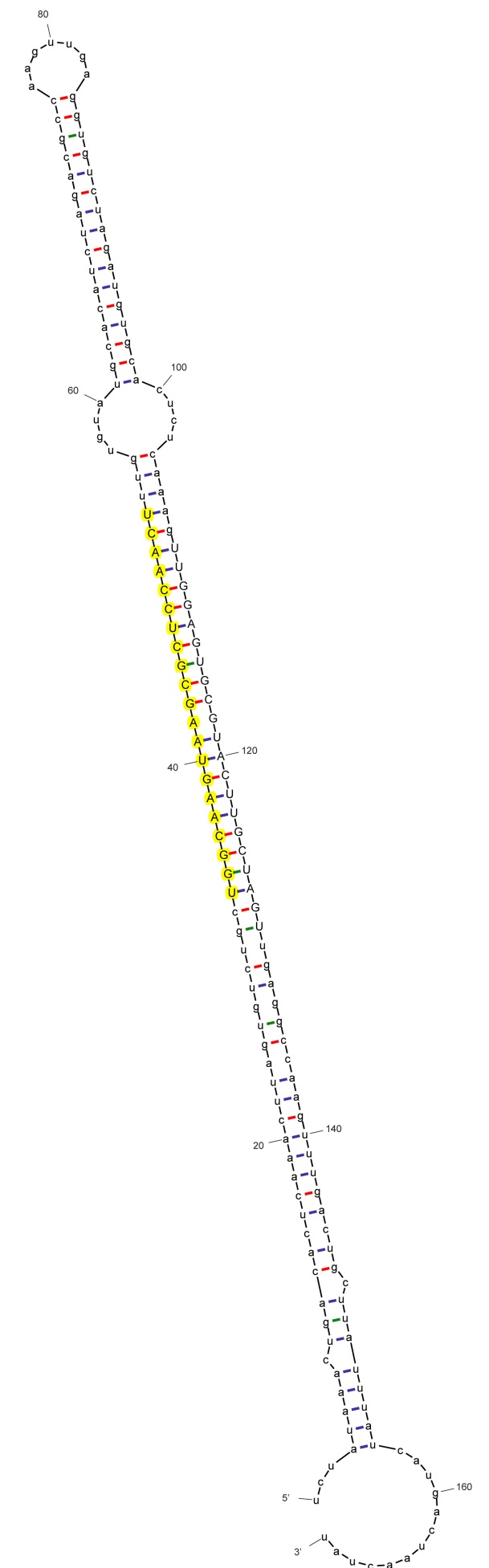


PC-60-5p


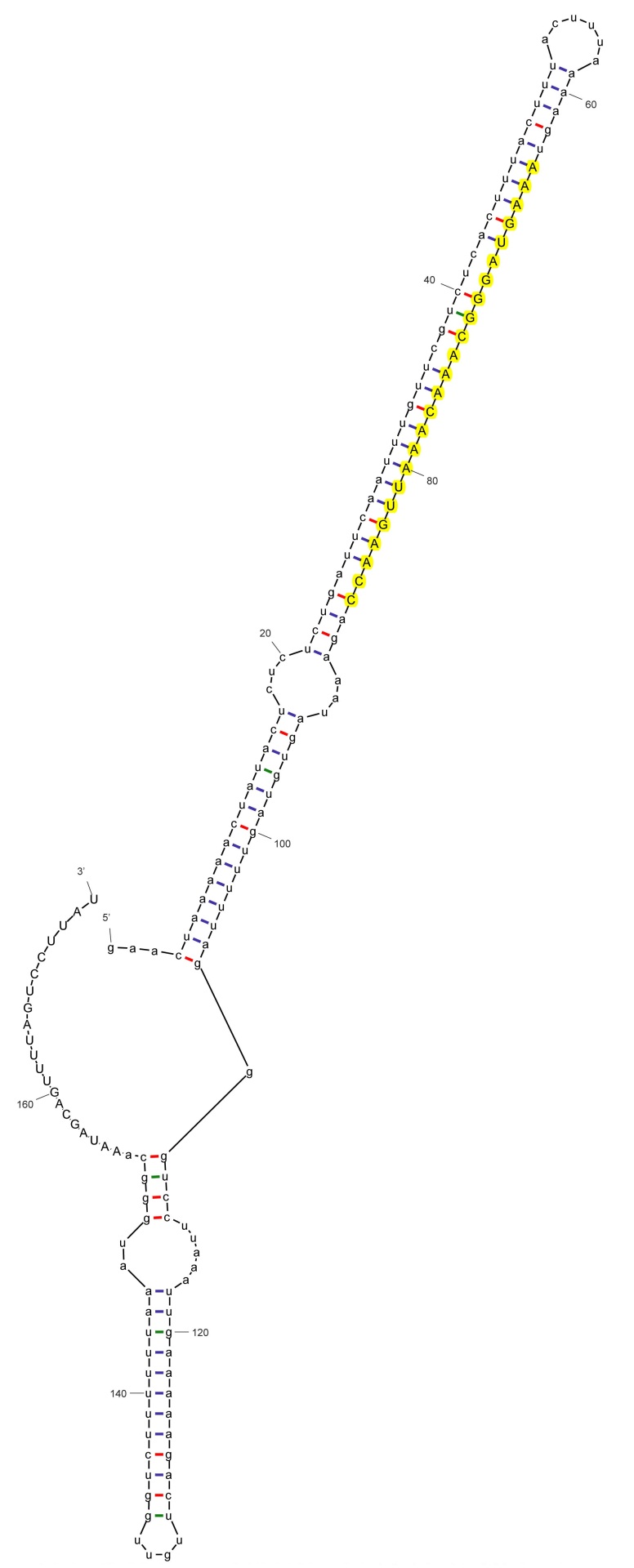


PC-61-5p


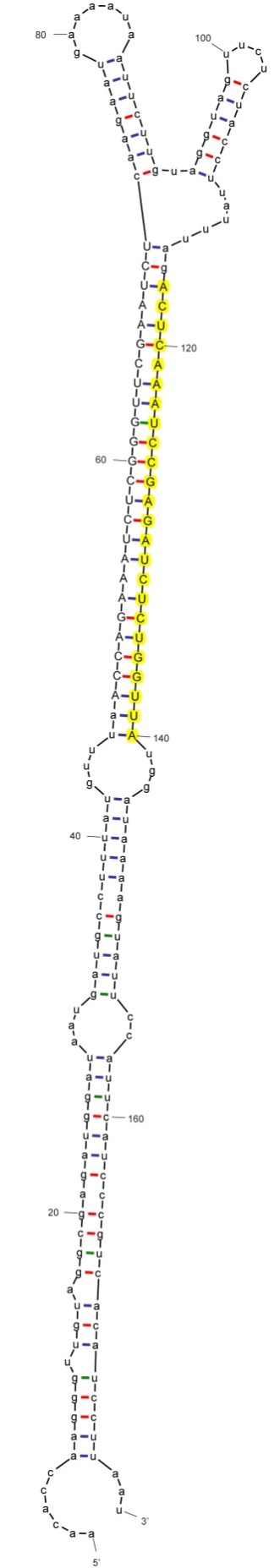


PC-62-3p


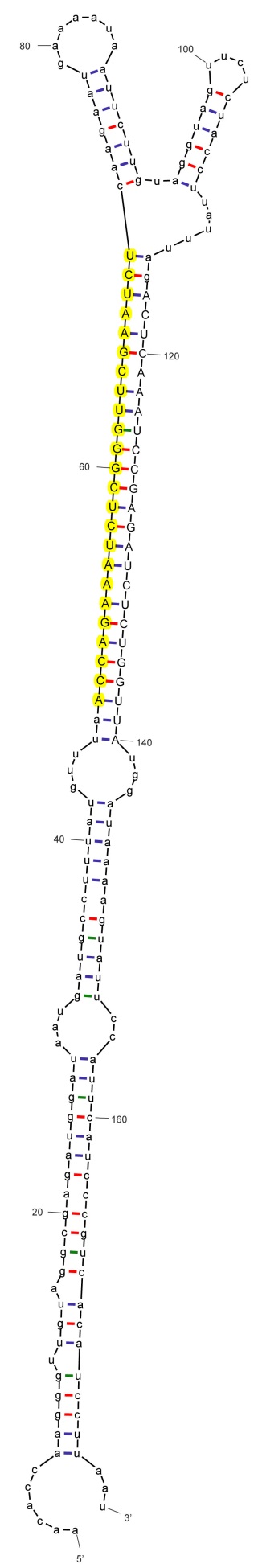


PC-62-5p


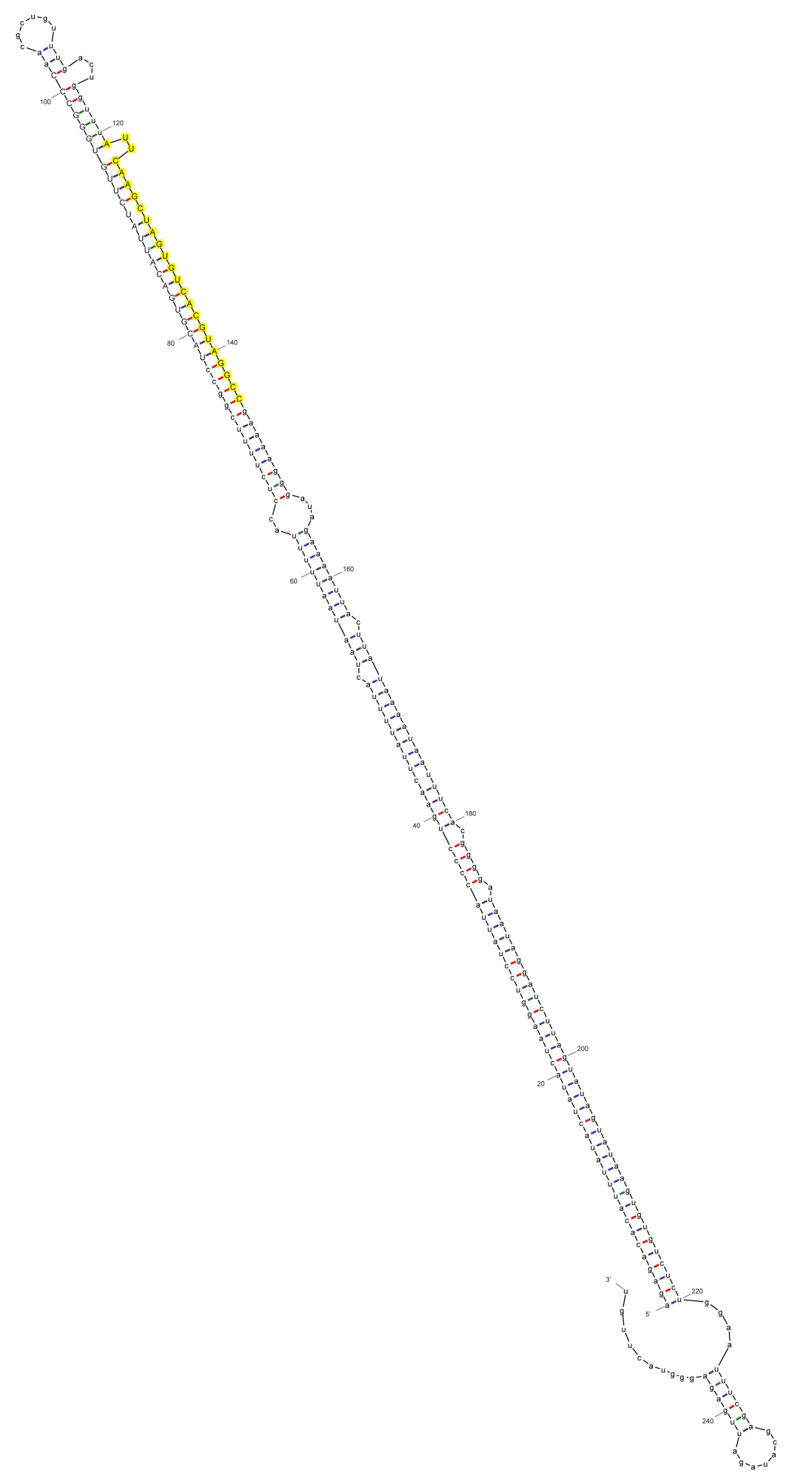


PC-63-3p


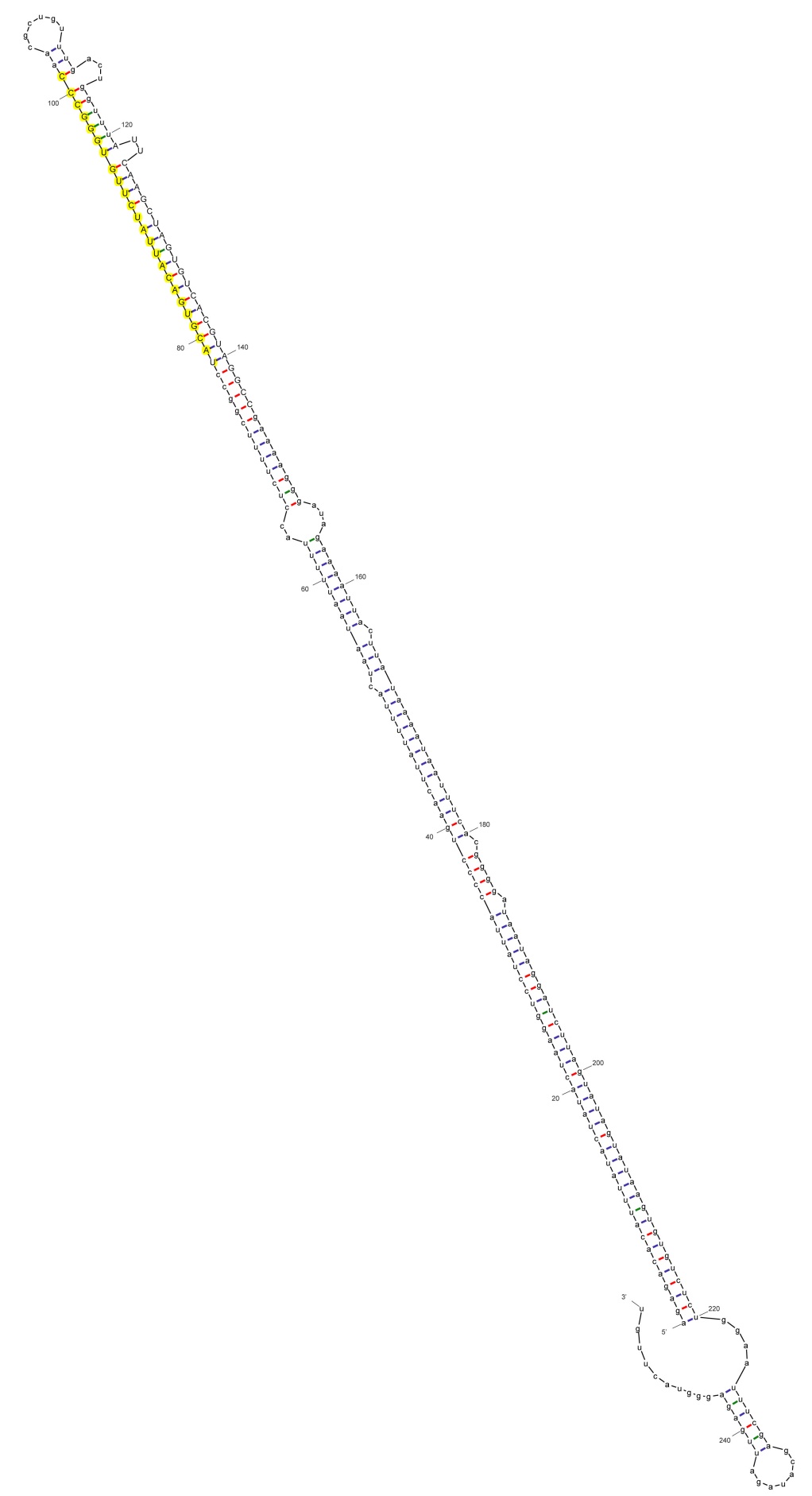


PC-63-5p


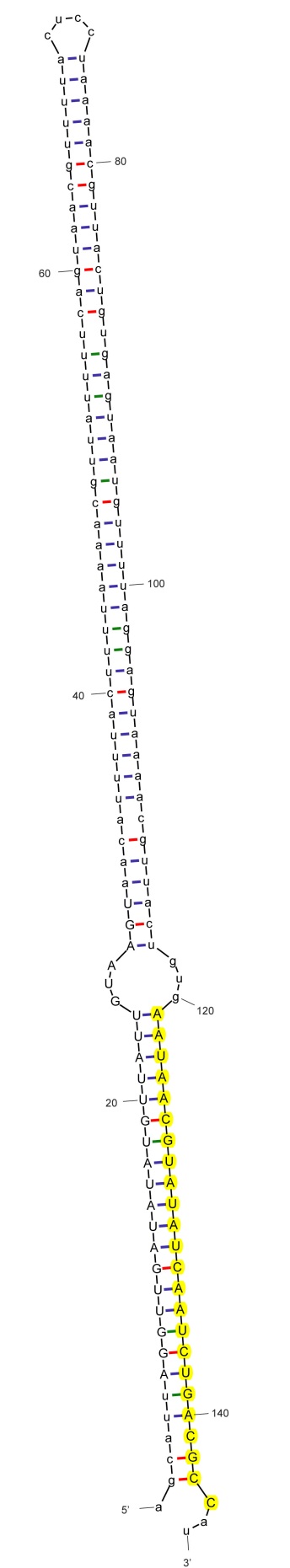


PC-64-3p


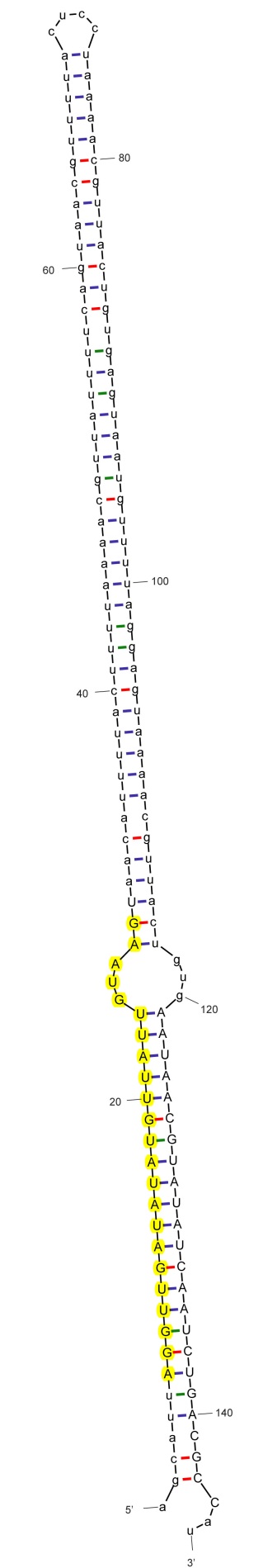


PC-64-5p


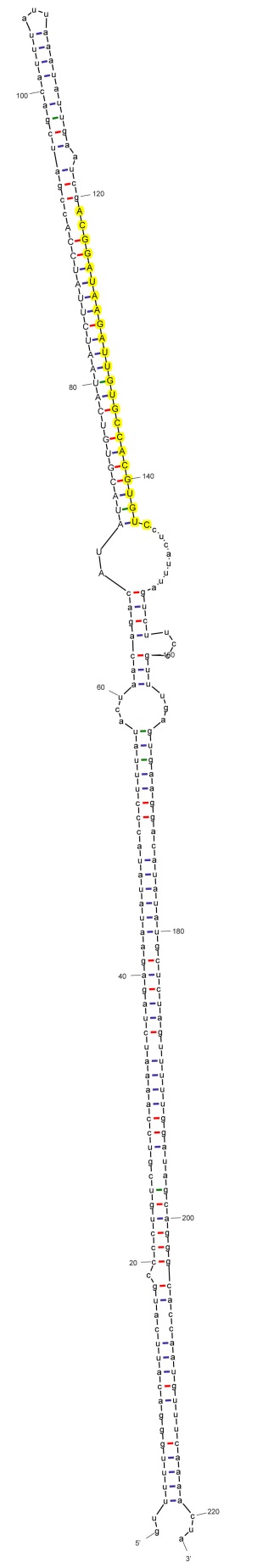


PC-65-3p


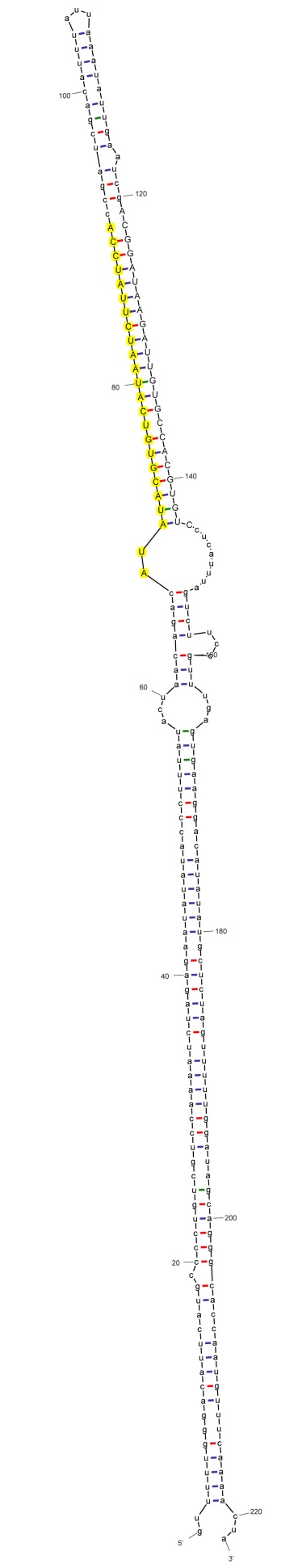


PC-65-5p


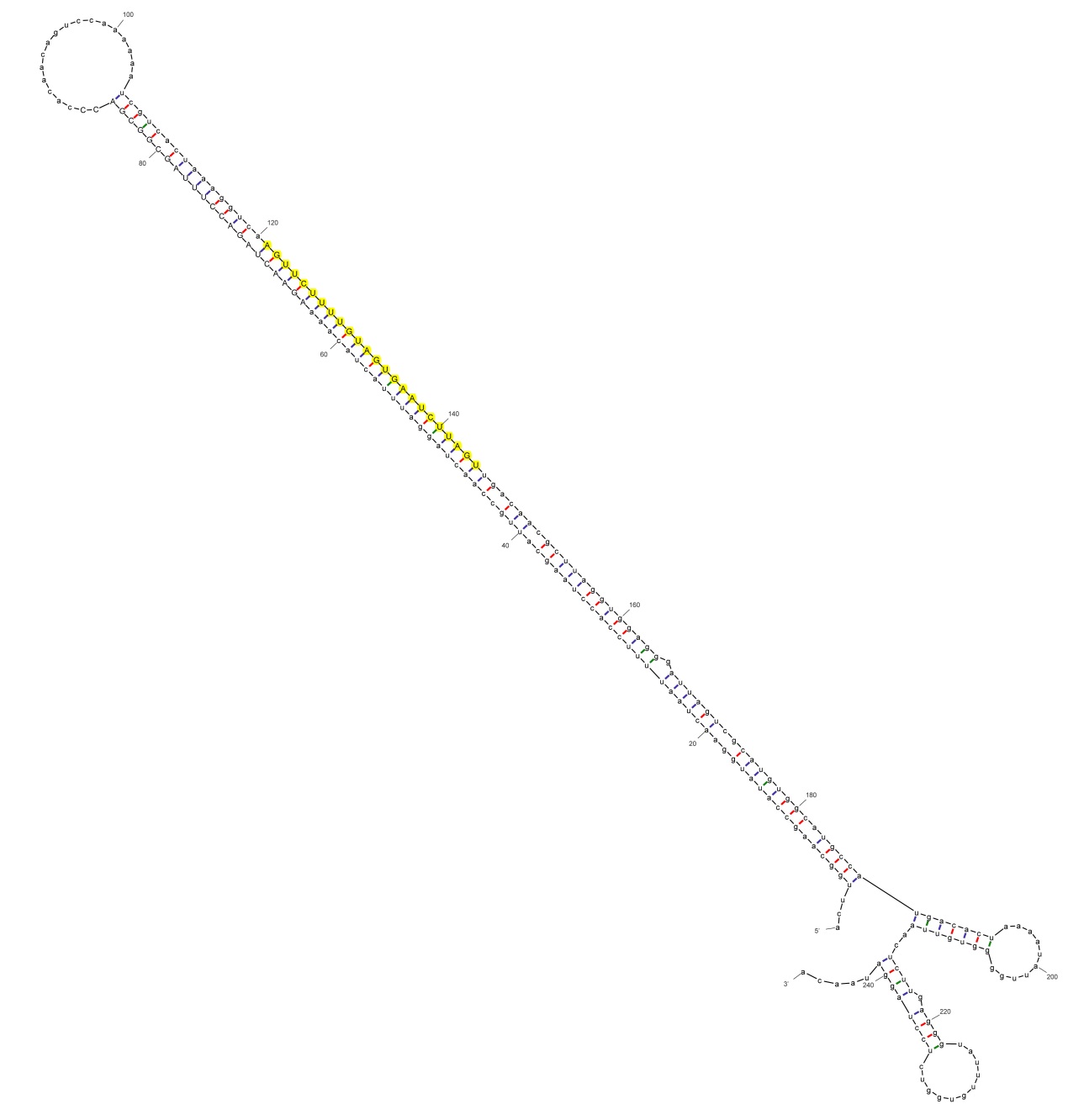


PC-66-3p


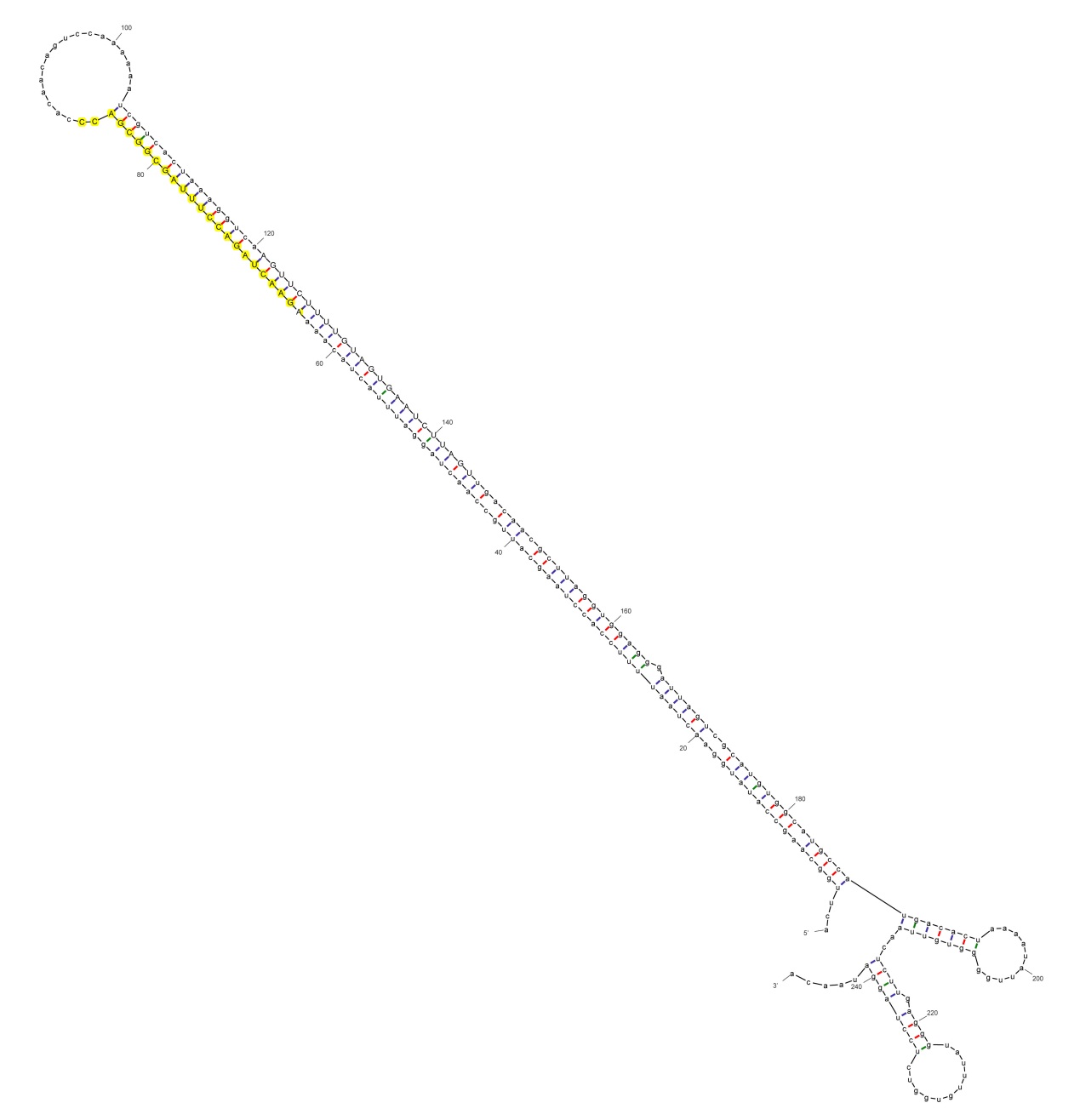


PC-66-5p


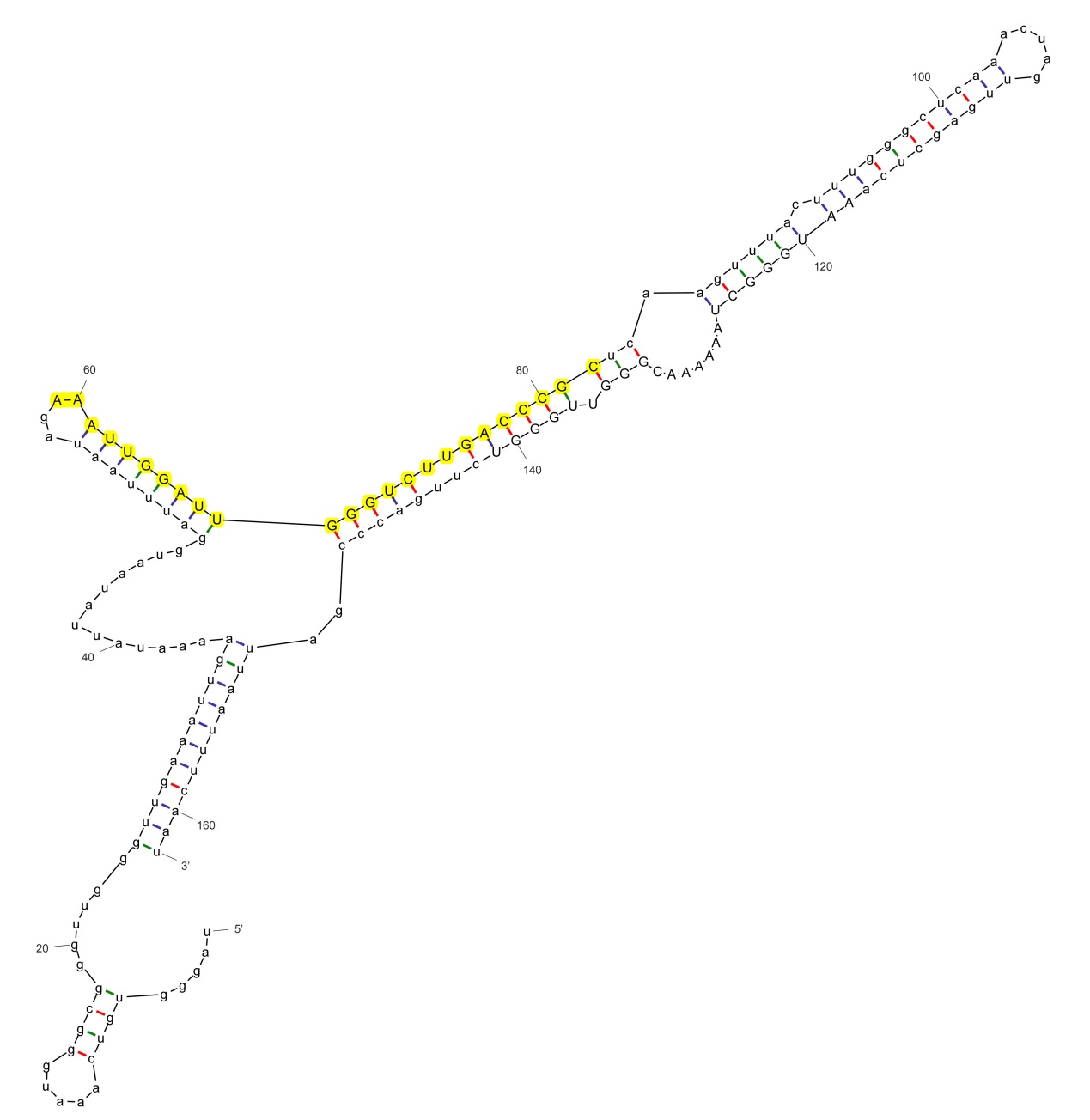


PC-67-5p


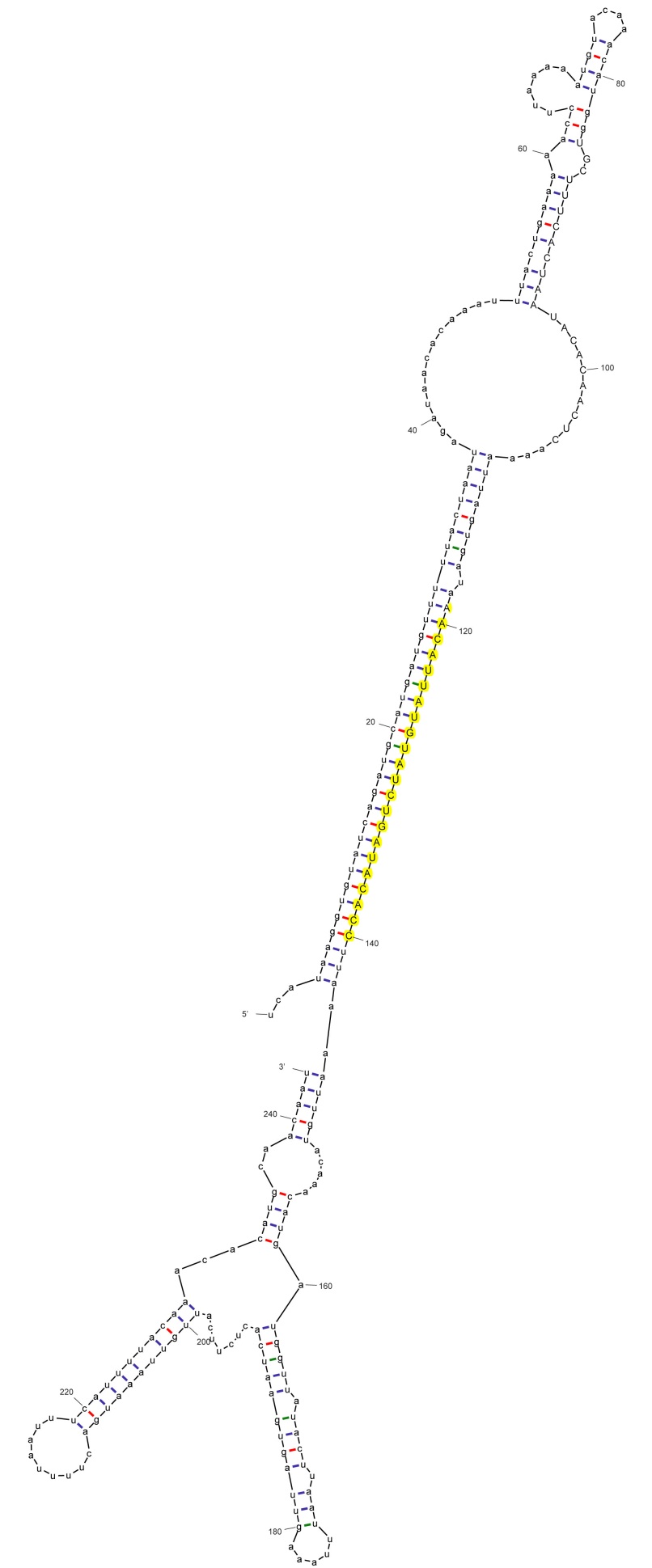


PC-68-3p


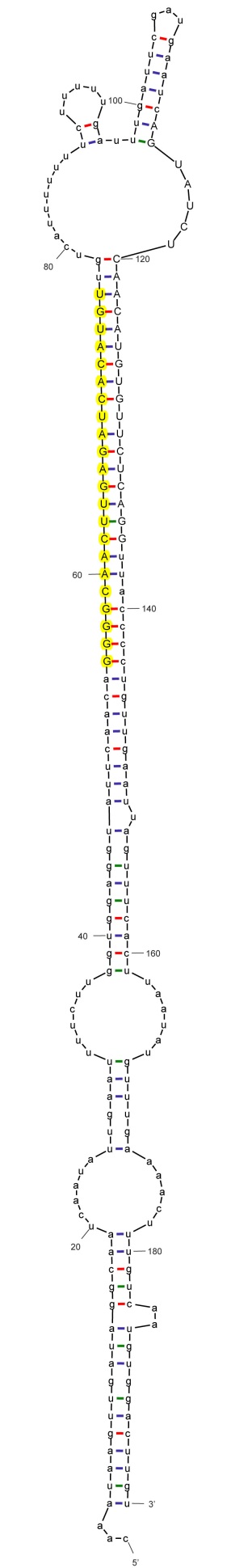


PC-69-5p


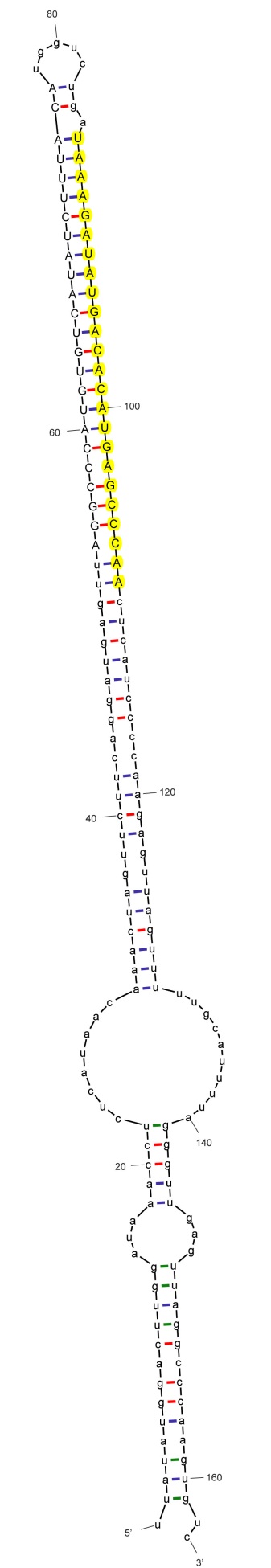


PC-70-3p


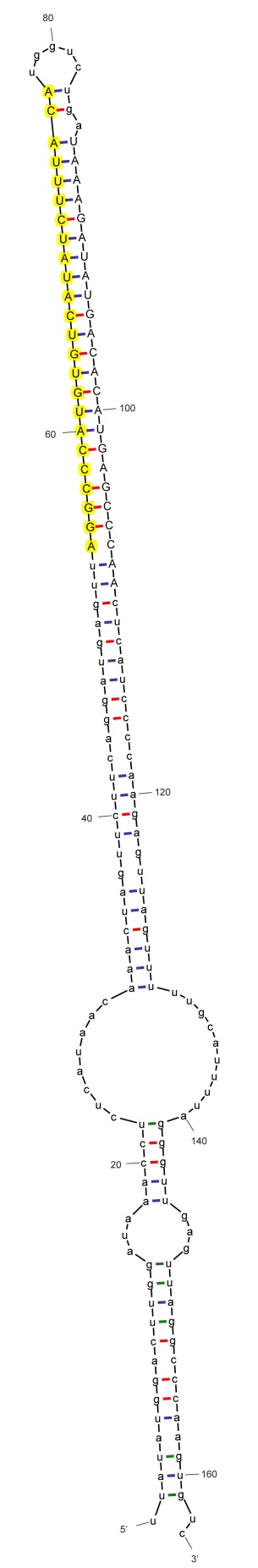


PC-70-5p


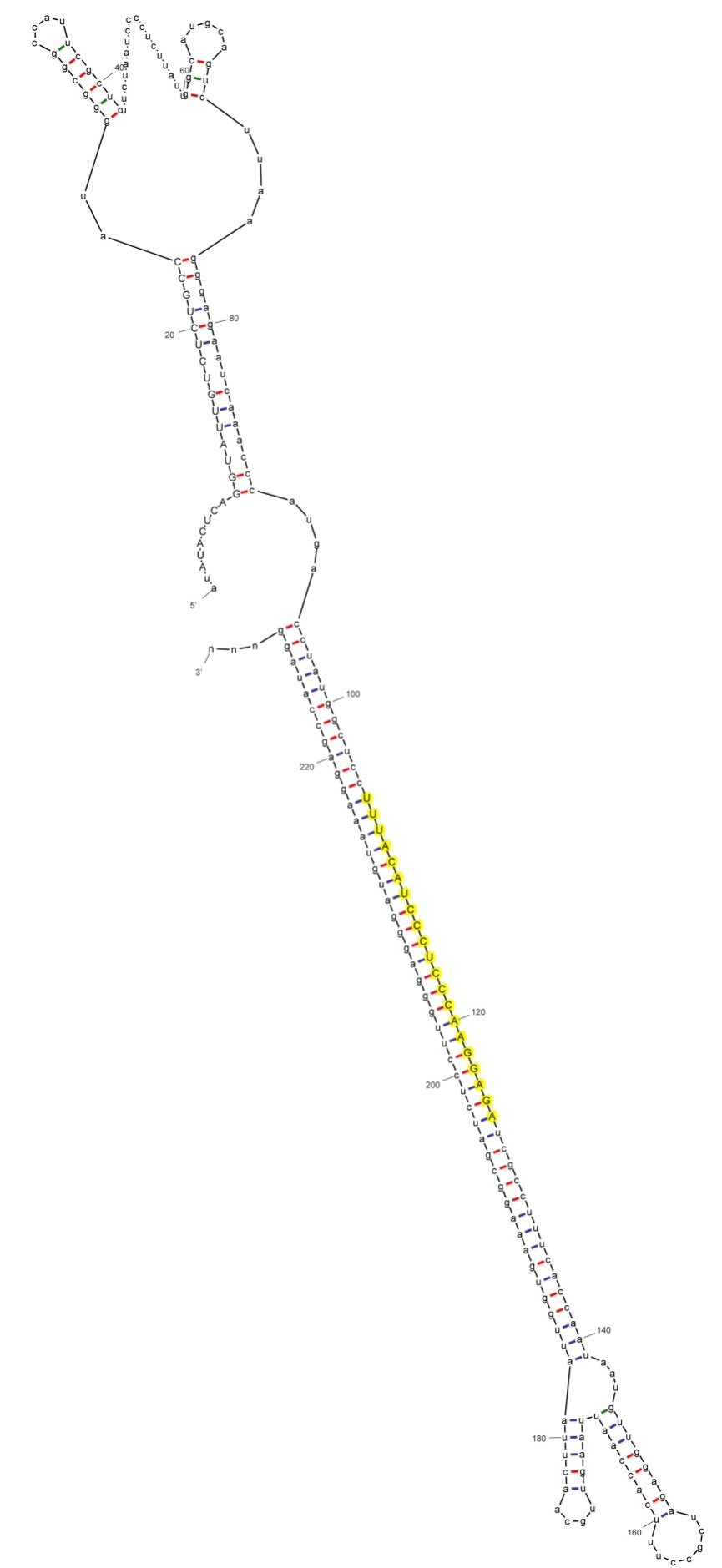


PC-71-3p


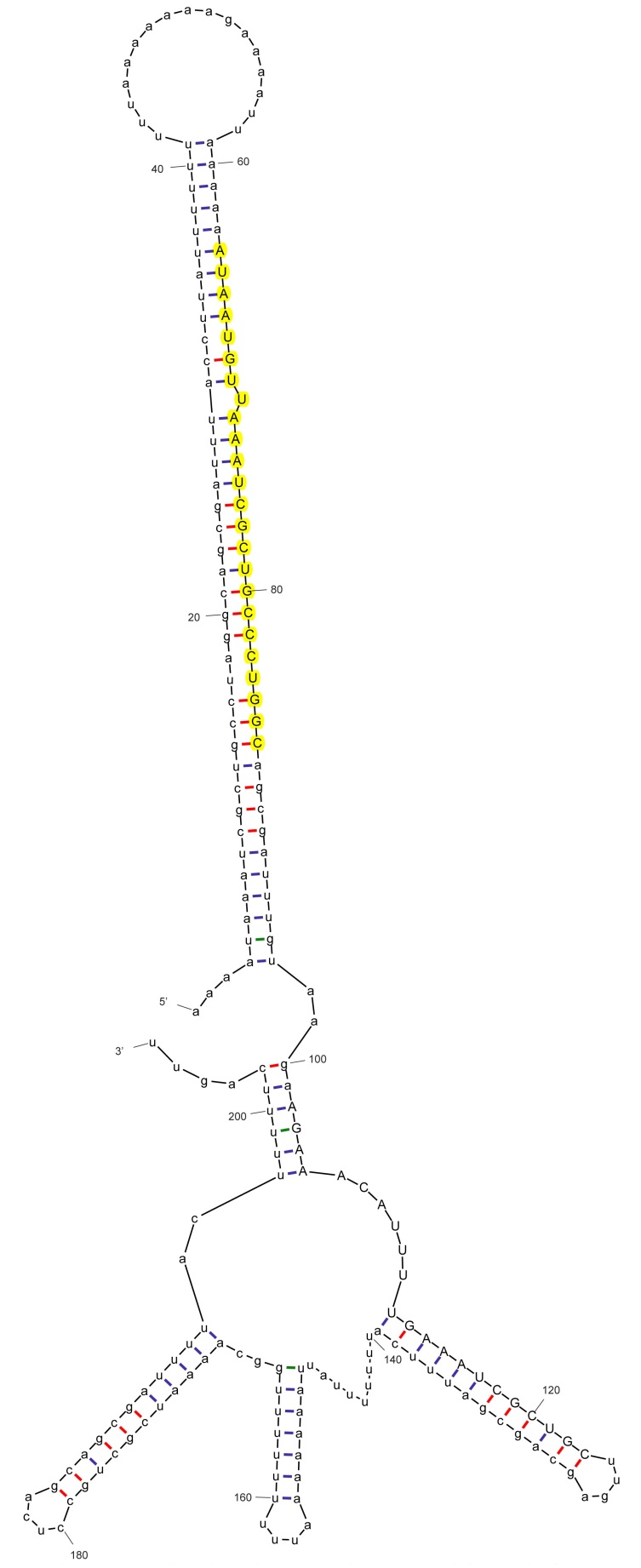


PC-72-5p


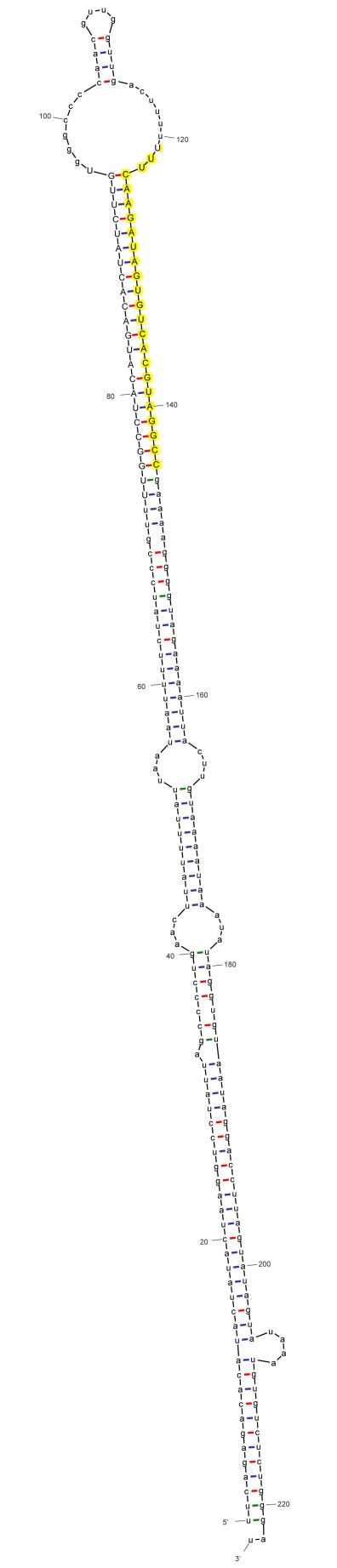


PC-73-3p

PC-73-5p

PC-74-3p

PC-75-3p

PC-75-5p

PC-76-3p

PC-76-5p

PC-77-3p

PC-77-5p

PC-78-3p

PC-78-5p

PC-79-5p

PC-80-5p

PC-81-3p

PC-81-5p

PC-82-3p

PC-82-5p

PC-83-5p

PC-84-3p

PC-84-5p

PC-85-5p

PC-86-3p

PC-87-3p

PC-87-5p

PC-88-3p

PC-88-5p

PC-89-3p

PC-89-5p

PC-90-3p

PC-90-5p

PC-91-3p

PC-92-3p

PC-93-3p

PC-93-5p

PC-94-3p

PC-94-5p

PC-95-5p

PC-96-5p

PC-97-3p

PC-97-5p

PC-98-5p

PC-99-3p

PC-99-5p

PC-100-5p

PC-101-3p

PC-102-3p

PC-102-5p

PC-103-3p

PC-103-5p

PC-104-3p

PC-104-5p

PC-105-3p

PC-105-5p

PC-106-5p

PC-107-5p

PC-108-3p

PC-109-3p

PC-110-5p

PC-111-3p

PC-112-3p

PC-112-5p

PC-113-3p

PC-113-5p

PC-114-5p

PC-115-3p

PC-115-5p

PC-116-3p

PC-116-5p

PC-117-3p

PC-117-5p

PC-118-3p

PC-118-5p

PC-119-3p

PC-120-5p

PC-121-3p

PC-121-5p

PC-122-3p

PC-122-5p

PC-123-3p

PC-123-5p

PC-124-3p

PC-124-5p

PC-125-3p

PC-125-5p

PC-126-3p

PC-126-5p

PC-127-3p

PC-128-3p

PC-128-5p

PC-129-5p

PC-130-3p

PC-131-3p

PC-131-5p

PC-132-3p

PC-132-5p

PC-133-3p

PC-133-5p

PC-134-3p

PC-134-5p

PC-135-3p

PC-136-3p

PC-136-5p

PC-137-5p

PC-138-3p

PC-139-5p

PC-140-5p

PC-141-5p

PC-142-5p

PC-143-5p

PC-144-5p

PC-145-5p

PC-146-5p

PC-147-3p

PC-148-3p

PC-149-5p

PC-150-3p

PC-151-3p

PC-152-5p

PC-153-5p

PC-154-3p

PC-155-5p

PC-156-3p

PC-157-3p

PC-158-5p

PC-159-3p

PC-160-3p

PC-161-3p

PC-162-3p

PC-163-5p

PC-164-3p

PC-165-5p

PC-166-3p

PC-167-5p

PC-168-3p

PC-169-5p

PC-170-5p

PC-171-5p
