# Supplementary material for: Identification of chilling stress-responsive tomato microRNAs and their target genes by high-throughput sequencing and degradome analysis
Source: BMC Genomics. 2014 Dec 17;15(1):1130. doi: 10.1186/1471-2164-15-1130 (PMC4377850; doi:10.1186/1471-2164-15-1130)
Supplement: Supplementary file 6 — Additional file 6: Figure S3: - T-plots for targets of tomato miRNAs identified in the NT library. According to German et al, t-plots are referred to as “target plots” and the normalized numbers are used to plot the cleavages on target mRNAs. The frequency of degradome tags with 5’ends at the indicated positions is marked in black, and the frequency cleaved at position 10 of the inset miRNA target alignment is highlighted in red. The abscissa number t indicates the cleavage site detected in the target mRNA. (DOCX 14 MB) [file 12864_2014_6877_MOESM6_ESM.docx]

sha-miR156a and sha-miR156c slicing SGN-E731350 at nt510


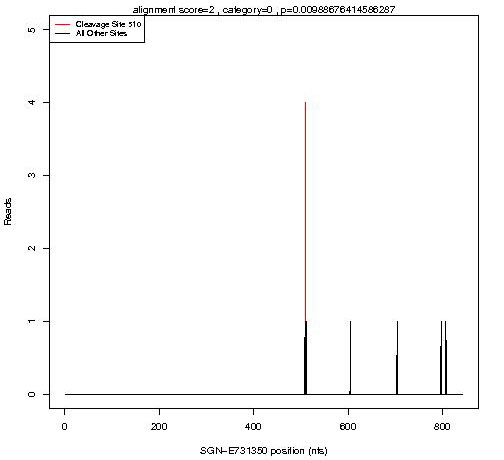

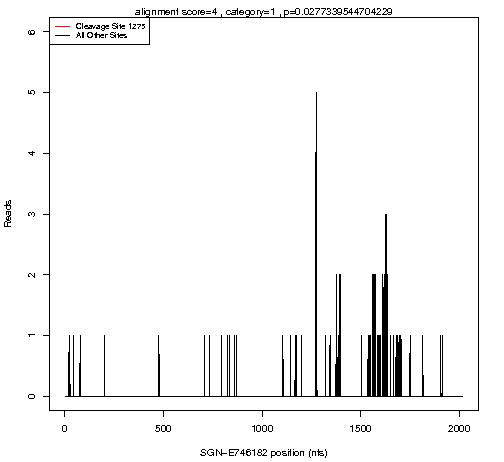


sha-miR156a and sha-miR156c slicing SGN-E746182 at nt1275


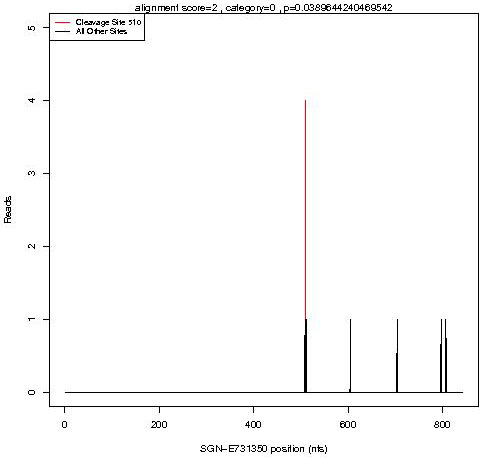


sha-miR156c_nta slicing SGN-E731350 at nt510

sha-miR156c_nta slicing SGN-E746182 at nt 1275


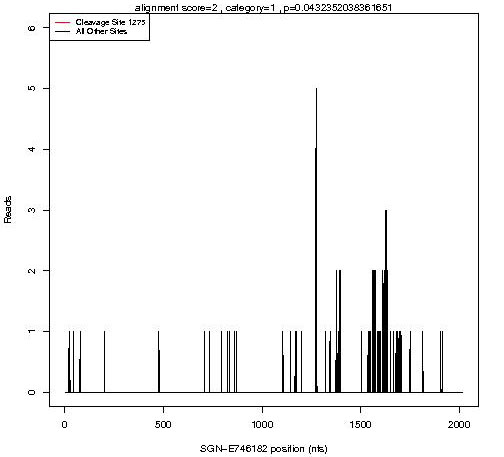

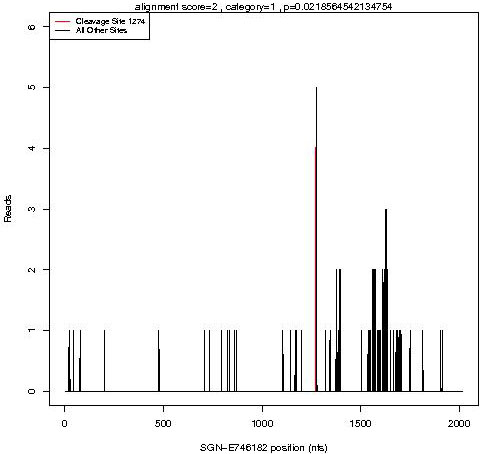


sha-miR156d_nta slicing SGN-E746182 at nt 1274


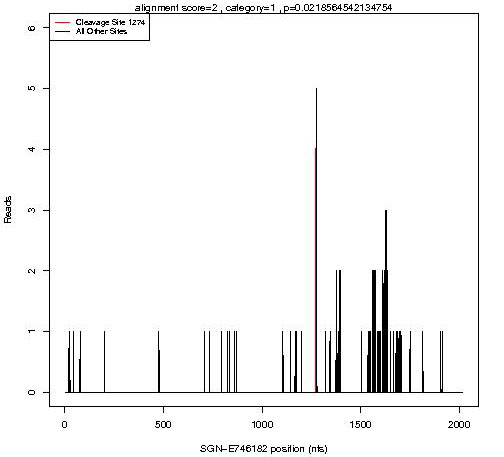


sha-miR156e_stu,sha-miR156g_stu,sha-miR156h_stu,sha-miR156i_stu and sha-miR156j_stu slicing SGN-E746182 at nt 1274


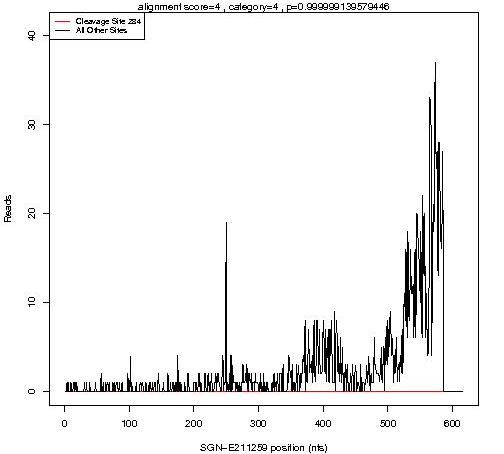


sha-miR156i-p3_nta slicing SGN-E211259 at nt 284

sha-miR156i-p3_nta slicing SGN-E236387 at nt 78


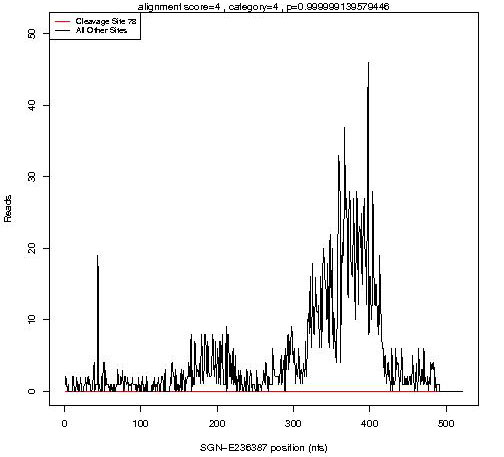


sha-miR156i-p3_nta slicing SGN-E211259 at nt 284

sha-miR156i-p3_nta slicing SGN-E211259 at nt 284

sha-miR156i-p3_nta slicing SGN-E241629 at nt 78


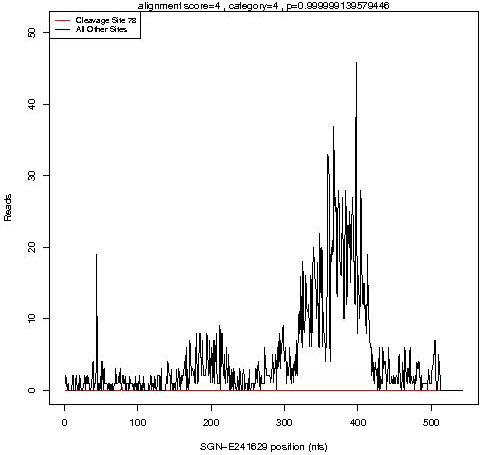

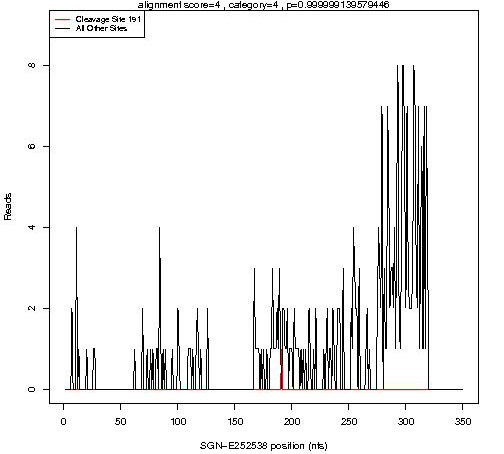


sha-miR156i-p3_nta slicing SGN-E252538 at nt 191

sha-miR156i-p3_nta slicing SGN-E258900 at nt 223


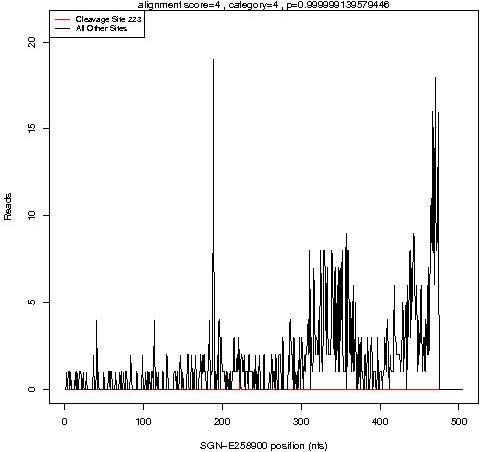


sha-miR156i-p3_nta slicing SGN-E297170 at nt 64


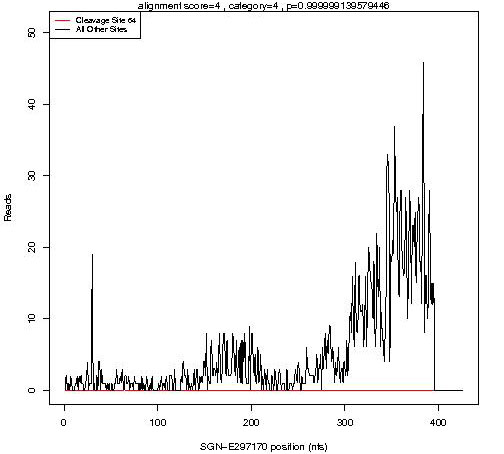

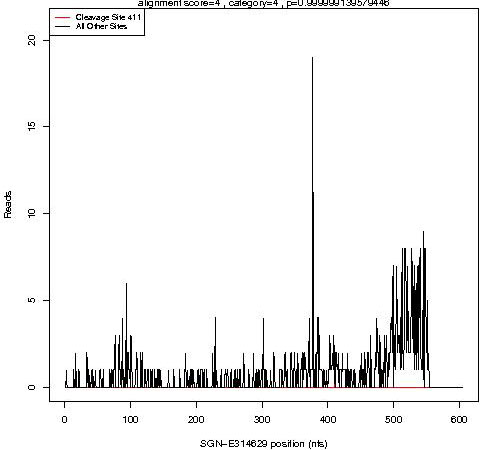


sha-miR156i-p3_nta slicing SGN-E314629 at nt 411


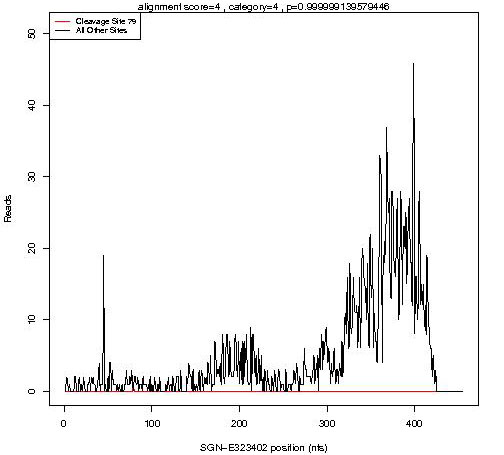


sha-miR156i-p3_nta slicing SGN-E323402 at nt 79

sha-miR156i-p3_nta slicing SGN-E720283 at nt 708


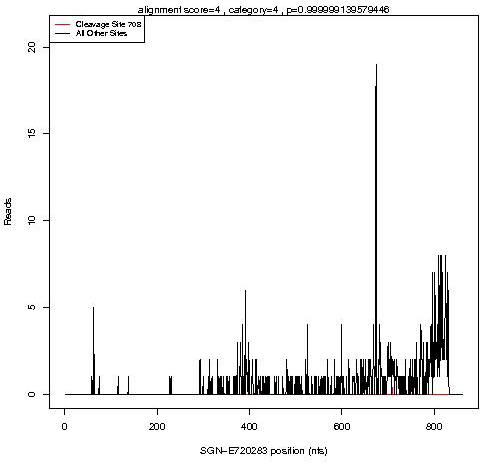

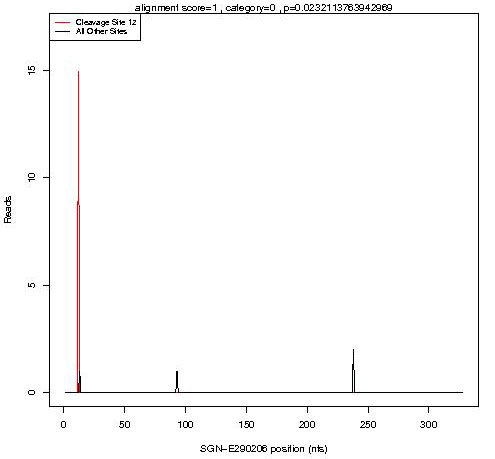


sha-miR160aslicing SGN-E290206 at nt12


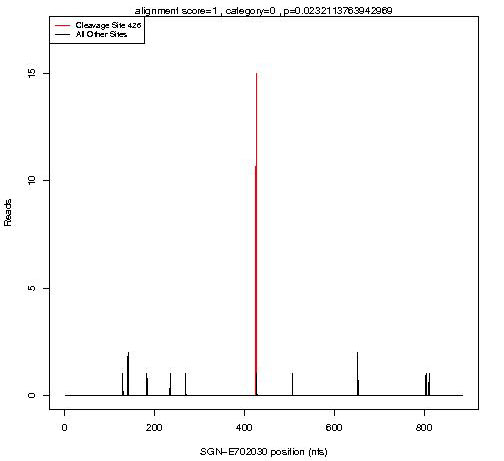


sha-miR160a slicing SGN-E702030 at nt 426


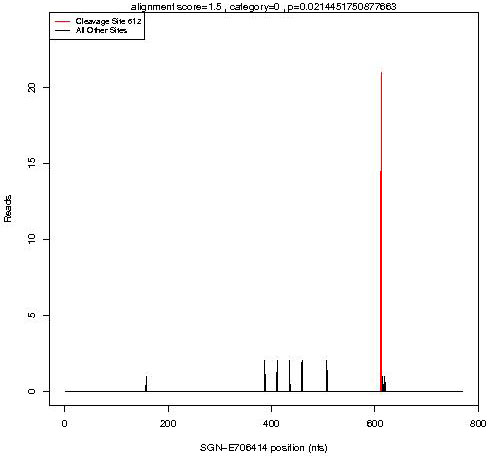


sha-miR160a slicing SGN-E706414 at nt612

sha-miR160a slicing SGN-E706426 at nt609


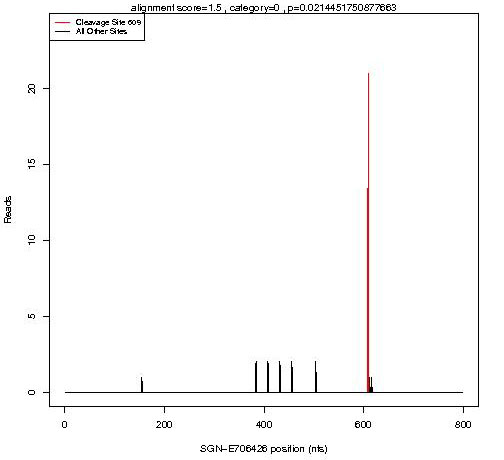


sha-miR164c-3p_aly slicing SGN-E273954 at nt 366


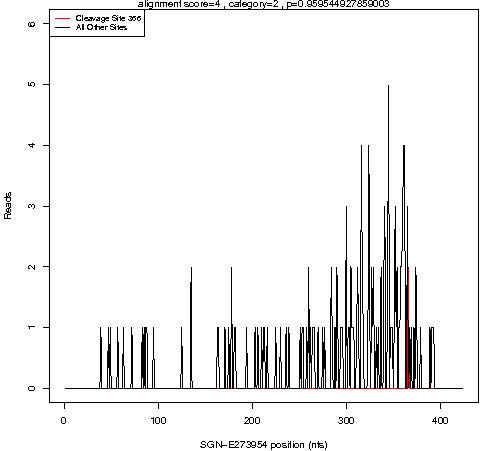

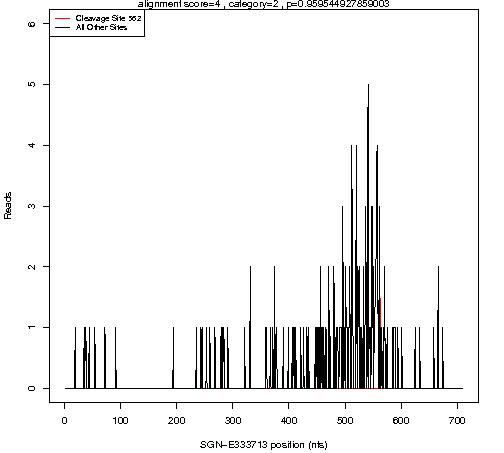


sha-miR164c-3p_aly slicing SGN-E333713 at nt 562

sha-miR164c-3p_aly slicing SGN-E698194 at nt 482


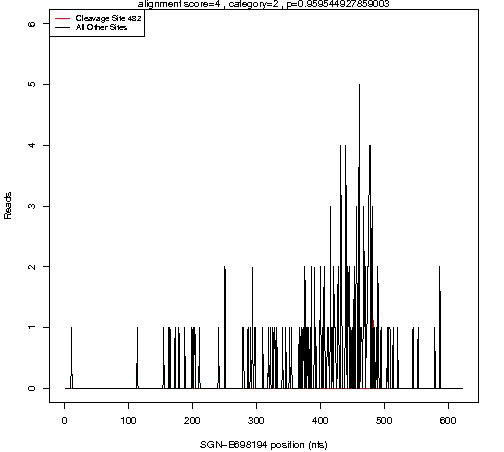

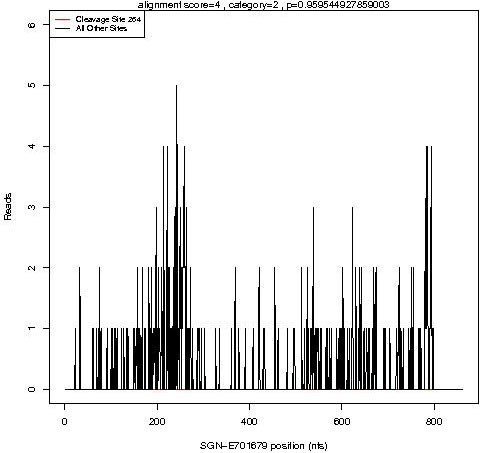


sha-miR164c-3p_aly slicing SGN-E701679 at nt 264

sha-miR164c-3p_aly slicing SGN-E703866 at nt 158


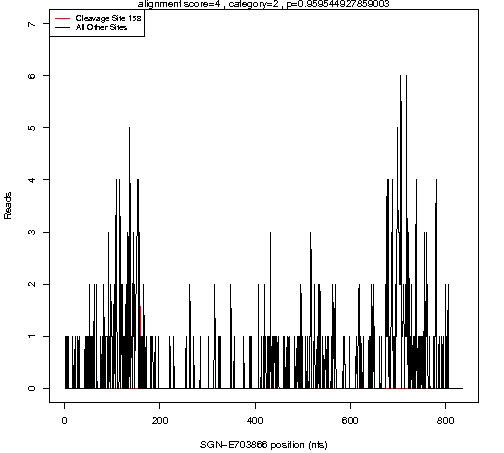


sha-miR164c-3p_aly slicing SGN-E710522 at nt 465


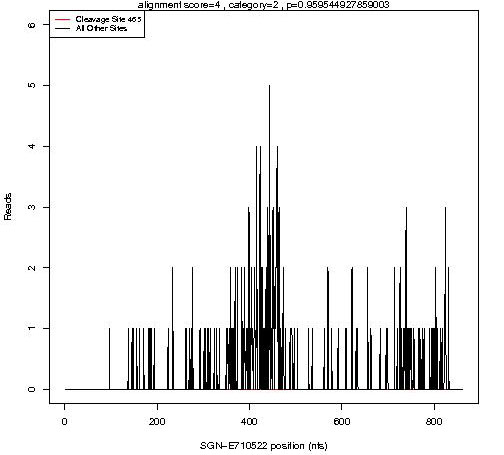


sha-miR164c-3p_aly slicing SGN-E719402 at nt 564


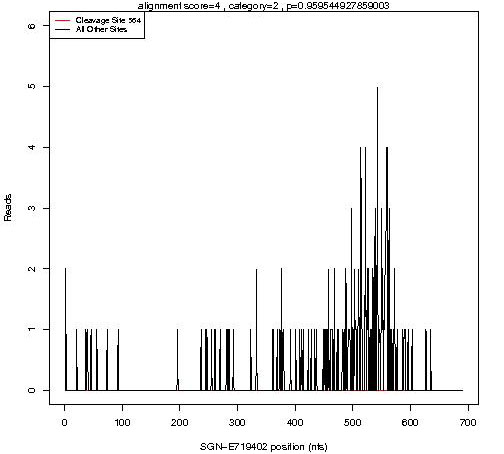


sha-miR164c-3p_aly slicing SGN-E745959 at nt 750


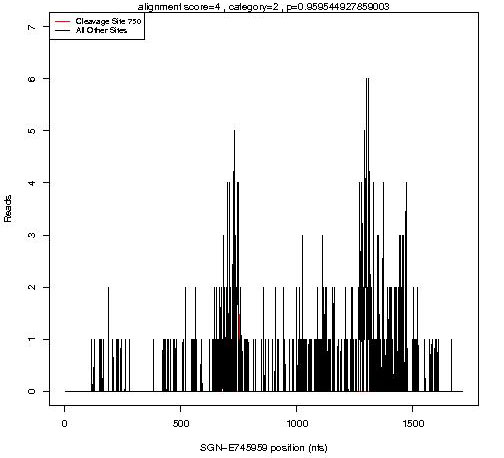

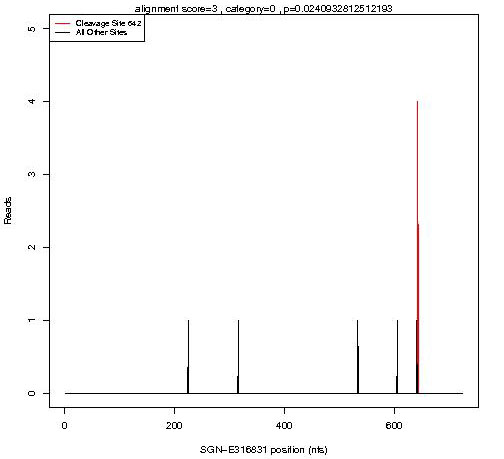


sha-miR166a and sha-miR166b slicing SGN-E316831 at nt642


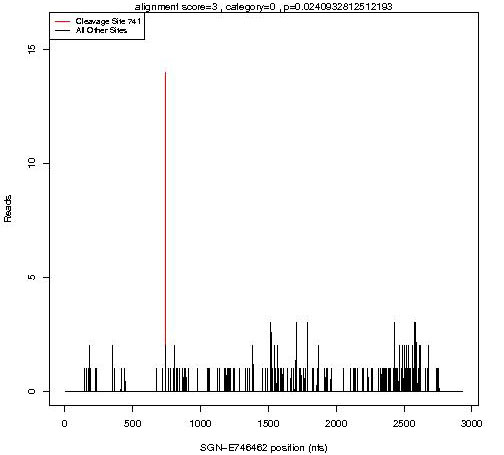


sha-miR166a and sha-miR166b slicing SGN-E746462 at nt741


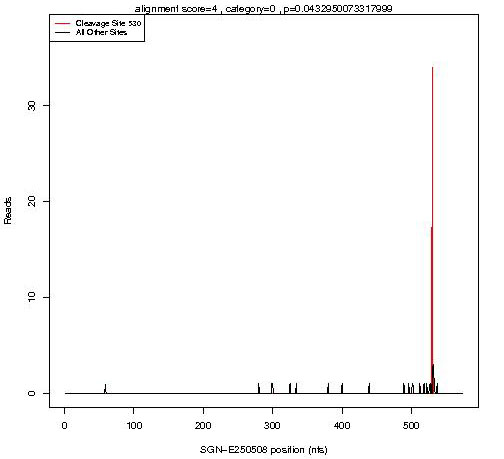


sha-miR167b_nta slicing SGN-E250508 at nt530


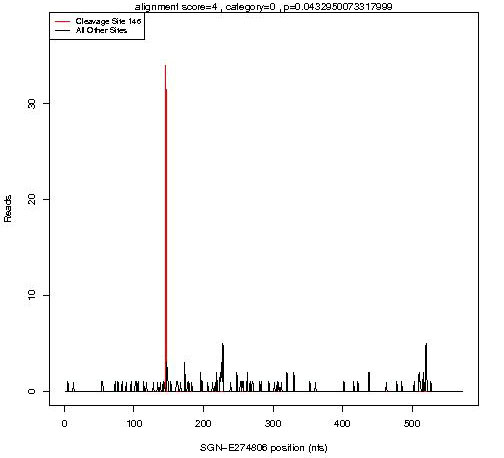


sha-miR167b_nta slicing SGN-E274806 at nt 146

sha-miR167b_nta slicing SGN-E550697 at nt 125


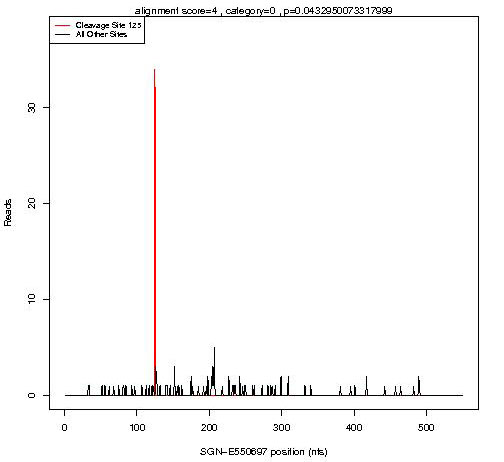

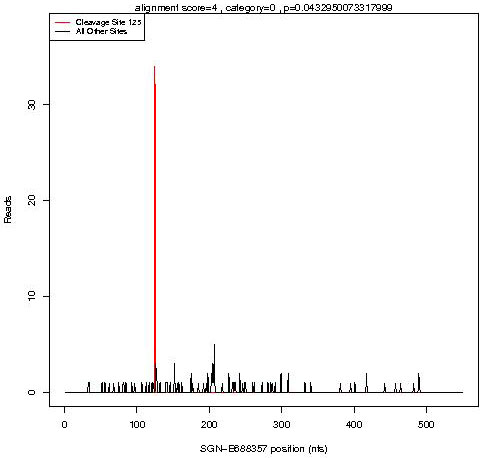


sha-miR167b_nta slicing SGN-E688357 at nt 125

sha-miR167b_nta slicing SGN-E707302 at nt545


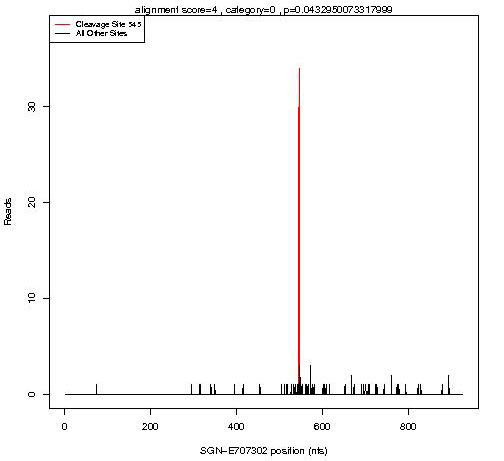


sha-miR168a-5p and sha-miR168b-5p slicing SGN-E740127 at nt517


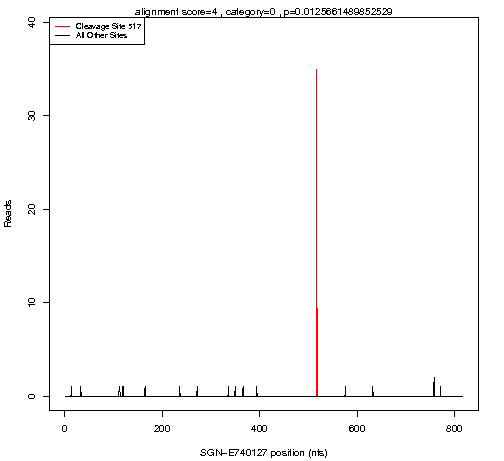

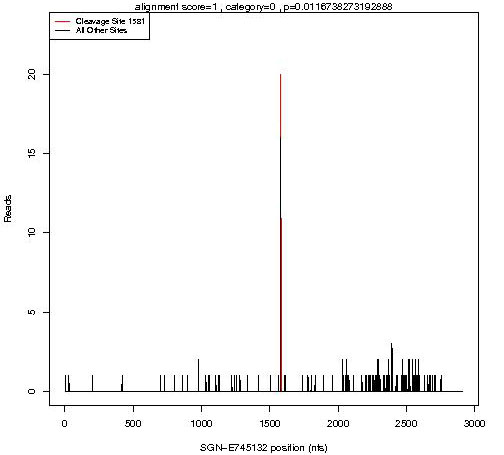


sha-miR171a slicing SGN-E745132 at nt1581

sha-miR171a_nta slicing SGN-E745132 at nt1581


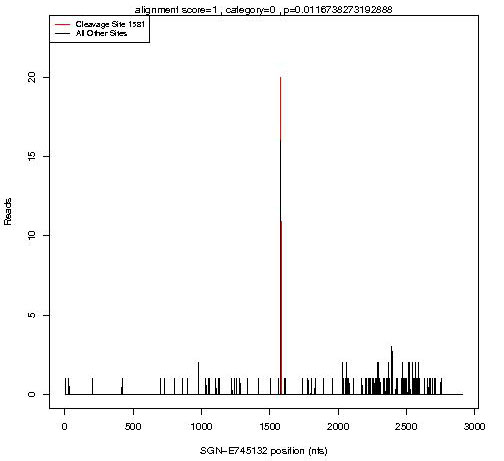


sha-miR171b-3p_stu slicing SGN-E745132 at nt 1578


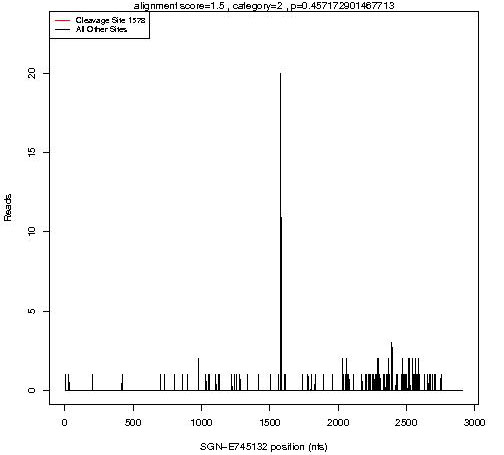


sha-miR156i-p3_nta slicing SGN-E211259 at nt 284

sha-miR171b-p3_mes slicing SGN-E745132 at nt1581


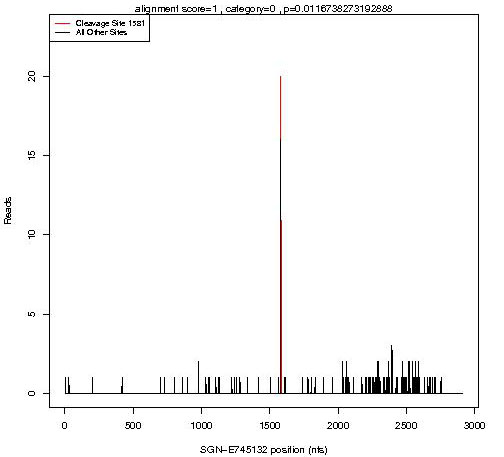

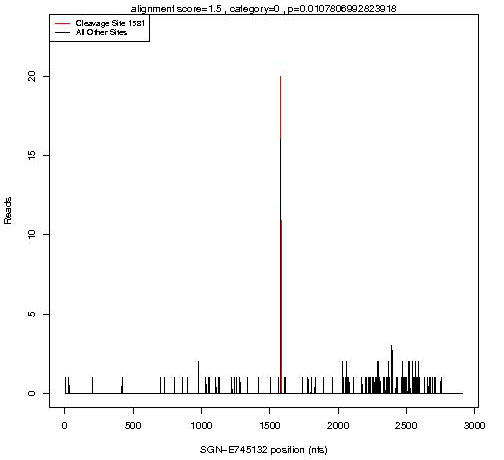


sha-miR171c_mtr slicing SGN-E745132 at nt1581

sha-miR171c-3p_stu slicing SGN-E745132 at nt1581


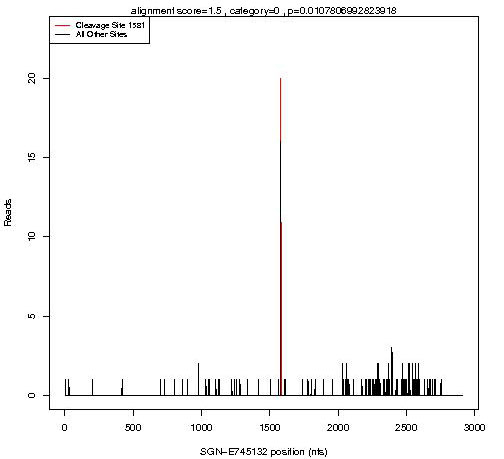

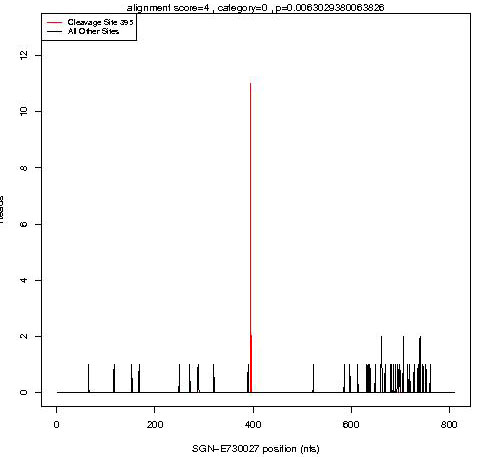


sha-miR171d slicing SGN-E730027 at nt395

sha-miR171d slicing SGN-E745132 at nt 1578


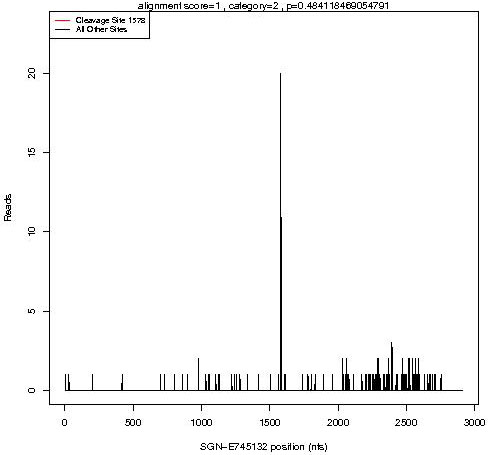


sha-miR172a and sha-miR172b slicing SGN-E377981 at nt 631


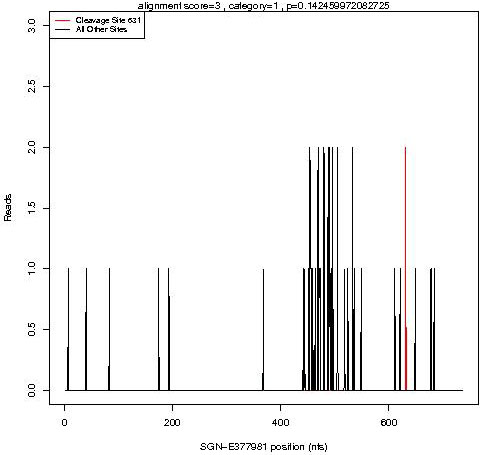


sha-miR172a and sha-miR172b slicing SGN-E745815 at nt 1569


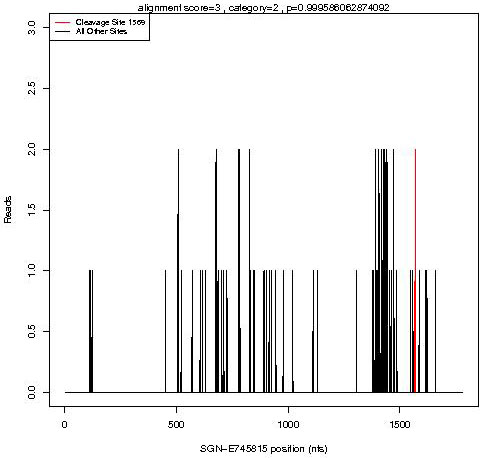


sha-miR172i_nta slicing SGN-E377981 at nt 631


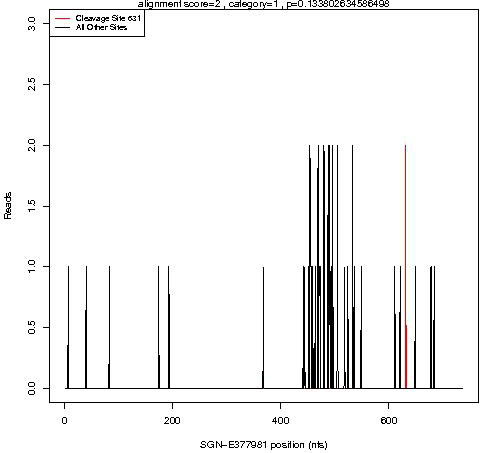


sha-miR172i_nta slicing SGN-E745815 at nt 1569


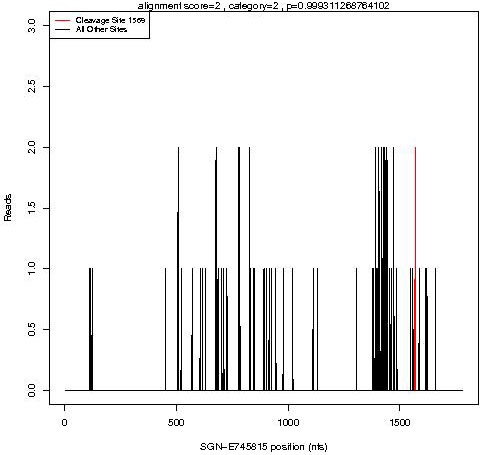


sha-miR172c-3p_aly slicing SGN-E377981 at nt 631


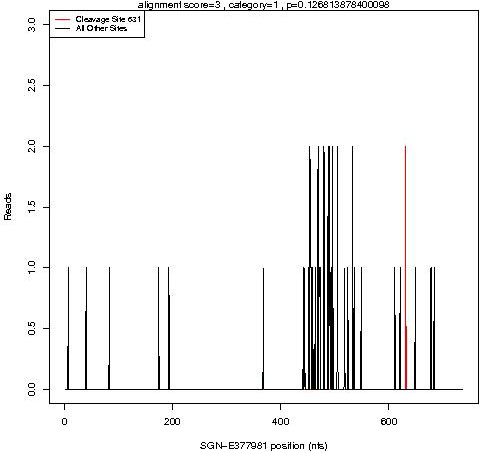


sha-miR319b_stu slicing SGN-E203353 at nt 502


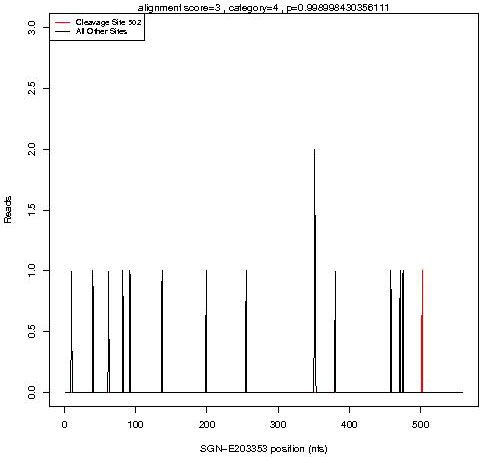


sha-miR172c-3p_aly slicing SGN-E745815 at nt 1569


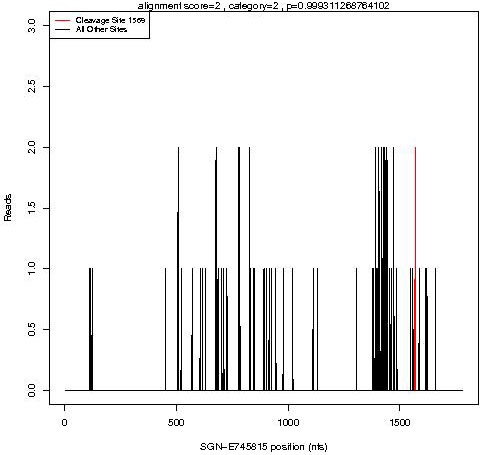

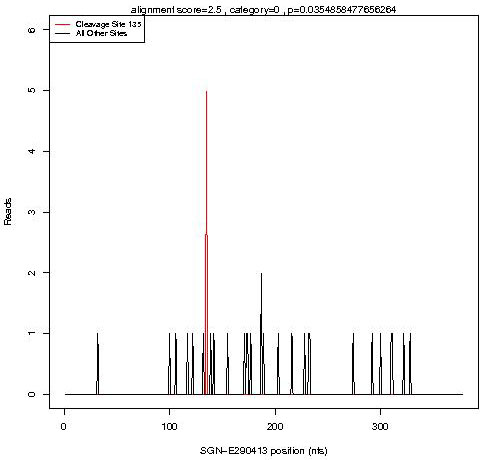


sha-miR319b_stu slicing SGN-E290413 at nt 135

sha-miR319b_stu slicing SGN-E339025 at nt 695


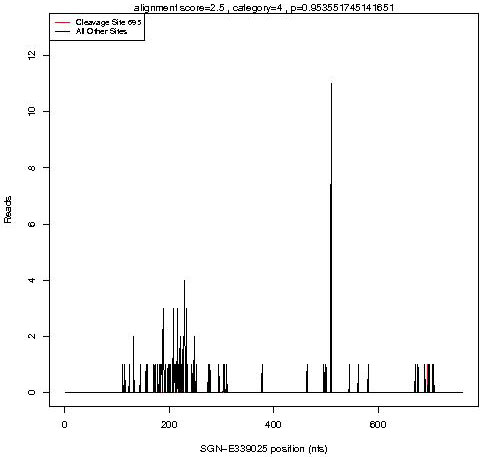


sha-miR319b_stu slicing SGN-E342729 at nt 167


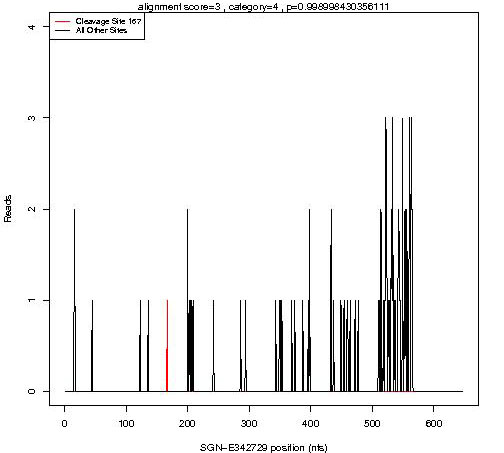


sha-miR319b_stu slicing SGN-E344717 at nt 495


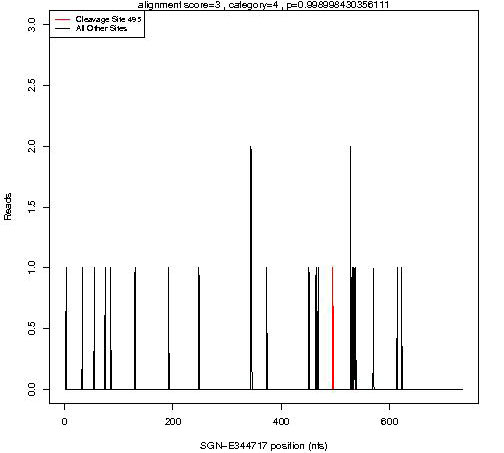

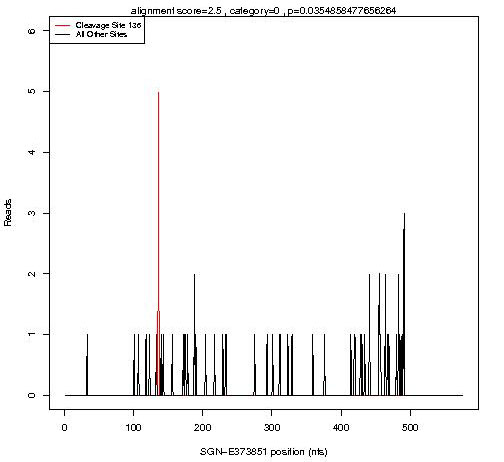


sha-miR319b_stu slicing SGN-E373851 at nt 136


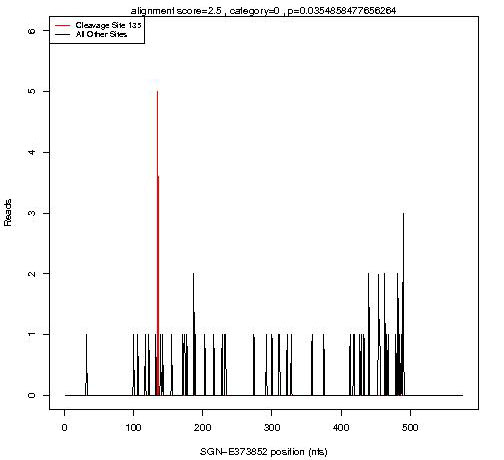


sha-miR319b_stu slicing SGN-E373852 at nt 135

sha-miR319b_stu slicing SGN-E700760 at nt 250


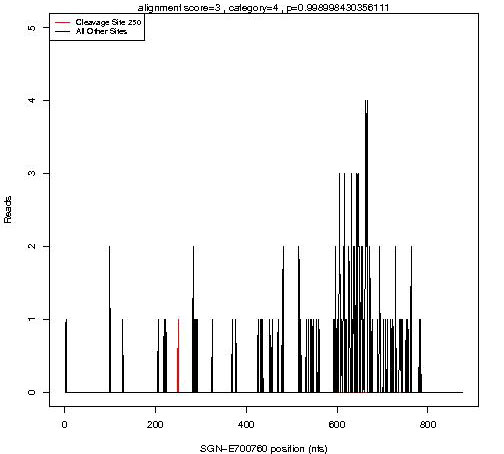


sha-miR319b_stu slicing SGN-E723238 at nt 786


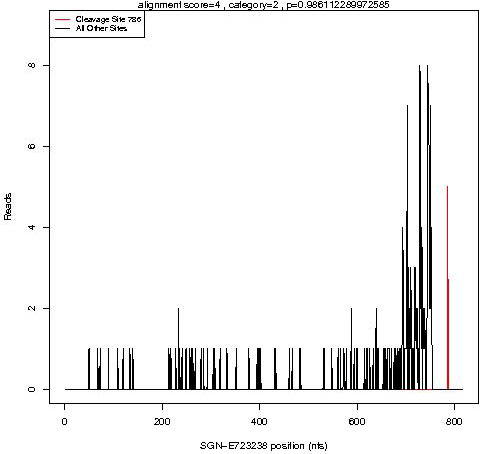


sha-miR319b_stu slicing SGN-E746988 at nt 1367


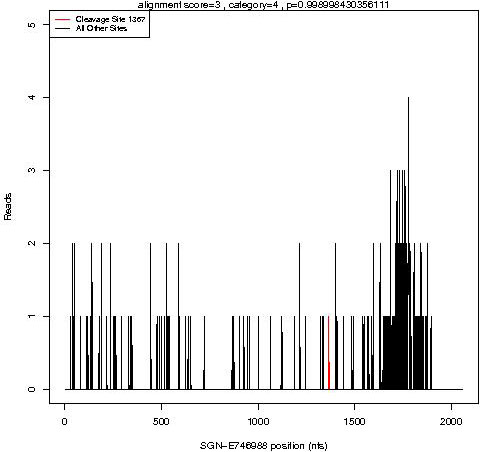


sha-miR319b_stu slicing SGN-E747253 at nt 2209


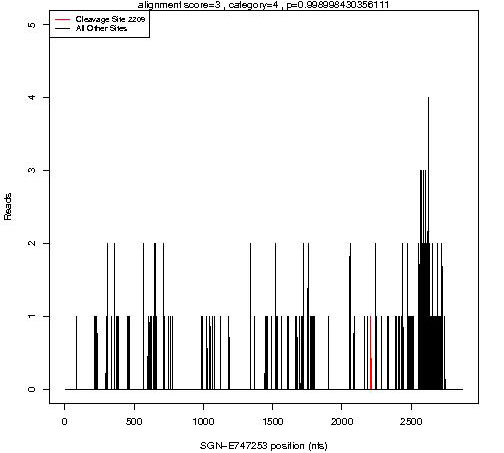

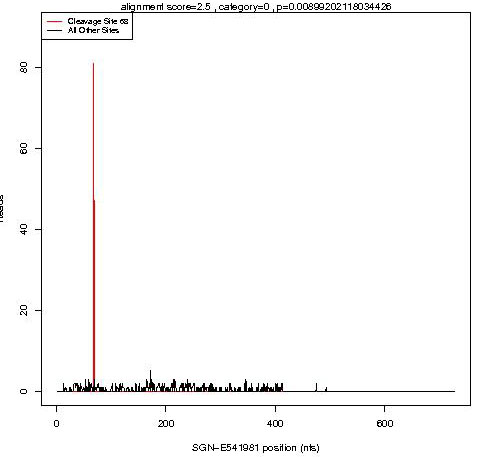


sha-miR393-5p_stuslicing SGN-E541981 at nt 68

sha-miR396a_nta slicing SGN-E203526 at nt 402


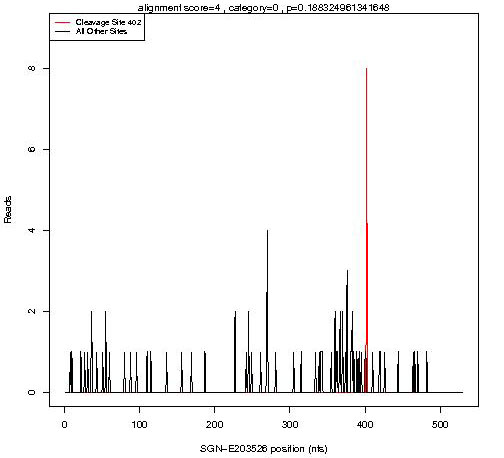


sha-miR396a_nta slicing SGN-E208738 at nt 149


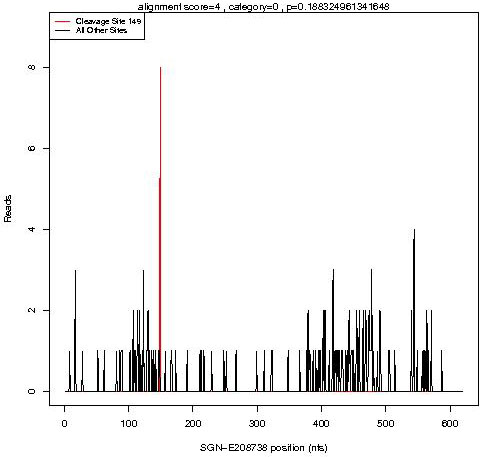


sha-miR396a_nta slicing SGN-E253668 at nt 246


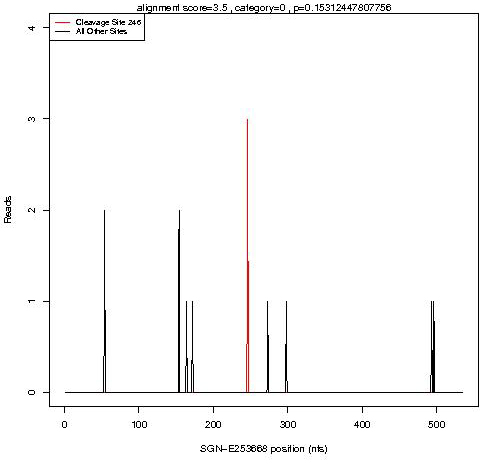


sha-miR396a_nta slicing SGN-E253713 at nt 80


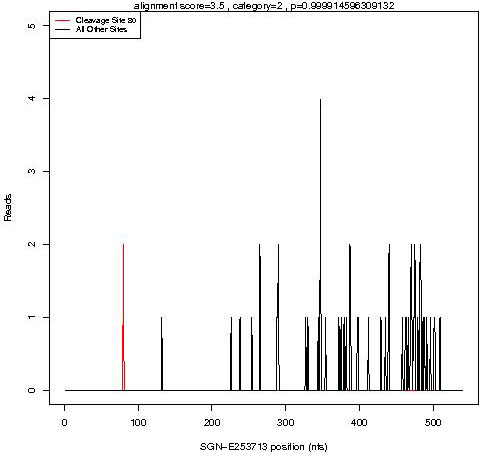


sha-miR396a_nta slicing SGN-E255531 at nt 251


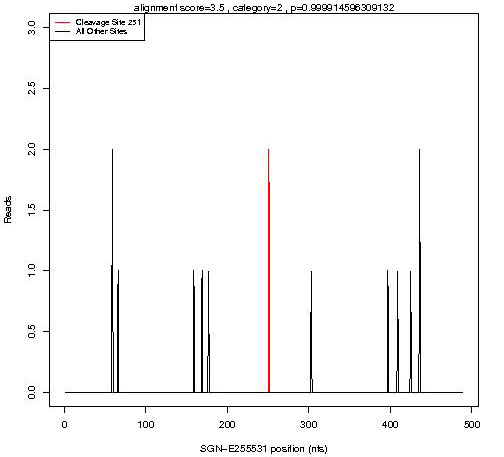


sha-miR396a_nta slicing SGN-E257588 at nt 248


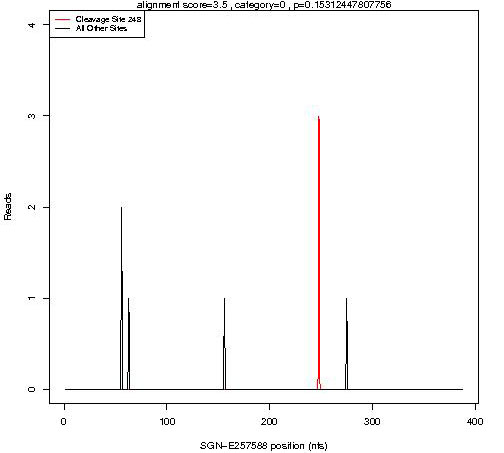


sha-miR396a_nta slicing SGN-E257650 at nt 251


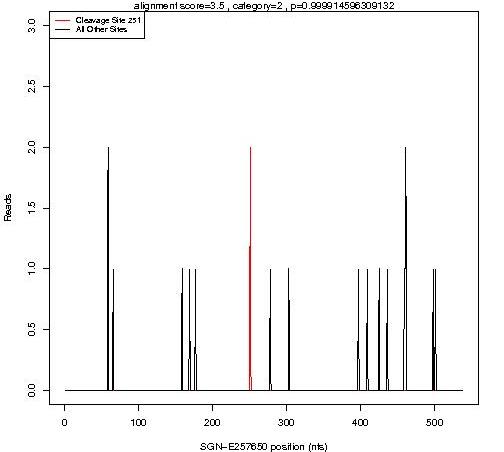


sha-miR396a_nta slicing SGN-E259324 at nt 251


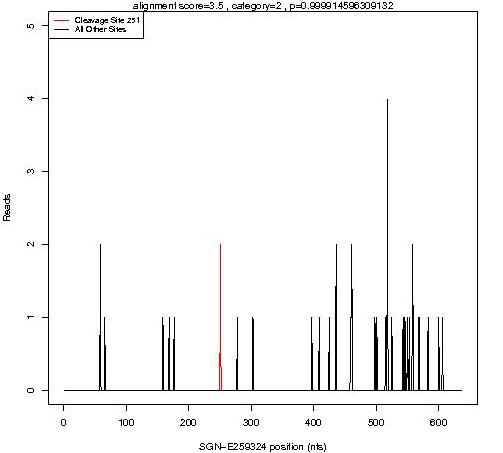


sha-miR396a_nta slicing SGN-E261365 at nt 251


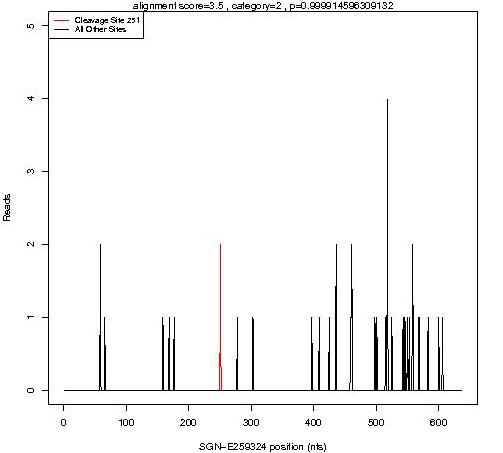


sha-miR396a_nta slicing SGN-E261582 at nt 245


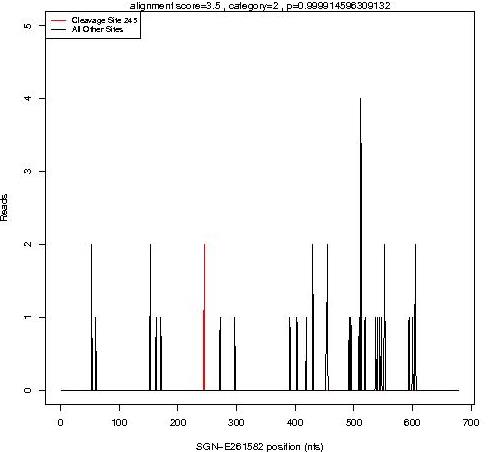


sha-miR396a_nta slicing SGN-E270049 at nt 466


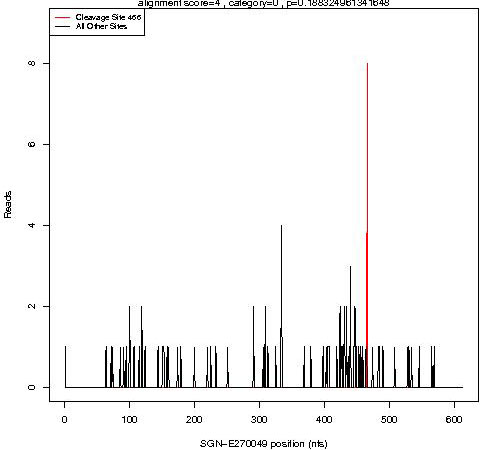


sha-miR396a_nta slicing SGN-E284673 at nt 374


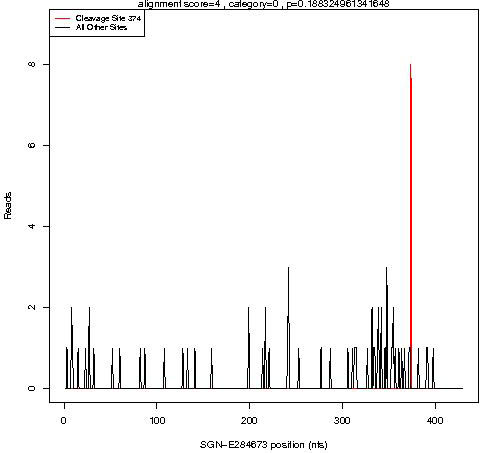


sha-miR396a_nta slicing SGN-E334427 at nt 646


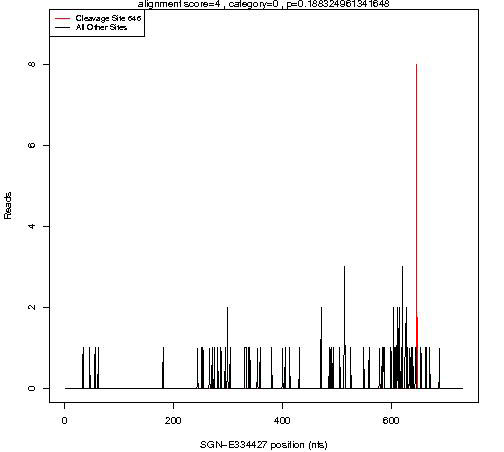


sha-miR396a_nta slicing SGN-E369054 at nt 251


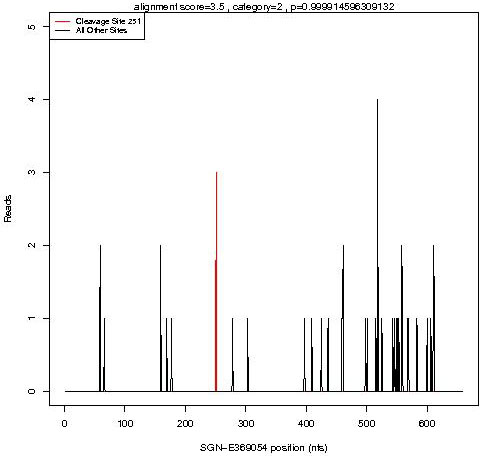


sha-miR396a_nta slicing SGN-E392273 at nt 252


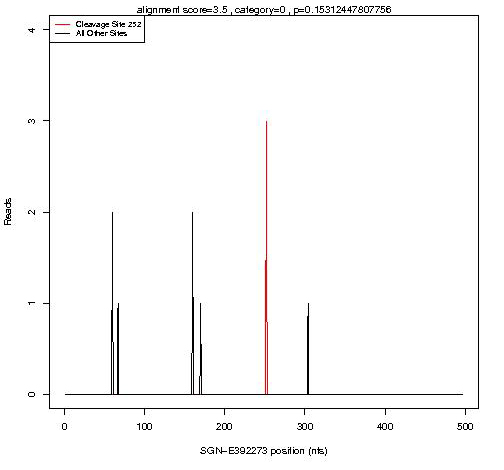


sha-miR396a_nta slicing SGN-E392304 at nt 252


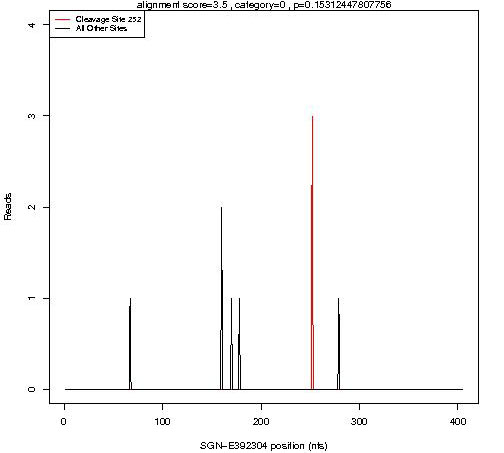


sha-miR396a_nta slicing SGN-E392323 at nt 252


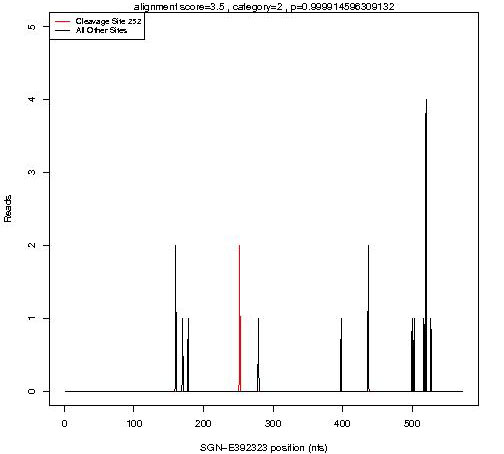


sha-miR396a_nta slicing SGN-E395508 at nt 437


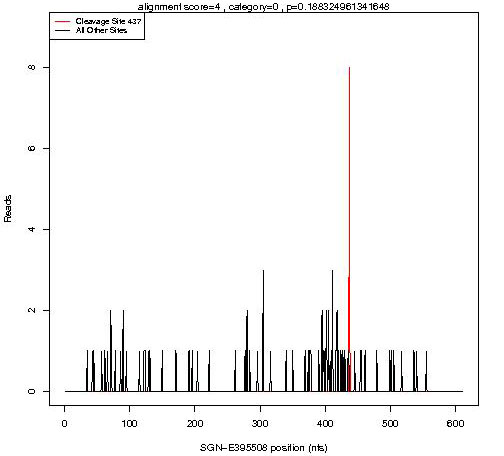


sha-miR396a_nta slicing SGN-E707067 at nt 133


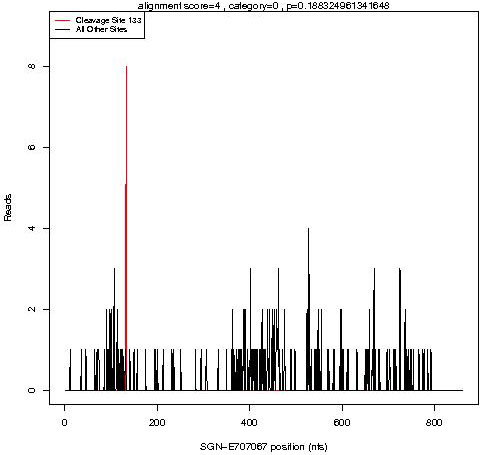


sha-miR396b_nta slicing SGN-E200713 at nt 115


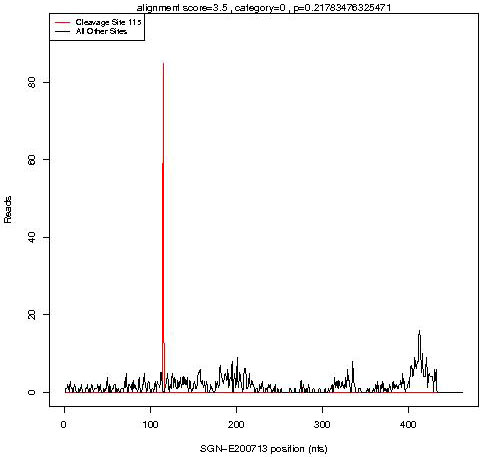

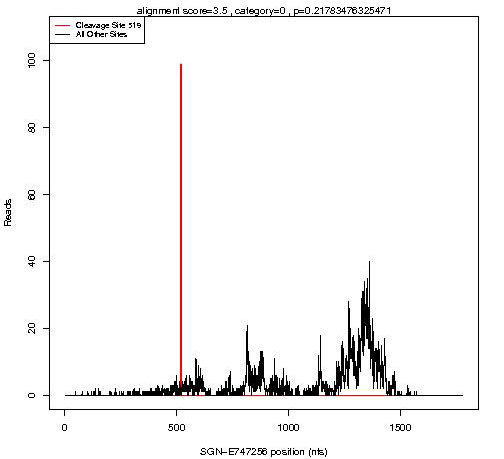


sha-miR396b_nta sling SGN-E747256 at nt 519

sha-miR396b_nta slicing SGN-E205705 at nt 273


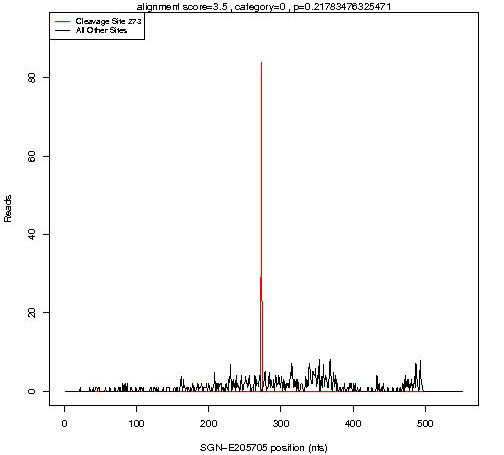


sha-miR396b_nta slicing SGN-E214917 at nt 503


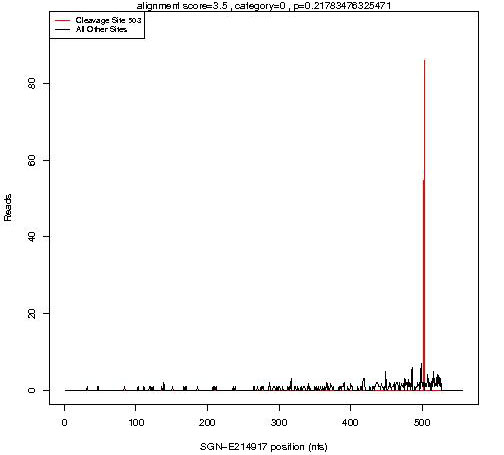


sha-miR396b_nta slicing SGN-E253668 at nt 246


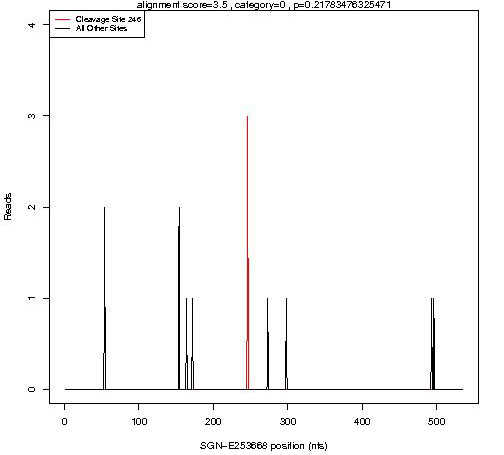


sha-miR396b_nta slicing SGN-E253713 at nt 80


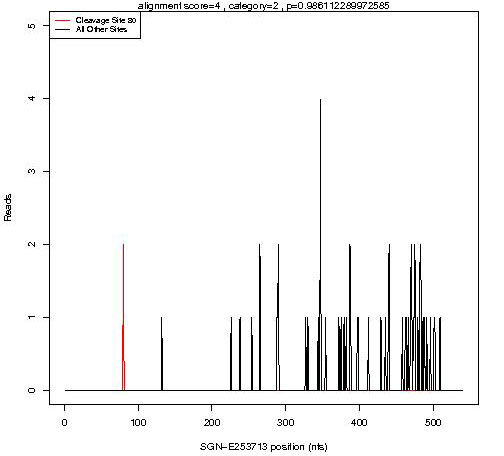


sha-miR396b_nta slicing SGN-E255531 at nt 251


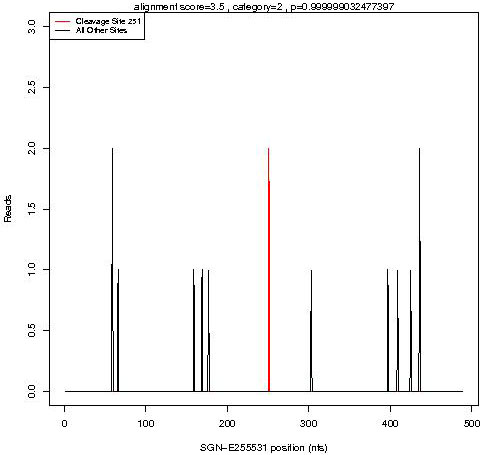


sha-miR396b_nta slicing SGN-E257588 at nt 248


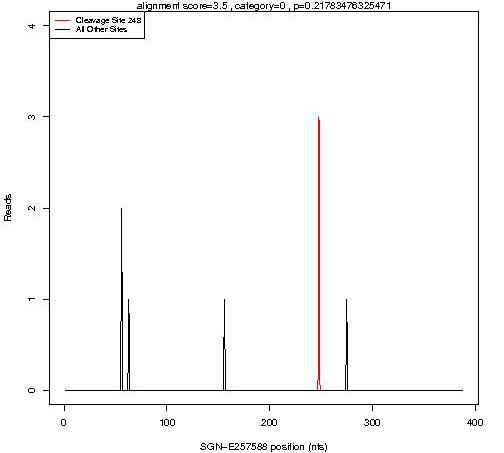


sha-miR396b_nta slicing SGN-E257650 at nt 251


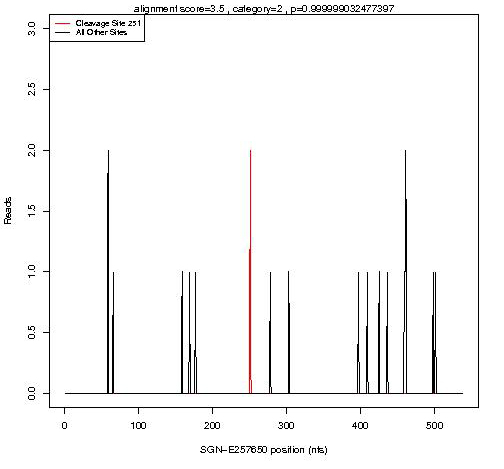


sha-miR396b_nta slicing SGN-E259324 at nt 251


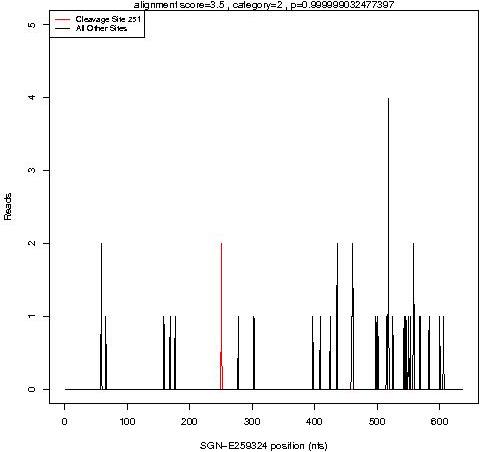


sha-miR396b_nta slicing SGN-E261365 at nt 251


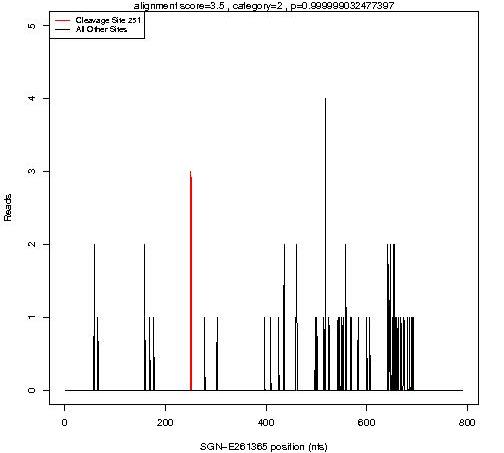


sha-miR396b_nta slicing SGN-E261582 at nt 245


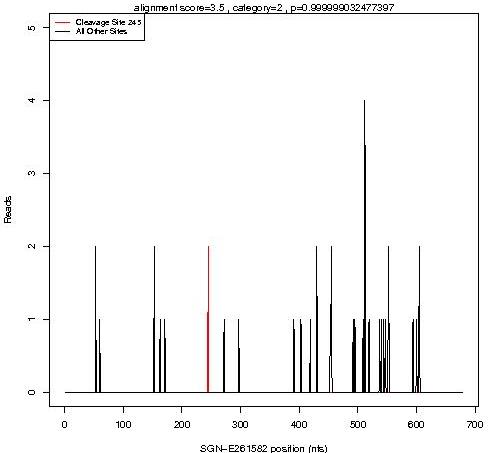


sha-miR396b_nta slicing SGN-E265911 at nt 504


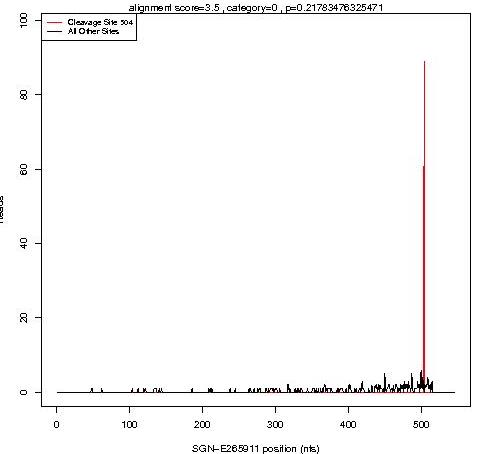


sha-miR396b_nta slicing SGN-E266983 at nt 356


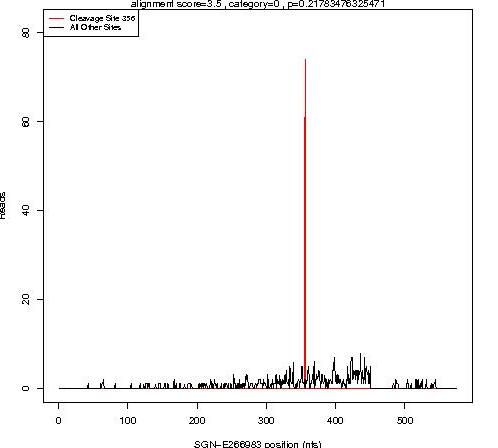


sha-miR396b_nta slicing SGN-E298573 at nt 214


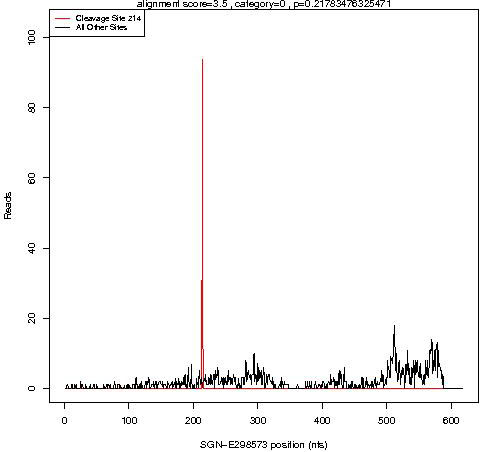


sha-miR396b_nta slicing SGN-E299086 at nt 100


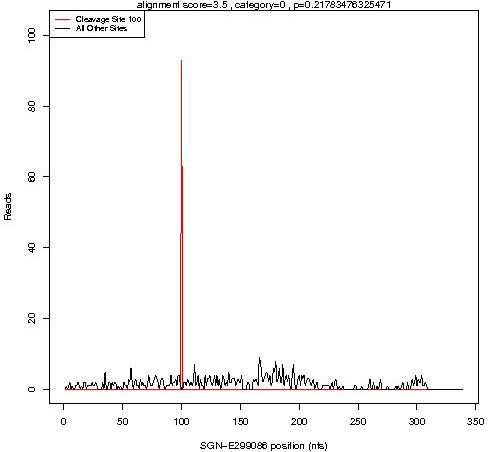


sha-miR396b_nta slicing SGN-E301892 at nt 434


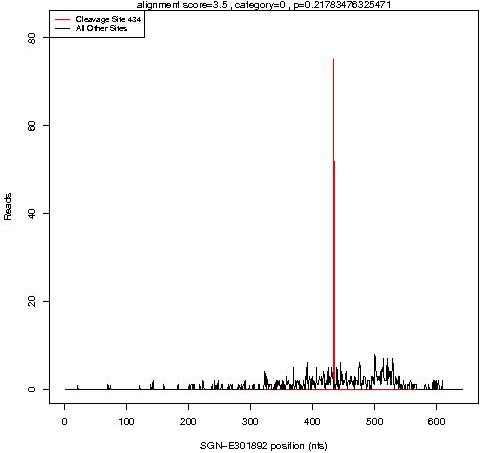


sha-miR396b_nta slicing SGN-E304743 at nt 368


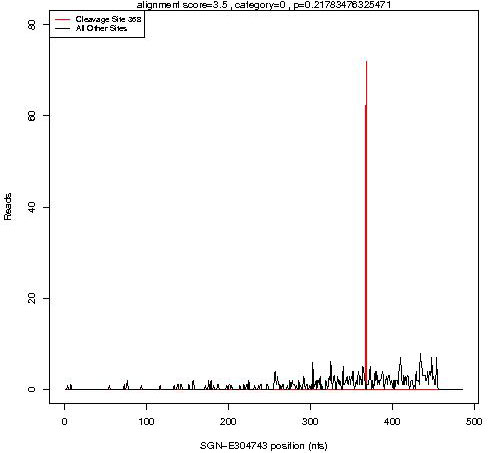


sha-miR396b_nta slicing SGN-E306168 at nt 494


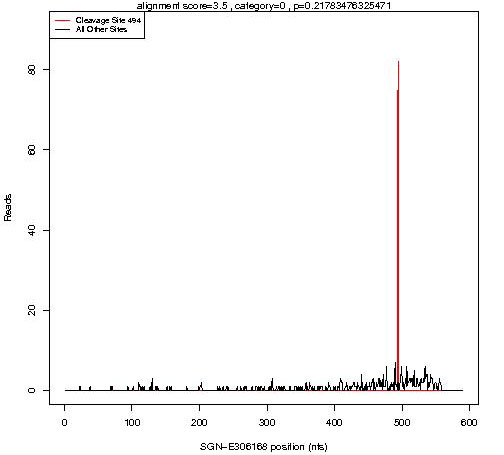


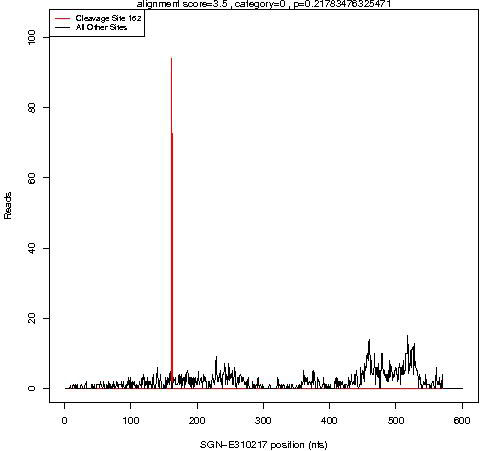


sha-miR396b_nta slicing SGN-E310217 at nt 162

sha-miR396b_nta slicing SGN-E322663 at nt 499

sha-miR396b_nta slicing SGN-E332208 at nt 496

sha-miR396b_nta slicing SGN-E334682 at nt 499

sha-miR396b_nta slicing SGN-E369054 at nt 251

sha-miR396b_nta slicing SGN-E392273 at nt 252

sha-miR396b_nta slicing SGN-E392304 at nt 252

sha-miR396b_nta slicing SGN-E392323 at nt 252

sha-miR396b_nta slicing SGN-E744501 at nt 112

sha-miR396b_nta slicing SGN-E744974 at nt 500

sha-miR396c_nta slicing SGN-E200713 at nt 115

sha-miR396c_nta slicing SGN-E205705 at nt 273

sha-miR396c_nta slicing SGN-E214917 at nt 503

sha-miR396c_nta slicing SGN-E253668 at nt246

sha-miR396c_nta slicing SGN-E253713 at nt 80

sha-miR396c_nta slicing SGN-E255531 at nt 251

sha-miR396c_nta slicing SGN-E257588 at nt 248

sha-miR396c_nta slicing SGN-E257650 at nt 251

sha-miR396c_nta slicing SGN-E259324 at nt 251

sha-miR396c_nta slicing SGN-E261365 at nt 251

sha-miR396c_nta slicing SGN-E261582 at nt 245

sha-miR396c_nta slicing SGN-E265911 at nt 504

sha-miR396c_nta slicing SGN-E266983 at nt 356

sha-miR396c_nta slicing SGN-E298573 at nt 214

sha-miR396c_nta slicing SGN-E299086 at nt 100

sha-miR396c_nta slicing SGN-E301892 at nt 434

sha-miR396c_nta slicing SGN-E304743 at nt 368

sha-miR396c_nta slicing SGN-E306168 at nt 494

sha-miR396c_nta slicing SGN-E310217 at nt 162

sha-miR396c_nta slicing SGN-E322663 at nt 499

sha-miR396c_nta slicing SGN-E332208 at nt 496

sha-miR396c_nta slicing SGN-E334682 at nt 499

sha-miR396c_nta slicing SGN-E369054 at nt251

sha-miR396c_nta slicing SGN-E392273 at nt 252

sha-miR396c_nta slicing SGN-E392304 at nt 252

sha-miR396c_nta slicing SGN-E392323 at nt252

sha-miR396c_nta slicing SGN-E744501 at nt112

sha-miR396c_nta slicing SGN-E744974at nt500

sha-miR396c_nta slicing SGN-E747256 at nt519

sha-miR398_nta slicing SGN-E232089 at nt 40

sha-miR398_nta slicing SGN-E233685 at nt 40

sha-miR398_nta slicing SGN-E246110 at nt 22

sha-miR398_nta slicing SGN-E261584 at nt 173

sha-miR398_nta slicing SGN-E279564 at nt40

sha-miR398_nta slicing SGN-E291338 at nt40

sha-miR398_nta slicing SGN-E332721 at nt29

sha-miR398_nta slicing SGN-E334772 at nt24

sha-miR398_nta slicing SGN-E344668 at nt 16

sha-miR398_nta slicing SGN-E345942 at nt35

sha-miR398_nta slicing SGN-E712604 at nt33

sha-miR398_nta slicing SGN-E713582 at nt23

sha-miR398_nta slicing SGN-E715403 at nt32

sha-miR398_nta slicing SGN-E716330 at nt34

sha-miR398_nta slicing SGN-E717993 at nt84

sha-miR398_nta slicing SGN-E718448 at nt 73

sha-miR398_nta slicing SGN-E720598 at nt55

sha-miR398_nta slicing SGN-E724642 at nt78

sha-miR408_ntaslicing SGN-E353197 at nt33

sha-miR408_nta slicing SGN-E354518 at nt30

sha-miR408_nta slicing SGN-E355169 at nt35

sha-miR408_nta slicing SGN-E356803 at nt 35

sha-miR408_nta slicing SGN-E552215 at nt 60

sha-miR482a_ghr slicing SGN-E236981 at nt 144

sha-miR482a_ghr slicing SGN-E352352 at nt 100

sha-miR482a_ghr slicing SGN-E355276 at nt83

sha-miR482a_ghr slicing SGN-E704204 at nt731

sha-miR482a_ghr slicing SGN-E344668 at nt727

sha-miR482b slicing SGN-E739892 at nt 236

sha-miR482b slicing SGN-E740719 at nt477

sha-miR827_nta slicing SGN-E309503 at nt235

sha-miR827_nta slicing SGN-E331370 at nt242

sha-miR5301-p3 slicing SGN-E352352 at nt100

sha-miR5301-p3 slicing SGN-E236981 at nt 144

sha-miR5301-p3 slicing SGN-E355276 at nt83

sha-miR5301-p3 slicing SGN-E704204at nt731

sha-miR5301-p3 slicing SGN-E707598at nt727

sha-miR6024 slicing SGN-E248561 at nt 334

sha-miR6024 slicing SGN-E376022 at nt 553

sha-miR6025_stu slicing SGN-E272042 at nt 294

sha-miR6025_stu slicing SGN-E354630 at nt432

sha-miR6025_stu slicing SGN-E370409 at nt238

sha-miR6025_stu slicing SGN-E370410 at nt294

sha-miR6025_stu slicing SGN-E389714 at nt446

sha-miR6025_stu slicing SGN-E550079 at nt447

sha-miR6025_stu slicing SGN-E550285 at nt447

sha-miR6025_stu slicing SGN-E550623 at nt336

sha-miR6025_stu slicing SGN-E551268 at nt295

sha-miR6025_stu slicing SGN-E551398 at nt295

sha-miR6025_stu slicing SGN-E688258 at nt295

sha-miR6025_stu slicing SGN-E688259 at nt336

sha-miR6025_stu slicingSGN-E688271 at nt295

sha-miR6025_stu slicingSGN-E711212 at nt367

sha-miR6027 slicing SGN-E204998 at nt 145

sha-miR6027 slicing SGN-E398442 at nt 146

PC-75-3p slicing SGN-E263621 at nt 661

PC-75-3p slicing SGN-E355515 at nt 467

PC-75-3p slicingSGN-E356830 at nt467

PC-75-3p slicingSGN-E356831 at nt653

PC-75-3p slicing SGN-E707134 at nt 782

PC-89-5pslicingSGN-E746319at nt69

PC-93-3p_stu slicingSGN-E226448 at nt43

PC-93-3p_stu slicingSGN-E287356 at nt297

PC-93-3p_stu slicingSGN-E703722at nt573

PC-93-3p_stu slicingSGN-E708964at nt556

PC-93-3p_stu slicingSGN-E713893at nt558

PC-102-5pslicingSGN-E233896at nt 98

PC-102-5pslicingSGN-E350418at nt 321

PC-102-5pslicingSGN-E351849at nt 415

PC-102-5pslicingSGN-E352584at nt415

PC-102-5pslicingSGN-E353944at nt 319

PC-102-5pslicingSGN-E701432at nt 798

PC-102-5pslicingSGN-E705552at nt 699

PC-102-5pslicingSGN-E713099at nt 271

PC-102-5pslicingSGN-E713896at nt 162

PC-117-5pslicingSGN-E720902at nt452

PC-125-3p slicingSGN-E294611 at nt458

PC-125-3p slicing SGN-E705851 at nt 765
