# Supplementary material for: Identification of chilling stress-responsive tomato microRNAs and their target genes by high-throughput sequencing and degradome analysis
Source: BMC Genomics. 2014 Dec 17;15(1):1130. doi: 10.1186/1471-2164-15-1130 (PMC4377850; doi:10.1186/1471-2164-15-1130)
Supplement: Supplementary file 7 — Additional file 7: Figure S4: - T-plots for targets of tomato miRNAs identified in the CT library. According to German et al, t-plots are referred to as “target plots” and the normalized numbers are used to plot the cleavages on target mRNAs. The frequency of degradome tags with 5’ends at the indicated positions is marked in black, and the frequency cleaved at position 10 of the inset miRNA target alignment is highlighted in red. The abscissa number t indicates the cleavage site detected in the target mRNA. (DOCX 13 MB) [file 12864_2014_6877_MOESM7_ESM.docx]

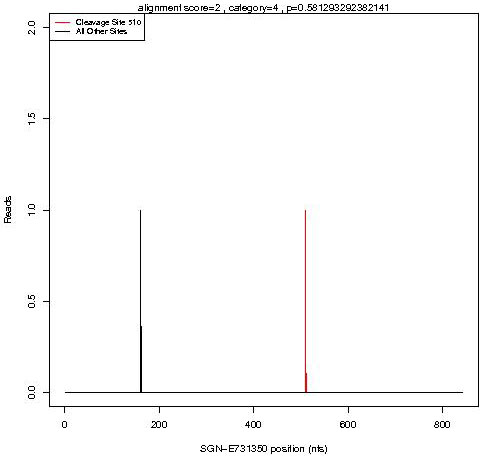


sha-miR156a and sha-miR156c slicing SGN-E731350 at nt 510

sha-miR156c_nta slicing SGN-E731350 at nt 510


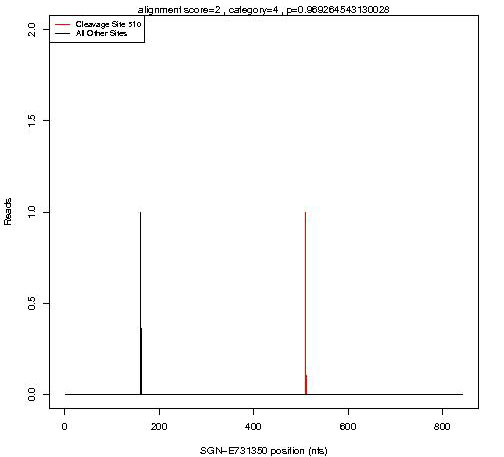

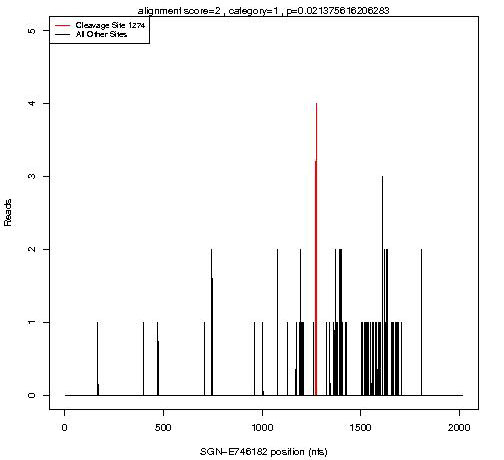


sha-miR156d_nta slicing SGN-E746182 at nt 1274

sha-miR156e_stu, sha-miR156g_stu,sha-miR156h_stu,sha-miR156i_stu and sha-miR156j_stu slicing SGN-E746182 at nt 1274


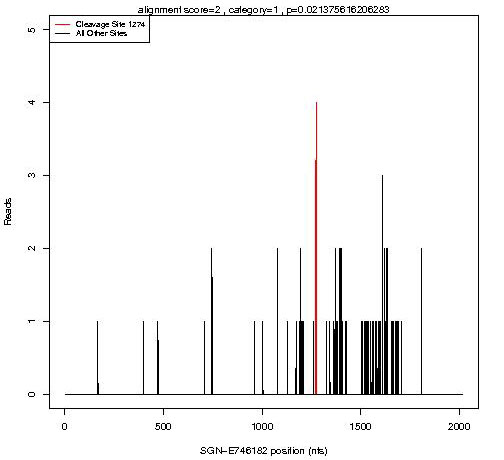

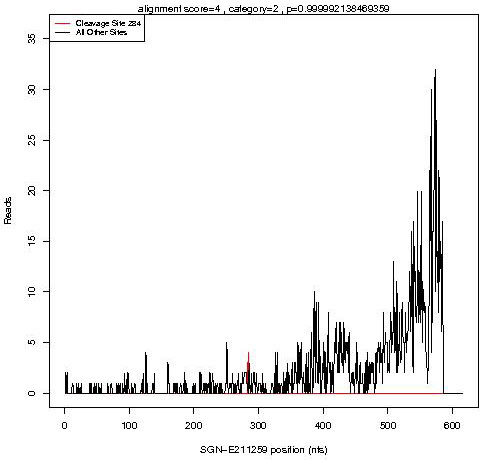


sha-miR156i-p3_nta slicing SGN-E211259 at nt 284


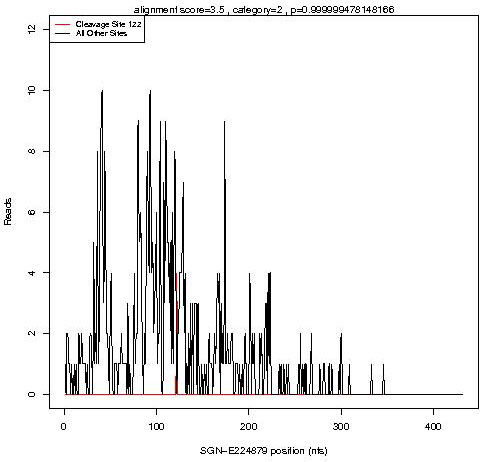


sha-miR156i-p3_nta slicing SGN-E224879 at nt 122

sha-miR156i-p3_nta slicing SGN-E236387 at nt 78


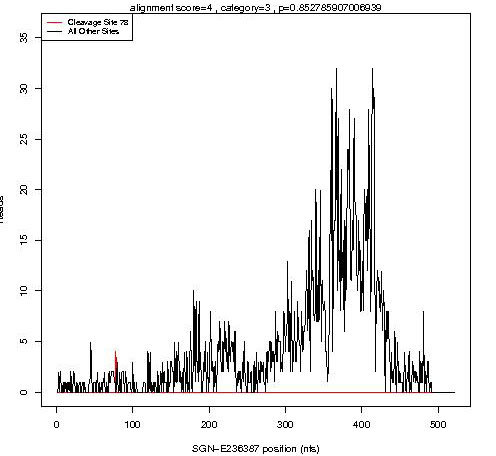

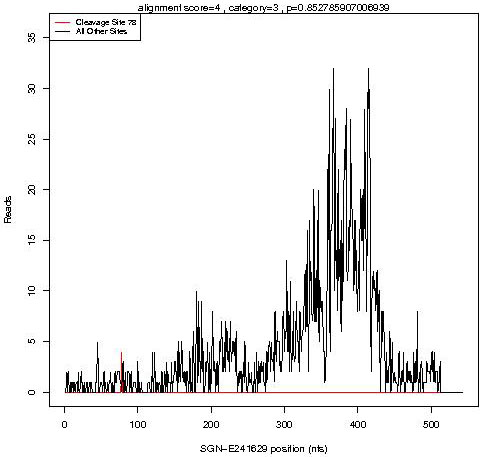


sha-miR156i-p3_nta slicing SGN-E241629 at nt 78

sha-miR156i-p3_nta slicing SGN-E246440 at nt 385


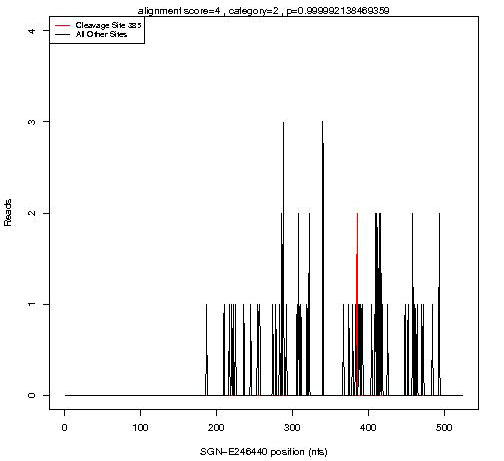

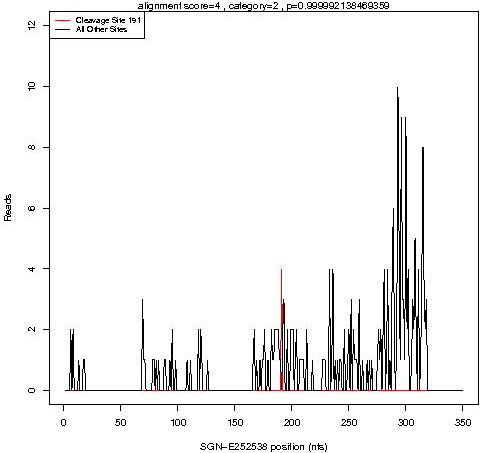


sha-miR156i-p3_nta slicing SGN-E252538 at nt 191


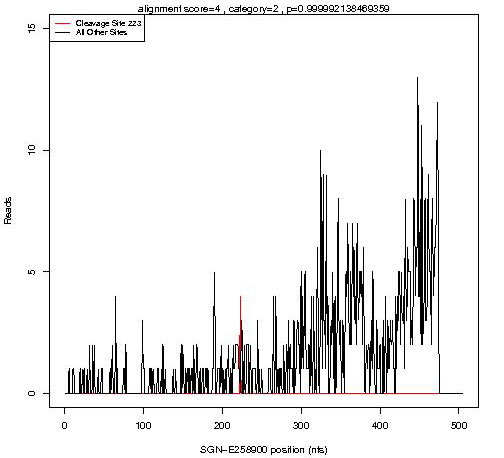


sha-miR156i-p3_nta slicing SGN-E258900 at nt 223

sha-miR156i-p3_nta slicing SGN-E297170 at nt 64


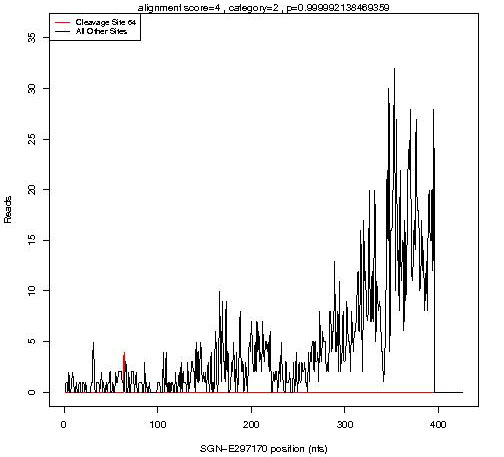

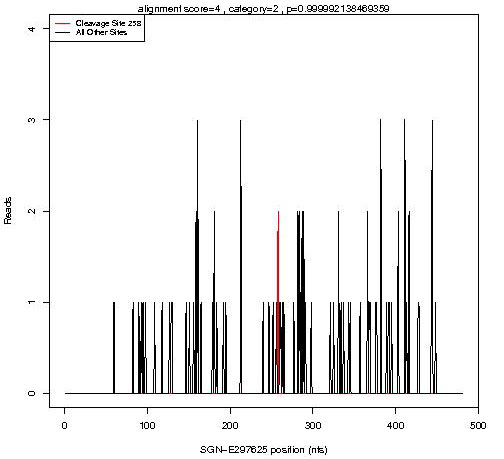


sha-miR156i-p3_nta slicing SGN-E297625 at nt 258


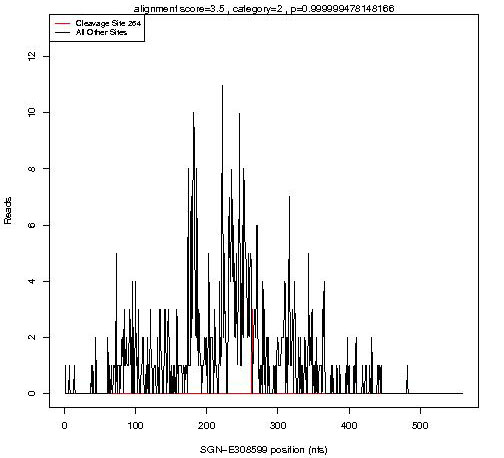


sha-miR156i-p3_nta slicing SGN-E308599 at 264

sha-miR156i-p3_nta slicing SGN-E311075 at 79


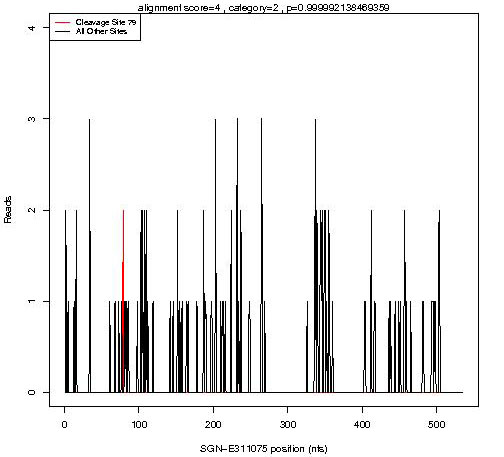


sha-miR156i-p3_nta slicing SGN-E314629 at nt 411


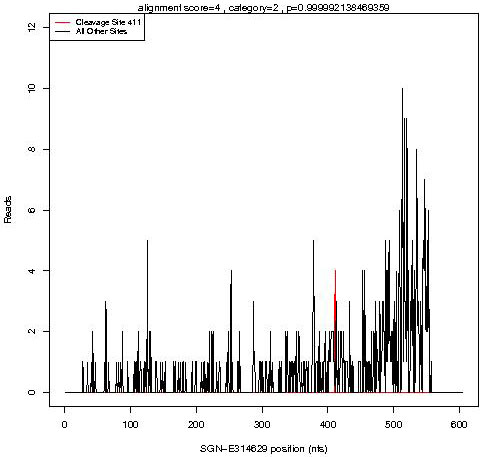


sha-miR156i-p3_nta slicing SGN-E323402 at nt 79


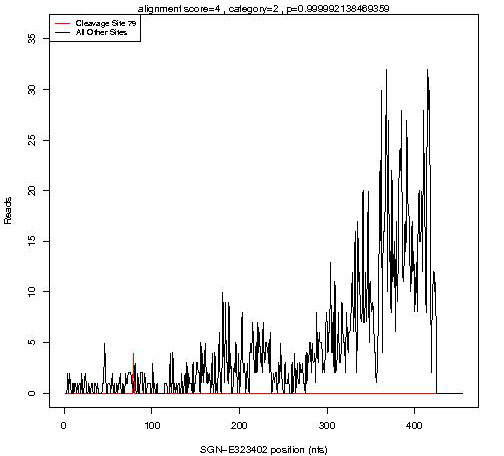


sha-miR156i-p3_nta slicing SGN-E340436 at nt 156


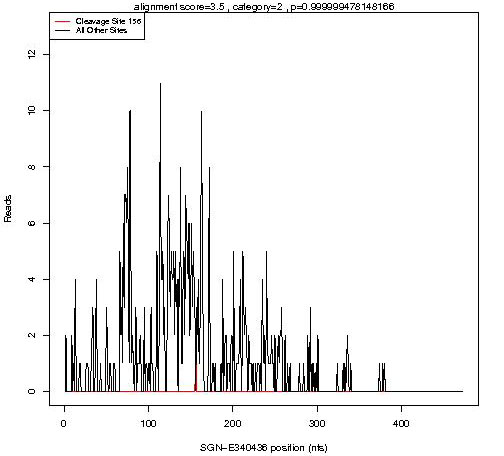


sha-miR156i-p3_nta slicing SGN-E344860 at nt 156


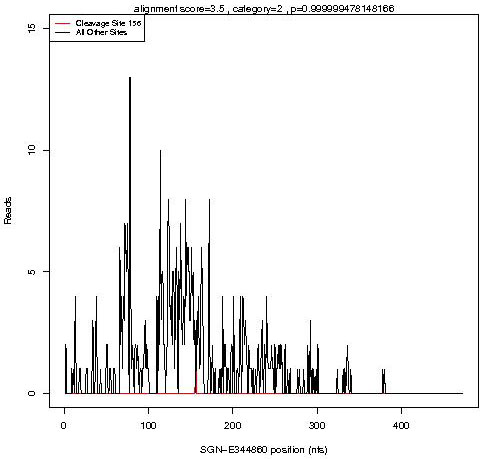


sha-miR156i-p3_nta slicing SGN-E345956 at nt 431


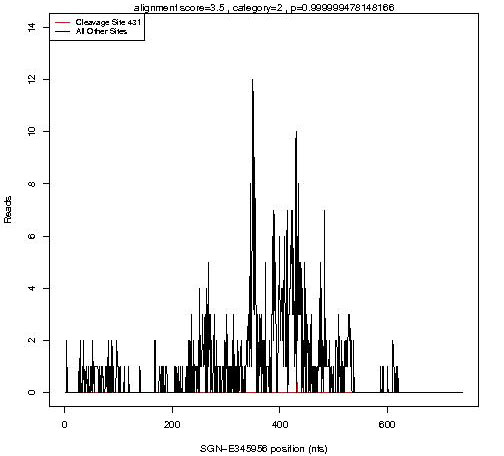


sha-miR156i-p3_nta slicing SGN-E346047 at nt 437


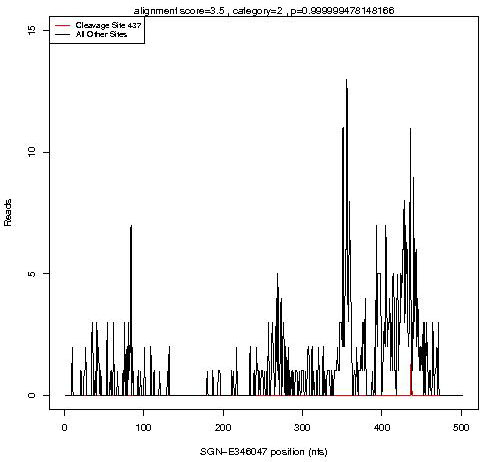


sha-miR156i-p3_nta slicing SGN-E348310 at nt 510


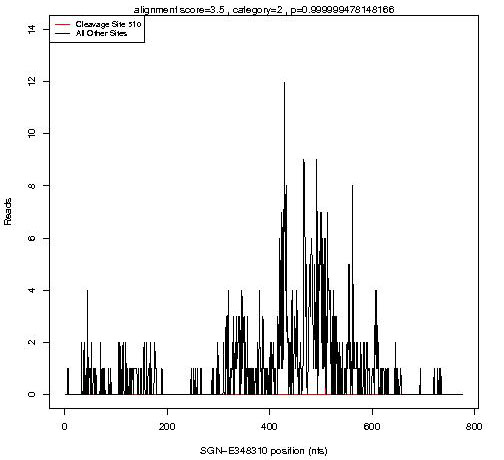

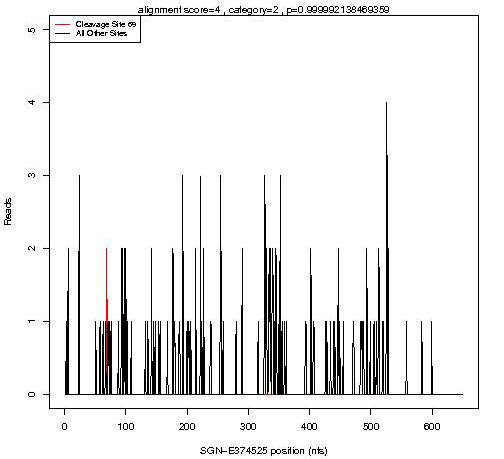


sha-miR156i-p3_nta slicing SGN-E374525 at 69

sha-miR156i-p3_nta slicing SGN-E374526 at 235


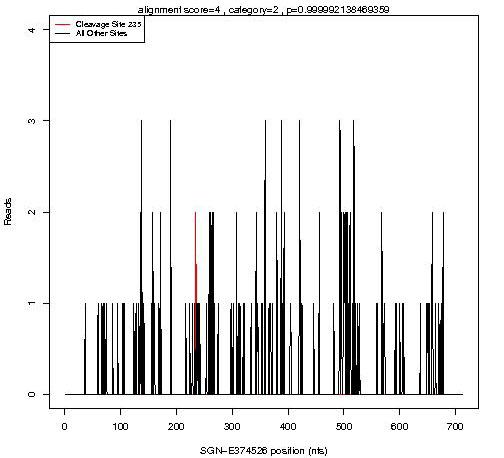


sha-miR156i-p3_nta slicing SGN-E711369 at 366


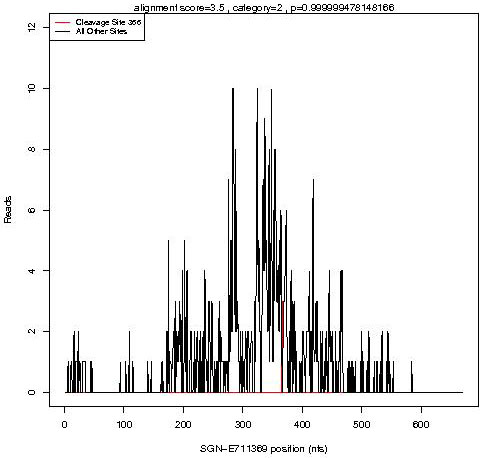


sha-miR156i-p3_nta slicing SGN-E712287 at 505


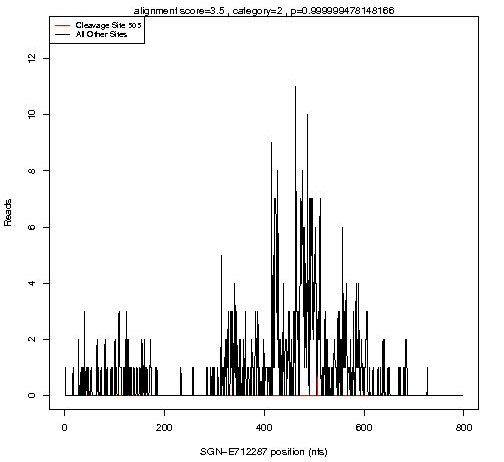


sha-miR156i-p3_nta slicing SGN-E712324 at 490


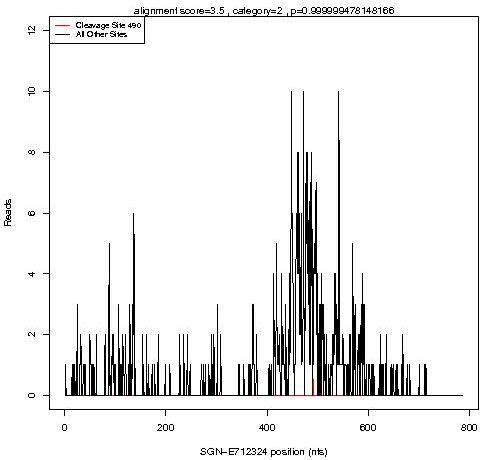


sha-miR156i-p3_nta slicing SGN-E716653 at 511


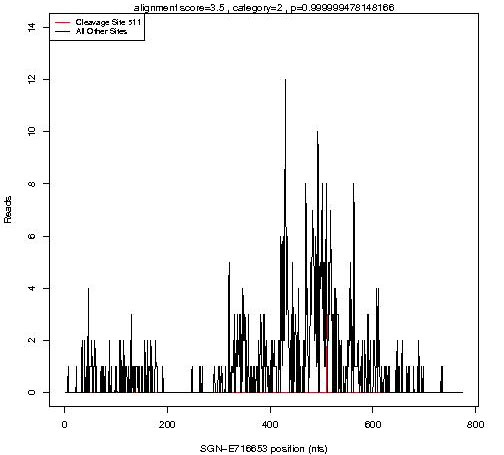

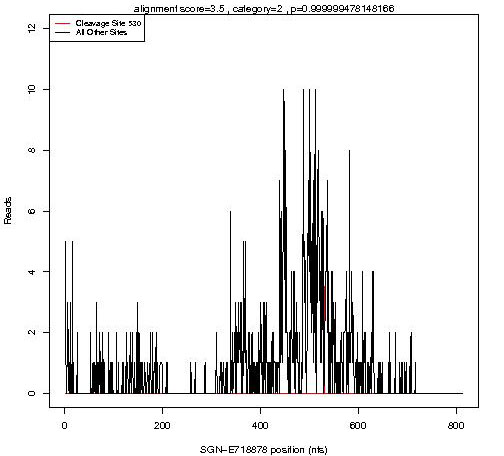


sha-miR156i-p3_nta slicing SGN-E718878 at 530


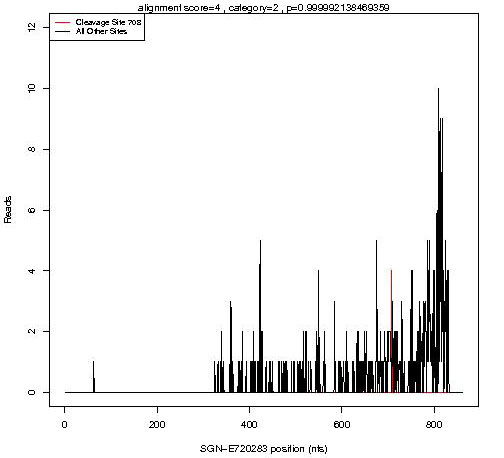


sha-miR156i-p3_nta slicing SGN-E720283 at nt 708

sha-miR156i-p3_nta slicing SGN-E722183 at 538


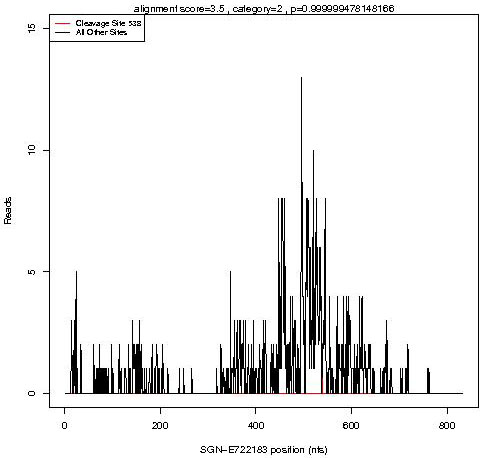

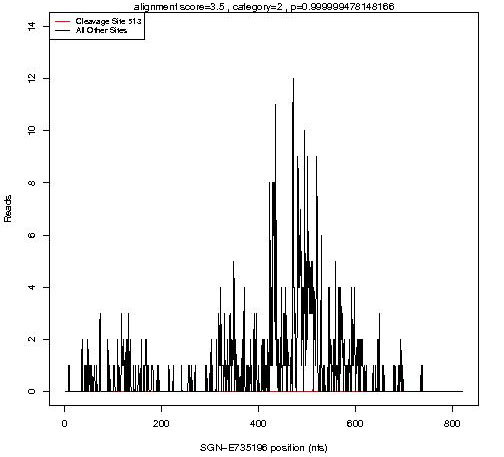


sha-miR156i-p3_nta slicing SGN-E735196 at 513


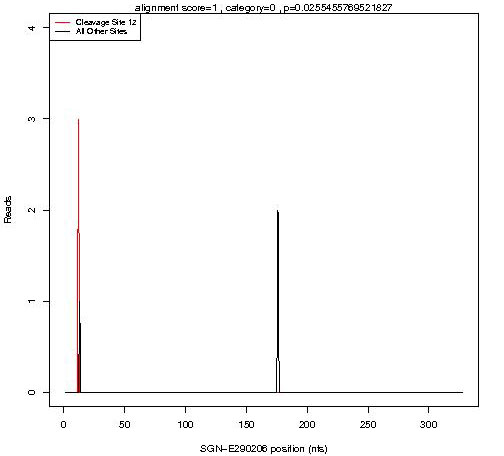


sha-miR160a slicing SGN-E290206 at nt 12

sha-miR160a slicing SGN-E702030 at nt 426


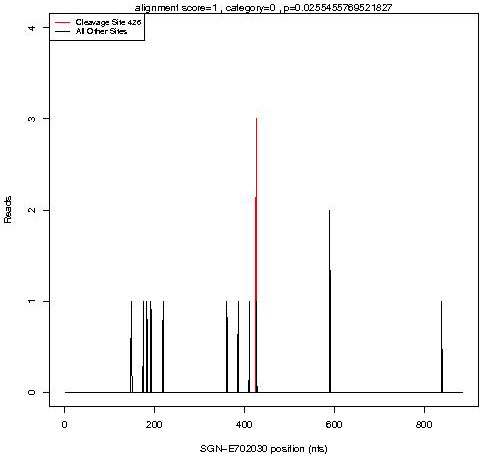

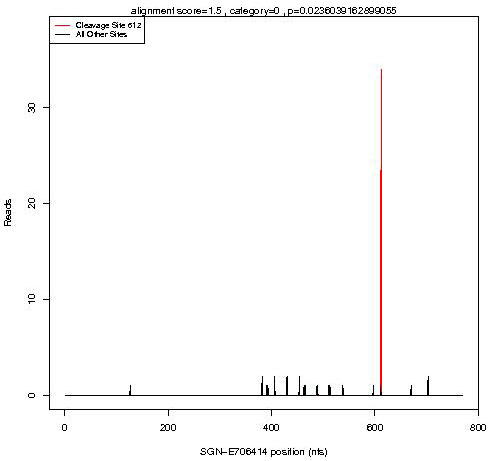


sha-miR160a slicing SGN-E706414 at nt 612

sha-miR160a slicing SGN-E706426 at nt 609


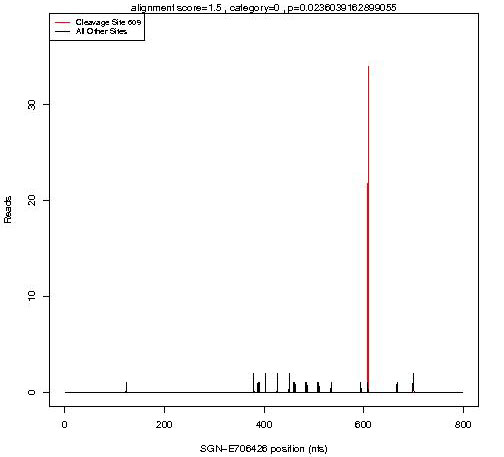


sha-miR166a and sha-miR166b slicing SGN-E316831 at nt 642


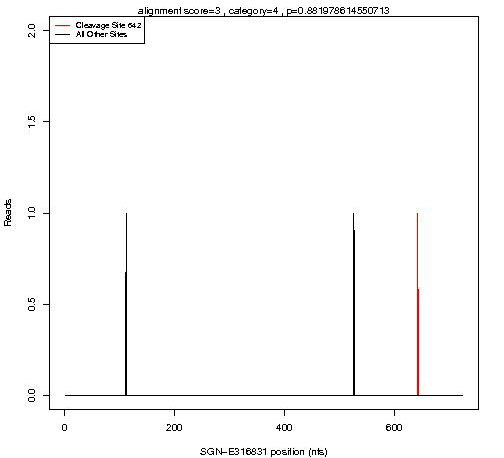

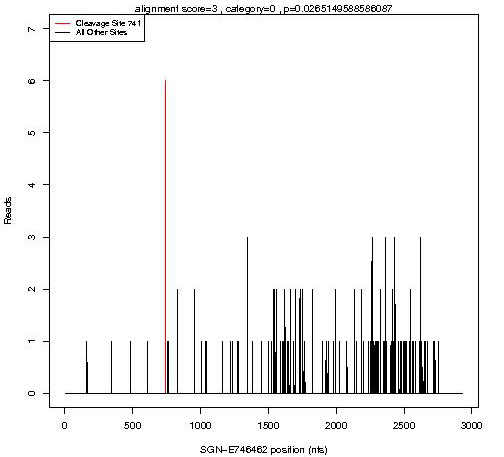


sha-miR166a and sha-miR166b slicingSGN-E746462 at nt 741


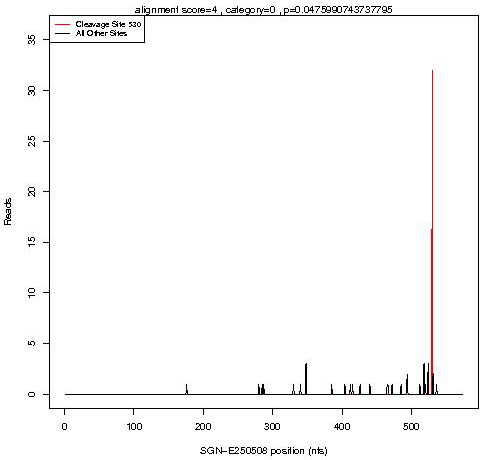


sha-miR167b_nta slicing SGN-E250508 at nt 530

sha-miR167b_nta slicing SGN-E274806 at nt 146


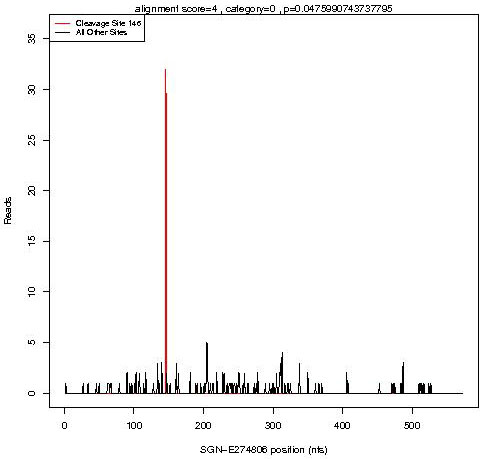


sha-miR167b_nta slicing SGN-E550697 at nt 125


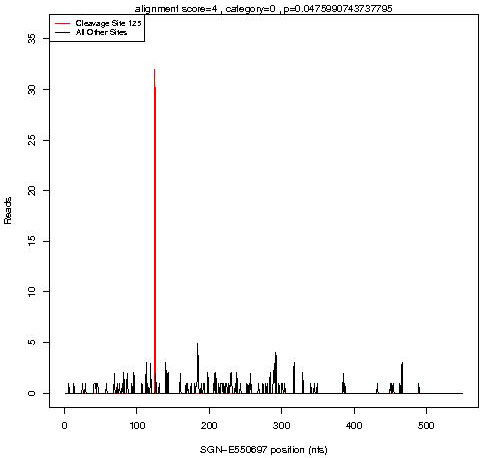

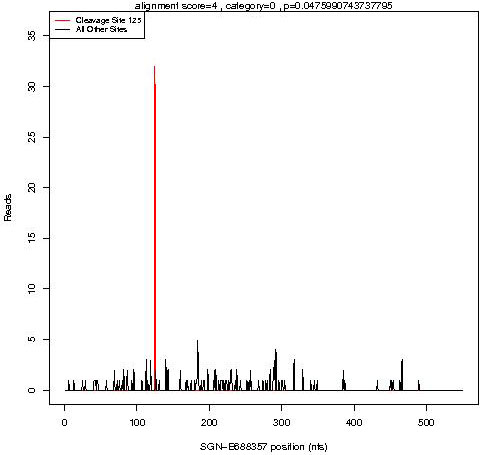


sha-miR167b_nta slicing SGN-E688357 at nt 125

sha-miR167b_nta slicing SGN-E707302 at nt 545


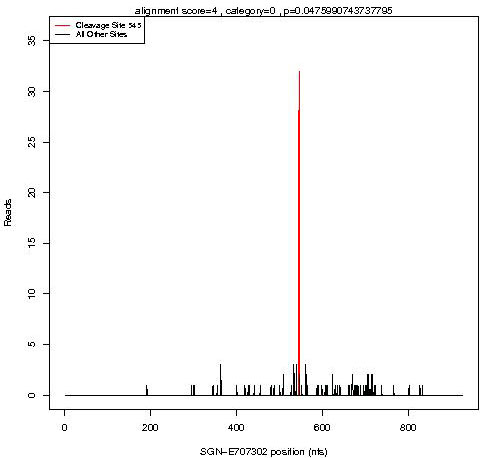


sha-miR168a-5p and sha-miR168b-5p slicingSGN-E740127at nt 517


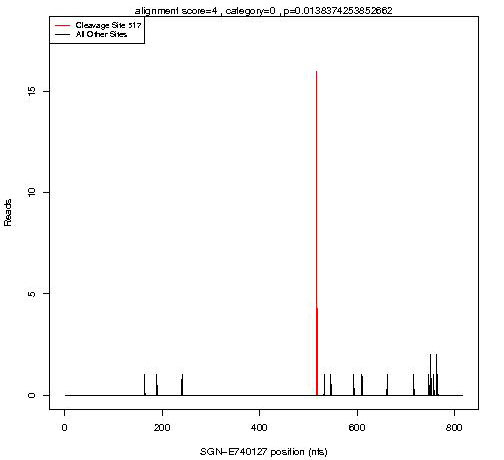


sha-miR171a slicing SGN-E246758 at nt 194


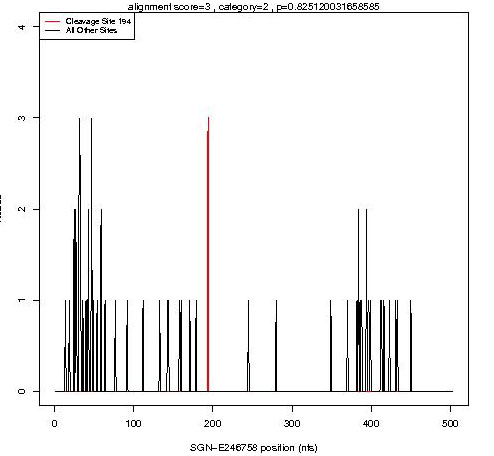

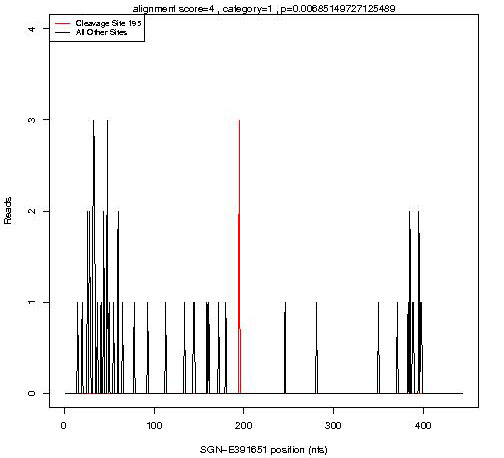


sha-miR171a slicing SGN-E391651 at nt 195

sha-miR171a slicing SGN-E739290 at nt 620


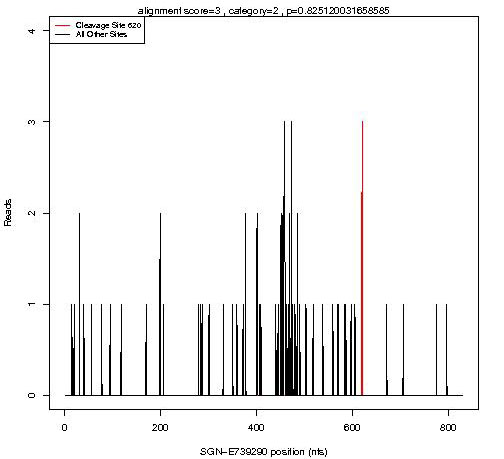


sha-miR171a slicing SGN-E740792 at nt 620


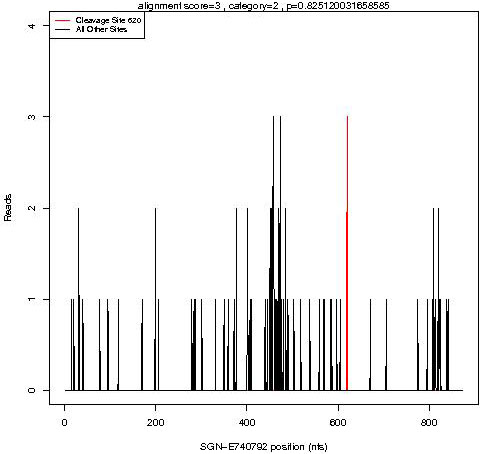

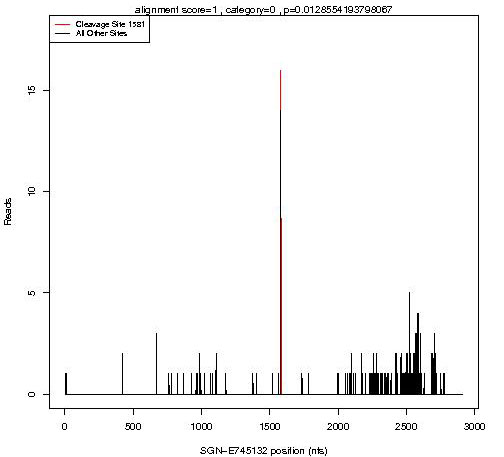


sha-miR171a slicing SGN-E745132 at nt 1581

sha-miR171a_nta slicing SGN-E246758 at nt 194


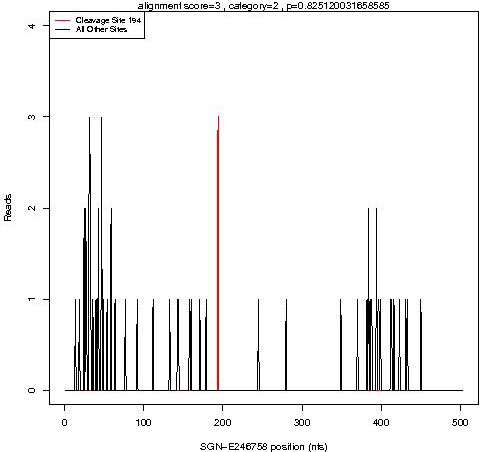

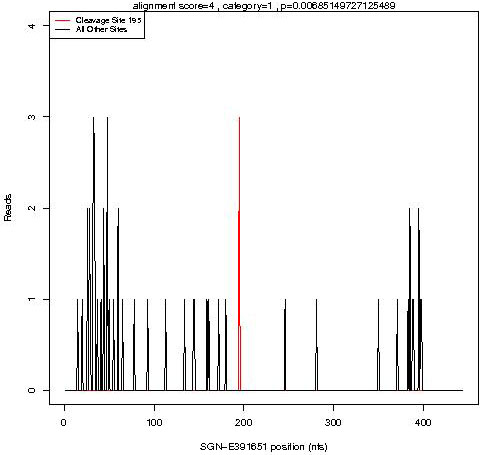


sha-miR171a_nta slicing SGN-E391651 at nt195


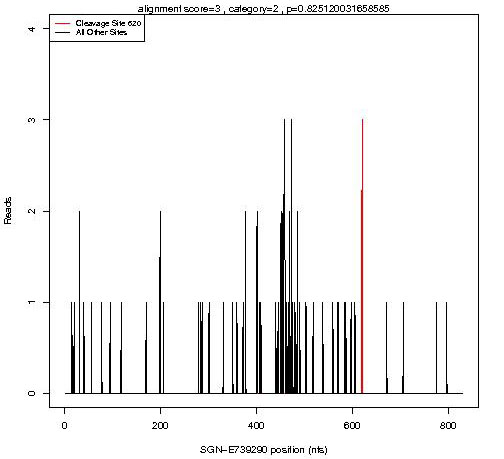


sha-miR171a_nta slicing SGN-E739290 at nt 620

sha-miR171a_nta slicing SGN-E740792 at nt 620


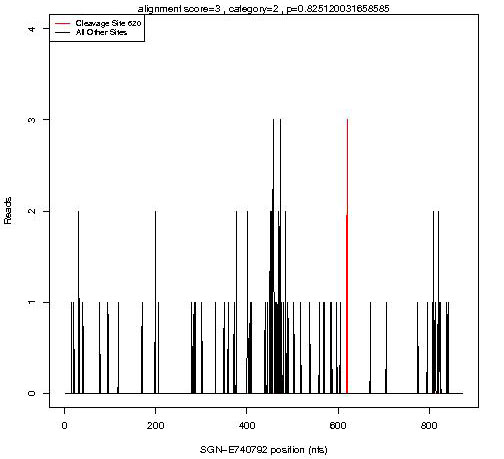

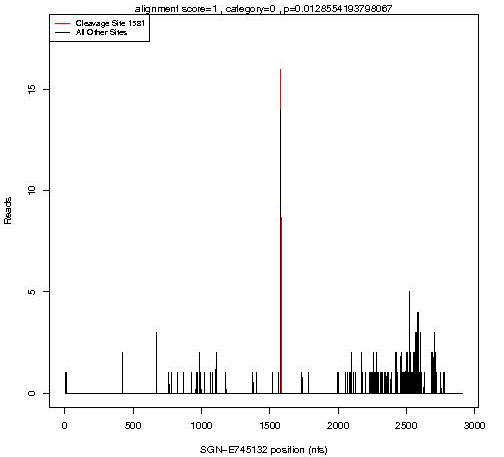


sha-miR171a_nta slicing SGN-E745132 at nt 1581

sha-miR171b-3p_stu slicing SGN-E745132 at nt 1578


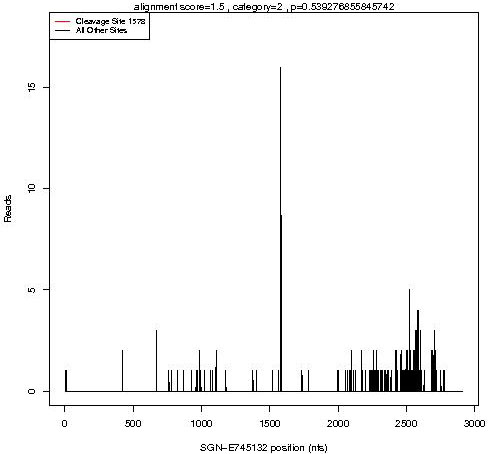


sha-miR171b-p3_mes slicing SGN-E246758 at nt 194


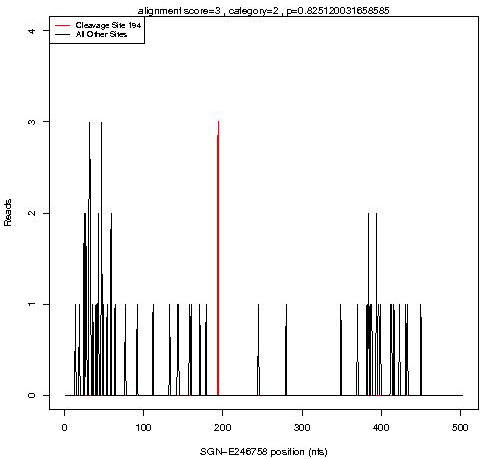

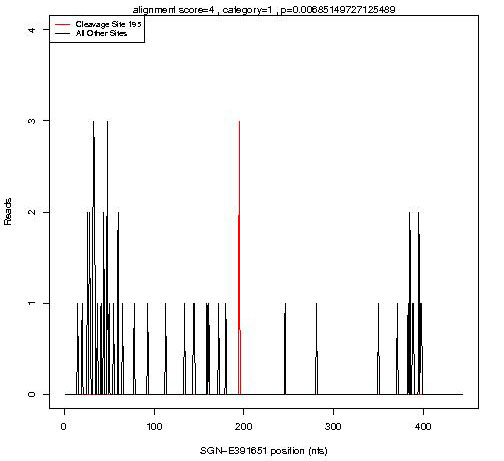


sha-miR171b-p3_mes slicing SGN-E391651 at nt 195

sha-miR171b-p3_mes slicing SGN-E739290 at nt 620


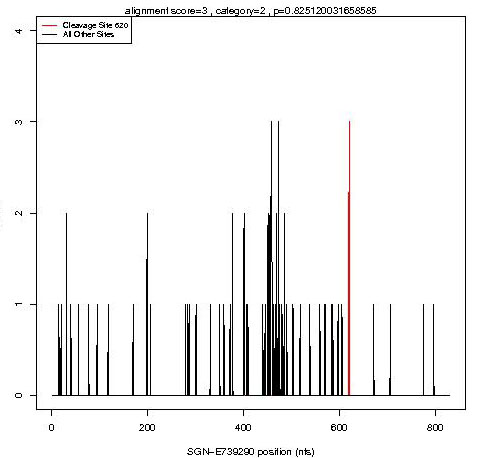


sha-miR171b-p3_mes slicing SGN-E740792 at nt 620


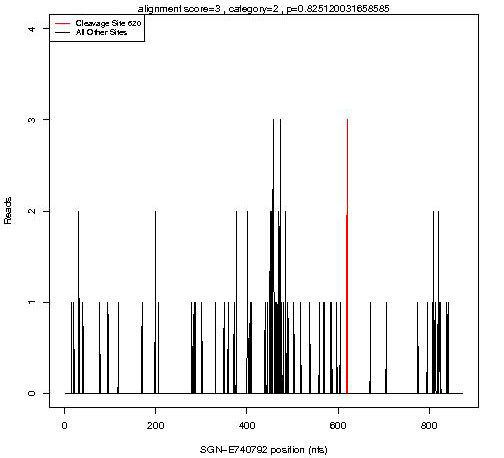


sha-miR171b-p3_mes slicing SGN-E745132 at nt 1581


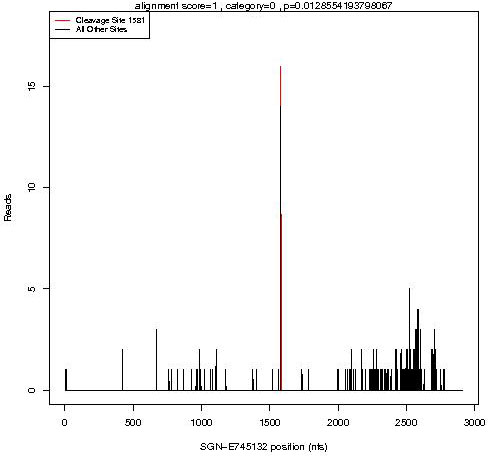


sha-miR171c_mtr slicing SGN-E246758 at nt 194


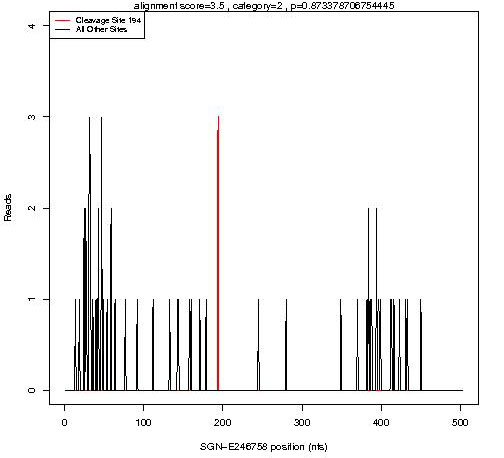


sha-miR171c_mtr slicing SGN-E739290 at nt 620


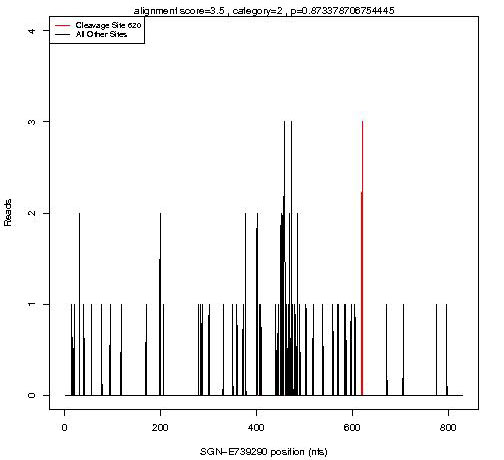

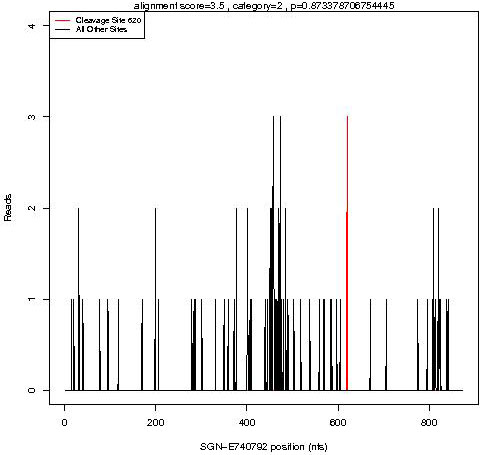


sha-miR171c_mtr slicing SGN-E740792at nt 620


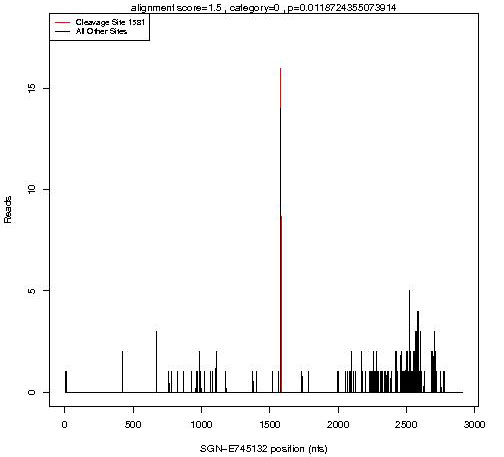


sha-miR171c_mtr slicing SGN-E745132 at nt 1581

sha-miR171c-3p_stu slicing SGN-E246758 at nt 194


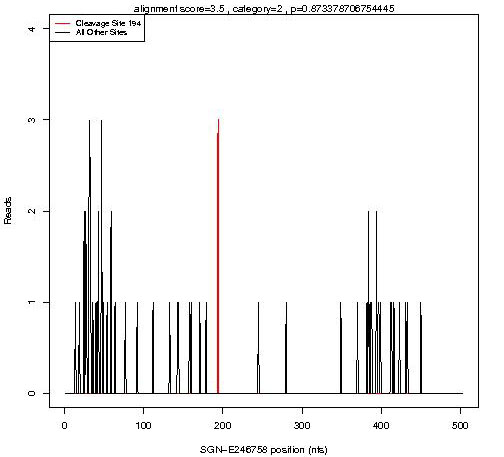

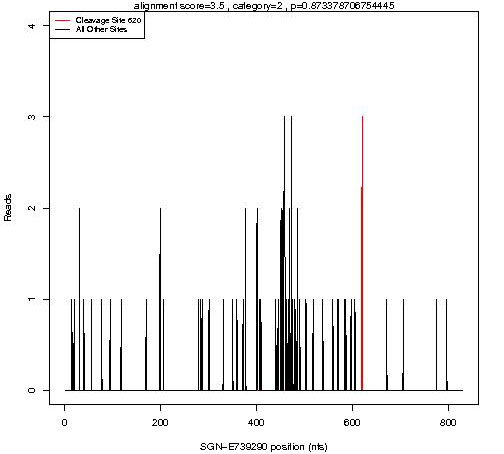


sha-miR171c-3p_stu slicing SGN-E739290 at nt 620

sha-miR171c-3p_stu slicing SGN-E740792 at nt 620


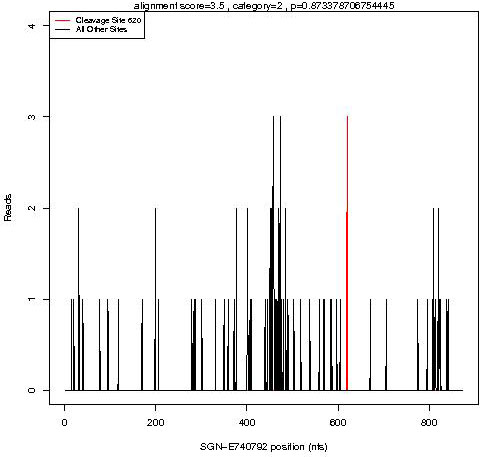


sha-miR171c-3p_stu slicing SGN-E745132 at nt 1581


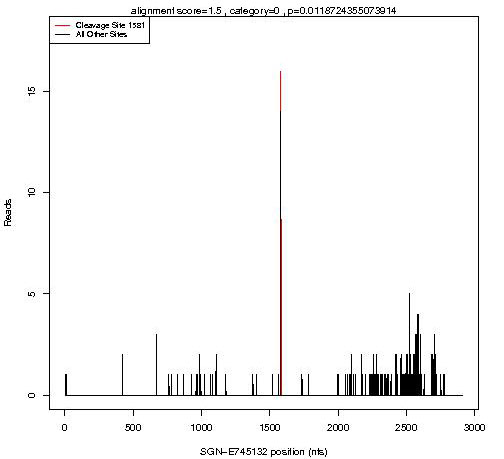


sha-miR171d slicing SGN-E730027 at nt 395


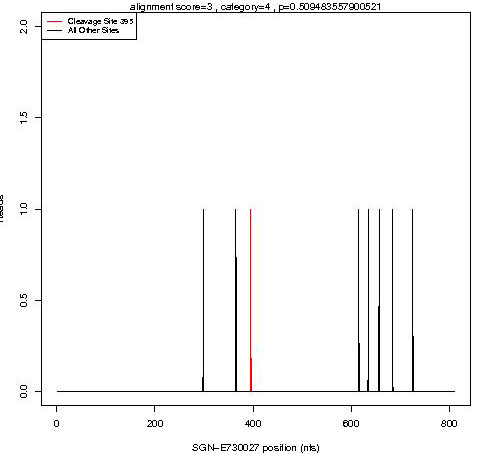


sha-miR171d slicing SGN-E745132 at nt 1578


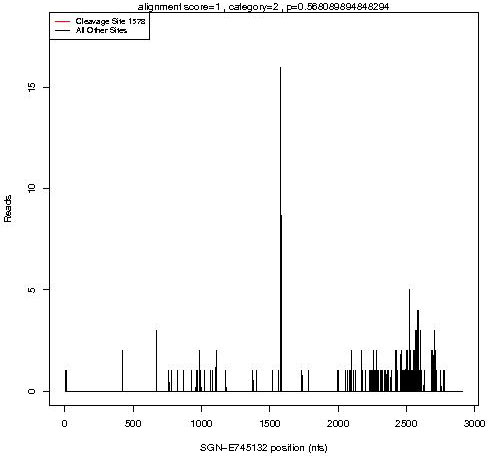

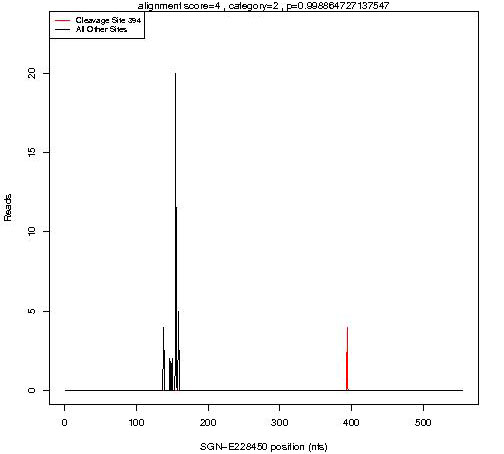


sha-miR172a and sha-miR172b slicing SGN-E228450 at nt 394

sha-miR172a and sha-miR172b slicing SGN-E377981 at nt 631


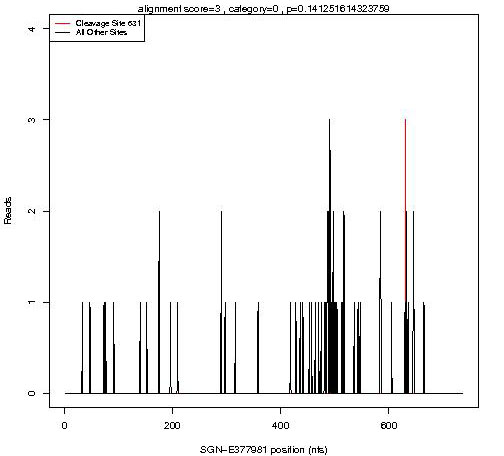


sha-miR172a and sha-miR172b slicing SGN-E745815 at nt 1569


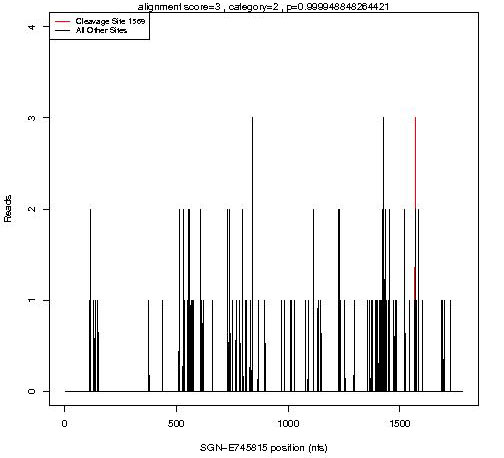

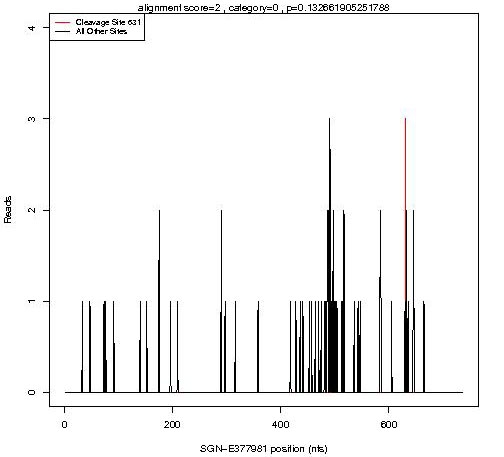


sha-miR172i_nta slicing SGN-E377981 at nt 631

sha-miR172i_nta slicing SGN-E745815 at nt 1569


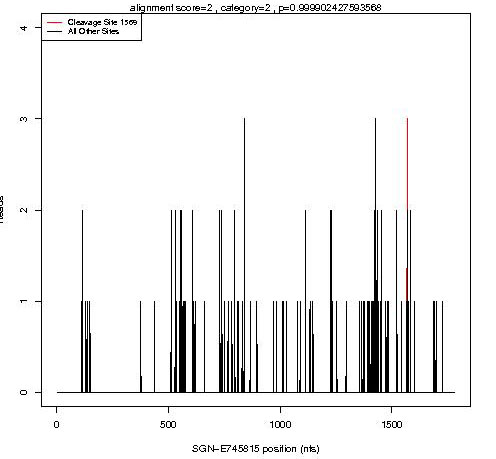


sha-miR172c-3p_aly slicing SGN-E228450 at nt 394


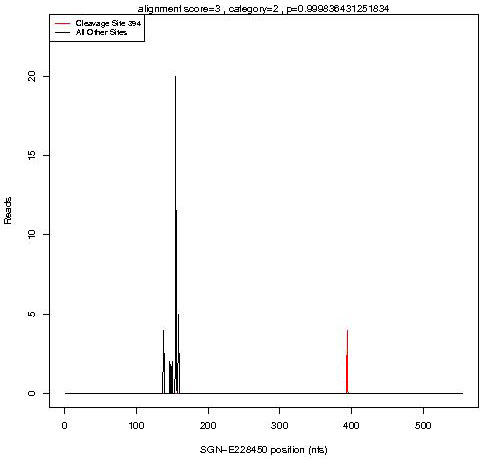


sha-miR172c-3p_aly slicing SGN-E377981 at nt 631


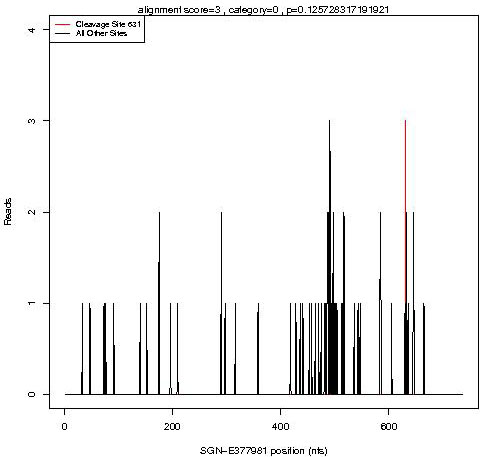

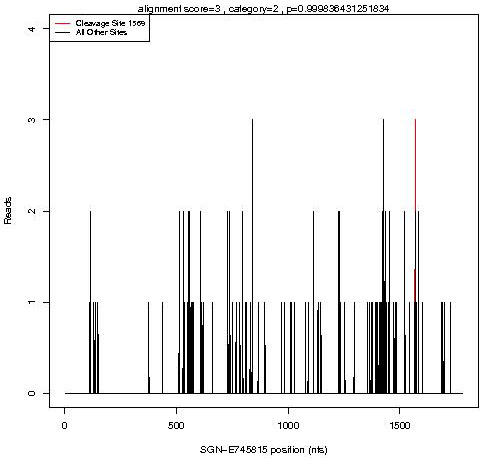


sha-miR172c-3p_aly slicing SGN-E745815 at nt 1569

sha-miR319b_stu slicing SGN-E203353 at nt 502


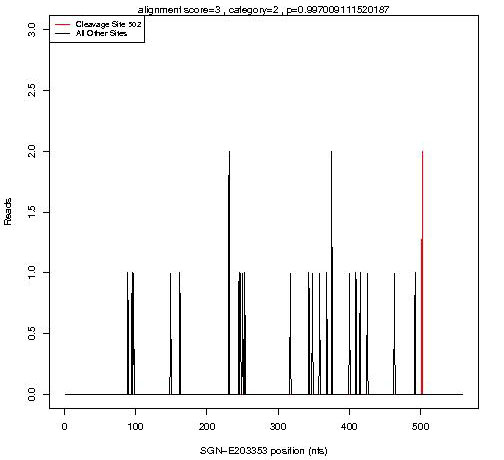


sha-miR319b_stu slicing SGN-E290413 at nt 135


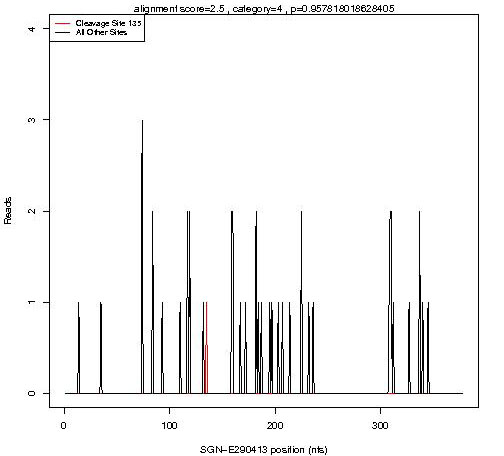

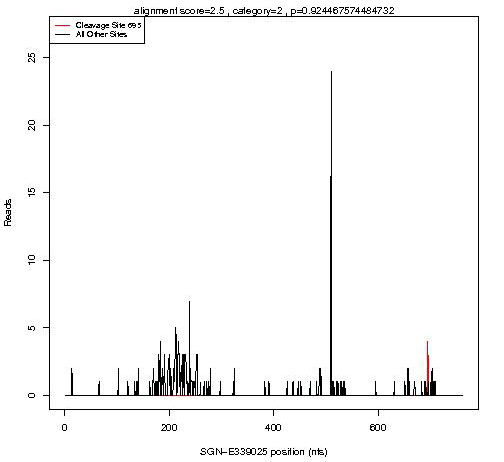


sha-miR319b_stu slicing SGN-E339025 at nt 695

sha-miR319b_stu slicing SGN-E342729 at nt 167


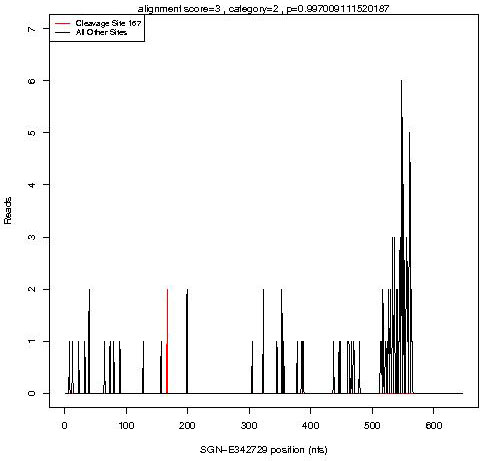


sha-miR319b_stu slicing SGN-E344717 at nt 495


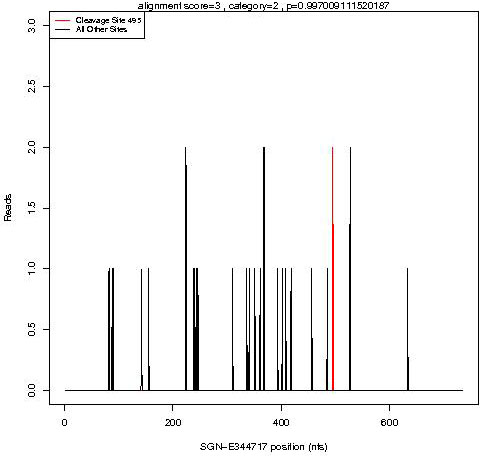

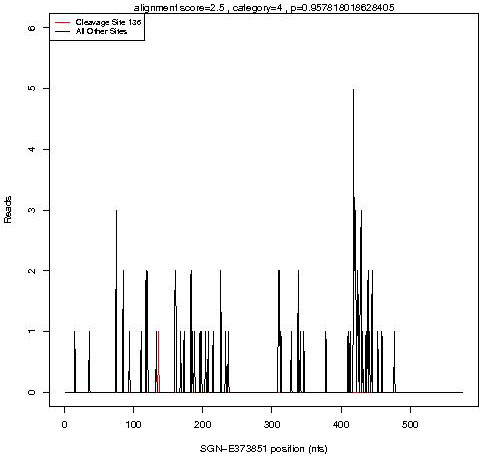


sha-miR319b_stu slicing SGN-E373851 at nt 136

sha-miR319b_stu slicing SGN-E373852 at nt 135


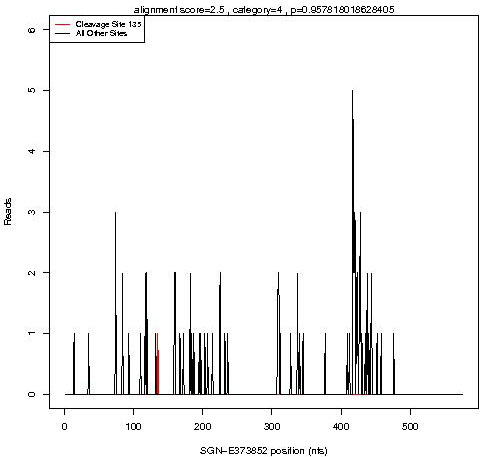


sha-miR319b_stu slicingSGN-E700760 at nt 250


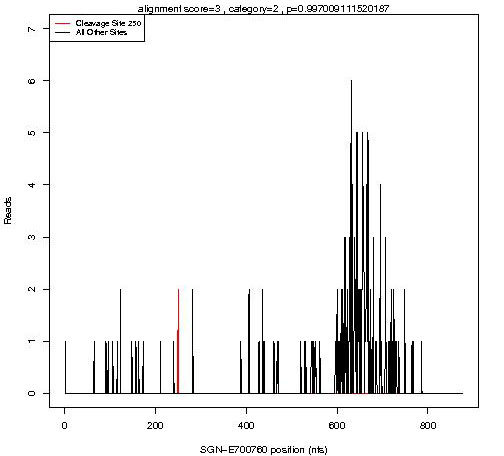


sha-miR319b_stu slicing SGN-E746988 at nt 1367


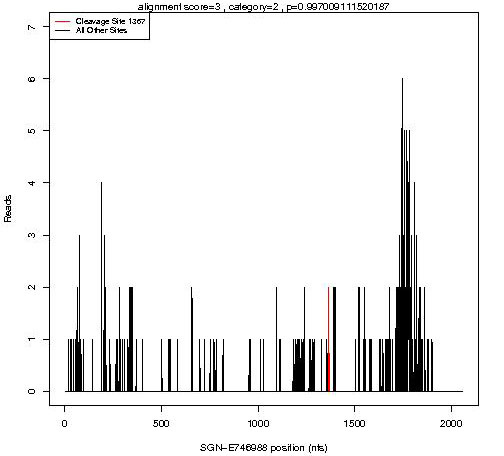


sha-miR319b_stu slicing SGN-E747253 at nt 2209


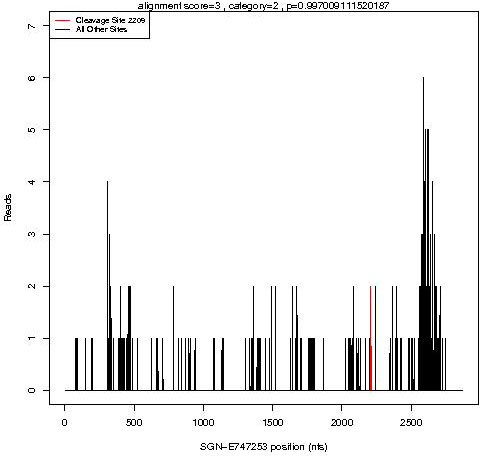


sha-miR393-5p_stu slicing SGN-E541981 at nt 68


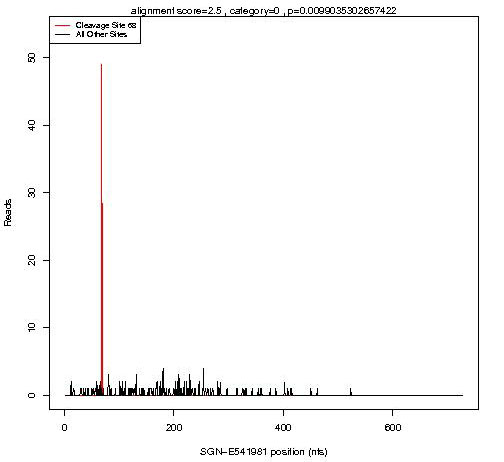


sha-miR396a_nta slicing SGN-E203526 at nt 402


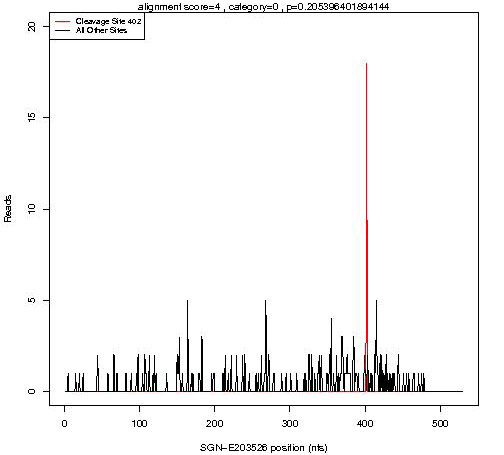


sha-miR396a_nta slicing SGN-E208738 at nt 149


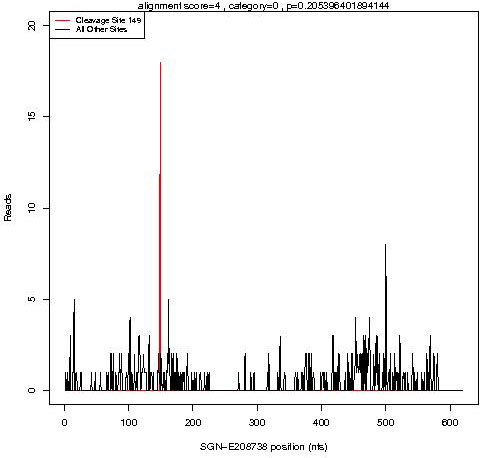

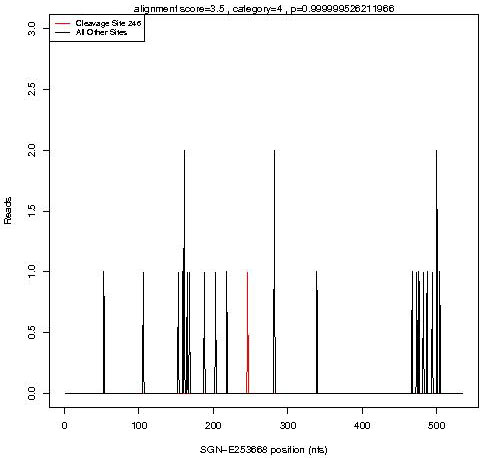


sha-miR396a_nta slicing SGN-E253668 at nt 246

sha-miR396a_nta slicing SGN-E253713 at nt 80


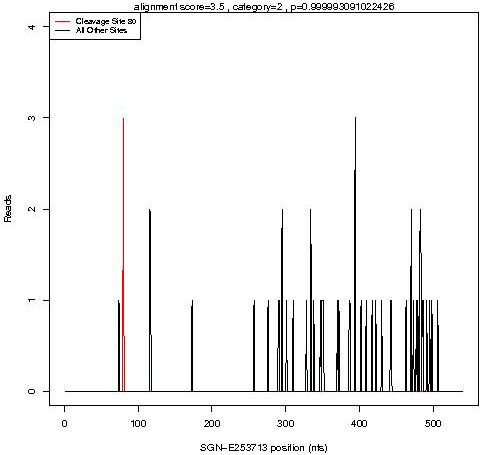


sha-miR396a_nta slicing SGN-E255531 at nt 251


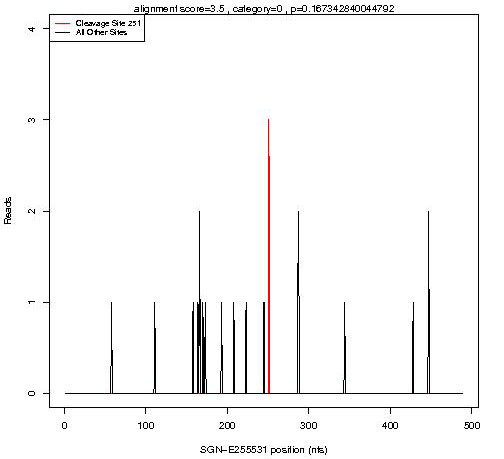


sha-miR396a_nta slicing SGN-E257588 at nt 248


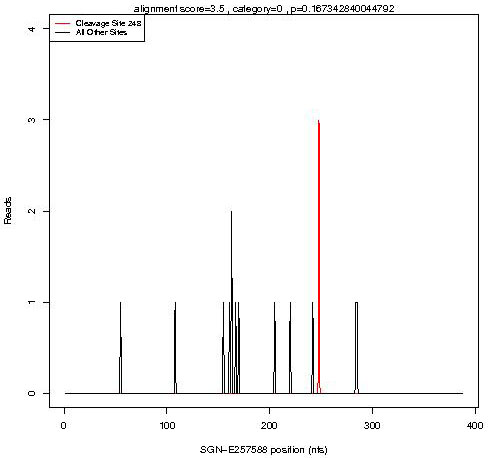


sha-miR396a_nta slicing SGN-E257650 at nt 251


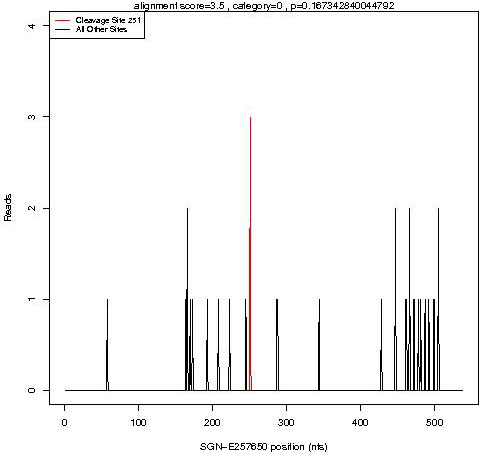

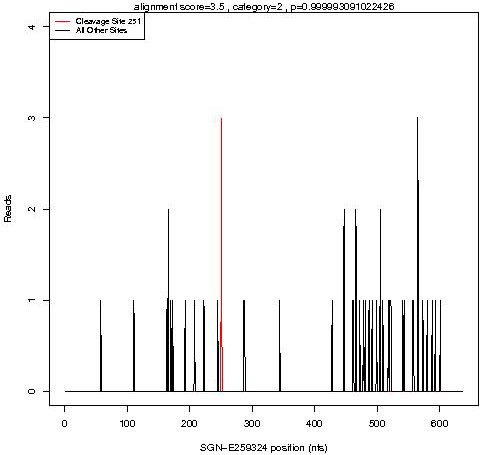


sha-miR396a_nta slicing SGN-E259324 at nt 251

sha-miR396a_nta slicing SGN-E261365 at nt 251


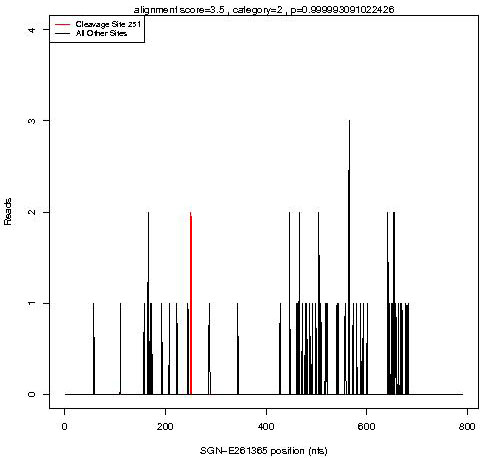


sha-miR396a_nta slicing SGN-E261582at nt 245


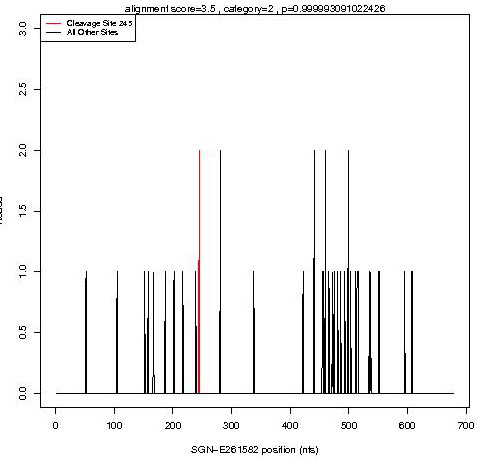


sha-miR396a_nta slicing SGN-E270049 at nt 466


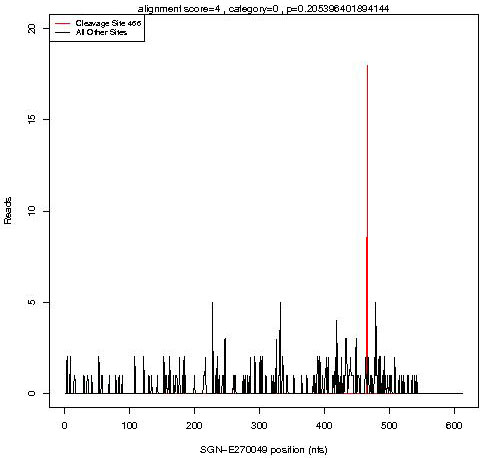


sha-miR396a_nta slicing SGN-E284673 at nt 374

sha-miR396a_nta slicing SGN-E284790 at nt 93

sha-miR396a_nta slicing SGN-E334427 at nt 646

sha-miR396a_nta slicing SGN-E369054 at nt 251

sha-miR396a_nta slicing SGN-E392273 at nt 252

sha-miR396a_nta slicing SGN-E392304 at nt 252

sha-miR396a_nta slicing SGN-E392323 at nt 252

sha-miR396a_nta slicing SGN-E395508 at nt 437

sha-miR396a_nta slicing SGN-E707067 at nt 133

sha-miR396b_nta slicing SGN-E200713 at nt 115

sha-miR396b_nta slicing SGN-E205705 at nt 273

sha-miR396b_nta slicing SGN-E214917 at nt 503

sha-miR396b_nta slicing SGN-E253668 at nt 246

sha-miR396b_nta slicing SGN-E253713 at nt 80

sha-miR396b_nta slicing SGN-E255531 at nt 251

sha-miR396b_nta slicing SGN-E257588 at nt 248

sha-miR396b_nta slicing SGN-E257650 at nt 251

sha-miR396b_nta slicing SGN-E259324 at nt 251

sha-miR396b_nta slicing SGN-E261365 at nt 251

sha-miR396b_nta slicing SGN-E261582 at nt 245

sha-miR396b_nta slicing SGN-E265911 at nt 504

sha-miR396b_nta slicing SGN-E266983 at nt 356

sha-miR396b_nta slicing SGN-E298573 at nt 214

sha-miR396b_nta slicing SGN-E299086 at nt 100

sha-miR396b_nta slicing SGN-E301892 at nt 434

sha-miR396b_nta slicing SGN-E304743 at nt 368

sha-miR396b_nta slicing SGN-E306168 at nt 494

sha-miR396b_nta slicing SGN-E310217 at nt 162

sha-miR396b_nta slicing SGN-E320077 at nt 612

sha-miR396b_nta slicing SGN-E322663 at nt 499

sha-miR396b_nta slicing SGN-E332208 at nt 496

sha-miR396b_nta slicing SGN-E334682 at nt 499

sha-miR396b_nta slicing SGN-E369054 at nt 251

sha-miR396b_nta slicing SGN-E392273 at nt 252

sha-miR396b_nta slicing SGN-E392304 at nt 252

sha-miR396b_nta slicing SGN-E392323 at nt 252

sha-miR396b_nta slicing SGN-E744501 at nt 112

sha-miR396b_nta slicing SGN-E744974 at nt 500

sha-miR396b_nta slicing SGN-E747256 at nt 519

sha-miR396c_nta slicing SGN-E200713 at nt 115

sha-miR396c_nta slicing SGN-E205705 at nt 273

sha-miR396c_nta slicing SGN-E214917 at nt 503

sha-miR396c_nta slicing SGN-E253668 at nt 246

sha-miR396c_nta slicing SGN-E253713 at nt 80

sha-miR396c_nta slicing SGN-E255531 at nt 251

sha-miR396c_nta slicing SGN-E257588 at nt 248

sha-miR396c_nta slicing SGN-E257650 at nt 251

sha-miR396c_nta slicing SGN-E259324 at nt 251

sha-miR396c_nta slicing SGN-E261365 at nt 251

sha-miR396c_nta slicing SGN-E261582 at nt 245

sha-miR396c_nta slicing SGN-E265911 at nt 504

sha-miR396c_nta slicing SGN-E266983 at nt 356

sha-miR396c_nta slicing SGN-E298573 at nt 214

sha-miR396c_nta slicing SGN-E299086 at nt 100

sha-miR396c_nta slicing SGN-E301892 at nt 434

sha-miR396c_nta slicing SGN-E304743 at nt 368

sha-miR396c_nta slicing SGN-E306168 at nt 494

sha-miR396c_nta slicing SGN-E310217 at nt 162

sha-miR396c_nta slicing SGN-E320077 at nt 612

sha-miR396c_nta slicing SGN-E322663 at nt 499

sha-miR396c_nta slicing SGN-E332208at nt 496

sha-miR396c_nta slicing SGN-E334682 at nt 499

sha-miR396c_nta slicing SGN-E369054 at nt 251

sha-miR396c_nta slicing SGN-E392273 at nt 252

sha-miR396c_nta slicing SGN-E392304 at nt 252

sha-miR396c_nta slicing SGN-E392323 at nt 252

sha-miR396c_nta slicing SGN-E744501 at nt 112

sha-miR396c_nta slicing SGN-E744974 at nt 500

sha-miR396c_nta slicing SGN-E747256 at nt 519

sha-miR398_nta slicing SGN-E230955 at nt 33

sha-miR398_nta slicing SGN-E232089 at nt 40

sha-miR398_nta slicing SGN-E233097 at nt 40

sha-miR398_nta slicing SGN-E233685 at nt 40

sha-miR398_nta slicing SGN-E235893 at nt 40

sha-miR398_nta slicing SGN-E240585 at nt 33

sha-miR398_nta slicing SGN-E242651at nt 20

sha-miR398_nta slicing SGN-E246110 at nt 22

sha-miR398_nta slicing SGN-E249213 at nt 40

sha-miR398_nta slicing SGN-E252035 at nt 33

sha-miR398_nta slicing SGN-E255914 at nt 39

sha-miR398_nta slicing SGN-E257573 at nt 40

sha-miR398_nta slicing SGN-E260931 at nt 40

sha-miR398_nta slicing SGN-E261584 at nt 173

sha-miR398_nta slicing SGN-E261859 at nt 38

sha-miR398_nta slicing SGN-E262708 at nt 40

sha-miR398_nta slicing SGN-E279564 at nt 40

sha-miR398_nta slicing SGN-E291338 at nt 40

sha-miR398_nta slicing SGN-E295417 at nt 62

sha-miR398_nta slicing SGN-E296409 at nt 33

sha-miR398_nta slicing SGN-E298678 at nt 53

sha-miR398_nta slicing SGN-E304459 at nt 34

sha-miR398_nta slicing SGN-E317833 at nt 29

sha-miR398_nta slicing SGN-E321152 at nt 12

sha-miR398_nta slicing SGN-E322178 at nt 33

sha-miR398_nta slicing SGN-E323027 at nt 18

sha-miR398_nta slicing SGN-E332721 at nt 29

sha-miR398_nta slicing SGN-E334772at nt 24

sha-miR398_nta slicing SGN-E335996 at nt 29

sha-miR398_nta slicing SGN-E337189 at nt 39

sha-miR398_nta slicing SGN-E338797 at nt 26

sha-miR398_nta slicing SGN-E340252 at nt 40

sha-miR398_nta slicing SGN-E344633 at nt 35

sha-miR398_nta slicing SGN-E344668 at nt 16

sha-miR398_nta slicing SGN-E345942 at nt 35

sha-miR398_nta slicing SGN-E346479 at nt 40

sha-miR398_nta slicing SGN-E372953 at nt 34

sha-miR398_nta slicing SGN-E552057 at nt 44

sha-miR398_nta slicing SGN-E552774 at nt 41

sha-miR398_nta slicing SGN-E708132 at nt 35

sha-miR398_nta slicing SGN-E711018 at nt 36

sha-miR398_nta slicing SGN-E712604 at nt 33

sha-miR398_nta slicing SGN-E713582 at nt 23

sha-miR398_nta slicing SGN-E713661 at nt 36

sha-miR398_nta slicing SGN-E715403 at nt 32

sha-miR398_nta slicing SGN-E716166 at nt 27

sha-miR398_nta slicing SGN-E716292 at nt 34

sha-miR398_nta slicing SGN-E716330 at nt 34

sha-miR398_nta slicing SGN-E717497 at nt 87

sha-miR398_nta slicing SGN-E717993 at nt 84

sha-miR398_nta slicing SGN-E718214 at nt 75

sha-miR398_nta slicing SGN-E718433 at nt 55

sha-miR398_nta slicing SGN-E718448 at nt 73

sha-miR398_nta slicing SGN-E718876 at nt 73

sha-miR398_nta slicing SGN-E720598 at nt 55

sha-miR398_nta slicing SGN-E721914 at nt 84

sha-miR398_nta slicing SGN-E722420 at nt 55

sha-miR398_nta slicing SGN-E722620 at nt 68

sha-miR398_nta slicing SGN-E724044 at nt 65

sha-miR398_nta slicing SGN-E724642 at nt 78

sha-miR398_nta slicing SGN-E724744 at nt 79

sha-miR398_nta slicing SGN-E726505at nt 41

sha-miR408_nta slicing SGN-E353197 at nt 33

sha-miR408_nta slicing SGN-E354518 at nt 30

sha-miR408_nta slicing SGN-E355169 at nt 35

sha-miR408_nta slicing SGN-E356803 at nt 35

sha-miR408_nta slicing SGN-E552215 at nt 60

sha-miR482a_ghr slicing SGN-E236981 at nt 144

sha-miR482a_ghr slicing SGN-E352352 at nt 100

sha-miR482a_ghr slicing SGN-E355276 at nt 83

sha-miR482a_ghr slicing SGN-E704204 at nt 731

sha-miR482a_ghr slicing SGN-E707598 at nt 727

sha-miR482a_ghr slicing SGN-E746410 at nt 1046

sha-miR482b slicing SGN-E739892 at nt 236

sha-miR482b slicing SGN-E740719 at nt 477

sha-miR827_nta slicing SGN-E309503 at nt 235

sha-miR827_ntaslicing SGN-E331370 at nt 242

sha-miR5301-p3 slicing SGN-E236981 at nt 144

sha-miR5301-p3 slicing SGN-E352352 at nt 100

sha-miR5301-p3 slicing SGN-E355276 at nt 83

sha-miR5301-p3 slicing SGN-E704204 at nt 731

sha-miR5301-p3 slicing SGN-E707598 at nt 727

sha-miR5301-p3 slicing SGN-E746410 at nt 1046

sha-miR6024 slicing SGN-E248561at nt 334

sha-miR6024 slicing SGN-E376022 at nt 553

sha-miR6025_stu slicing SGN-E272042 at nt 294

sha-miR6025_stu slicing SGN-E354630 at nt 432

sha-miR6025_stu slicing SGN-E370409 at nt 238

sha-miR6025_stu slicing SGN-E370410 at nt 294

sha-miR6025_stu slicing SGN-E389714 at nt 446

sha-miR6025_stu slicing SGN-E550079 at nt 447

sha-miR6025_stu slicing SGN-E550285 at nt 447

sha-miR6025_stu slicing SGN-E550623 at nt 336

sha-miR6025_stu slicing SGN-E551268 at nt 295

sha-miR6025_stu slicing SGN-E551398 at nt 295

sha-miR6025_stu slicing SGN-E688258 at nt 295

sha-miR6025_stu slicing SGN-E688259 at nt 336

sha-miR6025_stu slicing SGN-E688271at nt 295

sha-miR6025_stu slicing SGN-E711212 at nt 367

sha-miR6027 slicing SGN-E204998 at nt 145

sha-miR6027 slicing SGN-E398442 at nt 146

PC-46-5p slicing SGN-E548613 at nt 625

PC-58-5p slicing SGN-E232041at nt 121

PC-58-5p slicing SGN-E233471 at nt 121

PC-58-5p slicing SGN-E236245 at nt 121

PC-58-5p slicing SGN-E244660 at nt 121

PC-58-5p slicing SGN-E378026 at nt 121

PC-75-3p slicing SGN-E263621 at nt 661

PC-75-3p slicing SGN-E355515 at nt 467

PC-75-3p slicing SGN-E356830 at nt 467

PC-75-3p slicing SGN-E356831 at nt 653

PC-75-3p slicing SGN-E707134 at nt 782

PC-93-3p slicing SGN-E226448at nt 43

PC-93-3p slicing SGN-E287356 at nt 297

PC-93-3p slicing SGN-E703722 at nt 573

PC-93-3p slicing SGN-E708964 at nt 556

PC-93-3p slicing SGN-E713893 at nt 558

PC-102-5p slicing SGN-E233896 at nt 98

PC-102-5p slicing SGN-E350418 at nt 321

PC-102-5p slicing SGN-E351849 at nt 415

PC-102-5p slicing SGN-E352584 at nt 415

PC-102-5p slicing SGN-E353944 at nt 319

PC-102-5p slicing SGN-E701432 at nt 798

PC-102-5p slicing SGN-E705552 at nt 699

PC-102-5p slicing SGN-E713099 at nt 271

PC-102-5p slicing SGN-E713896 at nt 162

PC-117-5p slicing SGN-E720902 at nt 452

PC-146-5p slicing SGN-E208808 at nt 380

PC-146-5p slicing SGN-E254753 at nt 507

PC-146-5p slicing SGN-E262673 at nt 507

PC-146-5p slicing SGN-E269493 at nt 484

PC-146-5p slicing SGN-E303554 at nt 518
