# Supplementary figures and images for: Identification of chilling stress-responsive tomato microRNAs and their target genes by high-throughput sequencing and degradome analysis
Source: BMC Genomics. 2014 Dec 17;15(1):1130. doi: 10.1186/1471-2164-15-1130 (PMC4377850; doi:10.1186/1471-2164-15-1130)

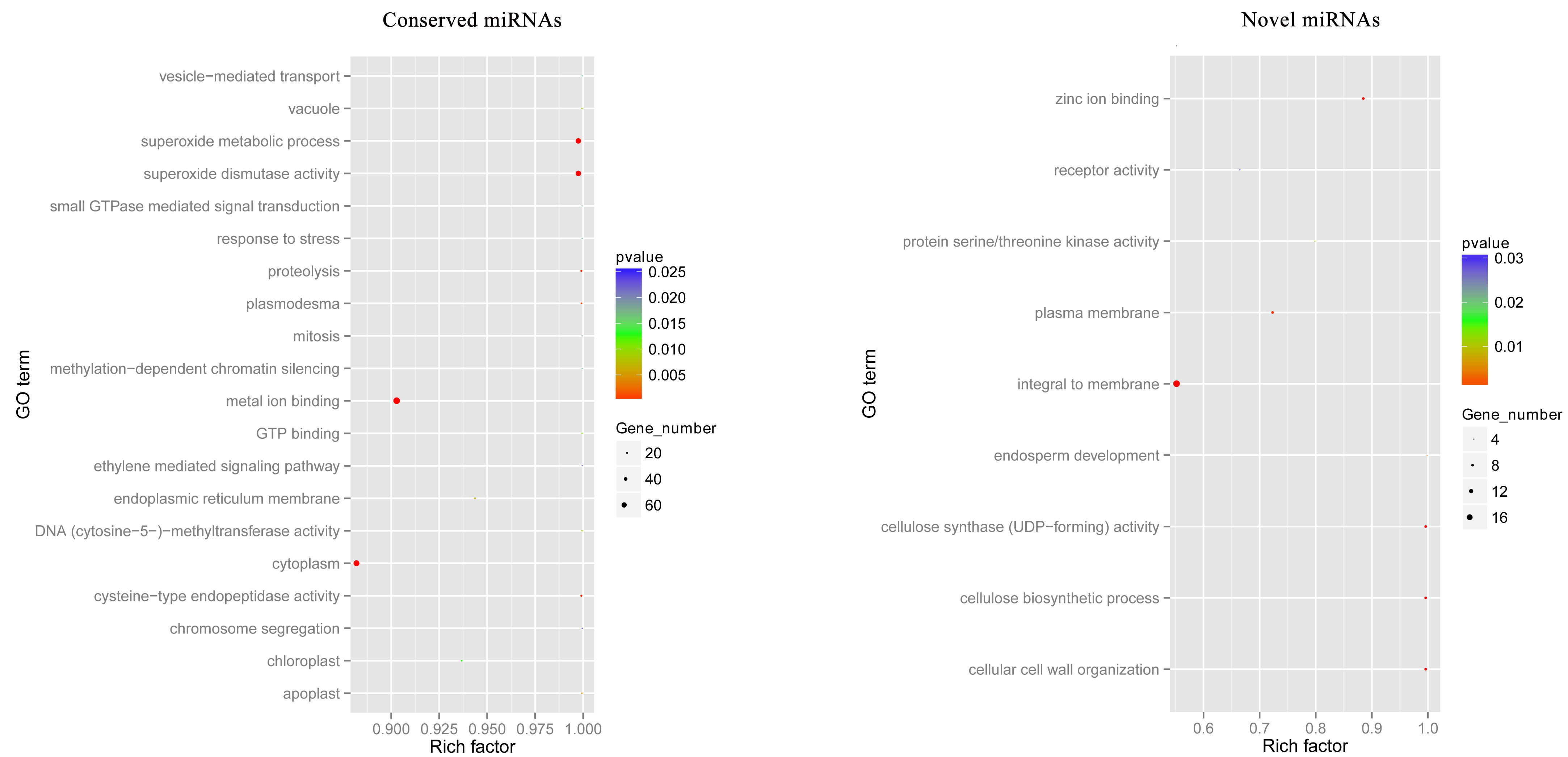

Supplement: Supplementary file 8 — Additional file 8: Figure S5: - GO enrichment analysis of the functions of target genes cleaved by conserved and novel miRNAs. (JPEG 728 KB) [file 12864_2014_6877_MOESM8_ESM.jpeg]
